# Venetoclax resistance in preclinical KMT2A-rearranged acute lymphoblastic leukemia models is characterized by high inter- and intra-model heterogeneity

**DOI:** 10.1038/s41698-025-01249-1

**Published:** 2025-12-29

**Authors:** Anna Richter, Lea Kinsky, Sandra Lange, Nares Trakooljul, Frieder Hadlich, Anett Sekora, Gudrun Knuebel, Saskia Krohn, Christian Schmidt, Michelle Busch, Tim Schreiber, Simone Kumstel, Klaus Wimmers, Hugo Murua Escobar, Christian Junghanss

**Affiliations:** 1 Department of Internal Medicine, Clinic for Hematology, Hemostasis, Oncology, Stem Cell Therapy and Palliative Medicine, Rostock University Medical Center, Ernst-Heydemann-Str. 6, 18057 Rostock, Germany; 2 Research Institute for Farm Animal Biology, Wilhelm-Stahl-Allee 2, 18196 Dummerstorf, Germany; 3 Institute of Medical Genetics, Rostock University Medical Center, Ernst-Heydemann-Str. 8, 18057 Rostock, Germany; 4 Rudolf-Zenker Institute of Experimental Surgery, Rostock University Medical Center, Schillingallee 69a, 18057 Rostock, Germany

**Keywords:** Cancer, Cell biology, Drug discovery, Oncology

## Abstract

The BCL-2 inhibitor venetoclax (VEN) has emerged as an important therapeutic backbone for hematological malignancies, but secondary resistance is a major challenge. In acute lymphoblastic leukemia (ALL), early clinical trials promise high efficacy as well. However, relapse is observed frequently, and so far, only a few resistance-inducing mechanisms have been discussed. We employed KMT2A-rearranged ALL cell lines and xenograft models to elucidate mechanisms of VEN resistance. Targeted DNA and mRNA sequencing, single-cell mRNAseq, as well as protein expression analyses were conducted. All models initially responded well but finally displayed secondary resistance. Novel as well as previously known pathogenic variants in tumor suppressor *TP53* as well as pro-apoptotic molecule *BAX*, but not *BCL2*, were observed. Gene and protein expression studies demonstrated multifarious changes in resistant cells, with high inter- and intra-model heterogeneity. Finally, single-cell RNA sequencing revealed a likely contribution of the tumor microenvironment in the development of VEN resistance, as indicated by modulation of genes involved in cell-cell interaction and humoral signaling in resistance-specific clusters. Our data demonstrate and characterize the rise of VEN resistance in KMT2A-rearranged ALL models, suggesting that relapse must be expected in the clinical setting and that multifactorial processes are involved in this process.

## Introduction

In recent years, the therapy of hematological malignancies has progressed immensely, for example, by the inclusion of immunotherapeutic approaches like bispecific antibodies^1^ and chimeric antigen receptor-expressing T-cells^2^, or the development of tyrosine kinase inhibitors such as Imatinib^3^. Blasts often exhibit high expression of the anti-apoptotic molecule BCL-2, which is a main member of the intrinsic apoptosis cascade and blocks cell death in leukemic cells^4^. The BCL-2 inhibitor Venetoclax (VEN) has demonstrated great clinical efficacy in the treatment of acute myeloid leukemia (AML) and chronic lymphoblastic leukemia (CLL) and is now part of standard care^5,6^. However, the majority of patients finally relapse with secondary VEN resistance. Interestingly, molecular and cellular patterns of resistant cells differ greatly between these two entities: While most of the VEN-resistant CLL cells harbor *BCL2* mutations^7^, these were usually not observed in AML cases. Instead, the molecular landscape in VEN-resistant AML cells is more heterogeneous, including mutations in pro-apoptotic signaling molecules *BAX* or *BAK1*, tumor suppressor TP53, or alterations of the *FLT3* ITD domain. Also, gene and protein expression profiles of BCL-2 family members and drug transporter proteins can be deregulated^8–12^. Consequently, clinical coping strategies vary between CLL and AML.

In acute B-lymphoblastic leukemia (B-ALL), VEN is not yet standard of care, but has been evaluated in several clinical studies. Some early phase 1/2 trials^13,14^ as well as preclinical investigation from our^15^ and other labs^16,17^ hint at promising response rates. Secondary VEN resistance was frequently observed in both clinical trials, but no molecular analyses were conducted in order to characterize the underlying mechanisms. Therefore, potential mechanisms of VEN resistance in ALL remain to be elucidated to identify combination strategies to avoid resistance development, detect arising resistance as early as possible or eradicate persisting clones.

To address this important subject, we used in vitro and in vivo B-ALL model systems to understand and characterize the development of VEN resistance. All analyzed models initially responded well to VEN but ultimately ended up in secondary resistance. Genetic, transcriptional and protein profiling revealed high inter- and intra-model heterogeneity, suggesting multifarious mechanisms of VEN resistance.

## Results

### Continuous VEN application effectively abrogates leukemic blasts but induces resistance in vitro and in vivo

**Fig. 1:**
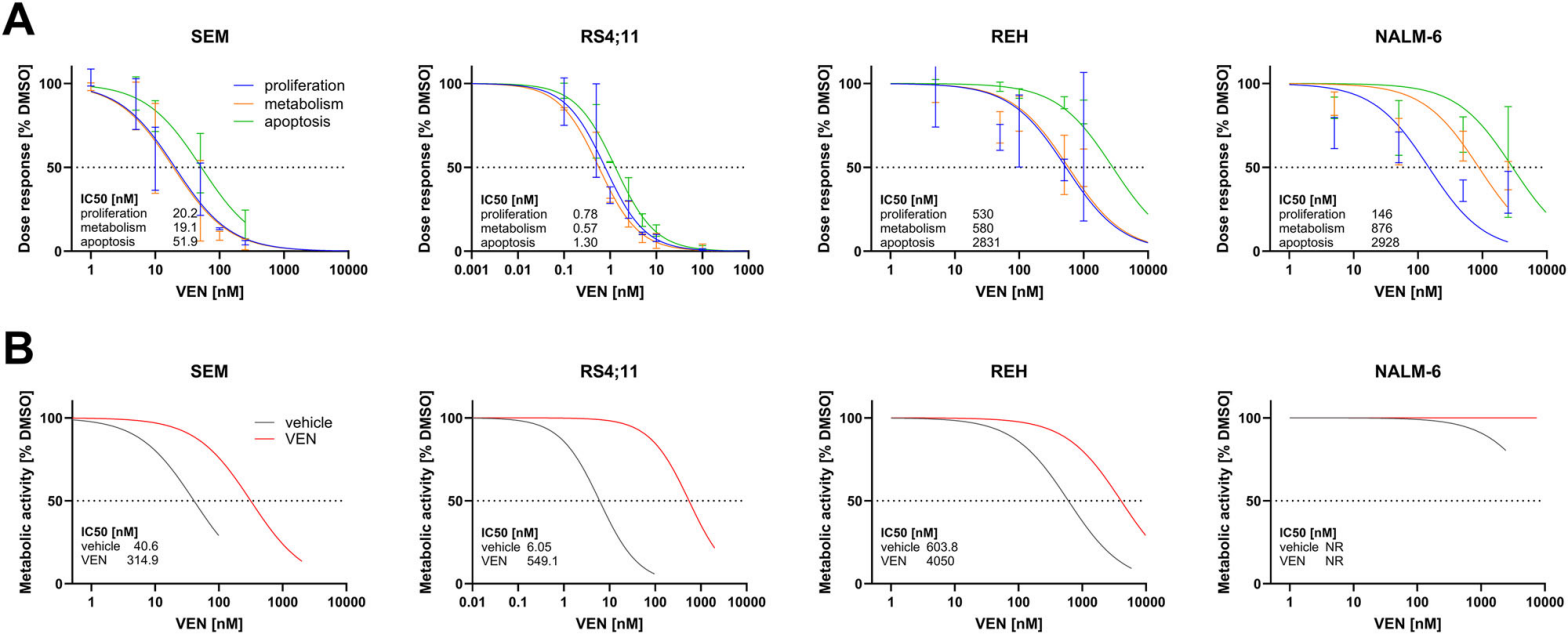
Induction of VEN resistance in B-ALL cell lines. **A** Determination of IC50 concentrations for B-ALL cell lines SEM, RS4;11, REH and NALM-6 using trypan blue staining and subsequent cell counting (proliferation), WST-1 assay (metabolic activity) and annexin V-FITC/propidium iodide staining (apoptosis). Nonlinear fitting of 2-4 individual biological replicates. **B** Cells were continuously incubated with increasing concentrations of VEN and the metabolic activity was assessed by WST-1 proliferation assay. Dose-response curves of vehicle-incubated cells (gray) and VEN-incubated cells (red) are depicted in joint diagrams. IC50 values were calculated for both settings. NR, not reached.

To assess resistance mechanisms, we first checked the basal IC50 concentrations of the B-ALL cell lines SEM, RS4;11, REH and NALM-6 using proliferation, metabolic activity and apoptosis assays, demonstrating response to low nanomolar concentrations in SEM and RS4;11 cells and intrinsic resistance in the REH and NALM-6 cell lines (Fig. 1A). Continuous incubation with increasing concentrations of VEN induced secondary resistance indicated by elevated IC50 doses (Fig. 1B). Cell lines REH and NALM-6, which were considered intrinsically resistant due to high basal IC50 concentrations, still presented with reduced response rates following VEN application. Altogether, the data suggest that especially cell lines that expose a good initial VEN response are characterized by a high secondary VEN resistance.

We next established VEN resistance in orthotopic B-ALL xenograft models, focusing on *KMT2A*-r samples because those demonstrated the highest therapeutic efficacy in our recent studies^15^. We previously reported significant anti-proliferative effects after in vivo VEN exposure for three weeks (five doses per week)^15^. To induce VEN resistance in vivo, we again used this orthotopic SEM- and RS4;11-derived xenograft model but decreased the application interval to three doses per week. Instead of a three-week treatment period, mice were now continuously treated. Despite the increased application interval, the continuous VEN application resulted in reduced tumor cell proliferation compared to the previous group (Fig. S1A–D). At the same time, continuously treated mice lost less weight and had a superior overall performance status (Fig. S1E).

**Fig. 2:**
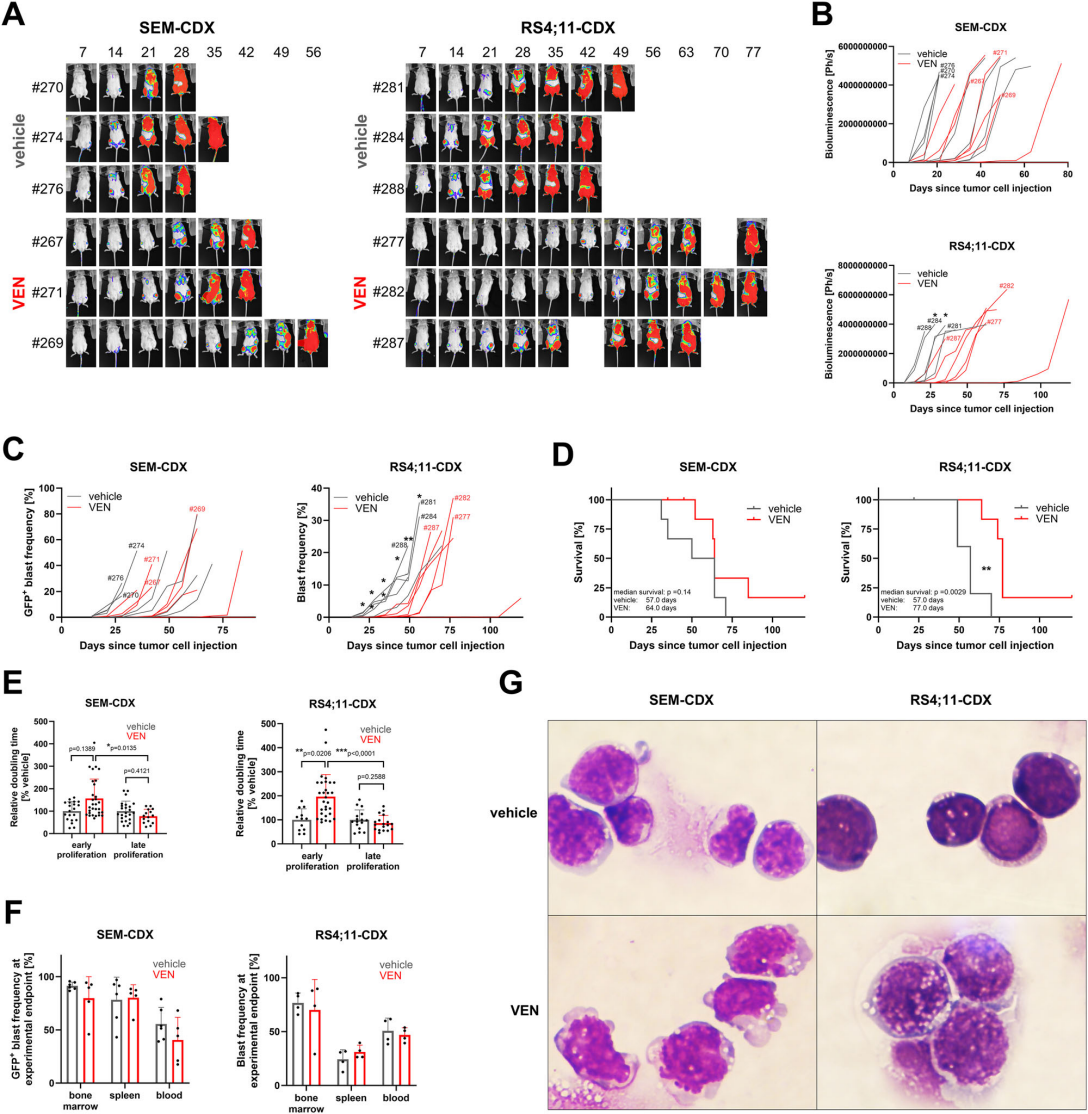
Induction of VEN resistance in SEM and RS4;11 cell line-derived B-ALL xenograft models. **A** Tumor cell proliferation was monitored by longitudinal full body bioluminescence imaging. Representative images of three animals per group. Mouse IDs are given for each animal to allow for allocation of individual animals in (**B**, **C**). **B** Bioluminescence signals from dorsal and ventral images were quantified. 5–8 animals per group, each line represents an individual animal. Mice displayed in (**A**) are marked with the respective mouse ID. Imaging was discontinued once technical signal saturation was achieved. Kolmogorov–Smirnov test for each time point. *p < 0.05. **C** Quantification of GFP^+^ tumor cell frequency in peripheral blood using flow cytometry. 5–8 animals per group, each line represents an individual animal. Mice displayed in (**A**) are marked with the respective mouse ID. Kolmogorov–Smirnov test for each time point. *p < 0.05; **p < 0.01. **D** Kaplan–Meier survival analysis. 6–8 animals per group, Log-rank test. **p < 0.01. **E** Relative tumor cell doubling times were calculated based on bioluminescence data (**B**; early proliferation) and peripheral blood flow cytometry values (**C**; late proliferation) and compared to time-matched vehicle cohorts. Mean ± SD of 5–8 animals per group, multiple data sets per animal during the exponential growth phase. Kolmogorov–Smirnov test. *p < 0.05; **p < 0.01; ***p < 0.001. **F** Determination of blast frequency in blood, bone marrow and spleen by flow cytometry when the mice reached humane endpoints (30% blasts in blood or weak performance status). Mean ± SD of 4-6 animals per group. Welch’s t test. **G** Isolated VEN-resistant bone marrow cells were spun onto microscopic slides and Pappenheim stained. Representative images of 5–8 mice per group, ×100 magnification.

Compared to controls, especially continuously VEN-treated RS4;11-engrafted mice demonstrated significantly reduced tumor cell proliferation while effects in SEM mice were less prominent (Fig. 2A–C). Due to therapy, the median survival was prolonged by twenty and seven days in RS4;11- and SEM-derived animals, respectively (Fig. 2D). In line, the doubling time of leukemic blasts was significantly higher in VEN-treated animals during early stages of therapy, indicating initial therapeutic response in both models (Fig. 2E). After prolonged inhibitor application, the tumor cell proliferation in the treated animals sped up to match or even surpass the control group. This decrease of blast doubling time in the presence of continuous VEN treatment depicts the outgrowth of VEN-resistant clones. Following experiment termination when mice reached 30% blasts in peripheral blood or humane endpoints, the leukemic blast frequency was determined in bone marrow, spleen, and blood (Fig. 2F). VEN-treated and control animals had comparable tumor cell infiltration across all compartments, suggesting similar kinetic and proliferation characteristics between untreated and secondary resistant blasts. Tumor cells were isolated from bone marrow and morphologically assessed (Fig. 2G). Despite the establishment of VEN resistance, the persistent and thus VEN-resistant cells in VEN-treated animals exhibited severe morphological damage, including apoptotic bodies, disintegrated cell membrane, and heavy vacuolization. Further, cell sizes of RS4;11-derived blasts were increased.

**Fig. 3:**
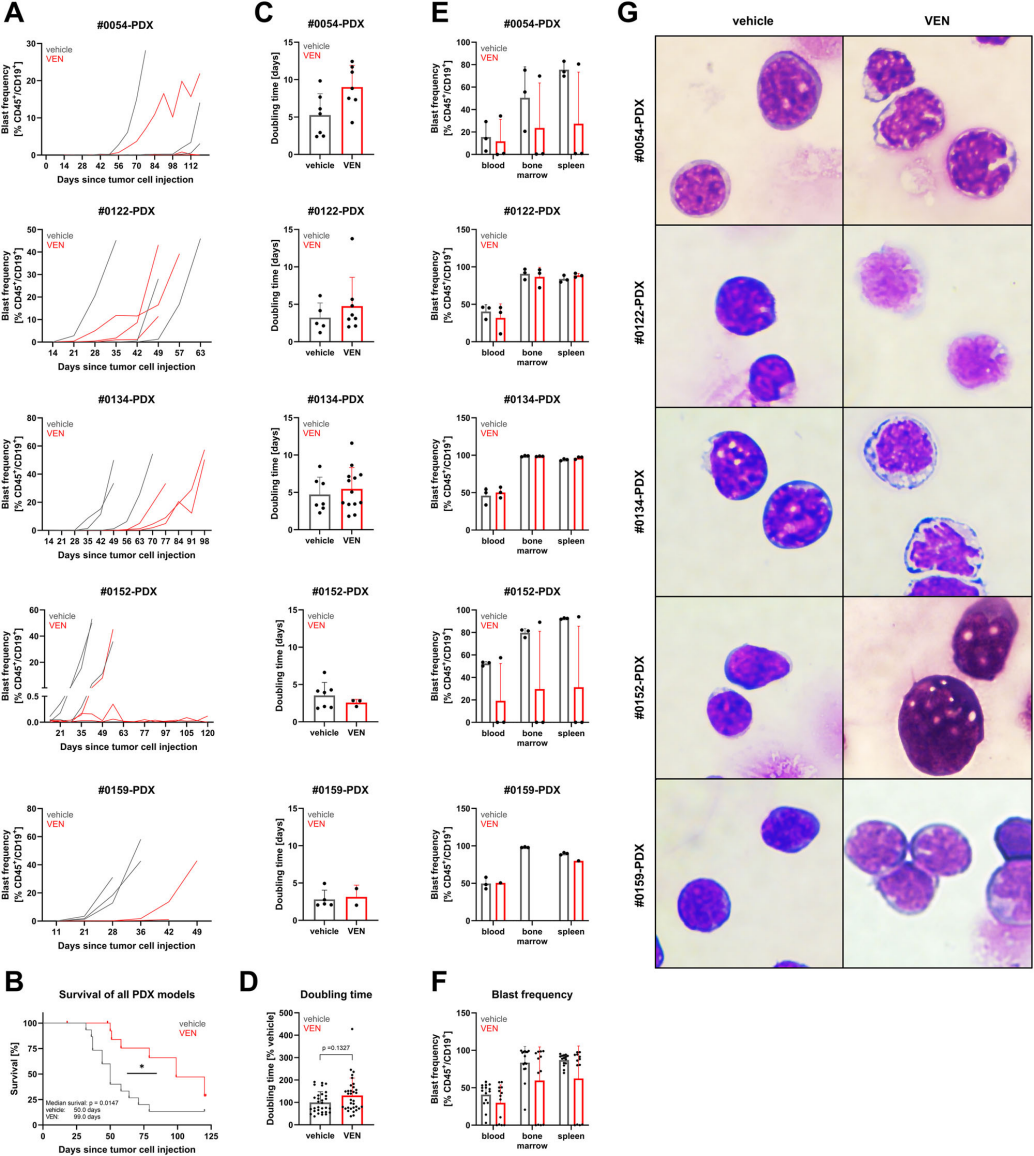
Induction of VEN resistance in PDX models. **A** Quantification of CD45^+^/CD19^+^ tumor cell frequency in peripheral blood using flow cytometry. 1–3 animals per group, each line represents an individual animal. **B** Kaplan–Meier survival analysis. All five PDX models were summarized. 15 animals per group, Log-rank test. *p < 0.05. **C** Tumor cell doubling times were calculated based on peripheral blood flow cytometry values (**A**). Mean ± SD of 1–3 animals per group, multiple data sets per animal during exponential growth phase. Unpaired *t* test. **D** Tumor cell doubling times summarized for all five PDX models. Mean ± SD of 1–3 animals per group, multiple data sets per animal during exponential growth phase. Kolmogorov–Smirnov test. **E** Determination of blast frequency in blood, bone marrow and spleen by flow cytometry when the mice reached humane endpoints (30% blasts in blood or weak performance status) or study endpoint (120 days post tumor cell injection). Mean ± SD of 1–3 animals per group. Kolmogorov–Smirnov test. **F** Tumor cell frequency in blood, bone marrow and spleen at experiment termination, summary of all five PDX models. Mean ± SD of 1–3 animals per group. No statistical assessment due to animals lacking VEN resistance. **G** Isolated VEN-resistant bone marrow cells were spun onto microscopic slides and Pappenheim stained. Representative images of 1–3 mice per group, 100x magnification. No statistic assessment of data from patients #0054 and #0152 derived models due to animals VEN-treated animals lacking resistance establishment.

Comparable results were achieved in five patient-derived xenograft models all harboring *KMT2A* rearrangements. Across all models, continuous treatment delayed blast proliferation and significantly prolonged survival (Fig. 3A, B). However, most mice and at least one animal per tumor model ultimately experienced VEN resistance and leukemia outgrowth. Tumor cell doubling times were increased during therapy in most settings, with limited explanatory power for models derived from patients #0152 and #0159 (Fig. 3C, D). Two out of three VEN-treated #0152-derived mice did not establish VEN resistance until the study endpoint 120 days after tumor cell injection. For #0159-derived animals, only one out of three VEN-treated mice could be evaluated. Similar to the cell line models, no difference between engraftment location was found (Fig. 3E, F) while morphology was severely impaired (Fig. 3G).

### VEN resistance is characterized by heterogeneous genetic, transcriptional and translational changes

We next set out to explore the underlying mechanisms of VEN resistance in B-ALL. Several pathways have been described in AML and CLL, mainly including mutations in *BCL2*^7^, *BAX*^8^, *TP53*^9,11^, and *FLT3*-ITD^9^ as well as changes in gene and protein expression of members of the BCL-2 signaling cascade or drug transporter MRP1 (coded by the *ABCC1* gene)^9,10,12^. Investigating parental and VEN-resistant cell lines as well as cell line- and patient-derived xenograft samples did not reveal deletions or insertions within the *FLT3*-ITD domain (Fig. S2). Gene expression of *ABCC1*, which encodes the transporter protein responsible for VEN efflux, was also not altered in VEN-resistant cell lines and cell line-derived xenografts compared to controls (Fig. S3). Three out of five PDX models presented with increased *ABCC1* transcriptional activity, while in the remaining two PDX models, *ABCC1* expression was decreased. Of note, intra-model heterogeneity was high for both control and VEN-treated animals, and only one to three animals were included in each group in the PDX models. These limitations contribute to a difficult overall assessment of the role of *ABCC1* expression in VEN resistance.

In contrast, several genetic variants were observed in the VEN-resistant cells, especially affecting the *TP53* gene (Table 1, Table S2). Importantly, the p.Phe134Leu and p.Arg337Cys variants found in one RS4;11-CDX and patient #0152-PDX mouse, respectively, are known to perturb the protein’s function via reduction of target promoter DNA binding affinity and thus loss of cell cycle arrest^18–20^. A well-described p.Pro72Arg variant that had constant allele frequencies in all three controls of PDX model #0134 was expanded in all corresponding VEN-resistant animals, suggesting clonal evolution towards an ALL-propagating environment^21^. Further, in vitro induction of VEN resistance resulted in two *BAX* mutations in RS4;11 cells and a *BAK1* frameshift insertion in the cell line REH. Overall, we observed no overlap between variants arising in resistant CDX mice and the corresponding in vitro cell line. These genetic analyses demonstrate high inter- and intra-model heterogeneity.

**Table 1:**
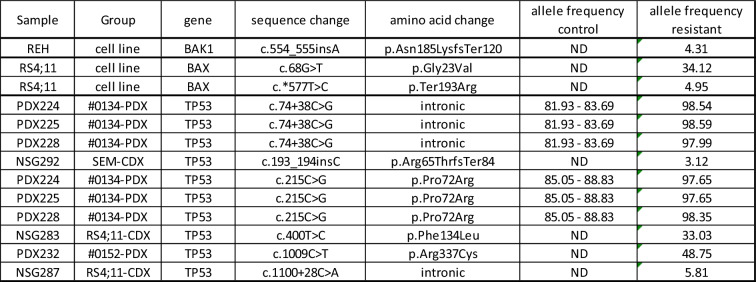
Variants detected in BAK1, BAX, BCL2 and TP53 across VEN-resistant cell lines, CDX and PDX samples

*ND* not detected.

To investigate if the detected genetic aberrations resulted in transcriptional changes, we further performed panel RNAseq of 214 genes related to apoptosis, leukemia and resistance-related pathways (Table S2). The *BAK1* and *BAX* variants detected in the VEN-resistant cell lines REH and RS4;11 did not result in relevant changes on the transcriptional level, but BAX protein expression was diminished in VEN-resistant and *BAX*-mutant RS4;11 cells (Fig. S4A, B). In line with reduced BAX protein expression, *BAX*-mutant cells were functionally impaired, as indicated by significantly lower capacity for apoptosis induction (Fig. S4C). In contrast, clonal proliferation of *TP53* variants in #0134-PDX models decreased *TP53* gene expression compared to controls. In SEM- and RS4;11-CDX animals, *TP53* expression was also reduced, but independently of arising mutations. Effects on TP53 downstream targets included altered *BBC3* and *BAX* expression, but also without a clear pattern (Fig. S4D).

**Fig. 4:**
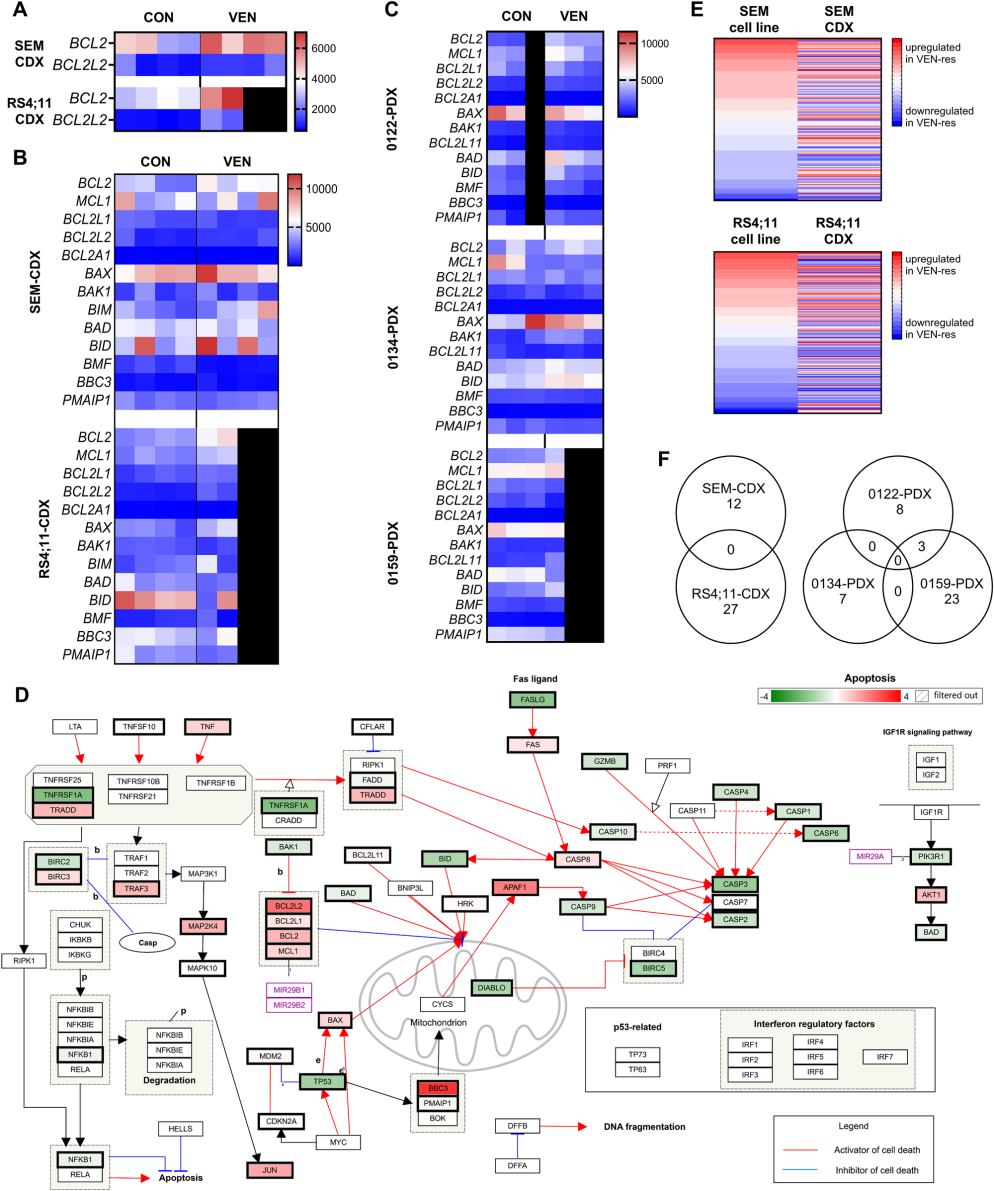
Gene expression profiles in CDX and PDX models. Each column represents an individual animal of the respective group (vehicle, left; VEN-resistant, right). Boxes are shaded black where fewer animals were analyzed in one subgroup. **A** BCL2 and BCL2L2 (BCL-w) expression patterns in CDX models. **B**, **C** Gene expression profiles of BCL-2 pathway genes in CDX (**B**) and PDX (**C**) mice. **D** Apoptosis pathway map including up- (red) and downregulation (green) of involved genes in VEN-resistant RS4;11-CDX animals compared to controls based on panel RNAseq data analyzed with the WikiPathways plugin in the Transcriptome Analysis Console software. **E** Heatmap demonstrating mean fold changes of 214 apoptosis-, leukemia- and resistance-related genes in VEN-resistant samples derived from SEM and RS4;11 cell line or CDX treatment. **F** Venn diagrams illustrating the number of overlapping significantly up- or down-regulated genes in CDX (left) and PDX (right) models.

Focusing on BCL-2 pathway signaling, we observed a high inter-model heterogeneity, while biological replicates in CDX and PDX models usually had similar gene expression patterns. We experienced upregulation of *BCL2* in both VEN-resistant CDX models, which was accompanied by increased anti-apoptotic *BCL2L2* (encoding the BCL-w protein) transcripts (Fig. 4A). SEM-CDX mice were further characterized by downregulation of apoptotic activator *BMF* while RS4;11-derived animals presented with higher *MCL1* and *BCL2L1* (BCL-xL) levels as well as significantly reduced *BIK* transcripts (Fig. 4B). Similarly, increased *BCL2* expression was observed in VEN-resistant PDX animals (Fig. 4C). Interestingly, and in contrast to expectations and CDX profiles, all PDX models also featured increased pro-apoptotic gene expression profiles to some extent, including *BAK1*, *BIM*, *BID* and *BAD*. This regulation, however, was no longer present on the protein level, suggesting post-transcriptional or post-translational modification or degradation to avoid apoptosis-inducing cellular programs as a mode of VEN resistance (Fig. S5).

Analyzing extended mechanisms of apoptosis, we further discovered overall reduced caspase and *TP53* expression accompanied by *JUN* upregulation in RS4;11-CDX mice (Fig. 4D). This supports the apoptosis-preventing transcriptional repression of pro-apoptotic genes as well as increased *BCL2*, *MCL1* and *BCL2L1* expression to induce VEN resistance. Similar modulations, including *FAS* and caspase downregulation and *MCL1* upregulation, were observed in SEM-CDX and PDX models (Table S3). In contrast, prolonged VEN incubation in the intrinsically resistant cell lines REH and NALM-6 did not change the overall gene expression profile of the cells towards an either more or less apoptosis-preventing pattern (Table S3). Comparison of VEN-induced effects in cell lines and the respective CDX models revealed major differences between both settings, suggesting a role for the leukemic niche and in vivo tumor microenvironment in resistance mechanisms (Fig. 4E). Also, the two CDX models as well as the three PDX models did not share any significantly up- or downregulated genes, underlining the intertumoral heterogeneity (Fig. 4F).

Further analyzing selected proteins within the BCL-2 pathway across VEN-resistant cell lines, CDX and PDX models, we once again discovered high inter- and intra-model heterogeneity (Fig. S6). Individual animals presented with strongly increased or decreased protein expression. Overall, and matching gene expression data, anti-apoptotic molecules like BCL-2, MCL-1, or BCL-xL tended to be rather upregulated, with the highest changes observed in PDX models. Alterations in pro-apoptotic proteins were less prominent, but more homogenous, as indicated by a significant reduction of BAK and BIM expression in SEM-CDX mice.

### Single-cell sequencing suggests a role for tumor microenvironment in resistance development

Of all five PDX models investigated, patient 0134 was the only sample with complete gene and protein expression profiles in three biological replicates as well as clear delay of tumor cell proliferation during VEN treatment (Fig. S7A). Due to the various modifications observed in #0134 PDX mice, including clonal expansion of a *TP53* subclone inducing reduced *TP53* gene expression (Fig. S7B, C) as well as upregulation of *BCL2* gene and protein expression (Fig. S7D, E), we investigated this model in more detail and performed single-cell transcriptomics of one control and two VEN-resistant animals.

**Fig. 5:**
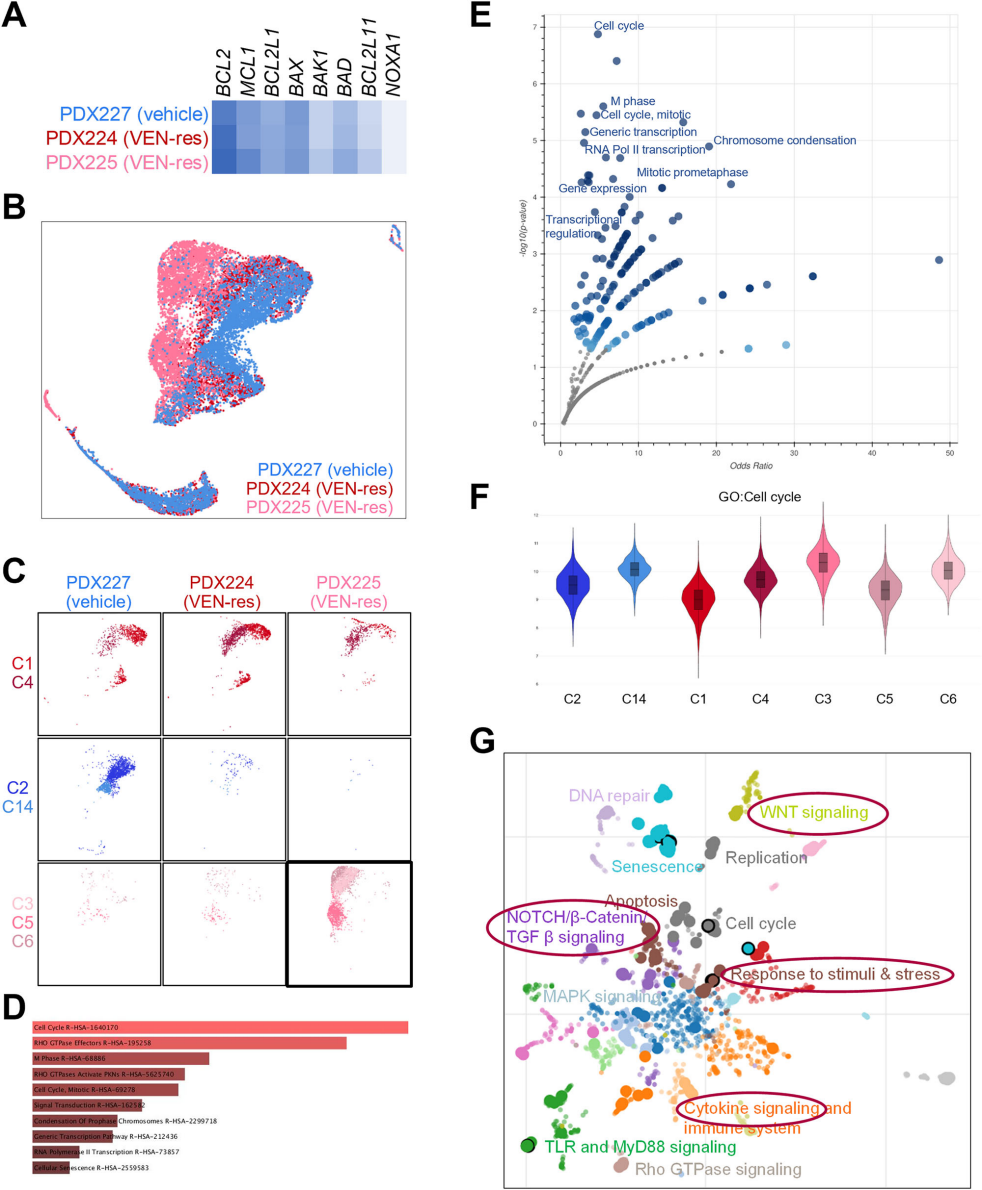
Gene expression profiling of three patient #0134-derived xenografts using single cell transcriptomics. PDX227 represents a vehicle-treated control animal while PDX224 and PDX225 continuously received VEN. **A** Bulk analysis of key BCL-2 pathway genes. **B** UMAP plot demonstrating the expression profiles of the control (blue) and the VEN-resistant animals (reds). **C** Distribution of specific clusters across the three samples. Clusters C3, C5, and C6 of sample PDX225 represent the further investigated resistance-specific signatures and are therefore highlighted. **D** Genes significantly overexpressed in PDX225-specific clusters C3, C5, and C6 were subjected to enrichment analysis and mapped to Reactome pathways to identify significantly regulated mechanisms. **E** Volcano plot visualizing the top regulated pathways in PDX225 (Clusters 3, 5, and 6). **F** Boxplots demonstrating the expression of genes allocated to the GO term Cell cycle throughout the sample-specific clusters. **G** UMAP plot visualizing Reactome pathways associated with significantly upregulated genes in PDX225-specific clusters 3, 5, and 6. Larger dots indicate a higher degree of regulation, and circled dots represent the top-influenced pathways.

Matching gene and protein expression data, bulk analysis of *BCL2* confirmed increased gene expression in both VEN-resistant samples compared to the control (Fig. 5A). Investigating the overall transcriptional profiles, we unexpectedly observed that one of the two VEN-resistant samples (PDX224) shared the majority of expression profiles with the control (PDX227) while the second resistant sample (PDX225) demonstrated a distinct pattern (Fig. 5B). Technical issues, variable sample quality and batch effect were excluded. Splitting all cells into 18 clusters revealed specific expression profiles that were exclusively observed within the control and lost in both VEN-resistant samples (clusters 2 and 14). We also determined clusters that were specific for the resistant sample PDX225 (clusters 3, 5, and 6). Clusters 1 and 4 were mainly attributed to VEN-resistant sample PDX224 but also present in the other animals (Fig. 5C, Fig. S8A).

Initially focusing on BCL2 family genes, we found that *BCL2* itself was overexpressed in clusters 3, 4, 5, and 6, which were all highly represented in VEN-resistant samples. PDX225-specific clusters 3, 5, and 6 further featured increased *MCL1* expression as well as downregulation of tumor-suppressive molecules *BAX* and *BAK1* compared to the control and the other resistant sample PDX224 (Fig. S8B).

Further analyzing the specific clusters in more detail identified genes and pathways that were differentially regulated in VEN-resistant and control cells. Clusters 3, 5, and 6, which were solely expressed in VEN-resistant sample PDX225, demonstrated up-regulation of genes that are involved in cell cycle regulation and transcription (Fig. 5D–G). Of note, only cluster 3 exhibited significantly upregulated genes in relation to all 18 clusters. These included cell cycle and proliferation promoters like *NCAPG*, *DYRK1A*, *CDCA5*, *KIF2C*, *SMC4*, *AURKB,* or *KIF18A*. Interestingly, some of these genes were also significantly downregulated in clusters 2 or 14, which are enriched in the vehicle sample PDX227. Moreover, these leukemic driver genes were also less expressed in clusters 5 and 6 of VEN-resistant sample PDX225, suggesting that cells allocated to cluster 3 exhibit a gene signature suggestive of highly proliferative, resistance-driving cells (Fig. S8C). Re-analysis of the cell cycle phases in patient #0134-derived xenografts did not reveal any significant differences between the three VEN-resistant animals or between resistant animals and controls (Fig. S8D), although the amount of cells in the G2/M phase was slightly lower in the VEN-treated mice.

Mapping significantly upregulated genes of the VEN-resistant clusters 3, 5, and 6 to the Reactome pathway database further revealed up-regulation of processes shaping the tumor microenvironment (TME) and response to cellular stimuli (Fig. 5G). In contrast, transcripts that were lost in resistant cells compared to the controls (clusters 2 and 14) included mainly factors related to cell-cell contacts (*PRSS12, MCTP1*), adhesion (*PTPN14, THSD7A, TRIO*) and membrane and cytoskeletal remodeling (*SNTB1, PLD1, ASPH, FMNL2*). Also, some tumor suppressor genes like *PTPN14*, *ZFHX3* or *LST1* were significantly down-regulated in VEN-resistant samples (Tables S4, S5).

To further investigate the role of the TME in more detail, we analyzed the protein expression of selected cell surface molecules involved in adhesion and cell-cell interaction in five different CDX and PDX models (Fig. S8E, F) following VEN treatment for five weeks. The overall abundance of the molecules was heterogeneous and treatment with VEN resulted in significant changes in protein expression. Interestingly, different proteins were regulated in the respective models, with CD9 (MRP-1), NOTCH1, CD148 (DEP1), CD26 (DPP4) and CD54 (ICAM-1) all being influenced in at least one system. Protein expression was usually upregulated upon VEN treatment but exceptions were observed, for example a significant down-regulation of CD9 in VEN-treated SEM-CDX mice. Applying a simple in vitro coculture model and incubating SEM and RS4;11 cells with VEN in the presence or absence of human stromal bone marrow cells revealed no influence of the feeder layer on blast proliferation and survival (Fig. S9). However, the lack of protecting effects was probably due to the limited three-dimensional architecture as well as insufficient cellular complexity of the model, which does not sufficiently recapitulate the bone marrow niche. Altogether, these data suggest a role for the TME in the development of VEN resistance that is worth further investigation.

## Discussion

VEN is successfully clinically used for several hematological malignancies, including AML and CLL^5,6^. However, high initial response rates are hampered by the development of secondary VEN resistance, which is a major problem resulting in leukemia relapse or progression. The mechanisms underlying resistance vary between CLL and AML, consequently necessitating different combination strategies^7–12^. For B-ALL, some early clinical trials promise high efficacy as well^13,14^, and preclinical analyses suggest high response rates also in high-risk cytogenetic subgroups like KMT2A-rearranged samples^15–17,22,23^. Still, seven out of nine ALL patients in a phase 1/2 study who initially showed complete remission finally relapsed under VEN and chemotherapy^14^. The molecular mechanism behind the resistance, however, is uninvestigated, prompting us to characterize VEN resistance in ALL.

As expected, our in vitro and in vivo models experienced secondary VEN resistance following initial response. On the genetic level, we detected several *TP53* variants that are known to perturb the TSG’s function^18–21^. In line, single-cell RNA sequencing of two VEN-resistant patient #0134-derived mice revealed upregulation of genes regulating the cell cycle in a *TP53*-related manner (*CENPE*^24^, *TOP2A*^25^, *NCAPG*^26^, *KIF2C*^27^, *AURKB*^28^, *CENPF*^29^, *TPX2*^30^) in clusters exclusively expressed in VEN-resistant cells. A subset (*NCAPG*, *KIF2C*, *AURKB*) of those genes was also significantly downregulated in control-specific clusters compared to cells that exhibited expression patterns found across all samples. Further, oncogenes and proliferation-promoting players known to facilitate leukemia like *DYRK1A*^31^, *CDCA5*^32^, or *SMC4*^33^ were also found overexpressed in resistance-specific clusters, suggesting a VEN-induced global initiation of growth-supporting cascades. Combinatory approaches with VEN and cell cycle modulators or inhibitors of DYRK1A, CDCA5, or SMC4 represent promising strategies for personalized ALL treatment and should be investigated in future studies. Due to the broad inter-model heterogeneity of VEN resistance observed in this study, it is important to individually identify patient-specific combination partners in order to achieve highly synergistic leukemia cell abrogation.

Further investigating BCL-2 pathway-related aberrations, we identified genetic variants in *BAX* and *BAK1* in our VEN-resistant cell lines, matching existing literature describing mutations arising in AML cells following VEN treatment^8^. In line, no BAX protein expression could be detected in the VEN-resistant RS4;11 cell line. Novel *BAX* and *BAK1* alterations result in alterations of the transmembrane domain. This suggests that continuous VEN application might force the leukemic cells to omit apoptosis by inhibition of BAX/BAK dimerization and integration in the outer mitochondrial membrane. This hypothesis is underlined by the fact that *BAX*-mutated RS4;11 cells were functionally impaired and required higher VEN concentrations to induce apoptosis compared to *BAX* wild-type cells.

Also matching experiences from AML treatment, following VEN treatment, we found upregulation of anti-apoptotic BCL-2 family members MCL1 and BCL-xL on transcriptional as well as protein level in some models^9,10^. Interestingly, all PDX systems with increased MCL-1 expression also had higher BCL-xL protein levels than the respective controls. To address this feedback mechanism, a clinical study combined VEN with Navitoclax to further target BCL-xL^13^. Another observed mechanism of VEN resistance was a significant downregulation of pro-apoptotic molecules BIM and BAK gene expression was mildly increased, suggesting once more that posttranslational modifications or protein degradation play a major role in BCL-2 pathway regulation. Overall correlation of gene and protein expression of most investigated BCL-2 pathway molecules was rather low, including several cases where biological replicates.

Taking together the data obtained from VEN-resistant cells on the genetic, transcriptional, and protein levels demonstrates a very high inter- and intra-model heterogeneity. This is also indicated by the fact that the VEN-resistant cell lines in vitro do not resemble patterns observed in the corresponding in vivo xenograft models, underlining the relevance and importance of suitable and realistic preclinical models. Previous studies have shown that orthotopic PDX do indeed recapitulate the genetic and transcriptional landscape of the primary tumor to a large extent, suggesting that the herein observed alterations following PDX VEN treatment might also occur in the clinical setting^34–37^.

Still, samples collected during clinical application using VEN in human ALL therapy would have increased the translational character of the study. Another limitation is the small sample size with only five PDX models investigated and a low number of biological replicates. This impedes a broad and holistic analysis of global mechanisms of VEN resistance and requests careful interpretation of the presented results. Together with two CDX models, our cohort comprises a total of 25 controls and 19 VEN-treated mice with a *KMT2A*-r karyotype; and even in this small group we observed several different and heterogeneous potential mechanisms of VEN resistance on the genetic, transcriptional and translational level, even within the same xenograft model. These results demonstrate, although with low numbers of biological replicates, the extensive biological heterogeneity of KMT2A-r ALL samples treated with VEN.

However, our cohort also featured some VEN-resistant mice that did not exhibit any obvious changes compared to the controls. This might be due to the fact that targeted mutational and expression analyses were restricted to only a small subset of genes and proteins, thus missing out on relevant alterations. For example, Enzenmüller et al. recently reported resistance-specific metabolic patterns in VEN-treated B-ALL models^38^. Another possible explanation is the involvement of the TME in resistance development. Single-cell RNAseq of patient #0134-derived xenograft samples hints that TME genes are upregulated in resistance-specific clusters, and proteins responsible for cell-cell interaction were modulated across all seven PDX and CDX models. Karjalainen et al. also found that in vitro co-culturing of AML blasts with stromal cells changes the cells’ dependency on BCL-2, suggesting a potential microenvironment-mediated way of BCL2 inhibitor resistance^39^. Chemokine axes and mitochondrial priming further contribute to BCL-2 family protein regulation and can thus contribute to resistance development and maintenance^40,41^. These mechanisms are also a possible explanation of the differences observed between in vitro and in vivo models in our study.

Altogether, our results demonstrate multiple possible mechanisms of VEN resistance arising in ALL xenograft models. VEN resistance appears to be highly heterogeneous between and within models. Finally, the role of the TME should be further investigated to analyze the BCL-2-dependent crosstalk between leukemic blasts and stromal cells within the leukemic niche.

## Methods

### Cell line cultivation and Venetoclax resistance induction

Human B—ALL cell lines SEM, RS4;11, REH, and NALM-6 were purchased from DSMZ (Braunschweig, Germany) and maintained at 37 °C and 5% CO_2_ in IMDM medium (SEM), Alpha MEM medium (RS4;11) or RPMI medium (REH, NALM-6) supplemented with 10% heat-inactivated fetal calf serum and 100 µg/ml penicillin/streptomycin (all PAN—biotech, Aidenbach, Germany). Medium was changed twice weekly and cells were seeded at a density of 3.3 ×105 cells per ml for further cultivation or inhibitor experiments. Cells were regularly checked for authenticity (cell surface flow cytometry) and mycoplasma contamination.

For resistance induction, Venetoclax (VEN) was purchased from MedChemExpress (Monmouth Junction, NJ, USA) and cell culture media were continuously supplemented with increasing VEN concentrations (1 nM to 2.5 µM over 15 weeks). Drug response was evaluated by WST-1 assay (Roche, Basel, Switzerland) and IC50 values were assessed via non-linear curve fit using GraphPad Prism 8.0.2.

### Coculturing and assessment of proliferation and viability

SEM or RS4;11 cells were cultivated alone or cocultured with human stromal bone marrow cells (HS-5 cell line, ATCC, Manassas, VI, USA) and incubated with DMSO (control) or VEN for 72 h. Absolute cell counts were assessed by trypan blue staining and subsequent microscopic quantification. Cells were then stained for CD19 and Annexin V to determine the ratio between leukemic blasts and stromal cells as well as the viability of both cell types.

### In vivo model systems

All animal experiments were approved by the review board of the federal state Mecklenburg-Vorpommern, Germany (reference number: LALLF MV/7221.3-1.1-063/20). Eight to twelve weeks old male and female NOD.Cg-*Prkdc*^*scid*^*Il2rg*^*tm1Wjl*^/Szj (NSG) mice were bred and housed in the accredited laboratory animal Core Facility of the Rostock University Medical Center with access to water and standard chow *ad libitum*. All experiments were carried out in a laboratory setting and no intervention was performed within the animal housing and breeding rooms. SEM and RS4;11 cells lentivirally transduced with GFP and luciferase (SEM–fluc, RS4;11–fluc)^42^, or primary adult ALL cells amplified in a patient-derived xenograft (PDX) model system were used for inhibitory experiments. Primary cells were isolated as previously described^43^. The study was performed in accordance to the Declaration of Helsinki and the local ethical standards of the Rostock University Medical Center. All participants gave informed consent. Detailed patient characteristics can be retrieved from Table S6. Tumor cell injection, monitoring of blast counts and distribution using in vivo bioluminescence imaging and peripheral blood flow cytometry (GFP^+^ or CD45^+^/CD19^+^) were performed as previously described^34,43–45^. Tumor cell doubling cell calculations were performed as explained before^15^.

### In vivo treatment procedures and study endpoints

For cell line-derived xenograft model systems, five to eight animals each were continuously treated with either VEN or vehicle on three days per week starting seven days after tumor cell injection (i.v., 2.5 × 10^6^ cells per animal). Group sizes were calculated in G*power based on expected effect sizes, a statistical power of 0.8 and alpha error of 0.05. Only mice with a bioluminescence signal indicating successful tumor cell engraftment were included in experiments. Randomization was performed based on sex, age, weight and bioluminescence signal at day 7 after cell injection. Study groups were not blinded to the investigators. VEN was dissolved in 60% Phosal 50 PG (Lipoid, Ludwigshafen, Germany), 30% PEG400 (Carl Roth, Karlsruhe, Germany) and 10% ethanol and applied via oral gavage. During the first week of treatment, VEN concentrations were increased according to the clinically applied protocol, starting at 20 mg/kg body weight, and raised to the maximum dose of 100 mg/kg body weight at day 11. For PDX models, six mice per PDX model were injected with cells derived from one patient. Three mice each were treated with either VEN or vehicle as described above. Mice were monitored and weighed daily and bioluminescence imaging as well as peripheral blood blast frequency measurement was performed once per week as previously described^43–45^. Mice were euthanized by narcotization (75 mg/kg ketamine, 5 mg/kg xylazine) followed by cervical dislocation when blast frequencies reached ≥ 30% in blood or when mice met pre-defined humane endpoints.

For the analysis of cell surface markers CD148, CD54, CD9, NOTCH1, and CD26, mice were treated three times a week for five weeks, and spleen cells were isolated when the above-mentioned criteria were reached.

### FLT3-ITD analysis

DNA was isolated using the AllPrep DNA/RNA Mini Kit (Qiagen, Hilden, Germany) according to the manufacturer’s guidelines. An endpoint PCR using the primers F: GCACATTCCATTCTTACCAAACTC and R: TATCTGCAGAACTGCCTATTCCTA and subsequent agarose gel electrophoresis was used to investigate insertions within the *FLT3*-ITD region. The mastermix was prepared using 10x DreamTaq buffer (Thermo Fisher Scientific, Waltham, MA, USA), 0.0625 U DreamTaq (Thermo Fisher Scientific), 0.2 mM of each dNTP (Hersteller), 0.5 µM of each primer (Eurofins, Ebersberg, Germany) and 100 ng of DNA in a total volume of 50 µl. The PCR protocol commenced with an initial denaturation of 4 min at 95 °C followed by thirty cycles of 95 °C for 30 sec, 56 °C for 45 sec and 72 °C for 30 sec and terminated by a final elongation step of 72 °C for 5 min. PCR products were loaded on a 3.5% agarose/TAE buffer (Biozym, Hessisch Oldendorf, Germany) gel and stained with Roti-Safe Gel Stain (Carl Roth, Karlsruhe, Germany). Electrophoresis was carried out at 160 V for 1 h.

### ABCC gene expression analysis

RNA isolation was carried out using the AllPrep DNA/RNA Mini Kit (Qiagen) and followed by cDNA synthesis facilitating High-Capacity cDNA Reverse Transcription Kit (Thermo Fisher Scientific) according to the manufacturer’s guidelines. Gene expression was measured in technical triplicates in a ViiA7 Real Time PCR system (Applied Biosystems, Foster City, USA) using the TB Green™ Premix Ex Taq™ II mastermix (Takara Bio Europe, Saint-Germain-en-Laye, France), 0.4 µM primers (*ABCC1*-F: TTACTCATTCAGCTCGTCTTGTC; *ABCC1*-R: CAGGGATTAGGGTCGTGGAT; *GAPDH*-F: CTGCACCACCAACTGCTTAG; *GAPDH*-R: GTCTTCTGGGTGGCAGTGAT) and 20 ng cDNA in a final volume of 20 µl. The PCR consisted of 30 sec initial denaturation at 95 °C followed by 40 cycles of 5 s denaturation at 95 °C and 30 s annealing/elongation at 60 °C. *ABCC1* gene expression was normalized to sample-matching *GAPDH* housekeeping gene values.

### Targeted DNA panel sequencing

Genomic DNA (gDNA) was isolated using the AllPrep DNA/RNA Mini Kit (Qiagen) according to the manufacturer’s guidelines. Full gene sequencing of BCL2, BAX, BAK1 and TP53 was performed by sequencing with an AmpliSeq Custom DNA panel (Thermo Fisher Scientific). Per primer pool, 10 ng of gDNA were amplified in a 21 cycle multiplex PCR. Library quantification was performed via a Taqman-based real-time PCR using the Ion Library TaqMan™ Quantitation Kit (Thermo Fisher Scientific) according to the manufacturer’s instructions. Templating and chip loading were accomplished using an Ion Chef™ instrument (Thermo Fisher Scientific). Sequencing runs were conducted on an Ion GeneStudio S5 Plus system (Thermo Fisher Scientific). For data analysis, the human genome assembly hg19 was used as the alignment reference sequence. Amplicon coverage and variant calling were analyzed using the Torrent Suite™ software (v5.18.1) and the corresponding allele frequencies were determined using the Ion Reporter™ Software (v5.20.8.0) (both Thermo Fisher Scientific). Relevant variants were further investigated and screened for listing in dbSNP and ClinVar databases.

### Targeted RNA panel sequencing

RNA isolation was carried out using the AllPrep DNA/RNA Mini Kit (Qiagen) according to the manufacturer’s guidelines. RNA panel sequencing was performed as previously described^15^. Genes relevant for either apoptotic processes or involved in leukemic signaling were included in the panel. A list of the 214 genes investigated can be retrieved from Table S1.

### Immunoblotting

Protein lysates were prepared using Ripa buffer including protease and phosphatase inhibitors (both Cell Signaling Technology, Danvers, MA, USA) and quantified by Bradford assay. Proteins were separated, blotted, imaged and quantified as previously described^44^. All antibodies and dilutions are listed in Table S7. Uncropped immunoblots are available in the supplemental material section.

### Single-cell transcriptomics

Tumor cells isolated from the spleens of PDX animals were washed and diluted to 1 × 10^6^ cells per ml in PBS/0.04% BSA. The Single Cell 3´ Gene Expression libraries were constructed using the Chromium Next GEM single Cell 3’ Reagent Kits v3.1, dual index (10x Genomics, Pleasanton, CA, USA) according to the manufacturer’s guidelines. Briefly, approximately 10,000 cells were loaded onto a chip to generate Gel Beads-in-emulsion (GEMs) reactions using the Chromium Single Cell Controller version 4.0. The cDNA samples and final libraries were quality-checked for fragment size distribution on Agilent Bioanalyzer High Sensitivity Chip. The libraries were normalized to a final concentration of 650 pM and paired-end sequenced (Read1 for 28 cycles, i7-Index for 10 cycles, i5-Index for 10 cycles and Read2 for 90 cycles) on the NextSeq 2000 system (Illumina) at the NGS facility of the Research Institute for Farm Animal Biology (FBN), Germany.

Raw reads (fastq) were demultiplexed and generated using dragen bcl convert v3.10.11. The data were processed using nf-core/scrnaseq pipeline^46^. Cell Ranger v7.1.0 was used for alignment of the data to the GRCh38 human reference genome (Ensembl, version 109) and generation of count matrix. QC matrices and cell selection and filtration were carried out using Seurat^47^. Data were further processed using Azimuth and the Loupe Browser software (version 8.1, 10X Genomics). Enrichment analyses were conducted using the Enrichr pipeline^48–50^. Raw data can be retrieved from the European Bioinformatics Institute’s (EBI) platform BioStudies (accession number E-MTAB-14805).

### Flow cytometric TME marker profiling

For quantification of CD148, CD54, CD9, NOTCH1, and CD26, mice were euthanized and spleen cells were isolated as previously described^34^. Cells were harvested and washed in cold PBS, fixed in methanol-free 4% formaldehyde for 15 min and washed twice in PBS before permeabilization in ice-cold 90% methanol for 30 min. After two steps of PBS washing, cells were blocked in antibody dilution buffer for 10 min and incubated with the antibodies listed in Table S8 for 35 min at room temperature. Cells were again washed twice in antibody dilution buffer and analyzed using the FACSLyric™ device (BD, Heidelberg, Germany) with FACSuite™ software (BD; version 1.0.6.5230).

### Statistical analyses

All values are expressed as mean ± standard deviation (SD). Gaussian normal distribution was tested in all cases, determining the following parametric or non-parametric with post hoc test. The exact test is indicated in the respective figure legends. Kaplan–Meier curves and respective statistics were applied to estimate survival benefits. Statistical analyses were performed using GraphPad PRISM software (version 8). Statistical significance was defined as *p < 0.05, **p < 0.01 and ***p < 0.001.

## Supplementary information

Supplemental material_Fig_Tab_blots

## Data Availability

All raw data are available from the corresponding author upon reasonable request. Single-cell sequencing raw data files can be retrieved from the European Bioinformatics Institute’s (EBI) platform BioStudies (accession number E-MTAB-14805).

## References

[1] Topp, M. S. et al. Phase II trial of the anti-CD19 bispecific T cell-engager blinatumomab shows hematologic and molecular remissions in patients with relapsed or refractory B-precursor acute lymphoblastic leukemia. *J. Clin. Oncol.* **32**, 4134–4140 (2014). 25385737 10.1200/JCO.2014.56.3247

[2] Maude, S. L. et al. Tisagenlecleucel in Children and Young Adults with B-Cell Lymphoblastic Leukemia. *N. Engl. J. Med.* **378**, 439–448 (2018). 29385370 10.1056/NEJMoa1709866 PMC5996391

[3] Ottmann, O. G. et al. A phase 2 study of imatinib in patients with relapsed or refractory Philadelphia chromosome-positive acute lymphoid leukemias. *Blood* **100**, 1965–1971 (2002). 12200353 10.1182/blood-2001-12-0181

[4] Valentin, R., Grabow, S. & Davids, M. S. The rise of apoptosis: targeting apoptosis in hematologic malignancies. *Blood* **132**, 1248–1264 (2018). 30012635 10.1182/blood-2018-02-791350

[5] DiNardo, C. D. et al. Azacitidine and venetoclax in previously untreated acute myeloid leukemia. *N. Engl. J. Med.* **383**, 617–629 (2020). 32786187 10.1056/NEJMoa2012971

[6] Roberts, A. W. et al. Targeting BCL2 with venetoclax in relapsed chronic lymphocytic leukemia. *N. Engl. J. Med.* **374**, 311–322 (2016). 26639348 10.1056/NEJMoa1513257 PMC7107002

[7] Blombery, P. et al. Multiple BCL2 mutations cooccurring with Gly101Val emerge in chronic lymphocytic leukemia progression on venetoclax. *Blood* **135**, 773–777 (2020). 31951646 10.1182/blood.2019004205 PMC7146015

[8] Moujalled, D. M. et al. Acquired mutations in BAX confer resistance to BH3-mimetic therapy in acute myeloid leukemia. *Blood* **141**, 634–644 (2023). 36219880 10.1182/blood.2022016090 PMC10651776

[9] DiNardo, C. D. et al. Molecular patterns of response and treatment failure after frontline venetoclax combinations in older patients with AML. *Blood* **135**, 791–803 (2020). 31932844 10.1182/blood.2019003988 PMC7068032

[10] Chyla, B. et al. Genetic biomarkers of sensitivity and resistance to venetoclax monotherapy in patients with relapsed acute myeloid leukemia. *Am. J. Hematol.* **93**, E202–E205 (2018). 29770480 10.1002/ajh.25146 PMC6120451

[11] Nechiporuk, T. et al. The TP53 apoptotic network is a primary mediator of resistance to BCL2 inhibition in AML cells. *Cancer Discov.* **9**, 910–925 (2019). 31048320 10.1158/2159-8290.CD-19-0125 PMC6606338

[12] Ebner, J. et al. ABCC1 and glutathione metabolism limit the efficacy of BCL-2 inhibitors in acute myeloid leukemia. *Nat. Commun.* **14**, 5709 (2023). 37726279 10.1038/s41467-023-41229-2 PMC10509209

[13] Pullarkat, V. A. et al. Venetoclax and navitoclax in combination with chemotherapy in patients with relapsed or refractory acute lymphoblastic leukemia and lymphoblastic lymphoma. *Cancer Discov.* **11**, 1440–1453 (2021). 33593877 10.1158/2159-8290.CD-20-1465 PMC9533326

[14] Short, N. J. et al. A phase 1/2 study of mini-hyper-CVD plus venetoclax in patients with relapsed/refractory acute lymphoblastic leukemia. *Blood Adv.* **8**, 909–915 (2024). 38207208 10.1182/bloodadvances.2023012231 PMC10875259

[15] Richter, A. et al. Effective tumor cell abrogation via Venetoclax-mediated BCL-2 inhibition in KMT2A-rearranged acute B-lymphoblastic leukemia. *Cell Death Discov*. **8** (2022). 10.1038/s41420-022-01093-3 PMC9249764 35778418

[16] Seyfried, F. et al. Prediction of venetoclax activity in precursor B-ALL by functional assessment of apoptosis signaling. *Cell Death Dis*. **10** (2019). 10.1038/s41419-019-1801-0 PMC6662703 31358732

[17] Benito, J. M. et al. MLL-rearranged acute lymphoblastic leukemias activate BCL-2 through H3K79 methylation and are sensitive to the BCL-2-specific antagonist ABT-199. *Cell Rep.* **13**, 2715–2727 (2015). 26711339 10.1016/j.celrep.2015.12.003 PMC4700051

[18] Bullock, A. N., Henckel, J. & Fersht, A. R. Quantitative analysis of residual folding and DNA binding in mutant p53 core domain: definition of mutant states for rescue in cancer therapy. *Oncogene* **19**, 1245–1256 (2000). 10713666 10.1038/sj.onc.1203434

[19] Jansson, M. et al. Arginine methylation regulates the p53 response. *Nat. Cell Biol.* **10**, 1431–1439 (2008). 19011621 10.1038/ncb1802

[20] Kato, S. et al. Understanding the function-structure and function-mutation relationships of p53 tumor suppressor protein by high-resolution missense mutation analysis. *Proc. Natl. Acad. Sci. USA.* **100**, 8424–8429 (2003). 12826609 10.1073/pnas.1431692100 PMC166245

[21] Tian, X., Dai, S., Sun, J., Jiang, S. & Jiang, Y. Association between TP53 Arg72Pro polymorphism and leukemia risk: a meta-analysis of 14 case-control studies. *Sci. Rep.* **6**, 24097 (2016). 27053289 10.1038/srep24097 PMC4823650

[22] Cheung, L. C. et al. Preclinical efficacy of azacitidine and venetoclax for infant KMT2A-rearranged acute lymphoblastic leukemia reveals a new therapeutic strategy. *Leukemia*10.1038/s41375-022-01746-3 (2022). 10.1038/s41375-022-01746-3 PMC9883157 36380143

[23] Khaw, S. L. et al. Venetoclax responses of pediatric ALL xenografts reveal sensitivity of MLL-rearranged leukemia. *Blood* **128**, 1382–1395 (2016). 27343252 10.1182/blood-2016-03-707414 PMC5016707

[24] Liu, S. et al. RUNX1 Upregulates CENPE to promote leukemic cell proliferation. *Front. Mol. Biosci.* **8**, 692880 (2021). 34434964 10.3389/fmolb.2021.692880 PMC8381024

[25] Chen, T., Sun, Y., Ji, P., Kopetz, S. & Zhang, W. Topoisomerase IIα in chromosome instability and personalized cancer therapy. *Oncogene* **34**, 4019–4031 (2015). 25328138 10.1038/onc.2014.332 PMC4404185

[26] Jia, Y. et al. Identification of NCAPG as an essential gene for neuroblastoma employing CRISPR-Cas9 Screening Database and experimental verification. *Int. J. Mol. Sci*. **24** (2023). 10.3390/ijms241914946 PMC10573393 37834394

[27] Gwon, M.-R., Cho, J. H. & Kim, J.-R. Mitotic centromere-associated kinase (MCAK/Kif2C) regulates cellular senescence in human primary cells through a p53-dependent pathway. *FEBS Lett.* **586**, 4148–4156 (2012). 23098759 10.1016/j.febslet.2012.10.012

[28] Qi, J. et al. Selective inhibition of Aurora A and B kinases effectively induces cell cycle arrest in t(8;21) acute myeloid leukemia. *Biomed. Pharmacother.* **117**, 109113 (2019). 31207577 10.1016/j.biopha.2019.109113

[29] Shi, M. et al. Upregulated mitosis-associated genes CENPE, CENPF, and DLGAP5 predict poor prognosis and chemotherapy resistance of Acute Myeloid Leukemia. *Cancer biomarkers* **35**, 11–25 (2022). 35634845 10.3233/CBM-203170 PMC12364220

[30] Byrum, A. K. et al. Mitotic regulators TPX2 and Aurora A protect DNA forks during replication stress by counteracting 53BP1 function. *J. Cell Biol.* **218**, 422–432 (2019). 30602538 10.1083/jcb.201803003 PMC6363440

[31] Li, Y. et al. DYRK1a mediates BAFF-induced noncanonical NF-κB activation to promote autoimmunity and B-cell leukemogenesis. *Blood* **138**, 2360–2371 (2021). 34255829 10.1182/blood.2021011247 PMC8832461

[32] Grothusen, G. P. et al. DCAF15 control of cohesin dynamics sustains acute myeloid leukemia. *Nat. Commun.* **15**, 5604 (2024). 38961054 10.1038/s41467-024-49882-x PMC11222469

[33] Peng, L. et al. Structural maintenance of chromosomes 4 is required for leukemia stem cell maintenance in MLL-AF9 induced acute myeloid leukemia. *Leuk. Lymphoma* **59**, 2423–2430 (2018). 29043883 10.1080/10428194.2017.1387906

[34] Richter, A. et al. The molecular subtype of adult acute lymphoblastic leukemia samples determines the engraftment site and proliferation kinetics in patient-derived xenograft models. *Cells* **11**, 1–22 (2022). 10.3390/cells11010150 PMC8750004 35011712

[35] Richter-Pechańska, P. et al. PDX models recapitulate the genetic and epigenetic landscape of pediatric T-cell leukemia. *EMBO Mol. Med.* **10**, 1–13 (2018). 30389682 10.15252/emmm.201809443 PMC6284381

[36] Uzozie, A. C. et al. PDX models reflect the proteome landscape of pediatric acute lymphoblastic leukemia but divert in select pathways. *J. Exp. Clin. Cancer Res.* **40**, 1–24 (2021). 33722259 10.1186/s13046-021-01835-8 PMC7958471

[37] Woiterski, J. et al. Engraftment of low numbers of pediatric acute lymphoid and myeloid leukemias into NOD/SCID/IL2Rcγnull mice reflects individual leukemogenecity and highly correlates with clinical outcome. *Int. J. Cancer* **133**, 1547–1556 (2013). 23526331 10.1002/ijc.28170

[38] Enzenmüller, S. et al. Venetoclax resistance in acute lymphoblastic leukemia is characterized by increased mitochondrial activity and can be overcome by co-targeting oxidative phosphorylation. *Cell Death Dis.* **15**, 475 (2024). 38961053 10.1038/s41419-024-06864-7 PMC11222427

[39] Karjalainen, R. et al. JAK1/2 and BCL2 inhibitors synergize to counteract bone marrow stromal cell-induced protection of AML. *Blood* **130**, 789–802 (2017). 28619982 10.1182/blood-2016-02-699363

[40] Gómez, A. M. et al. Chemokines and relapses in childhood acute lymphoblastic leukemia: a role in migration and in resistance to antileukemic drugs. *Blood Cells. Mol. Dis.* **55**, 220–227 (2015). 26227851 10.1016/j.bcmd.2015.07.001

[41] Chiron, D. et al. Rational targeted therapies to overcome microenvironment-dependent expansion of mantle cell lymphoma. *Blood* **128**, 2808–2818 (2016). 27697772 10.1182/blood-2016-06-720490

[42] Terziyska, N. et al. In vivo imaging enables high resolution preclinical trials on patients’ leukemia cells growing in mice. *PLoS ONE* **7**, e52798 (2012). 23300782 10.1371/journal.pone.0052798 PMC3534096

[43] Roolf, C. et al. Decitabine demonstrates antileukemic activity in B cell precursor acute lymphoblastic leukemia with MLL rearrangements. *J. Hematol. Oncol.* **11**, 62 (2018). 29728108 10.1186/s13045-018-0607-3 PMC5936021

[44] Richter, A. et al. Combined Casein Kinase II inhibition and epigenetic modulation in acute B-lymphoblastic leukemia. *BMC Cancer* **19**, 202 (2019). 30841886 10.1186/s12885-019-5411-0 PMC6404304

[45] Richter, A. et al. Influence of Casein kinase II inhibitor CX-4945 on BCL6-mediated apoptotic signaling in B-ALL in vitro and in vivo. *BMC Cancer* **20**, 184 (2020). 32131762 10.1186/s12885-020-6650-9 PMC7057698

[46] Marques de Almeida, F. et al. nf-core/scrnaseq: 4.0.0. at (2025).

[47] Hao, Y. et al. Dictionary learning for integrative, multimodal and scalable single-cell analysis. *Nat. Biotechnol.* **42**, 293–304 (2024). 37231261 10.1038/s41587-023-01767-y PMC10928517

[48] Chen, E. Y. et al. Enrichr: interactive and collaborative HTML5 gene list enrichment analysis tool. *BMC Bioinformatics* **14**, 128 (2013). 23586463 10.1186/1471-2105-14-128 PMC3637064

[49] Kuleshov, M. V. et al. Enrichr: a comprehensive gene set enrichment analysis web server 2016 update. *Nucleic Acids Res.* **44**, W90–W97 (2016). 27141961 10.1093/nar/gkw377 PMC4987924

[50] Xie, Z. et al. Gene set knowledge discovery with Enrichr. *Curr. Protoc.* **1**, e90 (2021). 33780170 10.1002/cpz1.90 PMC8152575

