# Supplementary material for: Venetoclax resistance in preclinical KMT2A-rearranged acute lymphoblastic leukemia models is characterized by high inter- and intra-model heterogeneity
Source: NPJ Precis Oncol. 2025 Dec 29;10:47. doi: 10.1038/s41698-025-01249-1 (PMC12855817; doi:10.1038/s41698-025-01249-1)
Supplement: Supplementary file 1 — Supplemental material_Fig_Tab_blots [file 41698_2025_1249_MOESM1_ESM.pdf]

Venetoclax resistance in preclinical KMT2A-rearranged acute lymphoblastic leukemia models is characterized by high inter- and intra-model heterogeneity

Anna Richter [1,*], Lea Kinsky [1], Sandra Lange [1], Nares Trakooljul [2], Frieder Hadlich [2], Anett Sekora [1], Gudrun Knuebel [1,3], Saskia Krohn [1,3], Christian Schmidt [1], Michelle Busch [1], Tim Schreiber [4], Simone Kumstel [4], Klaus Wimmers [2], Hugo Murua Escobar [1,3], Christian Junghanss [1]

Supplemental Figures

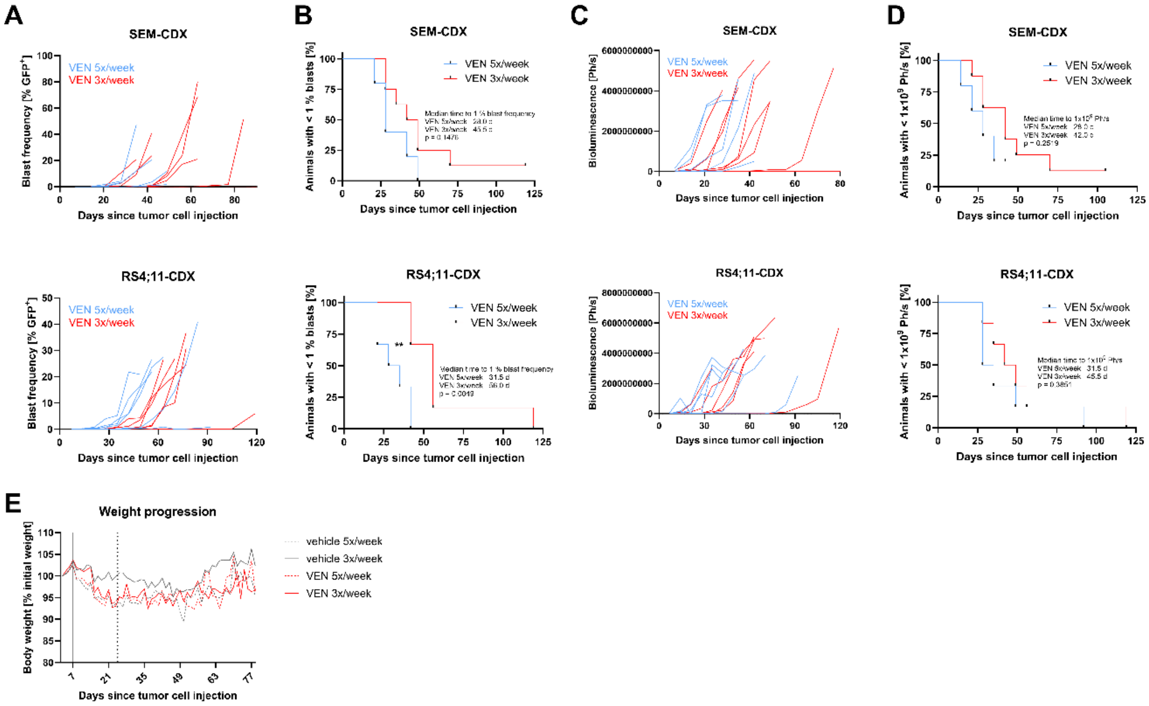

Figure S1: Comparison of previously published [15] fixed term (5x/week, 3 weeks) and the novel continuous (3x/week) VEN treatment. A Blast frequencies of SEM and RS4;11 xenograft mice were assessed using flow cytometry. 5-8 animals per group. **B** Kaplan Meier curve showing the fraction of animals reaching 1 % blasts in peripheral blood. **C** Tumor cell proliferation was analyzed by full body bioluminescence imaging and subsequent signal quantification. **D** Kaplan Meier curve showing the fraction of animals reaching $1x10^9$ ph/s in bioluminescence imaging. **E** Relative weight progression of all mice (cell line-derived xenografts, PDX) treated with vehicle or VEN following the previous (dotted line) or novel (continuous line) therapy scheme. Vertical lines indicate the therapy start date (d7) and end date (d25; fixed 5x/week for 3 weeks cohort only).

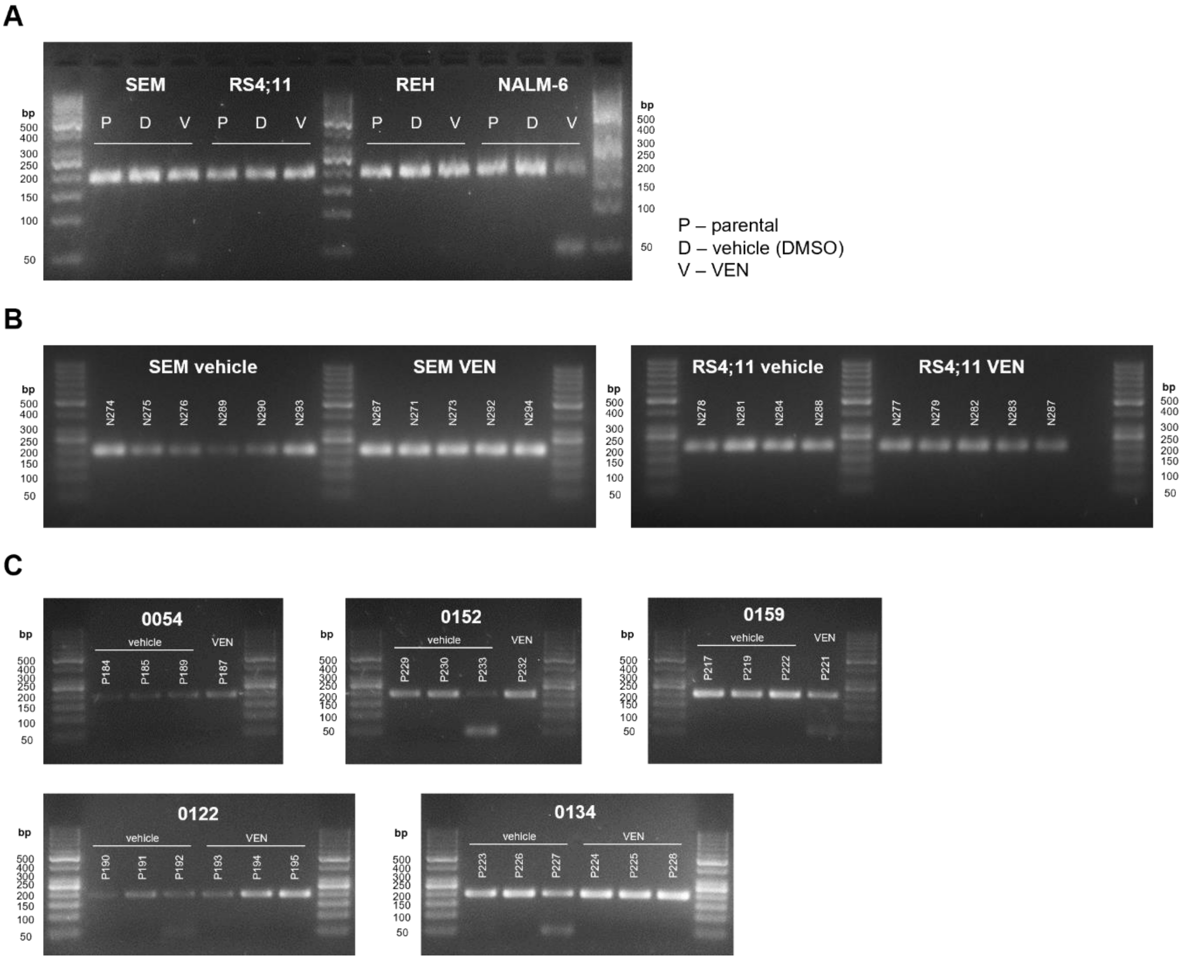

Figure S2: Analysis of FLT3-ITD variants in VEN-resistant cells. Agarose gel electrophoresis of products following PCR amplification of the FLT3 ITD region. **A** Cell lines SEM, RS4;11, REH and NALM-6 were continuously incubated with VEN (V) or vehicle (D) and compared to parental cell lines (P). **B** NSG mice engrafted with SEM or RS4;11 cells were continuously treated with either VEN or vehicle. Each lane indicates an individual animal. 4-6 mice per group. **C** NSG mice were engrafted with cells derived from primary samples 0054, 0122, 0134, 0152 or 0159 and treated with either VEN or vehicle. Each lane indicates an individual animal. 1-3 mice per group. Dim bands around 50 bp depict primer dimers and are no sign of a deletion.

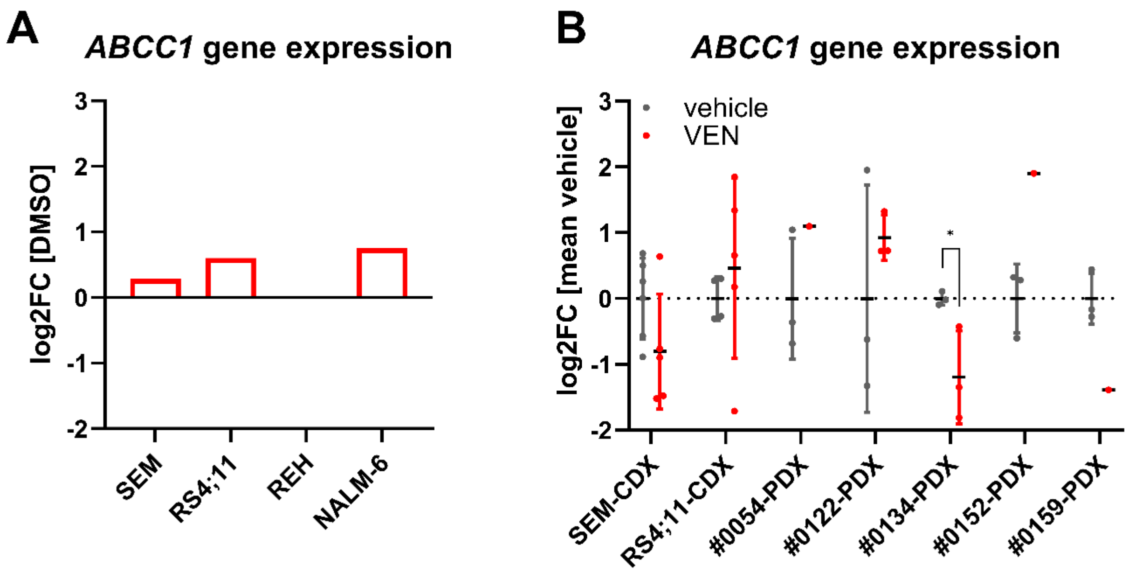

Figure S3: ***ABCC1* gene expression following continuous VEN treatment. A** Log2 fold changes of continuously VEN-incubated cell lines compared to DMSO-incubated controls. **B** Log2 fold changes of VEN- or vehicle-treated animals compared to the mean of the vehicle group. Each dot represents an individual animal. Mean ± SD; unpaired t test; * $p < 0.05$.

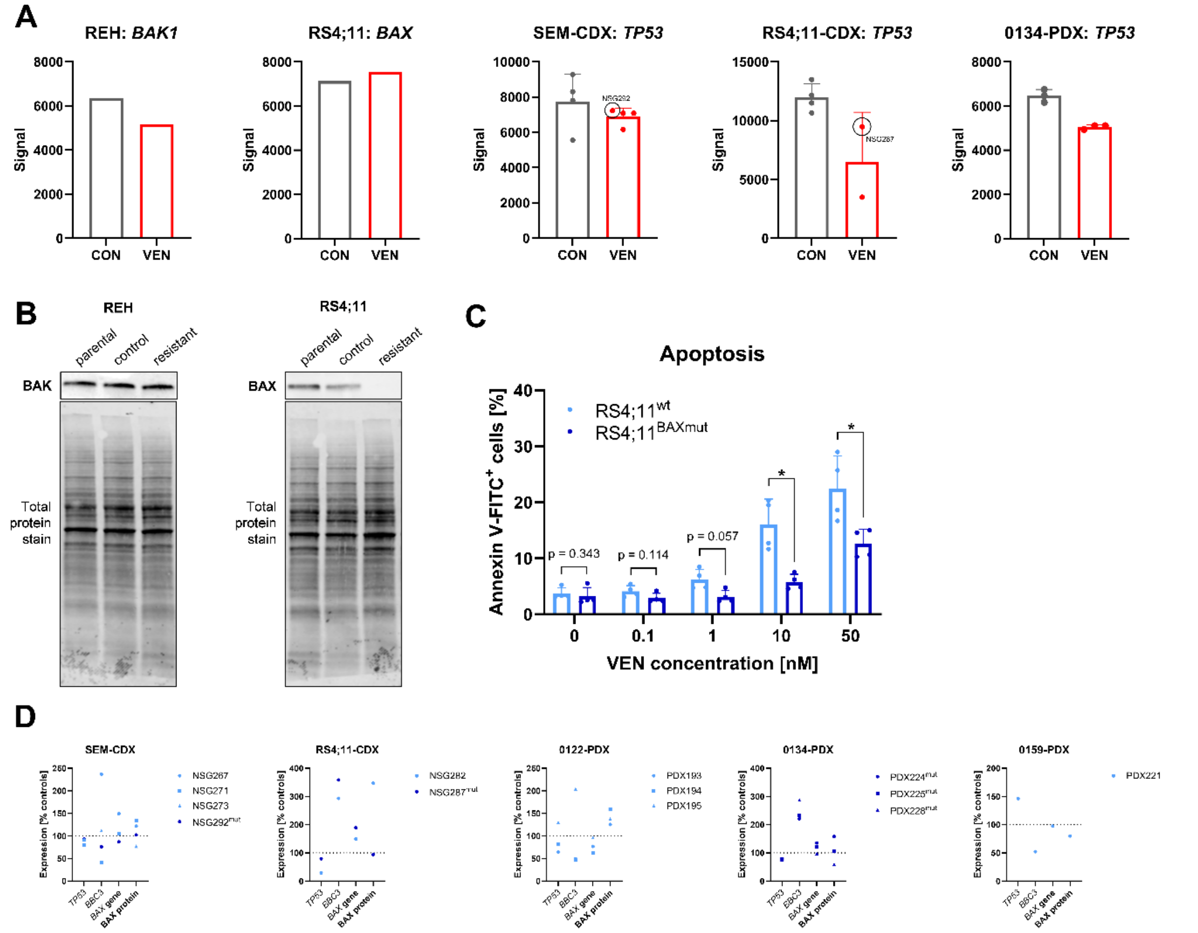

Figure S4: Influence of genomic variants on expression and biological function. A Gene expression in samples with detected variants of the respective gene. Each dot represents an individual animal. For in vivo models with several biological replicates, the sample featuring the genomic aberration is circled. Mean ± SD; Mann-Whitney test; * $p < 0.05$. **B** Immunoblots demonstrating the protein expression of BAK in BAK wild type and BAK mutant REH cells as well as the expression of BAX in BAX mutated and unmutated RS4;11 cells. Total protein stain was used as loading control. **C** Influence of increasing VEN concentrations on apoptosis induction in BAX wild type and BAX-mutated RS4;11 cells. Mean ± SD; paired t test; * $p < 0.05$. **D** Downstream effect of *TP53* mutations on *TP53*, *BBC3* and *BAX* gene expression as well as BAX protein expression. Expression patterns of all VEN-treated animals of each cohort are displayed individually and compared to the mean of all control animals. VEN-treated animals with TP53 mutations are marked as "mut" and painted in darker color.

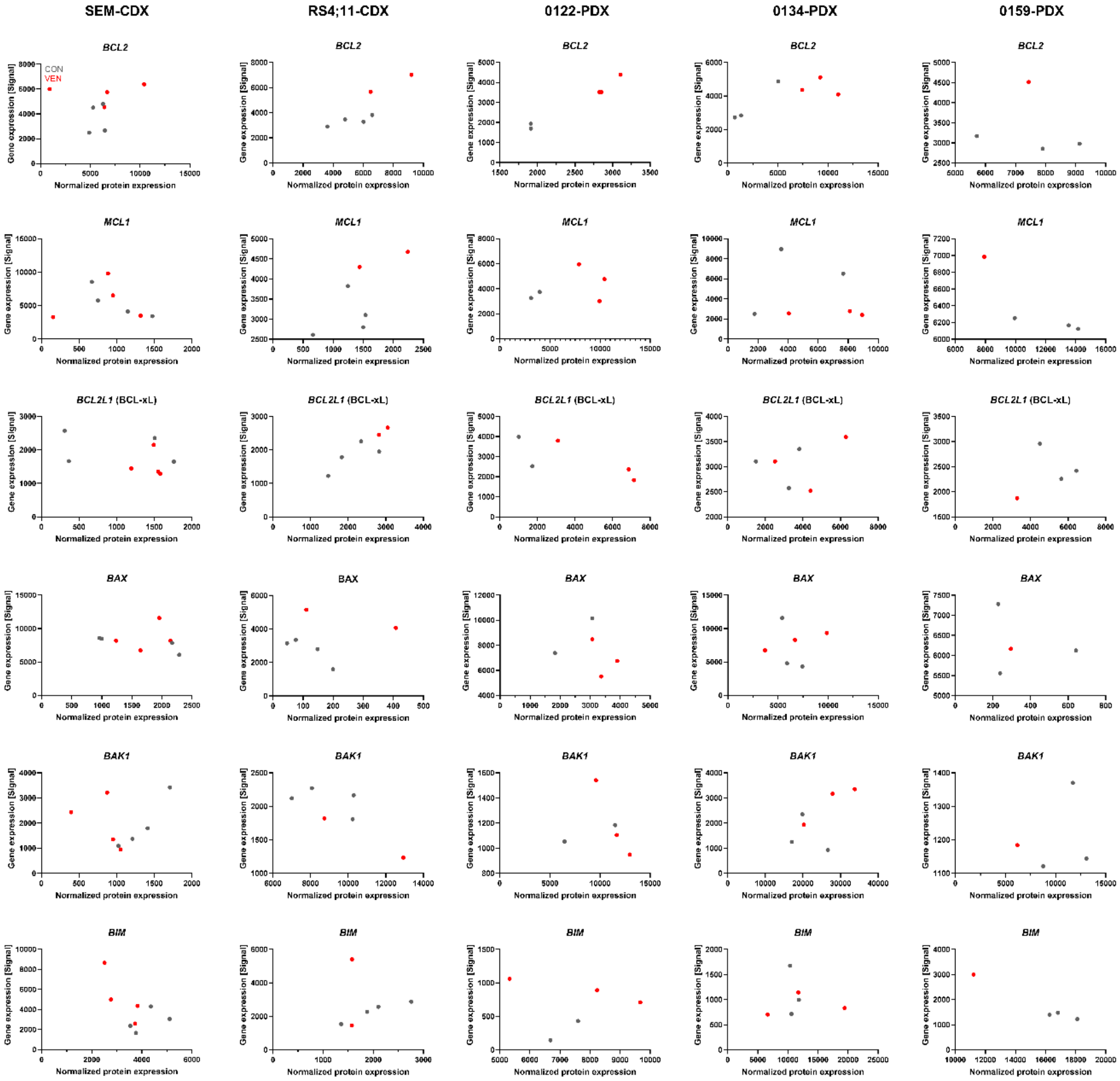

Figure S5: Correlation of gene and protein expression of BCL-2 pathway members BCL-2, MCL-1, BCL-xL, BAX, BAK and BIM in CDX and PDX models. Protein and gene expression of controls (grey) and VEN-resistant animals (red) was assessed by immunoblot normalization and quantification as well as panel RNAseq, respectively. Each dot represents an individual animal.

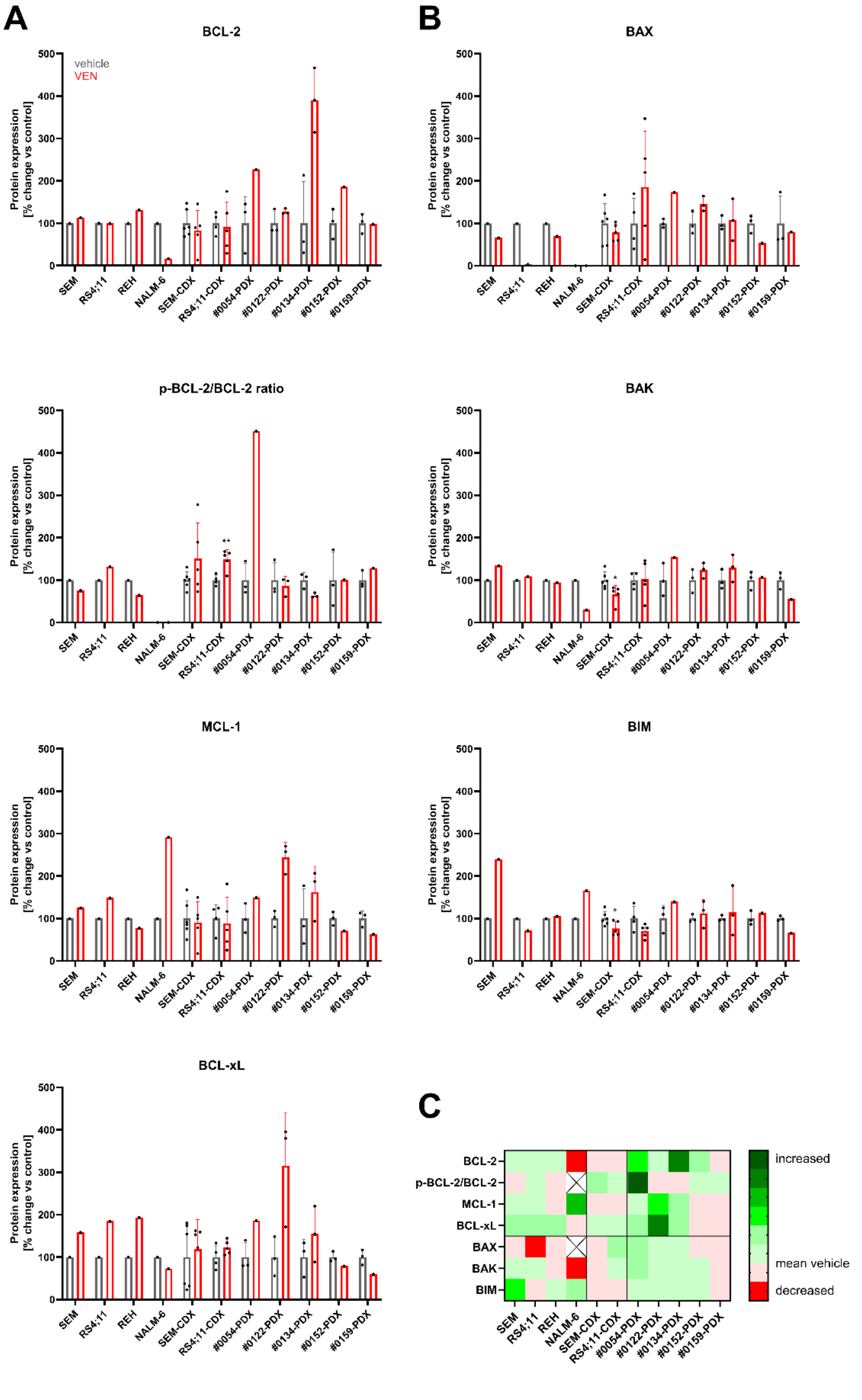

Figure S6: Expression profiles of BCL-2 family proteins in controls and VEN-resistant cell lines, CDX and PDX models. A,B Cell lines SEM, RS4;11, REH and NALM-6, CDX (4-6 mice per group) and PDX mice (1-3 mice per group) were continuously treated with VEN or vehicle. Protein expression of anti-apoptotic (**A**) and pro-apoptotic (**B**) molecules was assessed by immunoblot and quantification was performed using normalization to total protein stain. Statistical analyses were performed for CDX models only due to limited sample numbers in the other cohorts. Mean ± SD; unpaired t test; * $p < 0.05$; ** $p < 0.01$. **C** Heatmap demonstrating the mean protein expression in the VEN-resistant cells compared to the mean of the controls. Green color suggests increased protein expression while red color suggests a reduction. White and crossed fields indicate no detectable protein expression in the respective sample.

A

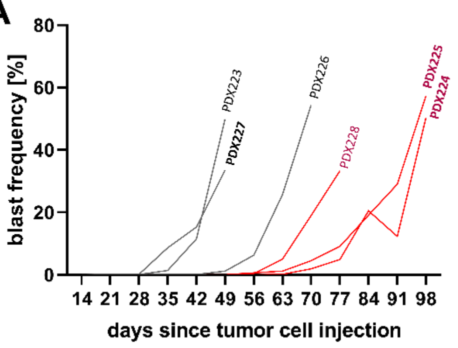

B

	TP53 c.74+38C>G	*TP53* p.Pro72Arg
PDX223 vehicle	81.9 %	85.1 %
PDX226 vehicle	83.7 %	85.1 %
PDX227 vehicle	83.0 %	88.8 %
PDX224 VEN	98.5 %	97.7 %
PDX225 VEN	98.6 %	97.7 %
PDX228 VEN	98.0 %	98.4 %

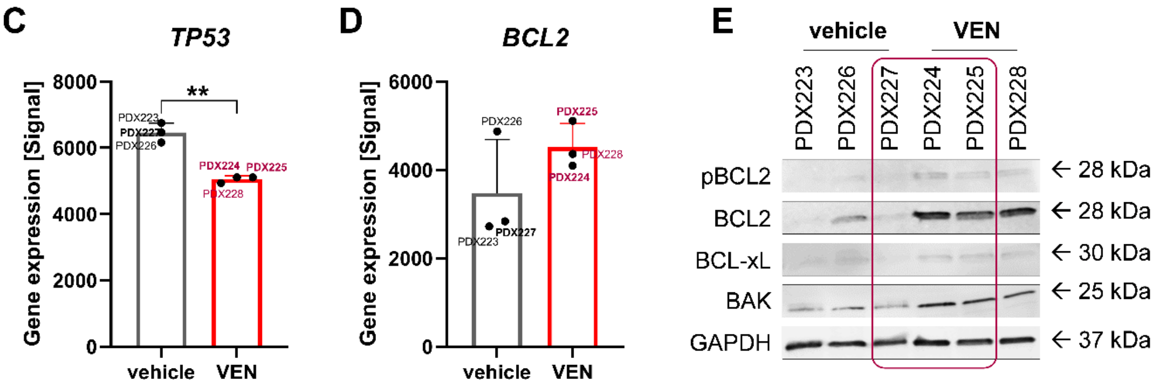

Figure S7: Clinical, genetic and BCL-2 pathway characteristics of #0134 PDX mice. Three mice each were treated with either vehicle (PDX223, PDX226, PDX227) or VEN (PDX224, PDX225, PDX228) until disease progression and defined endpoints (30 % blasts in peripheral blood) were reached. In each subfigure, the animals included in single cell sequencing analyses (PDX227, PDX224, PDX225) are highlighted. **A** Leukemic blast frequency in peripheral blood was measured weekly by flow cytometry examining human CD45/CD19 positive cells. **B** Variant allele frequencies of two *TP53* variants detected in both, controls and VEN-resistant animals. VEN-treated mice experienced clonal expansion of cells harboring this aberration. **C** Gene expression of TP53 in vehicle- and VEN-treated mice was measured by panel RNAseq. Mean ± SD, Welch's test, ** $p < 0.01$. **D** Gene expression of BCL2 in vehicle- and VEN-treated mice was measured by panel RNAseq. Mean ± SD, Welch's test, * $p < 0.05$. **E** Immunoblots demonstrating the modulation of selected BCL-2 family proteins in VEN-naïve and resistant animals.

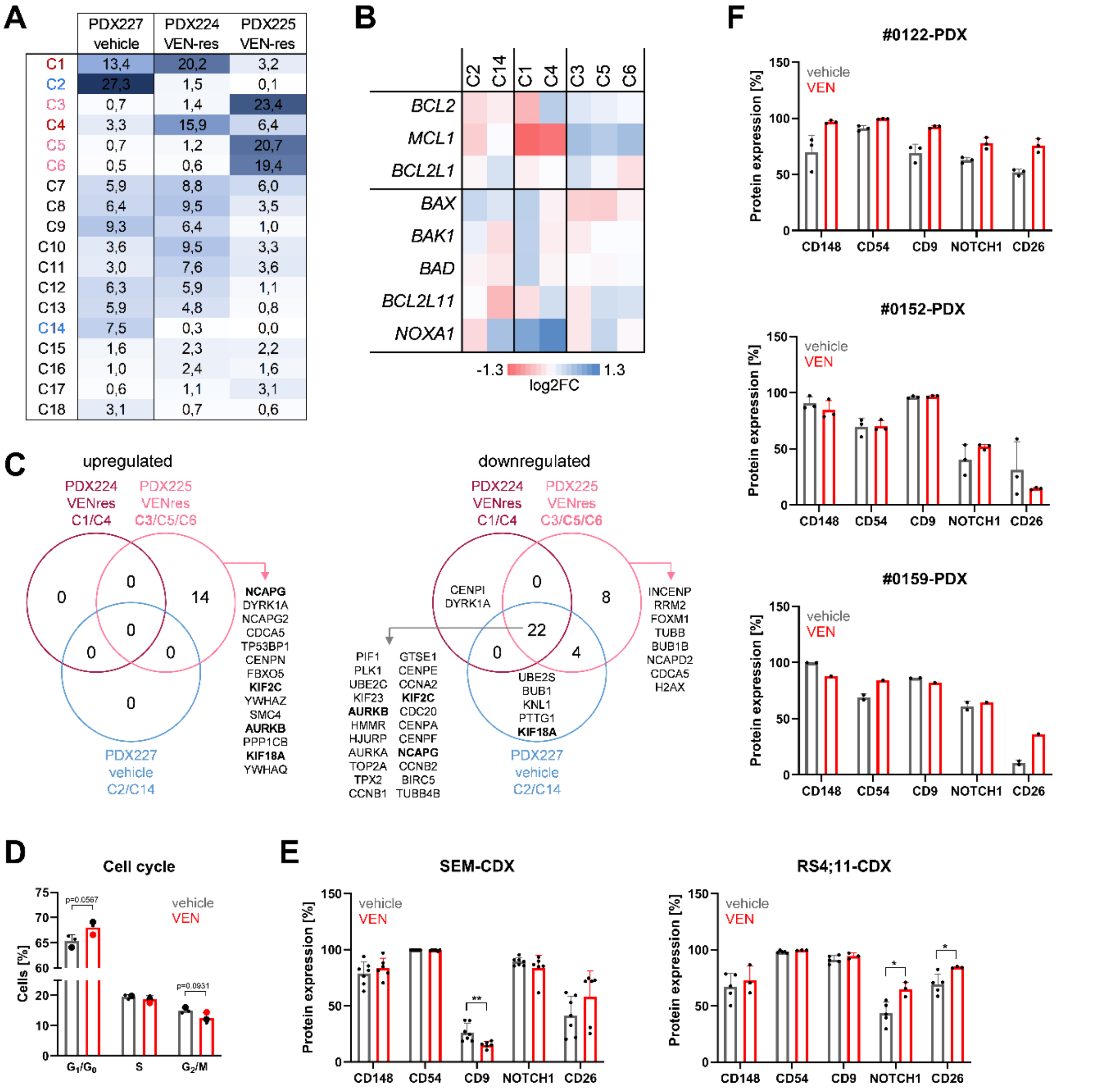

Figure S8: Gene expression profiling and cell cycle analysis of three patient #0134-derived xenografts. PDX227 represents a vehicle-treated control animal while PDX224 and PDX225 continuously received VEN. **A** Proportion of individual clusters in the total of all analyzed cells of each sample. Clusters specific for a sample are color-coded according to the code used in Figure 5 (blue: PDX227-vehicle; dark red: PDX224-VENres; light red: PDX225-VENres). **B** Heatmap representing the expression of key BCL-2 pathway genes across sample-specific clusters. **C** Venn diagrams showing significantly up- (left) and downregulated (right) genes in sample-specific clusters compared to all 18 clusters. **D** Cell cycle analysis of tumor cells derived from spleens of vehicle or VEN-treated animals at experimental endpoints. Mean and individual sample values; each dot represents one of three individual animals per group. Larger dots represent the three animals included in the single cell transcriptomics studies and the red dot indicates sample PDX225 (VENres). Mean ± SD; unpaired t test; * $p < 0.05$. **E,F** Protein expression of molecules involved in tumor microenvironment and cell-cell interaction in CDX (**E**) and PDX (**F**) mice treated with either vehicle or VEN. Each dot represents an individual animal. Mean ± SD; Mann-Whitney test; * $p < 0.05$, ** $p < 0.01$.

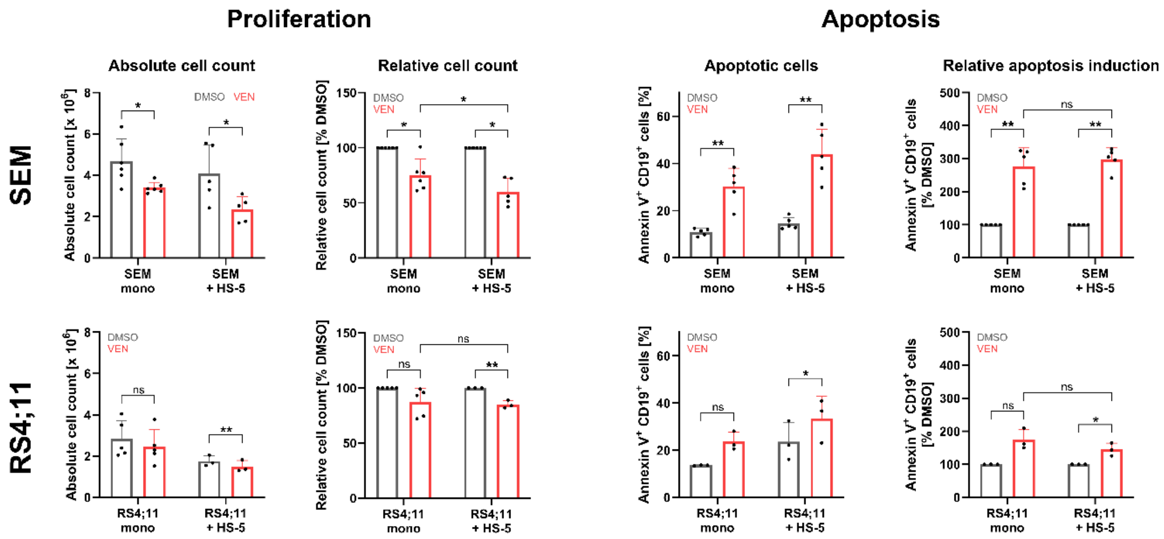

Figure S9: Influence of stromal cell coculturing on leukemic blast proliferation and viability. SEM (upper panel) and RS4;11 cells (lower panel) were cultivated in the presence or absence of HS-5 bone marrow stromal cells, treated with VEN or DMSO (control) and proliferation and apoptosis induction were assessed by trypan blue staining (left) and Annexin V flow cytometry (right), respectively. Mean ± SD; paired t test; * $p < 0.05$.

Table S1: Targeted gene expression panel.

Gene	RefSeq
ACIN1	NM_014977.3
AFF3	NM_002285.3
AIFM1	NM_004208.4
AKT1	NM_005163.2
AKT2	NM_001626.6
AKT3	NM_001370074.1
APAF1	NM_181861.2
ARID1A	NM_006015.6
ASXL1	NM_015338.6
ATF1	NM_005171.5
ATF2	NM_001256090.2
ATM	NM_000051.3
ATRX	NM_000489.5
AVEN	NM_020371.3
BACH2	NM_021813.4
BAD	NM_004322.3
BAK1	NM_001188.4
BAX	NM_004324.4
BBC3	NM_001127240.3
BCL11A	NM_018014.4
BCL2	NM_000633.2
BCL2A1	NM_001114735.2
BCL2L1	NM_001322240.2
BCL2L10	NM_001306168.1
BCL2L11	NM_138622.3
BCL2L13	NM_001270726.1
BCL2L2	NM_004050.4
BCL6	NM_001706.5
BID	NM_197966.2
BIK	NM_001197.5
BIRC2	NM_001166.4
BIRC3	NM_001165.5
BIRC5	NM_001012271.2
BIRC6	NM_016252.3
BIRC7	NM_139317.3
BMF	NM_001003940.2
BRAF	NM_001354609.2
BTG1	NM_001731.3
CASP1	NM_033292.4
CASP10	NM_032974.5
CASP14	NM_012114.3
CASP2	NM_032982.4

CASP3	NM_001354780.1
CASP4	NM_001225.4
CASP5	NM_001136112.2
CASP6	NM_001226.4
CASP7	NM_001267057.1
CASP8	NM_001080125.2
CASP9	NM_001229.5
CCND1	NM_053056.3
CDKN1A	NM_001291549.2
CDKN1B	NM_004064.4
CDKN2A	NM_001195132.1
CDX2	NM_001265.6
CELSR3	NM_001407.3
CFLAR	NM_003879.7
CKDN2B	NM_004936.4
CREB1	NM_134442.5
CREBBP	NM_004380.3
CRLF2	NM_022148.4
CSNK2A1	NM_177559.3
DAP	NM_001291963.2
DAPK1	NM_001288731.2
DAPK3	NM_001348.3
DAXX	NM_001141970.2
DDIT4	NM_019058.4
DEPTOR	NM_022783.4
DIABLO	NM_019887.6
DNMT3A	NM_175629.2
DOT1L	NM_032482.3
DUX4	NM_001306068.2
EIF4EBP1	NM_004095.4
EIF4G2	NM_001418.4
EP300	NM_001429.4
ERG	NM_001136154.1
ESR1	NM_001122742.1
ETV6	NM_001987.5
EZH2	NM_001203248.2
FADD	NM_003824.3
FAF1	NM_007051.3
FAIM	NM_001033030.2
FAS	NM_000043.6
FASLG	NM_000639.3
FHIT	NM_001320900.2
FLCN	NM_001353229.2
FLT1	NM_002019.4
FLT3	NM_004119.3

FOS	NM_005252.4
FOXO1	NM_002015.4
FOXO3	NM_201559.3
FOXO4	NM_005938.4
GAB2	NM_080491.3
GSK3A	NM_019884.3
GSK3B	NM_002093.4
GZMA	NM_006144.4
GZMB	NM_004131.6
HDAC9	NM_001321868.2
HOXA3	NM_153631.2
HOXA5	NM_019102.4
HOXA9	NM_152739.4
HRK	NM_003806.4
HTRA2	NM_013247.4
IKZF1	NM_006060.6
IL10	NM_000572.3
IL7	NM_000880.4
IL7R	NM_002185.5
JAK1	NM_001321853.2
JAK2	NM_001322194.1
JUN	NM_002228.4
KDM6A	NM_001291415.1
KMT2D	NM_003482.3
KRAS	NM_033360.4
LYN	NM_002350.4
MAP2K1	NM_002755.3
MAP2K2	NM_030662.3
MAP2K3	NM_145109.3
MAP2K4	NM_001281435.2
MAP2K6	NM_002758.4
MAP2K7	NM_001297555.2
MAP3K11	NM_002419.4
MAP3K12	NM_001193511.2
MAPK1	NM_002745.4
MAPK10	NM_002753.5
MAPK14	NM_001315.2
MAPK3	NM_002746.3
MAPK9	NM_001364608.2
MAPKAPK2	NM_032960.4
MAX	NM_145113.3
MCL1	NM_021960.5
MDM2	NM_002392.5
MDM4	NM_002393.5
MEIS1	NM_002398.3
MELK	NM_014791.4

MKNK2	NM_199054.2
MTOR	NM_004958.4
MXD1	NM_001202513.2
MYC	NM_001354870.1
NF1	NM_001042492.3
NFKB1	NM_003998.4
NOTCH1	NM_017617.5
NOTCH2	NM_024408.4
NOTCH3	NM_000435.3
NOTCH4	NM_004557.4
NRAS	NM_002524.5
PAWR	NM_002583.4
PAX5	NM_016734.3
PDCD4	NM_001199492.1
PDCD6IP	NM_001162429.2
PDK1	NM_001278549.2
PEA15	NM_001297576.2
PHLDA3	NM_012396.5
PIDD1	NM_145886.4
PIK3CA	NM_006218.4
PIK3R1	NM_181523.3
PMAIP1	NM_021127.3
POLR2A	NM_000937.5
POLR2B	NM_000938.3
PRKCD	NM_001354676.2
PTEN	NM_001304717.5
PTPN11	NM_002834.4
PTPRS	NM_002850.4
PTRH2	NM_001015509.2
RAG1	NM_000448.2
RB1	NM_000321.2
RGCC	NM_014059.3
RHEB	NM_005614.4
RICTOR	NM_001285439.2
ROS1	NM_002944.2
RPS6KA1	NM_002953.4
RPS6KA3	NM_004586.3
RPS6KB1	NM_001369672.1
RPS6KB2	NM_003952.3
RUNX1	NM_001754.4
SETD2	NM_014159.6
SH3RF1	NM_020870.4
SIVA1	NM_006427.4
STAT3	NM_139276.2
STAT5A	NM_003152.3
STAT5B	NM_012448.4

STK17B	NM_004226.4
STK26	NM_016542.4
STK3	NM_001256312.2
STK4	NM_006282.5
TET2	NM_001127208.2
THEM4	NM_053055.5
TNF	NM_000594.4
TNFRSF10A	NM_003844.4
TNFRSF10C	NM_003841.4
TNFRSF13B	NM_012452.3
TNFRSF13C	NM_052945.3
TNFRSF1A	NM_001065.4
TNFRSF6B	NM_003823.4
TNFSF10	NM_003810.4
TNFSF13	NM_003808.3
TNFSF13B	NM_006573.4
TP53	NM_000546.5
TRADD	NM_001323552.2
TRAF3	NM_003300.4
TRAF6	NM_145803.3
TRIM24	NM_015905.3
TSC1	NM_000368.5
TSC2	NM_000548.5
TWIST1	NM_000474.4
VCP	NM_007126.5
VDAC1	NM_003374.3
VDAC2	NM_001184783.2
WEE1	NM_003390.4
WWOX	NM_016373.4
XAF1	NM_001353135.1
XIAP	NM_001204401.1
YAP1	NM_001282101.1
YWHAB	NM_003404.5
YWHAE	NM_006761.5
YWHAZ	NM_003406.4

Table S2: Genetic variants detected in BAK1, BAX, BCL2 and TP53 in cell lines, CDX and PDX samples.

Sample	Group	Chromosomal location	Gene	RefSeq	coding sequence	amino acid	dbSNP	ClinVar	Allele frequency
SEM-parental	cell line	chr6:33541507	BAK1	NM_001188.4	c.*73T>C	p.?	rs511515	NA	99.85
SEM-DMSO control	cell line	chr6:33541507	BAK1	NM_001188.4	c.*73T>C	p.?	rs511515	NA	99.95
SEM-VEN-resistant	cell line	chr6:33541507	BAK1	NM_001188.4	c.*73T>C	p.?	rs511515	NA	99.85
SEM-parental	cell line	chr6:33545340	BAK1	NM_001188.4	c.42C>T	p.Cys14=	rs2227925	NA	99.25
SEM-DMSO control	cell line	chr6:33545340	BAK1	NM_001188.4	c.42C>T	p.Cys14=	rs2227925	NA	98.95
SEM-VEN-resistant	cell line	chr6:33545340	BAK1	NM_001188.4	c.42C>T	p.Cys14=	rs2227925	NA	99.6
SEM-parental	cell line	chr17:7577407	TP53	NM_000546.6	c.782+92T>G	p.?	rs12951053	germline benign	49.77
SEM-DMSO control	cell line	chr17:7577407	TP53	NM_000546.6	c.782+92T>G	p.?	rs12951053	germline benign	47.75
SEM-VEN-resistant	cell line	chr17:7577407	TP53	NM_000546.6	c.782+92T>G	p.?	rs12951053	germline benign	47.85
SEM-parental	cell line	chr17:7577427	TP53	NM_000546.6	c.782+72C>T	p.?	rs12947788	germline benign	50.45
SEM-DMSO control	cell line	chr17:7577427	TP53	NM_000546.6	c.782+72C>T	p.?	rs12947788	germline benign	48.7
SEM-VEN-resistant	cell line	chr17:7577427	TP53	NM_000546.6	c.782+72C>T	p.?	rs12947788	germline benign	47.4
SEM-parental	cell line	chr17:7577538	TP53	NM_000546.6	c.743G>A	p.Arg248Gln	rs11540652	germline/somatic (likely) pathogenic	49.7
SEM-DMSO control	cell line	chr17:7577538	TP53	NM_000546.6	c.743G>A	p.Arg248Gln	rs11540652	germline/somatic (likely) pathogenic	51.08
SEM-VEN-resistant	cell line	chr17:7577538	TP53	NM_000546.6	c.743G>A	p.Arg248Gln	rs11540652	germline/somatic (likely) pathogenic	52.93
SEM-parental	cell line	chr17:7578210	TP53	NM_000546.6	c.639A>G	p.Arg213=	rs1800372	germline (likely) benign	48.35
SEM-DMSO control	cell line	chr17:7578210	TP53	NM_000546.6	c.639A>G	p.Arg213=	rs1800372	germline (likely) benign	49.1
SEM-VEN-resistant	cell line	chr17:7578210	TP53	NM_000546.6	c.639A>G	p.Arg213=	rs1800372	germline (likely) benign	52.08
SEM-parental	cell line	chr17:7579312	TP53	NM_000546.6	c.375G>A	p.Thr125=	rs55863639	germline pathgenic	51.15
SEM-DMSO control	cell line	chr17:7579312	TP53	NM_000546.6	c.375G>A	p.Thr125=	rs55863639	germline pathgenic	49.52
SEM-VEN-resistant	cell line	chr17:7579312	TP53	NM_000546.6	c.375G>A	p.Thr125=	rs55863639	germline pathgenic	51.1
SEM-parental	cell line	chr17:7579472	TP53	NM_000546.6	c.215C>G	p.Pro72Arg	rs1042522	germline/somatic benign	51.57
SEM-DMSO control	cell line	chr17:7579472	TP53	NM_000546.6	c.215C>G	p.Pro72Arg	rs1042522	germline/somatic benign	50.64
SEM-VEN-resistant	cell line	chr17:7579472	TP53	NM_000546.6	c.215C>G	p.Pro72Arg	rs1042522	germline/somatic benign	52.46
SEM-parental	cell line	chr17:7579633	TP53	NM_000546.6	c.96+41_97-54delACCTGGAGGGCTGGGG	p.?	NA	NA	94.33
SEM-DMSO control	cell line	chr17:7579633	TP53	NM_000546.6	c.96+41_97-54delACCTGGAGGGCTGGGG	p.?	NA	NA	96.92
SEM-VEN-resistant	cell line	chr17:7579633	TP53	NM_000546.6	c.96+41_97-54delACCTGGAGGGCTGGGG	p.?	NA	NA	93.45
SEM-parental	cell line	chr17:7579801	TP53	NM_000546.6	c.74+38C>G	p.?	rs1642785	germline benign	51.38
SEM-DMSO control	cell line	chr17:7579801	TP53	NM_000546.6	c.74+38C>G	p.?	rs1642785	germline benign	52.03
SEM-VEN-resistant	cell line	chr17:7579801	TP53	NM_000546.6	c.74+38C>G	p.?	rs1642785	germline benign	53.46
SEM-parental	cell line	chr18:60985879	BCL2	NM_000633.3	c.21A>G	p.Thr7=	rs1801018	NA	49.5
SEM-DMSO control	cell line	chr18:60985879	BCL2	NM_000633.3	c.21A>G	p.Thr7=	rs1801018	NA	53.48
SEM-VEN-resistant	cell line	chr18:60985879	BCL2	NM_000633.3	c.21A>G	p.Thr7=	rs1801018	NA	51.15
SEM-parental	cell line	chr19:49458262	BAX	NM_004324.4	c.34+43C>T	p.?	rs4645881	NA	38.27
SEM-DMSO control	cell line	chr19:49458262	BAX	NM_004324.4	c.34+43C>T	p.?	rs4645881	NA	39.95
SEM-VEN-resistant	cell line	chr19:49458262	BAX	NM_004324.4	c.34+43C>T	p.?	rs4645881	NA	36.52
SEM-parental	cell line	chr19:49459104	BAX	NM_004324.4	c.233+14A>G	p.?	rs1805419	NA	55.66
SEM-DMSO control	cell line	chr19:49459104	BAX	NM_004324.4	c.233+14A>G	p.?	rs1805419	NA	53.53
SEM-VEN-resistant	cell line	chr19:49459104	BAX	NM_004324.4	c.233+14A>G	p.?	rs1805419	NA	52.96
SEM-parental	cell line	chr19:49464971	BAX	NM_004324.4	c.*617G>A	p.?	rs704243	NA	99.95
SEM-DMSO control	cell line	chr19:49464971	BAX	NM_004324.4	c.*617G>A	p.?	rs704243	NA	99.7
SEM-VEN-resistant	cell line	chr19:49464971	BAX	NM_004324.4	c.*617G>A	p.?	rs704243	NA	99.8
RS4;11-parental	cell line	chr6:33541507	BAK1	NM_001188.4	c.*73T>C	p.?	rs511515	NA	51.5
RS4;11-DMSO control	cell line	chr6:33541507	BAK1	NM_001188.4	c.*73T>C	p.?	rs511515	NA	49.3
RS4;11-VEN-resistant	cell line	chr6:33541507	BAK1	NM_001188.4	c.*73T>C	p.?	rs511515	NA	49.19
RS4;11-parental	cell line	chr6:33545340	BAK1	NM_001188.4	c.42C>T	p.Cys14=	rs2227925	NA	52.35
RS4;11-DMSO control	cell line	chr6:33545340	BAK1	NM_001188.4	c.42C>T	p.Cys14=	rs2227925	NA	44.44
RS4;11-VEN-resistant	cell line	chr6:33545340	BAK1	NM_001188.4	c.42C>T	p.Cys14=	rs2227925	NA	49.57
RS4;11-parental	cell line	chr17:7577520	TP53	NM_000546.6	c.761T>C	p.Ile254Thr	rs1330865474	germline pathogenic	4.3
RS4;11-parental	cell line	chr17:7579472	TP53	NM_000546.6	c.215C>G	p.Pro72Arg	rs1042522	germline/somatic benign	96.95
RS4;11-DMSO control	cell line	chr17:7579472	TP53	NM_000546.6	c.215C>G	p.Pro72Arg	rs1042522	germline/somatic benign	91.69
RS4;11-VEN-resistant	cell line	chr17:7579472	TP53	NM_000546.6	c.215C>G	p.Pro72Arg	rs1042522	germline/somatic benign	97.15
RS4;11-parental	cell line	chr17:7579633	TP53	NM_000546.6	c.96+41_97-54delACCTGGAGGGCTGGGG	p.?	NA	NA	99.15
RS4;11-DMSO control	cell line	chr17:7579633	TP53	NM_000546.6	c.96+41_97-54delACCTGGAGGGCTGGGG	p.?	NA	NA	93.07
RS4;11-VEN-resistant	cell line	chr17:7579633	TP53	NM_000546.6	c.96+41_97-54delACCTGGAGGGCTGGGG	p.?	NA	NA	97.24
RS4;11-parental	cell line	chr17:7579801	TP53	NM_000546.6	c.74+38C>G	p.?	rs1642785	germline benign	98.44
RS4;11-DMSO control	cell line	chr17:7579801	TP53	NM_000546.6	c.74+38C>G	p.?	rs1642785	germline benign	93.67
RS4;11-VEN-resistant	cell line	chr17:7579801	TP53	NM_000546.6	c.74+38C>G	p.?	rs1642785	germline benign	97.89
RS4;11-parental	cell line	chr18:60985600	BCL2	NM_000633.3	c.300C>T	p.Ala100=	rs61733416	NA	54.28
RS4;11-DMSO control	cell line	chr18:60985600	BCL2	NM_000633.3	c.300C>T	p.Ala100=	rs61733416	NA	51.95
RS4;11-VEN-resistant	cell line	chr18:60985600	BCL2	NM_000633.3	c.300C>T	p.Ala100=	rs61733416	NA	54.8
RS4;11-parental	cell line	chr18:60985879	BCL2	NM_000633.3	c.21A>G	p.Thr7=	rs1801018	NA	50.48
RS4;11-DMSO control	cell line	chr18:60985879	BCL2	NM_000633.3	c.21A>G	p.Thr7=	rs1801018	NA	49.8
RS4;11-VEN-resistant	cell line	chr18:60985879	BCL2	NM_000633.3	c.21A>G	p.Thr7=	rs1801018	NA	53.31
RS4;11-parental	cell line	chr19:49458262	BAX	NM_004324.4	c.34+43C>T	p.?	rs4645881	NA	99.75
RS4;11-DMSO control	cell line	chr19:49458262	BAX	NM_004324.4	c.34+43C>T	p.?	rs4645881	NA	99.85
RS4;11-VEN-resistant	cell line	chr19:49458262	BAX	NM_004324.4	c.34+43C>T	p.?	rs4645881	NA	99.85
RS4;11-VEN-resistant	cell line	chr19:49458838	BAX	NM_004324.4	c.68G>T	p.Gly23Val	NA	NA	34.12
RS4;11-parental	cell line	chr19:49459104	BAX	NM_004324.4	c.233+14A>G	p.?	rs1805419	NA	99.95
RS4;11-DMSO control	cell line	chr19:49459104	BAX	NM_004324.4	c.233+14A>G	p.?	rs1805419	NA	100
RS4;11-VEN-resistant	cell line	chr19:49459104	BAX	NM_004324.4	c.233+14A>G	p.?	rs1805419	NA	100
RS4;11-DMSO control	cell line	chr19:49464830	BAX	NM_004324.4	c.*476C>T	p.?	rs760456972	NA	5.4
RS4;11-DMSO control	cell line	chr19:49464889	BAX	NM_004324.4	c.*535G>A	p.?	NA	NA	6.25
RS4;11-VEN-resistant	cell line	chr19:49464891	BAX	NM_004324.4	c.*537T>C	p.?	NA	NA	4.95
RS4;11-parental	cell line	chr19:49464971	BAX	NM_004324.4	c.*617G>A	p.?	rs704243	NA	99.8
RS4;11-DMSO control	cell line	chr19:49464971	BAX	NM_004324.4	c.*617G>A	p.?	rs704243	NA	99.84
RS4;11-VEN-resistant	cell line	chr19:49464971	BAX	NM_004324.4	c.*617G>A	p.?	rs704243	NA	99.95
REH-parental	cell line	chr6:33541507	BAK1	NM_001188.4	c.*73T>C	p.?	rs511515	NA	99.75
REH-DMSO control	cell line	chr6:33541507	BAK1	NM_001188.4	c.*73T>C	p.?	rs511515	NA	99.86
REH-VEN-resistant	cell line	chr6:33541507	BAK1	NM_001188.4	c.*73T>C	p.?	rs511515	NA	99.95
REH-VEN-resistant	cell line	chr6:33541661	BAK1	NM_001188.4	c.554_555insA	p.Asn185LysfsTer120	NA	NA	4.31
REH-parental	cell line	chr17:7576569	TP53	NM_000546.6	c.993+284C>T	p.?	rs554738122	germline likely benign	4.15
REH-DMSO control	cell line	chr17:7576569	TP53	NM_000546.6	c.993+284C>T	p.?	rs554738122	germline likely benign	6.15
REH-VEN-resistant	cell line	chr17:7576569	TP53	NM_000546.6	c.993+284C>T	p.?	rs554738122	germline likely benign	5.6
REH-parental	cell line	chr17:7578389	TP53	NM_000546.6	c.541C>T	p.Arg181Cys	rs587782596	germline (likely) pathogenic	6
REH-parental	cell line	chr17:7579472	TP53	NM_000546.6	c.215C>G	p.Pro72Arg	rs1042522	germline/somatic benign	50.54
REH-DMSO control	cell line	chr17:7579472	TP53	NM_000546.6	c.215C>G	p.Pro72Arg	rs1042522	germline/somatic benign	50.1
REH-VEN-resistant	cell line	chr17:7579472	TP53	NM_000546.6	c.215C>G	p.Pro72Arg	rs1042522	germline/somatic benign	49.49
REH-parental	cell line	chr17:7579619	TP53	NM_000546.6	c.97-29C>A	p.?	rs17883323	germline benign	49.65
REH-DMSO control	cell line	chr17:7579619	TP53	NM_000546.6	c.97-29C>A	p.?	rs17883323	germline benign	50.35
REH-VEN-resistant	cell line	chr17:7579619	TP53	NM_000546.6	c.97-29C>A	p.?	rs17883323	germline benign	46.92
REH-parental	cell line	chr17:7579633	TP53	NM_000546.6	c.96+41_97-54delACCTGGAGGGCTGGGG	p.?	NA	NA	97.14
REH-DMSO control	cell line	chr17:7579633	TP53	NM_000546.6	c.96+41_97-54delACCTGGAGGGCTGGGG	p.?	NA	NA	96.16
REH-VEN-resistant	cell line	chr17:7579633	TP53	NM_000546.6	c.96+41_97-54delACCTGGAGGGCTGGGG	p.?	NA	NA	97.32
REH-parental	cell line	chr17:7579801	TP53	NM_000546.6	c.74+38C>G	p.?	rs1642785	germline benign	54.09
REH-DMSO control	cell line	chr17:7579801	TP53	NM_000546.6	c.74+38C>G	p.?	rs1642785	germline benign	54.14
REH-VEN-resistant	cell line	chr17:7579801	TP53	NM_000546.6	c.74+38C>G	p.?	rs1642785	germline benign	51.48
REH-parental	cell line	chr18:60985879	BCL2	NM_000633.3	c.21A>G	p.Thr7=	rs1801018	NA	52.03
REH-DMSO control	cell line	chr18:60985879	BCL2	NM_000633.3	c.21A>G	p.Thr7=	rs1801018	NA	47.05
REH-VEN-resistant	cell line	chr18:60985879	BCL2	NM_000633.3	c.21A>G	p.Thr7=	rs1801018	NA	51.08
REH-parental	cell line	chr19:49458262	BAX	NM_004324.4	c.34+43C>T	p.?	rs4645881	NA	99.8
REH-DMSO control	cell line	chr19:49458262	BAX	NM_004324.4	c.34+43C>T	p.?	rs4645881	NA	99.75
REH-VEN-resistant	cell line	chr19:49458262	BAX	NM_004324.4	c.34+43C>T	p.?	rs4645881	NA	99.75
REH-parental	cell line	chr19:49459104	BAX	NM_004324.4	c.233+14A>G	p.?	rs1805419	NA	99.9
REH-DMSO control	cell line	chr19:49459104	BAX	NM_004324.4	c.233+14A>G	p.?	rs1805419	NA	99.95
REH-VEN-resistant	cell line	chr19:49459104	BAX	NM_004324.4	c.233+14A>G	p.?	rs1805419	NA	99.85
REH-parental	cell line	chr19:49464971	BAX	NM_004324.4	c.*617G>A	p.?	rs704243	NA	99.8
REH-DMSO control	cell line	chr19:49464971	BAX	NM_004324.4	c.*617G>A	p.?	rs704243	NA	99.73
REH-VEN-resistant	cell line	chr19:49464971	BAX	NM_004324.4	c.*617G>A	p.?	rs704243	NA	99.57
NALM-6-parental	cell line	chr6:33541507	BAK1	NM_001188.4	c.*73T>C	p.?	rs511515	NA	49
NALM-6-DMSO control	cell line	chr6:33541507	BAK1	NM_001188.4	c.*73T>C	p.?	rs511515	NA	47.9
NALM-6-VEN-resistant	cell line	chr6:33541507	BAK1	NM_001188.4	c.*73T>C	p.?	rs511515	NA	49.95
NALM-6-parental	cell line	chr17:7579472	TP53	NM_000546.6	c.215C>G	p.Pro72Arg	rs1042522	germline/somatic benign	48.73

NALM-6-DMSO control	cell line	chr17:7579472	TP53	NM_000546.6	c.215C>G	p.Pro72Arg	rs1042522	germline/somatic benign	52.77
NALM-6-VEN-resistant	cell line	chr17:7579472	TP53	NM_000546.6	c.215C>G	p.Pro72Arg	rs1042522	germline/somatic benign	48.93
NALM-6-parental	cell line	chr17:7579633	TP53	NM_000546.6	c.96+41_97-54delACCTGGAGGGCTGGGG	p.?	NA	NA	53.35
NALM-6-DMSO control	cell line	chr17:7579633	TP53	NM_000546.6	c.96+41_97-54delACCTGGAGGGCTGGGG	p.?	NA	NA	53.51
NALM-6-VEN-resistant	cell line	chr17:7579633	TP53	NM_000546.6	c.96+41_97-54delACCTGGAGGGCTGGGG	p.?	NA	NA	54.42
NALM-6-parental	cell line	chr17:7579801	TP53	NM_000546.6	c.74+38C>G	p.?	rs1642785	germline benign	51.89
NALM-6-DMSO control	cell line	chr17:7579801	TP53	NM_000546.6	c.74+38C>G	p.?	rs1642785	germline benign	52.59
NALM-6-VEN-resistant	cell line	chr17:7579801	TP53	NM_000546.6	c.74+38C>G	p.?	rs1642785	germline benign	50.65
NALM-6-parental	cell line	chr19:49458262	BAX	NM_004324.4	c.34+43C>T	p.?	rs4645881	NA	99.8
NALM-6-DMSO control	cell line	chr19:49458262	BAX	NM_004324.4	c.34+43C>T	p.?	rs4645881	NA	99.8
NALM-6-VEN-resistant	cell line	chr19:49458262	BAX	NM_004324.4	c.34+43C>T	p.?	rs4645881	NA	99.45
NALM-6-parental	cell line	chr19:49459104	BAX	NM_004324.4	c.233+14A>G	p.?	rs1805419	NA	100
NALM-6-DMSO control	cell line	chr19:49459104	BAX	NM_004324.4	c.233+14A>G	p.?	rs1805419	NA	99.95
NALM-6-VEN-resistant	cell line	chr19:49459104	BAX	NM_004324.4	c.233+14A>G	p.?	rs1805419	NA	100
NALM-6-parental	cell line	chr19:49464830	BAX	NM_004324.4	c.*476C>T	p.?	rs760456972	NA	53.75
NALM-6-DMSO control	cell line	chr19:49464830	BAX	NM_004324.4	c.*476C>T	p.?	rs760456972	NA	48.6
NALM-6-VEN-resistant	cell line	chr19:49464830	BAX	NM_004324.4	c.*476C>T	p.?	rs760456972	NA	50.33
NALM-6-parental	cell line	chr19:49464971	BAX	NM_004324.4	c.*617G>A	p.?	rs704243	NA	99.8
NALM-6-DMSO control	cell line	chr19:49464971	BAX	NM_004324.4	c.*617G>A	p.?	rs704243	NA	99.65
NALM-6-VEN-resistant	cell line	chr19:49464971	BAX	NM_004324.4	c.*617G>A	p.?	rs704243	NA	99.95
NSG274	SEM-CDX vehicle	chr6:33541507	BAK1	NM_001188.4	c.*73T>C	p.?	rs511515	NA	99.85
NSG275	SEM-CDX vehicle	chr6:33541507	BAK1	NM_001188.4	c.*73T>C	p.?	rs511515	NA	99.85
NSG276	SEM-CDX vehicle	chr6:33541507	BAK1	NM_001188.4	c.*73T>C	p.?	rs511515	NA	99.75
NSG289	SEM-CDX vehicle	chr6:33541507	BAK1	NM_001188.4	c.*73T>C	p.?	rs511515	NA	99.95
NSG290	SEM-CDX vehicle	chr6:33541507	BAK1	NM_001188.4	c.*73T>C	p.?	rs511515	NA	99.75
NSG293	SEM-CDX vehicle	chr6:33541507	BAK1	NM_001188.4	c.*73T>C	p.?	rs511515	NA	92.92
NSG267	SEM-CDX VEN	chr6:33541507	BAK1	NM_001188.4	c.*73T>C	p.?	rs511515	NA	99.95
NSG271	SEM-CDX VEN	chr6:33541507	BAK1	NM_001188.4	c.*73T>C	p.?	rs511515	NA	99.55
NSG273	SEM-CDX VEN	chr6:33541507	BAK1	NM_001188.4	c.*73T>C	p.?	rs511515	NA	99.8
NSG292	SEM-CDX VEN	chr6:33541507	BAK1	NM_001188.4	c.*73T>C	p.?	rs511515	NA	99.9
NSG294	SEM-CDX VEN	chr6:33541507	BAK1	NM_001188.4	c.*73T>C	p.?	rs511515	NA	99.75
NSG274	SEM-CDX vehicle	chr6:33545340	BAK1	NM_001188.4	c.42C>T	p.Cys14=	rs2227925	NA	99.95
NSG275	SEM-CDX vehicle	chr6:33545340	BAK1	NM_001188.4	c.42C>T	p.Cys14=	rs2227925	NA	99.15
NSG276	SEM-CDX vehicle	chr6:33545340	BAK1	NM_001188.4	c.42C>T	p.Cys14=	rs2227925	NA	99.1
NSG289	SEM-CDX vehicle	chr6:33545340	BAK1	NM_001188.4	c.42C>T	p.Cys14=	rs2227925	NA	99.85
NSG290	SEM-CDX vehicle	chr6:33545340	BAK1	NM_001188.4	c.42C>T	p.Cys14=	rs2227925	NA	99.6
NSG293	SEM-CDX vehicle	chr6:33545340	BAK1	NM_001188.4	c.42C>T	p.Cys14=	rs2227925	NA	99.3
NSG267	SEM-CDX VEN	chr6:33545340	BAK1	NM_001188.4	c.42C>T	p.Cys14=	rs2227925	NA	99.85
NSG271	SEM-CDX VEN	chr6:33545340	BAK1	NM_001188.4	c.42C>T	p.Cys14=	rs2227925	NA	99.3
NSG273	SEM-CDX VEN	chr6:33545340	BAK1	NM_001188.4	c.42C>T	p.Cys14=	rs2227925	NA	99.65
NSG292	SEM-CDX VEN	chr6:33545340	BAK1	NM_001188.4	c.42C>T	p.Cys14=	rs2227925	NA	99.5
NSG294	SEM-CDX VEN	chr6:33545340	BAK1	NM_001188.4	c.42C>T	p.Cys14=	rs2227925	NA	99.65
NSG274	SEM-CDX vehicle	chr17:7577407	TP53	NM_000546.6	c.782+92T>G	p.?	rs12951053	germline benign	47.82
NSG275	SEM-CDX vehicle	chr17:7577407	TP53	NM_000546.6	c.782+92T>G	p.?	rs12951053	germline benign	46.72
NSG276	SEM-CDX vehicle	chr17:7577407	TP53	NM_000546.6	c.782+92T>G	p.?	rs12951053	germline benign	46.49
NSG289	SEM-CDX vehicle	chr17:7577407	TP53	NM_000546.6	c.782+92T>G	p.?	rs12951053	germline benign	46.24
NSG290	SEM-CDX vehicle	chr17:7577407	TP53	NM_000546.6	c.782+92T>G	p.?	rs12951053	germline benign	47.27
NSG293	SEM-CDX vehicle	chr17:7577407	TP53	NM_000546.6	c.782+92T>G	p.?	rs12951053	germline benign	44.52
NSG267	SEM-CDX VEN	chr17:7577407	TP53	NM_000546.6	c.782+92T>G	p.?	rs12951053	germline benign	46
NSG271	SEM-CDX VEN	chr17:7577407	TP53	NM_000546.6	c.782+92T>G	p.?	rs12951053	germline benign	49.8
NSG273	SEM-CDX VEN	chr17:7577407	TP53	NM_000546.6	c.782+92T>G	p.?	rs12951053	germline benign	48.3
NSG292	SEM-CDX VEN	chr17:7577407	TP53	NM_000546.6	c.782+92T>G	p.?	rs12951053	germline benign	46.4
NSG294	SEM-CDX VEN	chr17:7577407	TP53	NM_000546.6	c.782+92T>G	p.?	rs12951053	germline benign	51.93
NSG274	SEM-CDX vehicle	chr17:7577427	TP53	NM_000546.6	c.782+72C>T	p.?	rs12947788	germline benign	48.62
NSG275	SEM-CDX vehicle	chr17:7577427	TP53	NM_000546.6	c.782+72C>T	p.?	rs12947788	germline benign	48.6
NSG276	SEM-CDX vehicle	chr17:7577427	TP53	NM_000546.6	c.782+72C>T	p.?	rs12947788	germline benign	49.42
NSG289	SEM-CDX vehicle	chr17:7577427	TP53	NM_000546.6	c.782+72C>T	p.?	rs12947788	germline benign	45.55
NSG290	SEM-CDX vehicle	chr17:7577427	TP53	NM_000546.6	c.782+72C>T	p.?	rs12947788	germline benign	46.4
NSG293	SEM-CDX vehicle	chr17:7577427	TP53	NM_000546.6	c.782+72C>T	p.?	rs12947788	germline benign	44.42
NSG267	SEM-CDX VEN	chr17:7577427	TP53	NM_000546.6	c.782+72C>T	p.?	rs12947788	germline benign	46.15
NSG271	SEM-CDX VEN	chr17:7577427	TP53	NM_000546.6	c.782+72C>T	p.?	rs12947788	germline benign	48
NSG273	SEM-CDX VEN	chr17:7577427	TP53	NM_000546.6	c.782+72C>T	p.?	rs12947788	germline benign	46.6
NSG292	SEM-CDX VEN	chr17:7577427	TP53	NM_000546.6	c.782+72C>T	p.?	rs12947788	germline benign	47.27
NSG294	SEM-CDX VEN	chr17:7577427	TP53	NM_000546.6	c.782+72C>T	p.?	rs12947788	germline benign	52.6
NSG274	SEM-CDX vehicle	chr17:7577538	TP53	NM_000546.6	c.743G>A	p.Arg248Gln	rs11540652	germline/somatic (likely) pathogenic	52.68
NSG275	SEM-CDX vehicle	chr17:7577538	TP53	NM_000546.6	c.743G>A	p.Arg248Gln	rs11540652	germline/somatic (likely) pathogenic	51.4
NSG276	SEM-CDX vehicle	chr17:7577538	TP53	NM_000546.6	c.743G>A	p.Arg248Gln	rs11540652	germline/somatic (likely) pathogenic	53.65
NSG289	SEM-CDX vehicle	chr17:7577538	TP53	NM_000546.6	c.743G>A	p.Arg248Gln	rs11540652	germline/somatic (likely) pathogenic	51.13
NSG290	SEM-CDX vehicle	chr17:7577538	TP53	NM_000546.6	c.743G>A	p.Arg248Gln	rs11540652	germline/somatic (likely) pathogenic	53
NSG293	SEM-CDX vehicle	chr17:7577538	TP53	NM_000546.6	c.743G>A	p.Arg248Gln	rs11540652	germline/somatic (likely) pathogenic	55.33
NSG267	SEM-CDX VEN	chr17:7577538	TP53	NM_000546.6	c.743G>A	p.Arg248Gln	rs11540652	germline/somatic (likely) pathogenic	52.85
NSG271	SEM-CDX VEN	chr17:7577538	TP53	NM_000546.6	c.743G>A	p.Arg248Gln	rs11540652	germline/somatic (likely) pathogenic	50
NSG273	SEM-CDX VEN	chr17:7577538	TP53	NM_000546.6	c.743G>A	p.Arg248Gln	rs11540652	germline/somatic (likely) pathogenic	50.9
NSG292	SEM-CDX VEN	chr17:7577538	TP53	NM_000546.6	c.743G>A	p.Arg248Gln	rs11540652	germline/somatic (likely) pathogenic	54.4
NSG294	SEM-CDX VEN	chr17:7577538	TP53	NM_000546.6	c.743G>A	p.Arg248Gln	rs11540652	germline/somatic (likely) pathogenic	47.42
NSG274	SEM-CDX vehicle	chr17:7578210	TP53	NM_000546.6	c.639A>G	p.Arg213=	rs1800372	germline (likely) benign	47.7
NSG275	SEM-CDX vehicle	chr17:7578210	TP53	NM_000546.6	c.639A>G	p.Arg213=	rs1800372	germline (likely) benign	48.9
NSG276	SEM-CDX vehicle	chr17:7578210	TP53	NM_000546.6	c.639A>G	p.Arg213=	rs1800372	germline (likely) benign	48.8
NSG289	SEM-CDX vehicle	chr17:7578210	TP53	NM_000546.6	c.639A>G	p.Arg213=	rs1800372	germline (likely) benign	48.4
NSG290	SEM-CDX vehicle	chr17:7578210	TP53	NM_000546.6	c.639A>G	p.Arg213=	rs1800372	germline (likely) benign	50.15
NSG293	SEM-CDX vehicle	chr17:7578210	TP53	NM_000546.6	c.639A>G	p.Arg213=	rs1800372	germline (likely) benign	48.25
NSG267	SEM-CDX VEN	chr17:7578210	TP53	NM_000546.6	c.639A>G	p.Arg213=	rs1800372	germline (likely) benign	49.52
NSG271	SEM-CDX VEN	chr17:7578210	TP53	NM_000546.6	c.639A>G	p.Arg213=	rs1800372	germline (likely) benign	50.23
NSG273	SEM-CDX VEN	chr17:7578210	TP53	NM_000546.6	c.639A>G	p.Arg213=	rs1800372	germline (likely) benign	50.31
NSG292	SEM-CDX VEN	chr17:7578210	TP53	NM_000546.6	c.639A>G	p.Arg213=	rs1800372	germline (likely) benign	48.97
NSG294	SEM-CDX VEN	chr17:7578210	TP53	NM_000546.6	c.639A>G	p.Arg213=	rs1800372	germline (likely) benign	48.05
NSG274	SEM-CDX vehicle	chr17:7579312	TP53	NM_000546.6	c.375G>A	p.Thr125=	rs55863639	germline pathgenic	48.62
NSG275	SEM-CDX vehicle	chr17:7579312	TP53	NM_000546.6	c.375G>A	p.Thr125=	rs55863639	germline pathgenic	50.73
NSG276	SEM-CDX vehicle	chr17:7579312	TP53	NM_000546.6	c.375G>A	p.Thr125=	rs55863639	germline pathgenic	49.42
NSG289	SEM-CDX vehicle	chr17:7579312	TP53	NM_000546.6	c.375G>A	p.Thr125=	rs55863639	germline pathgenic	49.7
NSG290	SEM-CDX vehicle	chr17:7579312	TP53	NM_000546.6	c.375G>A	p.Thr125=	rs55863639	germline pathgenic	50.63
NSG293	SEM-CDX vehicle	chr17:7579312	TP53	NM_000546.6	c.375G>A	p.Thr125=	rs55863639	germline pathgenic	47.2
NSG267	SEM-CDX VEN	chr17:7579312	TP53	NM_000546.6	c.375G>A	p.Thr125=	rs55863639	germline pathgenic	49
NSG271	SEM-CDX VEN	chr17:7579312	TP53	NM_000546.6	c.375G>A	p.Thr125=	rs55863639	germline pathgenic	47.65
NSG273	SEM-CDX VEN	chr17:7579312	TP53	NM_000546.6	c.375G>A	p.Thr125=	rs55863639	germline pathgenic	49.5
NSG292	SEM-CDX VEN	chr17:7579312	TP53	NM_000546.6	c.375G>A	p.Thr125=	rs55863639	germline pathgenic	49.27
NSG294	SEM-CDX VEN	chr17:7579312	TP53	NM_000546.6	c.375G>A	p.Thr125=	rs55863639	germline pathgenic	50.93
NSG274	SEM-CDX vehicle	chr17:7579472	TP53	NM_000546.6	c.215C>G	p.Pro72Arg	rs1042522	germline/somatic benign	56.13
NSG275	SEM-CDX vehicle	chr17:7579472	TP53	NM_000546.6	c.215C>G	p.Pro72Arg	rs1042522	germline/somatic benign	54.28
NSG276	SEM-CDX vehicle	chr17:7579472	TP53	NM_000546.6	c.215C>G	p.Pro72Arg	rs1042522	germline/somatic benign	59.42
NSG289	SEM-CDX vehicle	chr17:7579472	TP53	NM_000546.6	c.215C>G	p.Pro72Arg	rs1042522	germline/somatic benign	52.67
NSG290	SEM-CDX vehicle	chr17:7579472	TP53	NM_000546.6	c.215C>G	p.Pro72Arg	rs1042522	germline/somatic benign	56.05
NSG293	SEM-CDX vehicle	chr17:7579472	TP53	NM_000546.6	c.215C>G	p.Pro72Arg	rs1042522	germline/somatic benign	64.79
NSG267	SEM-CDX VEN	chr17:7579472	TP53	NM_000546.6	c.215C>G	p.Pro72Arg	rs1042522	germline/somatic benign	53.3
NSG271	SEM-CDX VEN	chr17:7579472	TP53	NM_000546.6	c.215C>G	p.Pro72Arg	rs1042522	germline/somatic benign	52.89
NSG273	SEM-CDX VEN	chr17:7579472	TP53	NM_000546.6	c.215C>G	p.Pro72Arg	rs1042522	germline/somatic benign	60.85
NSG292	SEM-CDX VEN	chr17:7579472	TP53	NM_000546.6	c.215C>G	p.Pro72Arg	rs1042522	germline/somatic benign	55.34
NSG294	SEM-CDX VEN	chr17:7579472	TP53	NM_000546.6	c.215C>G	p.Pro72Arg	rs1042522	germline/somatic benign	53.42
NSG292	SEM-CDX VEN	chr17:7579493	TP53	NM_000546.6	c.193_194insC	p.Arg65ThrfsTer84	NA	NA	3.12
NSG274	SEM-CDX vehicle	chr17:7579633	TP53	NM_000546.6	c.96+41_97-54delACCTGGAGGGCTGGGG	p.?	NA	NA	96.64
NSG275	SEM-CDX vehicle	chr17:7579633	TP53	NM_000546.6	c.96+41_97-54delACCTGGAGGGCTGGGG	p.?	NA	NA	96.26
NSG276	SEM-CDX vehicle	chr17:7579633	TP53	NM_000546.6	c.96+41_97-54delACCTGGAGGGCTGGGG	p.?	NA	NA	95.71
NSG289	SEM-CDX vehicle	chr17:7579633	TP53	NM_000546.6	c.96+41_97-54delACCTGGAGGGCTGGGG	p.?	NA	NA	97.18
NSG290	SEM-CDX vehicle	chr17:7579633	TP53	NM_000546.6	c.96+41_97-54delACCTGGAGGGCTGGGG	p.?	NA	NA	95.47
NSG293	SEM-CDX vehicle	chr17:7579633	TP53	NM_000546.6	c.96+41_97-54delACCTGGAGGGCTGGGG	p.?	NA	NA	98.1
NSG267	SEM-CDX VEN	chr17:7579633	TP53	NM_000546.6	c.96+41_97-54delACCTGGAGGGCTGGGG	p.?	NA	NA	96.73
NSG271	SEM-CDX VEN	chr17:7579633	TP53	NM_000546.6	c.96+41_97-54delACCTGGAGGGCTGGGG	p.?	NA	NA	96.79

NSG273	SEM-CDX VEN	chr17:7579633	TP53	NM_000546.6	c.96+41_97-54delACCTGGAGGGCTGGGG	p.?	NA	NA	92.56
NSG292	SEM-CDX VEN	chr17:7579633	TP53	NM_000546.6	c.96+41_97-54delACCTGGAGGGCTGGGG	p.?	NA	NA	96.85
NSG294	SEM-CDX VEN	chr17:7579633	TP53	NM_000546.6	c.96+41_97-54delACCTGGAGGGCTGGGG	p.?	NA	NA	87.11
NSG274	SEM-CDX vehicle	chr17:7579801	TP53	NM_000546.6	c.74+38C>G	p.?	rs1642785	germline benign	52.32
NSG275	SEM-CDX vehicle	chr17:7579801	TP53	NM_000546.6	c.74+38C>G	p.?	rs1642785	germline benign	50.28
NSG276	SEM-CDX vehicle	chr17:7579801	TP53	NM_000546.6	c.74+38C>G	p.?	rs1642785	germline benign	54.34
NSG289	SEM-CDX vehicle	chr17:7579801	TP53	NM_000546.6	c.74+38C>G	p.?	rs1642785	germline benign	52.22
NSG290	SEM-CDX vehicle	chr17:7579801	TP53	NM_000546.6	c.74+38C>G	p.?	rs1642785	germline benign	52.78
NSG293	SEM-CDX vehicle	chr17:7579801	TP53	NM_000546.6	c.74+38C>G	p.?	rs1642785	germline benign	47.16
NSG267	SEM-CDX VEN	chr17:7579801	TP53	NM_000546.6	c.74+38C>G	p.?	rs1642785	germline benign	55.28
NSG271	SEM-CDX VEN	chr17:7579801	TP53	NM_000546.6	c.74+38C>G	p.?	rs1642785	germline benign	51.23
NSG273	SEM-CDX VEN	chr17:7579801	TP53	NM_000546.6	c.74+38C>G	p.?	rs1642785	germline benign	47.03
NSG292	SEM-CDX VEN	chr17:7579801	TP53	NM_000546.6	c.74+38C>G	p.?	rs1642785	germline benign	52.12
NSG294	SEM-CDX VEN	chr17:7579801	TP53	NM_000546.6	c.74+38C>G	p.?	rs1642785	germline benign	51.51
NSG267	SEM-CDX VEN	chr18:60985399	BCL2	NM_000633.3	c.501G>A	p.Ser167=	NA	NA	3.36
NSG271	SEM-CDX VEN	chr18:60985399	BCL2	NM_000633.3	c.501G>A	p.Ser167=	NA	NA	5.5
NSG292	SEM-CDX VEN	chr18:60985399	BCL2	NM_000633.3	c.501G>A	p.Ser167=	NA	NA	2.95
NSG267	SEM-CDX VEN	chr18:60985410	BCL2	NM_000633.3	c.490C>A	p.Arg164=	rs996326337	NA	3.4
NSG271	SEM-CDX VEN	chr18:60985410	BCL2	NM_000633.3	c.490C>A	p.Arg164=	rs996326337	NA	5.3
NSG292	SEM-CDX VEN	chr18:60985410	BCL2	NM_000633.3	c.490C>A	p.Arg164=	rs996326337	NA	3.65
NSG267	SEM-CDX VEN	chr18:60985475	BCL2	NM_000633.3	c.420C>T	p.Asp140=	rs779104297	NA	5.74
NSG271	SEM-CDX VEN	chr18:60985475	BCL2	NM_000633.3	c.420C>T	p.Asp140=	rs779104297	NA	7.55
NSG292	SEM-CDX VEN	chr18:60985475	BCL2	NM_000633.3	c.420C>T	p.Asp140=	rs779104297	NA	7.45
NSG267	SEM-CDX VEN	chr18:60985492	BCL2	NM_000633.3	c.408G>A	p.Glu136=	NA	NA	6.61
NSG271	SEM-CDX VEN	chr18:60985492	BCL2	NM_000633.3	c.408G>A	p.Glu136=	NA	NA	9.42
NSG292	SEM-CDX VEN	chr18:60985492	BCL2	NM_000633.3	c.408G>A	p.Glu136=	NA	NA	8.1
NSG267	SEM-CDX VEN	chr18:60985521	BCL2	NM_000633.3	c.379C>A	p.Arg127=	NA	NA	6.61
NSG271	SEM-CDX VEN	chr18:60985521	BCL2	NM_000633.3	c.379C>A	p.Arg127=	NA	NA	8.43
NSG292	SEM-CDX VEN	chr18:60985521	BCL2	NM_000633.3	c.379C>A	p.Arg127=	NA	NA	8.15
NSG267	SEM-CDX VEN	chr18:60985549	BCL2	NM_000633.3	c.351C>T	p.Ser117=	NA	NA	6.21
NSG271	SEM-CDX VEN	chr18:60985549	BCL2	NM_000633.3	c.351C>T	p.Ser117=	NA	NA	8.08
NSG292	SEM-CDX VEN	chr18:60985549	BCL2	NM_000633.3	c.351C>T	p.Ser117=	NA	NA	7.35
NSG292	SEM-CDX VEN	chr18:60985561	BCL2	NM_000633.3	c.339C>A	p.Ala113=	NA	NA	6.06
NSG292	SEM-CDX VEN	chr18:60985570	BCL2	NM_000633.3	c.327_330delCCGCinsTCGT	p.[Arg109=;Arg110=]	NA	NA	6.44
NSG274	SEM-CDX vehicle	chr18:60985879	BCL2	NM_000633.3	c.21A>G	p.Thr7=	rs1801018	NA	51.6
NSG275	SEM-CDX vehicle	chr18:60985879	BCL2	NM_000633.3	c.21A>G	p.Thr7=	rs1801018	NA	53.04
NSG276	SEM-CDX vehicle	chr18:60985879	BCL2	NM_000633.3	c.21A>G	p.Thr7=	rs1801018	NA	55.26
NSG289	SEM-CDX vehicle	chr18:60985879	BCL2	NM_000633.3	c.21A>G	p.Thr7=	rs1801018	NA	53.33
NSG290	SEM-CDX vehicle	chr18:60985879	BCL2	NM_000633.3	c.21A>G	p.Thr7=	rs1801018	NA	50.15
NSG293	SEM-CDX vehicle	chr18:60985879	BCL2	NM_000633.3	c.21A>G	p.Thr7=	rs1801018	NA	53.96
NSG267	SEM-CDX VEN	chr18:60985879	BCL2	NM_000633.3	c.21A>G	p.Thr7=	rs1801018	NA	50.48
NSG271	SEM-CDX VEN	chr18:60985879	BCL2	NM_000633.3	c.21A>G	p.Thr7=	rs1801018	NA	46.59
NSG273	SEM-CDX VEN	chr18:60985879	BCL2	NM_000633.3	c.21A>G	p.Thr7=	rs1801018	NA	51.8
NSG292	SEM-CDX VEN	chr18:60985879	BCL2	NM_000633.3	c.21A>G	p.Thr7=	rs1801018	NA	50.35
NSG294	SEM-CDX VEN	chr18:60985879	BCL2	NM_000633.3	c.21A>G	p.Thr7=	rs1801018	NA	51.68
NSG274	SEM-CDX vehicle	chr19:49458262	BAX	NM_004324.4	c.34+43C>T	p.?	rs4645881	NA	37.49
NSG275	SEM-CDX vehicle	chr19:49458262	BAX	NM_004324.4	c.34+43C>T	p.?	rs4645881	NA	42.17
NSG276	SEM-CDX vehicle	chr19:49458262	BAX	NM_004324.4	c.34+43C>T	p.?	rs4645881	NA	44.07
NSG289	SEM-CDX vehicle	chr19:49458262	BAX	NM_004324.4	c.34+43C>T	p.?	rs4645881	NA	43.12
NSG290	SEM-CDX vehicle	chr19:49458262	BAX	NM_004324.4	c.34+43C>T	p.?	rs4645881	NA	38.04
NSG293	SEM-CDX vehicle	chr19:49458262	BAX	NM_004324.4	c.34+43C>T	p.?	rs4645881	NA	45.37
NSG267	SEM-CDX VEN	chr19:49458262	BAX	NM_004324.4	c.34+43C>T	p.?	rs4645881	NA	39.82
NSG271	SEM-CDX VEN	chr19:49458262	BAX	NM_004324.4	c.34+43C>T	p.?	rs4645881	NA	35.6
NSG273	SEM-CDX VEN	chr19:49458262	BAX	NM_004324.4	c.34+43C>T	p.?	rs4645881	NA	34.35
NSG292	SEM-CDX VEN	chr19:49458262	BAX	NM_004324.4	c.34+43C>T	p.?	rs4645881	NA	41.97
NSG294	SEM-CDX VEN	chr19:49458262	BAX	NM_004324.4	c.34+43C>T	p.?	rs4645881	NA	43.65
NSG274	SEM-CDX vehicle	chr19:49459104	BAX	NM_004324.4	c.233+14A>G	p.?	rs1805419	NA	55.83
NSG275	SEM-CDX vehicle	chr19:49459104	BAX	NM_004324.4	c.233+14A>G	p.?	rs1805419	NA	51.75
NSG276	SEM-CDX vehicle	chr19:49459104	BAX	NM_004324.4	c.233+14A>G	p.?	rs1805419	NA	53.13
NSG289	SEM-CDX vehicle	chr19:49459104	BAX	NM_004324.4	c.233+14A>G	p.?	rs1805419	NA	50.48
NSG290	SEM-CDX vehicle	chr19:49459104	BAX	NM_004324.4	c.233+14A>G	p.?	rs1805419	NA	50.08
NSG293	SEM-CDX vehicle	chr19:49459104	BAX	NM_004324.4	c.233+14A>G	p.?	rs1805419	NA	49.45
NSG267	SEM-CDX VEN	chr19:49459104	BAX	NM_004324.4	c.233+14A>G	p.?	rs1805419	NA	52.15
NSG271	SEM-CDX VEN	chr19:49459104	BAX	NM_004324.4	c.233+14A>G	p.?	rs1805419	NA	51.38
NSG273	SEM-CDX VEN	chr19:49459104	BAX	NM_004324.4	c.233+14A>G	p.?	rs1805419	NA	52.88
NSG292	SEM-CDX VEN	chr19:49459104	BAX	NM_004324.4	c.233+14A>G	p.?	rs1805419	NA	52.15
NSG294	SEM-CDX VEN	chr19:49459104	BAX	NM_004324.4	c.233+14A>G	p.?	rs1805419	NA	50.33
NSG274	SEM-CDX vehicle	chr19:49464971	BAX	NM_004324.4	c.*617G>A	p.?	rs704243	NA	99.9
NSG275	SEM-CDX vehicle	chr19:49464971	BAX	NM_004324.4	c.*617G>A	p.?	rs704243	NA	99.9
NSG276	SEM-CDX vehicle	chr19:49464971	BAX	NM_004324.4	c.*617G>A	p.?	rs704243	NA	99.9
NSG289	SEM-CDX vehicle	chr19:49464971	BAX	NM_004324.4	c.*617G>A	p.?	rs704243	NA	99.75
NSG290	SEM-CDX vehicle	chr19:49464971	BAX	NM_004324.4	c.*617G>A	p.?	rs704243	NA	99.85
NSG293	SEM-CDX vehicle	chr19:49464971	BAX	NM_004324.4	c.*617G>A	p.?	rs704243	NA	99.5
NSG267	SEM-CDX VEN	chr19:49464971	BAX	NM_004324.4	c.*617G>A	p.?	rs704243	NA	99.8
NSG271	SEM-CDX VEN	chr19:49464971	BAX	NM_004324.4	c.*617G>A	p.?	rs704243	NA	99.65
NSG273	SEM-CDX VEN	chr19:49464971	BAX	NM_004324.4	c.*617G>A	p.?	rs704243	NA	99.95
NSG292	SEM-CDX VEN	chr19:49464971	BAX	NM_004324.4	c.*617G>A	p.?	rs704243	NA	99.8
NSG294	SEM-CDX VEN	chr19:49464971	BAX	NM_004324.4	c.*617G>A	p.?	rs704243	NA	99.85
NSG278	RS4;11-CDX vehicle	chr6:33541507	BAK1	NM_001188.4	c.*73T>C	p.?	rs511515	NA	49.32
NSG281	RS4;11-CDX vehicle	chr6:33541507	BAK1	NM_001188.4	c.*73T>C	p.?	rs511515	NA	50.5
NSG284	RS4;11-CDX vehicle	chr6:33541507	BAK1	NM_001188.4	c.*73T>C	p.?	rs511515	NA	51.05
NSG288	RS4;11-CDX vehicle	chr6:33541507	BAK1	NM_001188.4	c.*73T>C	p.?	rs511515	NA	49.75
NSG277	RS4;11-CDX VEN	chr6:33541507	BAK1	NM_001188.4	c.*73T>C	p.?	rs511515	NA	51.75
NSG279	RS4;11-CDX VEN	chr6:33541507	BAK1	NM_001188.4	c.*73T>C	p.?	rs511515	NA	49.82
NSG282	RS4;11-CDX VEN	chr6:33541507	BAK1	NM_001188.4	c.*73T>C	p.?	rs511515	NA	47.55
NSG283	RS4;11-CDX VEN	chr6:33541507	BAK1	NM_001188.4	c.*73T>C	p.?	rs511515	NA	48.1
NSG287	RS4;11-CDX VEN	chr6:33541507	BAK1	NM_001188.4	c.*73T>C	p.?	rs511515	NA	50.73
NSG278	RS4;11-CDX vehicle	chr6:33543085	BAK1	NM_001188.4	c.340A>G	p.Ile114Val	NA	NA	50.9
NSG281	RS4;11-CDX vehicle	chr6:33543085	BAK1	NM_001188.4	c.340A>G	p.Ile114Val	NA	NA	51.58
NSG284	RS4;11-CDX vehicle	chr6:33543085	BAK1	NM_001188.4	c.340A>G	p.Ile114Val	NA	NA	50.85
NSG288	RS4;11-CDX vehicle	chr6:33543085	BAK1	NM_001188.4	c.340A>G	p.Ile114Val	NA	NA	49.67
NSG277	RS4;11-CDX VEN	chr6:33543085	BAK1	NM_001188.4	c.340A>G	p.Ile114Val	NA	NA	51.7
NSG279	RS4;11-CDX VEN	chr6:33543085	BAK1	NM_001188.4	c.340A>G	p.Ile114Val	NA	NA	50.6
NSG282	RS4;11-CDX VEN	chr6:33543085	BAK1	NM_001188.4	c.340A>G	p.Ile114Val	NA	NA	52.13
NSG283	RS4;11-CDX VEN	chr6:33543085	BAK1	NM_001188.4	c.340A>G	p.Ile114Val	NA	NA	51.3
NSG287	RS4;11-CDX VEN	chr6:33543085	BAK1	NM_001188.4	c.340A>G	p.Ile114Val	NA	NA	52.73
NSG278	RS4;11-CDX vehicle	chr6:33545340	BAK1	NM_001188.4	c.42C>T	p.Cys14=	rs2227925	NA	52.25
NSG281	RS4;11-CDX vehicle	chr6:33545340	BAK1	NM_001188.4	c.42C>T	p.Cys14=	rs2227925	NA	52.1
NSG284	RS4;11-CDX vehicle	chr6:33545340	BAK1	NM_001188.4	c.42C>T	p.Cys14=	rs2227925	NA	50.4
NSG288	RS4;11-CDX vehicle	chr6:33545340	BAK1	NM_001188.4	c.42C>T	p.Cys14=	rs2227925	NA	48.97
NSG277	RS4;11-CDX VEN	chr6:33545340	BAK1	NM_001188.4	c.42C>T	p.Cys14=	rs2227925	NA	52.13
NSG279	RS4;11-CDX VEN	chr6:33545340	BAK1	NM_001188.4	c.42C>T	p.Cys14=	rs2227925	NA	49.27
NSG282	RS4;11-CDX VEN	chr6:33545340	BAK1	NM_001188.4	c.42C>T	p.Cys14=	rs2227925	NA	49.12
NSG283	RS4;11-CDX VEN	chr6:33545340	BAK1	NM_001188.4	c.42C>T	p.Cys14=	rs2227925	NA	48.42
NSG287	RS4;11-CDX VEN	chr6:33545340	BAK1	NM_001188.4	c.42C>T	p.Cys14=	rs2227925	NA	49.37
NSG287	RS4;11-CDX VEN	chr17:7573899	TP53	NM_000546.6	c.1100+28C>A	p.?	NA	NA	5.81
NSG283	RS4;11-CDX VEN	chr17:7578530	TP53	NM_000546.6	c.400T>C	p.Phe134Leu	rs267605077	uncertain significance	33.03
NSG278	RS4;11-CDX vehicle	chr17:7579472	TP53	NM_000546.6	c.215C>G	p.Pro72Arg	rs1042522	germline/somatic benign	97.25
NSG281	RS4;11-CDX vehicle	chr17:7579472	TP53	NM_000546.6	c.215C>G	p.Pro72Arg	rs1042522	germline/somatic benign	97.7
NSG284	RS4;11-CDX vehicle	chr17:7579472	TP53	NM_000546.6	c.215C>G	p.Pro72Arg	rs1042522	germline/somatic benign	97.55
NSG288	RS4;11-CDX vehicle	chr17:7579472	TP53	NM_000546.6	c.215C>G	p.Pro72Arg	rs1042522	germline/somatic benign	100
NSG277	RS4;11-CDX VEN	chr17:7579472	TP53	NM_000546.6	c.215C>G	p.Pro72Arg	rs1042522	germline/somatic benign	97.35
NSG279	RS4;11-CDX VEN	chr17:7579472	TP53	NM_000546.6	c.215C>G	p.Pro72Arg	rs1042522	germline/somatic benign	97.25
NSG282	RS4;11-CDX VEN	chr17:7579472	TP53	NM_000546.6	c.215C>G	p.Pro72Arg	rs1042522	germline/somatic benign	97.95
NSG283	RS4;11-CDX VEN	chr17:7579472	TP53	NM_000546.6	c.215C>G	p.Pro72Arg	rs1042522	germline/somatic benign	100
NSG287	RS4;11-CDX VEN	chr17:7579472	TP53	NM_000546.6	c.215C>G	p.Pro72Arg	rs1042522	germline/somatic benign	97.5
NSG278	RS4;11-CDX vehicle	chr17:7579633	TP53	NM_000546.6	c.96+41_97-54delACCTGGAGGGCTGGGG	p.?	NA	NA	96.27

NSG281	RS4;11-CDX vehicle	chr17:7579633	TP53	NM_000546.6	c.96+41_97-54delACCTGGAGGGCTGGGG	p.?	NA	NA	94.48
NSG284	RS4;11-CDX vehicle	chr17:7579633	TP53	NM_000546.6	c.96+41_97-54delACCTGGAGGGCTGGGG	p.?	NA	NA	95.68
NSG288	RS4;11-CDX vehicle	chr17:7579633	TP53	NM_000546.6	c.96+41_97-54delACCTGGAGGGCTGGGG	p.?	NA	NA	96.46
NSG277	RS4;11-CDX VEN	chr17:7579633	TP53	NM_000546.6	c.96+41_97-54delACCTGGAGGGCTGGGG	p.?	NA	NA	96.28
NSG279	RS4;11-CDX VEN	chr17:7579633	TP53	NM_000546.6	c.96+41_97-54delACCTGGAGGGCTGGGG	p.?	NA	NA	96.66
NSG282	RS4;11-CDX VEN	chr17:7579633	TP53	NM_000546.6	c.96+41_97-54delACCTGGAGGGCTGGGG	p.?	NA	NA	96.59
NSG283	RS4;11-CDX VEN	chr17:7579633	TP53	NM_000546.6	c.96+41_97-54delACCTGGAGGGCTGGGG	p.?	NA	NA	96.96
NSG287	RS4;11-CDX VEN	chr17:7579633	TP53	NM_000546.6	c.96+41_97-54delACCTGGAGGGCTGGGG	p.?	NA	NA	96.76
NSG278	RS4;11-CDX vehicle	chr17:7579801	TP53	NM_000546.6	c.74+38C>G	p.?	rs1642785	germline benign	98.5
NSG281	RS4;11-CDX vehicle	chr17:7579801	TP53	NM_000546.6	c.74+38C>G	p.?	rs1642785	germline benign	98.24
NSG284	RS4;11-CDX vehicle	chr17:7579801	TP53	NM_000546.6	c.74+38C>G	p.?	rs1642785	germline benign	98.29
NSG288	RS4;11-CDX vehicle	chr17:7579801	TP53	NM_000546.6	c.74+38C>G	p.?	rs1642785	germline benign	98.74
NSG277	RS4;11-CDX VEN	chr17:7579801	TP53	NM_000546.6	c.74+38C>G	p.?	rs1642785	germline benign	98.75
NSG279	RS4;11-CDX VEN	chr17:7579801	TP53	NM_000546.6	c.74+38C>G	p.?	rs1642785	germline benign	98.79
NSG282	RS4;11-CDX VEN	chr17:7579801	TP53	NM_000546.6	c.74+38C>G	p.?	rs1642785	germline benign	98.59
NSG283	RS4;11-CDX VEN	chr17:7579801	TP53	NM_000546.6	c.74+38C>G	p.?	rs1642785	germline benign	98.19
NSG287	RS4;11-CDX VEN	chr17:7579801	TP53	NM_000546.6	c.74+38C>G	p.?	rs1642785	germline benign	98.7
NSG279	RS4;11-CDX VEN	chr18:60985399	BCL2	NM_000633.3	c.501G>A	p.Ser167=	NA	NA	6.86
NSG287	RS4;11-CDX VEN	chr18:60985399	BCL2	NM_000633.3	c.501G>A	p.Ser167=	NA	NA	13.47
NSG279	RS4;11-CDX VEN	chr18:60985410	BCL2	NM_000633.3	c.490C>A	p.Arg164=	rs996326337	NA	5.65
NSG287	RS4;11-CDX VEN	chr18:60985410	BCL2	NM_000633.3	c.490C>A	p.Arg164=	rs996326337	NA	10.82
NSG279	RS4;11-CDX VEN	chr18:60985475	BCL2	NM_000633.3	c.420C>T	p.Asp140=	rs779104297	NA	10.28
NSG287	RS4;11-CDX VEN	chr18:60985475	BCL2	NM_000633.3	c.420C>T	p.Asp140=	rs779104297	NA	17.81
NSG279	RS4;11-CDX VEN	chr18:60985492	BCL2	NM_000633.3	c.408G>A	p.Glu136=	NA	NA	9.78
NSG287	RS4;11-CDX VEN	chr18:60985492	BCL2	NM_000633.3	c.408G>A	p.Glu136=	NA	NA	18.67
NSG279	RS4;11-CDX VEN	chr18:60985521	BCL2	NM_000633.3	c.379C>A	p.Arg127=	NA	NA	9.44
NSG287	RS4;11-CDX VEN	chr18:60985521	BCL2	NM_000633.3	c.379C>A	p.Arg127=	NA	NA	19.23
NSG279	RS4;11-CDX VEN	chr18:60985549	BCL2	NM_000633.3	c.351C>T	p.Ser117=	NA	NA	9
NSG287	RS4;11-CDX VEN	chr18:60985549	BCL2	NM_000633.3	c.351C>T	p.Ser117=	NA	NA	16.95
NSG278	RS4;11-CDX vehicle	chr18:60985600	BCL2	NM_000633.3	c.300C>T	p.Ala100=	rs61733416	NA	52.05
NSG281	RS4;11-CDX vehicle	chr18:60985600	BCL2	NM_000633.3	c.300C>T	p.Ala100=	rs61733416	NA	51.75
NSG284	RS4;11-CDX vehicle	chr18:60985600	BCL2	NM_000633.3	c.300C>T	p.Ala100=	rs61733416	NA	52.6
NSG288	RS4;11-CDX vehicle	chr18:60985600	BCL2	NM_000633.3	c.300C>T	p.Ala100=	rs61733416	NA	48.67
NSG277	RS4;11-CDX VEN	chr18:60985600	BCL2	NM_000633.3	c.300C>T	p.Ala100=	rs61733416	NA	52
NSG279	RS4;11-CDX VEN	chr18:60985600	BCL2	NM_000633.3	c.300C>T	p.Ala100=	rs61733416	NA	51.85
NSG282	RS4;11-CDX VEN	chr18:60985600	BCL2	NM_000633.3	c.300C>T	p.Ala100=	rs61733416	NA	51.5
NSG283	RS4;11-CDX VEN	chr18:60985600	BCL2	NM_000633.3	c.300C>T	p.Ala100=	rs61733416	NA	52.25
NSG287	RS4;11-CDX VEN	chr18:60985600	BCL2	NM_000633.3	c.300C>T	p.Ala100=	rs61733416	NA	56.15
NSG282	RS4;11-CDX VEN	chr18:60985775	BCL2	NM_000633.3	c.119_120delCG, c.124delG	p.Pro40ArgfsTer112, p.	rs1184260603	NA	52.71
NSG278	RS4;11-CDX vehicle	chr18:60985879	BCL2	NM_000633.3	c.21A>G	p.Thr7=	rs1801018	NA	51.66
NSG281	RS4;11-CDX vehicle	chr18:60985879	BCL2	NM_000633.3	c.21A>G	p.Thr7=	rs1801018	NA	48.62
NSG284	RS4;11-CDX vehicle	chr18:60985879	BCL2	NM_000633.3	c.21A>G	p.Thr7=	rs1801018	NA	48.95
NSG288	RS4;11-CDX vehicle	chr18:60985879	BCL2	NM_000633.3	c.21A>G	p.Thr7=	rs1801018	NA	50
NSG277	RS4;11-CDX VEN	chr18:60985879	BCL2	NM_000633.3	c.21A>G	p.Thr7=	rs1801018	NA	49
NSG279	RS4;11-CDX VEN	chr18:60985879	BCL2	NM_000633.3	c.21A>G	p.Thr7=	rs1801018	NA	50.63
NSG282	RS4;11-CDX VEN	chr18:60985879	BCL2	NM_000633.3	c.21A>G	p.Thr7=	rs1801018	NA	49.32
NSG283	RS4;11-CDX VEN	chr18:60985879	BCL2	NM_000633.3	c.21A>G	p.Thr7=	rs1801018	NA	50.23
NSG287	RS4;11-CDX VEN	chr18:60985879	BCL2	NM_000633.3	c.21A>G	p.Thr7=	rs1801018	NA	49.45
NSG278	RS4;11-CDX vehicle	chr19:49458262	BAX	NM_004324.4	c.34+43C>T	p.?	rs4645881	NA	99.7
NSG281	RS4;11-CDX vehicle	chr19:49458262	BAX	NM_004324.4	c.34+43C>T	p.?	rs4645881	NA	99.5
NSG284	RS4;11-CDX vehicle	chr19:49458262	BAX	NM_004324.4	c.34+43C>T	p.?	rs4645881	NA	99.8
NSG288	RS4;11-CDX vehicle	chr19:49458262	BAX	NM_004324.4	c.34+43C>T	p.?	rs4645881	NA	100
NSG277	RS4;11-CDX VEN	chr19:49458262	BAX	NM_004324.4	c.34+43C>T	p.?	rs4645881	NA	99.75
NSG279	RS4;11-CDX VEN	chr19:49458262	BAX	NM_004324.4	c.34+43C>T	p.?	rs4645881	NA	99.8
NSG282	RS4;11-CDX VEN	chr19:49458262	BAX	NM_004324.4	c.34+43C>T	p.?	rs4645881	NA	99.65
NSG283	RS4;11-CDX VEN	chr19:49458262	BAX	NM_004324.4	c.34+43C>T	p.?	rs4645881	NA	99.75
NSG287	RS4;11-CDX VEN	chr19:49458262	BAX	NM_004324.4	c.34+43C>T	p.?	rs4645881	NA	99.45
NSG281	RS4;11-CDX vehicle	chr19:49458970	BAX	NM_004324.4	c.122delA	p.Glu41GlyfsTer19	NA	NA	13.75
NSG278	RS4;11-CDX vehicle	chr19:49459104	BAX	NM_004324.4	c.233+14A>G	p.?	rs1805419	NA	100
NSG281	RS4;11-CDX vehicle	chr19:49459104	BAX	NM_004324.4	c.233+14A>G	p.?	rs1805419	NA	99.95
NSG284	RS4;11-CDX vehicle	chr19:49459104	BAX	NM_004324.4	c.233+14A>G	p.?	rs1805419	NA	99.9
NSG288	RS4;11-CDX vehicle	chr19:49459104	BAX	NM_004324.4	c.233+14A>G	p.?	rs1805419	NA	100
NSG277	RS4;11-CDX VEN	chr19:49459104	BAX	NM_004324.4	c.233+14A>G	p.?	rs1805419	NA	99.9
NSG279	RS4;11-CDX VEN	chr19:49459104	BAX	NM_004324.4	c.233+14A>G	p.?	rs1805419	NA	99.95
NSG282	RS4;11-CDX VEN	chr19:49459104	BAX	NM_004324.4	c.233+14A>G	p.?	rs1805419	NA	100
NSG283	RS4;11-CDX VEN	chr19:49459104	BAX	NM_004324.4	c.233+14A>G	p.?	rs1805419	NA	100
NSG287	RS4;11-CDX VEN	chr19:49459104	BAX	NM_004324.4	c.233+14A>G	p.?	rs1805419	NA	99.95
NSG278	RS4;11-CDX vehicle	chr19:49464971	BAX	NM_004324.4	c.*617G>A	p.?	rs704243	NA	99.9
NSG281	RS4;11-CDX vehicle	chr19:49464971	BAX	NM_004324.4	c.*617G>A	p.?	rs704243	NA	99.75
NSG284	RS4;11-CDX vehicle	chr19:49464971	BAX	NM_004324.4	c.*617G>A	p.?	rs704243	NA	99.6
NSG288	RS4;11-CDX vehicle	chr19:49464971	BAX	NM_004324.4	c.*617G>A	p.?	rs704243	NA	99.85
NSG277	RS4;11-CDX VEN	chr19:49464971	BAX	NM_004324.4	c.*617G>A	p.?	rs704243	NA	99.95
NSG279	RS4;11-CDX VEN	chr19:49464971	BAX	NM_004324.4	c.*617G>A	p.?	rs704243	NA	99.9
NSG282	RS4;11-CDX VEN	chr19:49464971	BAX	NM_004324.4	c.*617G>A	p.?	rs704243	NA	99.9
NSG283	RS4;11-CDX VEN	chr19:49464971	BAX	NM_004324.4	c.*617G>A	p.?	rs704243	NA	99.8
NSG287	RS4;11-CDX VEN	chr19:49464971	BAX	NM_004324.4	c.*617G>A	p.?	rs704243	NA	100
PDX184	0054-PDX vehicle	chr6:33541507	BAK1	NM_001188.4	c.*73T>C	p.?	rs511515	NA	99.65
PDX185	0054-PDX vehicle	chr6:33541507	BAK1	NM_001188.4	c.*73T>C	p.?	rs511515	NA	99.95
PDX187	0054-PDX VEN	chr6:33541507	BAK1	NM_001188.4	c.*73T>C	p.?	rs511515	NA	99.85
PDX184	0054-PDX vehicle	chr17:7576501	TP53	NM_000546.6	c.9993+352C>T	p.?	rs77697176	germline benign	51.35
PDX185	0054-PDX vehicle	chr17:7576501	TP53	NM_000546.6	c.9993+352C>T	p.?	rs77697176	germline benign	46.15
PDX187	0054-PDX VEN	chr17:7576501	TP53	NM_000546.6	c.9993+352C>T	p.?	rs77697176	germline benign	49.67
PDX184	0054-PDX vehicle	chr17:7579416	TP53	NM_000546.6	c.271T>C	p.Trp91Arg	rs2073465780	germline uncertain	2.32
PDX184	0054-PDX vehicle	chr17:7579472	TP53	NM_000546.6	c.215C>G	p.Pro72Arg	rs1042522	germline/somatic benign	98
PDX185	0054-PDX vehicle	chr17:7579472	TP53	NM_000546.6	c.215C>G	p.Pro72Arg	rs1042522	germline/somatic benign	97.7
PDX187	0054-PDX VEN	chr17:7579472	TP53	NM_000546.6	c.215C>G	p.Pro72Arg	rs1042522	germline/somatic benign	97.15
PDX184	0054-PDX vehicle	chr17:7579633	TP53	NM_000546.6	c.96+41_97-54delACCTGGAGGGCTGGGG	p.?	NA	NA	97.34
PDX185	0054-PDX vehicle	chr17:7579633	TP53	NM_000546.6	c.96+41_97-54delACCTGGAGGGCTGGGG	p.?	NA	NA	97.43
PDX187	0054-PDX VEN	chr17:7579633	TP53	NM_000546.6	c.96+41_97-54delACCTGGAGGGCTGGGG	p.?	NA	NA	97.02
PDX184	0054-PDX vehicle	chr17:7579801	TP53	NM_000546.6	c.74+38C>G	p.?	rs1642785	germline benign	98.19
PDX185	0054-PDX vehicle	chr17:7579801	TP53	NM_000546.6	c.74+38C>G	p.?	rs1642785	germline benign	98.44
PDX187	0054-PDX VEN	chr17:7579801	TP53	NM_000546.6	c.74+38C>G	p.?	rs1642785	germline benign	97.69
PDX184	0054-PDX vehicle	chr18:60985399	BCL2	NM_000633.3	c.501G>A	p.Ser167=	NA	NA	13.17
PDX185	0054-PDX vehicle	chr18:60985399	BCL2	NM_000633.3	c.501G>A	p.Ser167=	NA	NA	4.3
PDX184	0054-PDX vehicle	chr18:60985410	BCL2	NM_000633.3	c.490C>A	p.Arg164=	rs996326337	NA	11.99
PDX185	0054-PDX vehicle	chr18:60985410	BCL2	NM_000633.3	c.490C>A	p.Arg164=	rs996326337	NA	4.86
PDX184	0054-PDX vehicle	chr18:60985475	BCL2	NM_000633.3	c.420C>T	p.Asp140=	rs779104297	NA	26.59
PDX185	0054-PDX vehicle	chr18:60985475	BCL2	NM_000633.3	c.420C>T	p.Asp140=	rs779104297	NA	8.5
PDX187	0054-PDX VEN	chr18:60985475	BCL2	NM_000633.3	c.420C>T	p.Asp140=	rs779104297	NA	3.88
PDX184	0054-PDX vehicle	chr18:60985492	BCL2	NM_000633.3	c.408G>A	p.Glu136=	NA	NA	27.76
PDX185	0054-PDX vehicle	chr18:60985492	BCL2	NM_000633.3	c.408G>A	p.Glu136=	NA	NA	8.6
PDX187	0054-PDX VEN	chr18:60985492	BCL2	NM_000633.3	c.408G>A	p.Glu136=	NA	NA	4.66
PDX184	0054-PDX vehicle	chr18:60985521	BCL2	NM_000633.3	c.379C>A	p.Arg127=	NA	NA	28.38
PDX185	0054-PDX vehicle	chr18:60985521	BCL2	NM_000633.3	c.379C>A	p.Arg127=	NA	NA	9.77
PDX187	0054-PDX VEN	chr18:60985521	BCL2	NM_000633.3	c.379C>A	p.Arg127=	NA	NA	4.8
PDX184	0054-PDX vehicle	chr18:60985549	BCL2	NM_000633.3	c.351C>T	p.Ser117=	NA	NA	26.95
PDX185	0054-PDX vehicle	chr18:60985549	BCL2	NM_000633.3	c.351C>T	p.Ser117=	NA	NA	9.9
PDX187	0054-PDX VEN	chr18:60985549	BCL2	NM_000633.3	c.351C>T	p.Ser117=	NA	NA	5.08
PDX184	0054-PDX vehicle	chr18:60985561	BCL2	NM_000633.3	c.339C>A	p.Ala113=	NA	NA	30.93
PDX184	0054-PDX vehicle	chr18:60985570	BCL2	NM_000633.3	c.327_330delCCGCinsTCGT	p.[Arg109=;Arg110=]	NA	NA	29.03
PDX184	0054-PDX vehicle	chr18:60985594	BCL2	NM_000633.3	c.306C>T	p.Asp102=	NA	NA	6.91
PDX185	0054-PDX vehicle	chr18:60985594	BCL2	NM_000633.3	c.306C>T	p.Asp102=	NA	NA	4.55
PDX184	0054-PDX vehicle	chr18:60985879	BCL2	NM_000633.3	c.21A>G	p.Thr7=	rs1801018	NA	99.85
PDX185	0054-PDX vehicle	chr18:60985879	BCL2	NM_000633.3	c.21A>G	p.Thr7=	rs1801018	NA	99.75
PDX187	0054-PDX VEN	chr18:60985879	BCL2	NM_000633.3	c.21A>G	p.Thr7=	rs1801018	NA	99.6
PDX184	0054-PDX vehicle	chr19:49458262	BAX	NM_004324.4	c.34+43C>T	p.?	rs4645881	NA	99.9
PDX185	0054-PDX vehicle	chr19:49458262	BAX	NM_004324.4	c.34+43C>T	p.?	rs4645881	NA	99.4

PDX187	0054-PDX VEN	chr19:49458262	BAX	NM_004324.4	c.34+43C>T	p.?	rs4645881	NA	99.85
PDX184	0054-PDX vehicle	chr19:49459104	BAX	NM_004324.4	c.233+14A>G	p.?	rs1805419	NA	54.58
PDX185	0054-PDX vehicle	chr19:49459104	BAX	NM_004324.4	c.233+14A>G	p.?	rs1805419	NA	55.56
PDX187	0054-PDX VEN	chr19:49459104	BAX	NM_004324.4	c.233+14A>G	p.?	rs1805419	NA	55.11
PDX184	0054-PDX vehicle	chr19:49464971	BAX	NM_004324.4	c.*617G>A	p.?	rs704243	NA	99.84
PDX185	0054-PDX vehicle	chr19:49464971	BAX	NM_004324.4	c.*617G>A	p.?	rs704243	NA	99.71
PDX187	0054-PDX VEN	chr19:49464971	BAX	NM_004324.4	c.*617G>A	p.?	rs704243	NA	99.9
PDX190	0122-PDX vehicle	chr6:33541507	BAK1	NM_001188.4	c.*73T>C	p.?	rs511515	NA	48.82
PDX191	0122-PDX vehicle	chr6:33541507	BAK1	NM_001188.4	c.*73T>C	p.?	rs511515	NA	47.47
PDX192	0122-PDX vehicle	chr6:33541507	BAK1	NM_001188.4	c.*73T>C	p.?	rs511515	NA	49.4
PDX193	0122-PDX VEN	chr6:33541507	BAK1	NM_001188.4	c.*73T>C	p.?	rs511515	NA	48.55
PDX194	0122-PDX VEN	chr6:33541507	BAK1	NM_001188.4	c.*73T>C	p.?	rs511515	NA	52.88
PDX195	0122-PDX VEN	chr6:33541507	BAK1	NM_001188.4	c.*73T>C	p.?	rs511515	NA	47.52
PDX190	0122-PDX vehicle	chr17:7579472	TP53	NM_000546.6	c.215C>G	p.Pro72Arg	rs1042522	germline/somatic benign	98.05
PDX191	0122-PDX vehicle	chr17:7579472	TP53	NM_000546.6	c.215C>G	p.Pro72Arg	rs1042522	germline/somatic benign	97.55
PDX192	0122-PDX vehicle	chr17:7579472	TP53	NM_000546.6	c.215C>G	p.Pro72Arg	rs1042522	germline/somatic benign	100
PDX193	0122-PDX VEN	chr17:7579472	TP53	NM_000546.6	c.215C>G	p.Pro72Arg	rs1042522	germline/somatic benign	97.55
PDX194	0122-PDX VEN	chr17:7579472	TP53	NM_000546.6	c.215C>G	p.Pro72Arg	rs1042522	germline/somatic benign	100
PDX195	0122-PDX VEN	chr17:7579472	TP53	NM_000546.6	c.215C>G	p.Pro72Arg	rs1042522	germline/somatic benign	99.65
PDX190	0122-PDX vehicle	chr17:7579633	TP53	NM_000546.6	c.96+41_97-54delACCTGGAGGGCTGGGG	p.?	NA	NA	96.42
PDX191	0122-PDX vehicle	chr17:7579633	TP53	NM_000546.6	c.96+41_97-54delACCTGGAGGGCTGGGG	p.?	NA	NA	97.3
PDX192	0122-PDX vehicle	chr17:7579633	TP53	NM_000546.6	c.96+41_97-54delACCTGGAGGGCTGGGG	p.?	NA	NA	96.45
PDX193	0122-PDX VEN	chr17:7579633	TP53	NM_000546.6	c.96+41_97-54delACCTGGAGGGCTGGGG	p.?	NA	NA	96.55
PDX194	0122-PDX VEN	chr17:7579633	TP53	NM_000546.6	c.96+41_97-54delACCTGGAGGGCTGGGG	p.?	NA	NA	96.74
PDX195	0122-PDX VEN	chr17:7579633	TP53	NM_000546.6	c.96+41_97-54delACCTGGAGGGCTGGGG	p.?	NA	NA	97.58
PDX190	0122-PDX vehicle	chr17:7579801	TP53	NM_000546.6	c.74+38C>G	p.?	rs1642785	germline benign	98.39
PDX191	0122-PDX vehicle	chr17:7579801	TP53	NM_000546.6	c.74+38C>G	p.?	rs1642785	germline benign	98.29
PDX192	0122-PDX vehicle	chr17:7579801	TP53	NM_000546.6	c.74+38C>G	p.?	rs1642785	germline benign	98.19
PDX193	0122-PDX VEN	chr17:7579801	TP53	NM_000546.6	c.74+38C>G	p.?	rs1642785	germline benign	98.44
PDX194	0122-PDX VEN	chr17:7579801	TP53	NM_000546.6	c.74+38C>G	p.?	rs1642785	germline benign	98.54
PDX195	0122-PDX VEN	chr17:7579801	TP53	NM_000546.6	c.74+38C>G	p.?	rs1642785	germline benign	97.43
PDX190	0122-PDX vehicle	chr18:60985879	BCL2	NM_000633.3	c.21A>G	p.Thr7=	rs1801018	NA	51.55
PDX191	0122-PDX vehicle	chr18:60985879	BCL2	NM_000633.3	c.21A>G	p.Thr7=	rs1801018	NA	48.15
PDX192	0122-PDX vehicle	chr18:60985879	BCL2	NM_000633.3	c.21A>G	p.Thr7=	rs1801018	NA	50.83
PDX193	0122-PDX VEN	chr18:60985879	BCL2	NM_000633.3	c.21A>G	p.Thr7=	rs1801018	NA	48.4
PDX194	0122-PDX VEN	chr18:60985879	BCL2	NM_000633.3	c.21A>G	p.Thr7=	rs1801018	NA	51.9
PDX195	0122-PDX VEN	chr18:60985879	BCL2	NM_000633.3	c.21A>G	p.Thr7=	rs1801018	NA	51.15
PDX190	0122-PDX vehicle	chr19:49458262	BAX	NM_004324.4	c.34+43C>T	p.?	rs4645881	NA	99.85
PDX191	0122-PDX vehicle	chr19:49458262	BAX	NM_004324.4	c.34+43C>T	p.?	rs4645881	NA	99.7
PDX192	0122-PDX vehicle	chr19:49458262	BAX	NM_004324.4	c.34+43C>T	p.?	rs4645881	NA	99.95
PDX193	0122-PDX VEN	chr19:49458262	BAX	NM_004324.4	c.34+43C>T	p.?	rs4645881	NA	99.9
PDX194	0122-PDX VEN	chr19:49458262	BAX	NM_004324.4	c.34+43C>T	p.?	rs4645881	NA	99.95
PDX195	0122-PDX VEN	chr19:49458262	BAX	NM_004324.4	c.34+43C>T	p.?	rs4645881	NA	99.7
PDX190	0122-PDX vehicle	chr19:49459104	BAX	NM_004324.4	c.233+14A>G	p.?	rs1805419	NA	48.75
PDX191	0122-PDX vehicle	chr19:49459104	BAX	NM_004324.4	c.233+14A>G	p.?	rs1805419	NA	54.43
PDX192	0122-PDX vehicle	chr19:49459104	BAX	NM_004324.4	c.233+14A>G	p.?	rs1805419	NA	52.43
PDX193	0122-PDX VEN	chr19:49459104	BAX	NM_004324.4	c.233+14A>G	p.?	rs1805419	NA	52.61
PDX194	0122-PDX VEN	chr19:49459104	BAX	NM_004324.4	c.233+14A>G	p.?	rs1805419	NA	52.73
PDX195	0122-PDX VEN	chr19:49459104	BAX	NM_004324.4	c.233+14A>G	p.?	rs1805419	NA	52.96
PDX190	0122-PDX vehicle	chr19:49464971	BAX	NM_004324.4	c.*617G>A	p.?	rs704243	NA	99.7
PDX191	0122-PDX vehicle	chr19:49464971	BAX	NM_004324.4	c.*617G>A	p.?	rs704243	NA	99.65
PDX192	0122-PDX vehicle	chr19:49464971	BAX	NM_004324.4	c.*617G>A	p.?	rs704243	NA	100
PDX193	0122-PDX VEN	chr19:49464971	BAX	NM_004324.4	c.*617G>A	p.?	rs704243	NA	99.9
PDX194	0122-PDX VEN	chr19:49464971	BAX	NM_004324.4	c.*617G>A	p.?	rs704243	NA	99.65
PDX195	0122-PDX VEN	chr19:49464971	BAX	NM_004324.4	c.*617G>A	p.?	rs704243	NA	99.85
PDX223	0134-PDX vehicle	chr6:33541507	BAK1	NM_001188.4	c.*73T>C	p.?	rs511515	NA	33.2
PDX226	0134-PDX vehicle	chr6:33541507	BAK1	NM_001188.4	c.*73T>C	p.?	rs511515	NA	31.35
PDX227	0134-PDX vehicle	chr6:33541507	BAK1	NM_001188.4	c.*73T>C	p.?	rs511515	NA	32.47
PDX224	0134-PDX VEN	chr6:33541507	BAK1	NM_001188.4	c.*73T>C	p.?	rs511515	NA	31.75
PDX225	0134-PDX VEN	chr6:33541507	BAK1	NM_001188.4	c.*73T>C	p.?	rs511515	NA	34.1
PDX228	0134-PDX VEN	chr6:33541507	BAK1	NM_001188.4	c.*73T>C	p.?	rs511515	NA	34.65
PDX228	0134-PDX VEN	chr17:7577035	TP53	NM_000546.6	c.902delC	p.Pro301GlnfsTer44	rs876660726	germline pathogenic	4.63
PDX223	0134-PDX vehicle	chr17:7579470	TP53	NM_000546.6	c.215C>G	p.Pro72Arg	rs1042522	germline/somatic benign	85.06
PDX226	0134-PDX vehicle	chr17:7579470	TP53	NM_000546.6	c.215C>G	p.Pro72Arg	rs1042522	germline/somatic benign	85.05
PDX227	0134-PDX vehicle	chr17:7579470	TP53	NM_000546.6	c.215C>G	p.Pro72Arg	rs1042522	germline/somatic benign	88.83
PDX224	0134-PDX VEN	chr17:7579470	TP53	NM_000546.6	c.215C>G	p.Pro72Arg	rs1042522	germline/somatic benign	97.65
PDX225	0134-PDX VEN	chr17:7579470	TP53	NM_000546.6	c.215C>G	p.Pro72Arg	rs1042522	germline/somatic benign	97.65
PDX228	0134-PDX VEN	chr17:7579470	TP53	NM_000546.6	c.215C>G	p.Pro72Arg	rs1042522	germline/somatic benign	98.35
PDX223	0134-PDX vehicle	chr17:7579633	TP53	NM_000546.6	c.96+41_97-54delACCTGGAGGGCTGGGG	p.?	NA	NA	84.22
PDX226	0134-PDX vehicle	chr17:7579633	TP53	NM_000546.6	c.96+41_97-54delACCTGGAGGGCTGGGG	p.?	NA	NA	85.84
PDX227	0134-PDX vehicle	chr17:7579633	TP53	NM_000546.6	c.96+41_97-54delACCTGGAGGGCTGGGG	p.?	NA	NA	82.52
PDX224	0134-PDX VEN	chr17:7579633	TP53	NM_000546.6	c.96+41_97-54delACCTGGAGGGCTGGGG	p.?	NA	NA	96.28
PDX225	0134-PDX VEN	chr17:7579633	TP53	NM_000546.6	c.96+41_97-54delACCTGGAGGGCTGGGG	p.?	NA	NA	97.02
PDX228	0134-PDX VEN	chr17:7579633	TP53	NM_000546.6	c.96+41_97-54delACCTGGAGGGCTGGGG	p.?	NA	NA	97.14
PDX223	0134-PDX vehicle	chr17:7579801	TP53	NM_000546.6	c.74+38C>G	p.?	rs1642785	germline benign	81.93
PDX226	0134-PDX vehicle	chr17:7579801	TP53	NM_000546.6	c.74+38C>G	p.?	rs1642785	germline benign	83.69
PDX227	0134-PDX vehicle	chr17:7579801	TP53	NM_000546.6	c.74+38C>G	p.?	rs1642785	germline benign	83.01
PDX224	0134-PDX VEN	chr17:7579801	TP53	NM_000546.6	c.74+38C>G	p.?	rs1642785	germline benign	98.54
PDX225	0134-PDX VEN	chr17:7579801	TP53	NM_000546.6	c.74+38C>G	p.?	rs1642785	germline benign	98.59
PDX228	0134-PDX VEN	chr17:7579801	TP53	NM_000546.6	c.74+38C>G	p.?	rs1642785	germline benign	97.99
PDX225	0134-PDX VEN	chr18:60985775	BCL2	NM_000633.3	c.119_120delCG, c.124delG	p.Pro40ArgfsTer112, p.	rs1184260603	NA	65.14
PDX223	0134-PDX vehicle	chr19:49458262	BAX	NM_004324.4	c.34+43C>T	p.?	rs4645881	NA	37.37
PDX226	0134-PDX vehicle	chr19:49458262	BAX	NM_004324.4	c.34+43C>T	p.?	rs4645881	NA	40.74
PDX227	0134-PDX vehicle	chr19:49458262	BAX	NM_004324.4	c.34+43C>T	p.?	rs4645881	NA	36.59
PDX225	0134-PDX VEN	chr19:49458262	BAX	NM_004324.4	c.34+43C>T	p.?	rs4645881	NA	35.07
PDX228	0134-PDX VEN	chr19:49458262	BAX	NM_004324.4	c.34+43C>T	p.?	rs4645881	NA	33.55
PDX223	0134-PDX vehicle	chr19:49459104	BAX	NM_004324.4	c.233+14A>G	p.?	rs1805419	NA	56.21
PDX226	0134-PDX vehicle	chr19:49459104	BAX	NM_004324.4	c.233+14A>G	p.?	rs1805419	NA	55.56
PDX227	0134-PDX vehicle	chr19:49459104	BAX	NM_004324.4	c.233+14A>G	p.?	rs1805419	NA	54.5
PDX224	0134-PDX VEN	chr19:49459104	BAX	NM_004324.4	c.233+14A>G	p.?	rs1805419	NA	53.6
PDX225	0134-PDX VEN	chr19:49459104	BAX	NM_004324.4	c.233+14A>G	p.?	rs1805419	NA	53.68
PDX228	0134-PDX VEN	chr19:49459104	BAX	NM_004324.4	c.233+14A>G	p.?	rs1805419	NA	54.58
PDX223	0134-PDX vehicle	chr19:49464971	BAX	NM_004324.4	c.*617G>A	p.?	rs704243	NA	52.94
PDX226	0134-PDX vehicle	chr19:49464971	BAX	NM_004324.4	c.*617G>A	p.?	rs704243	NA	58.41
PDX227	0134-PDX vehicle	chr19:49464971	BAX	NM_004324.4	c.*617G>A	p.?	rs704243	NA	56.08
PDX224	0134-PDX VEN	chr19:49464971	BAX	NM_004324.4	c.*617G>A	p.?	rs704243	NA	58.03
PDX225	0134-PDX VEN	chr19:49464971	BAX	NM_004324.4	c.*617G>A	p.?	rs704243	NA	53.26
PDX228	0134-PDX VEN	chr19:49464971	BAX	NM_004324.4	c.*617G>A	p.?	rs704243	NA	52.63
PDX229	0152-PDX vehicle	chr6:33541507	BAK1	NM_001188.4	c.*73T>C	p.?	rs511515	NA	46.7
PDX230	0152-PDX vehicle	chr6:33541507	BAK1	NM_001188.4	c.*73T>C	p.?	rs511515	NA	47.4
PDX233	0152-PDX vehicle	chr6:33541507	BAK1	NM_001188.4	c.*73T>C	p.?	rs511515	NA	49.8
PDX232	0152-PDX VEN	chr6:33541507	BAK1	NM_001188.4	c.*73T>C	p.?	rs511515	NA	48.17
PDX232	0152-PDX VEN	chr17:7574018	TP53	NM_000546.6	c.1009C>T	p.Arg337Cys	rs587782529	germline pathogenic	48.75
PDX229	0152-PDX vehicle	chr17:7577121	TP53	NM_000546.6	c.817C>A	p.Arg273Ser	rs121913343	germline pathogenic	52.45
PDX230	0152-PDX vehicle	chr17:7577121	TP53	NM_000546.6	c.817C>A	p.Arg273Ser	rs121913343	germline pathogenic	49.75
PDX233	0152-PDX vehicle	chr17:7577121	TP53	NM_000546.6	c.817C>A	p.Arg273Ser	rs121913343	germline pathogenic	54.1
PDX232	0152-PDX VEN	chr17:7577121	TP53	NM_000546.6	c.817C>A	p.Arg273Ser	rs121913343	germline pathogenic	53.58
PDX229	0152-PDX vehicle	chr17:7579472	TP53	NM_000546.6	c.215C>G	p.Pro72Arg	rs1042522	germline/somatic benign	97.55
PDX230	0152-PDX vehicle	chr17:7579472	TP53	NM_000546.6	c.215C>G	p.Pro72Arg	rs1042522	germline/somatic benign	97.35
PDX233	0152-PDX vehicle	chr17:7579472	TP53	NM_000546.6	c.215C>G	p.Pro72Arg	rs1042522	germline/somatic benign	97.9
PDX232	0152-PDX VEN	chr17:7579472	TP53	NM_000546.6	c.215C>G	p.Pro72Arg	rs1042522	germline/somatic benign	97.19
PDX229	0152-PDX vehicle	chr17:7579633	TP53	NM_000546.6	c.96+41_97-54delACCTGGAGGGCTGGGG	p.?	NA	NA	95.1
PDX230	0152-PDX vehicle	chr17:7579633	TP53	NM_000546.6	c.96+41_97-54delACCTGGAGGGCTGGGG	p.?	NA	NA	97.02
PDX233	0152-PDX vehicle	chr17:7579633	TP53	NM_000546.6	c.96+41_97-54delACCTGGAGGGCTGGGG	p.?	NA	NA	97.55
PDX232	0152-PDX VEN	chr17:7579633	TP53	NM_000546.6	c.96+41_97-54delACCTGGAGGGCTGGGG	p.?	NA	NA	95.99
PDX229	0152-PDX vehicle	chr17:7579801	TP53	NM_000546.6	c.74+38C>G	p.?	rs1642785	germline benign	97.89
PDX230	0152-PDX vehicle	chr17:7579801	TP53	NM_000546.6	c.74+38C>G	p.?	rs1642785	germline benign	98.75

PDX233	0152-PDX vehicle	chr17:7579801	TP53	NM_000546.6	c.74+38C>G	p.?	rs1642785	germline benign	97.94
PDX232	0152-PDX VEN	chr17:7579801	TP53	NM_000546.6	c.74+38C>G	p.?	rs1642785	germline benign	98.59
PDX229	0152-PDX vehicle	chr18:60985475	BCL2	NM_000633.3	c.420C>T	p.Asp140=	rs779104297	NA	4.84
PDX233	0152-PDX vehicle	chr18:60985475	BCL2	NM_000633.3	c.420C>T	p.Asp140=	rs779104297	NA	3.74
PDX232	0152-PDX VEN	chr18:60985475	BCL2	NM_000633.3	c.420C>T	p.Asp140=	rs779104297	NA	2.86
PDX229	0152-PDX vehicle	chr18:60985492	BCL2	NM_000633.3	c.408G>A	p.Glu136=	NA	NA	5.3
PDX233	0152-PDX vehicle	chr18:60985492	BCL2	NM_000633.3	c.408G>A	p.Glu136=	NA	NA	3.9
PDX232	0152-PDX VEN	chr18:60985492	BCL2	NM_000633.3	c.408G>A	p.Glu136=	NA	NA	2.85
PDX229	0152-PDX vehicle	chr18:60985521	BCL2	NM_000633.3	c.379C>A	p.Arg127=	NA	NA	4.96
PDX233	0152-PDX vehicle	chr18:60985521	BCL2	NM_000633.3	c.379C>A	p.Arg127=	NA	NA	3.69
PDX232	0152-PDX VEN	chr18:60985521	BCL2	NM_000633.3	c.379C>A	p.Arg127=	NA	NA	2.41
PDX229	0152-PDX vehicle	chr18:60985549	BCL2	NM_000633.3	c.351C>T	p.Ser117=	NA	NA	5.33
PDX230	0152-PDX vehicle	chr18:60985549	BCL2	NM_000633.3	c.351C>T	p.Ser117=	NA	NA	2.45
PDX233	0152-PDX vehicle	chr18:60985549	BCL2	NM_000633.3	c.351C>T	p.Ser117=	NA	NA	3.75
PDX232	0152-PDX VEN	chr18:60985549	BCL2	NM_000633.3	c.351C>T	p.Ser117=	NA	NA	2.85
PDX229	0152-PDX vehicle	chr18:60985561	BCL2	NM_000633.3	c.339C>A	p.Ala113=	NA	NA	4.43
PDX233	0152-PDX vehicle	chr18:60985561	BCL2	NM_000633.3	c.339C>A	p.Ala113=	NA	NA	4.24
PDX229	0152-PDX vehicle	chr18:60985570	BCL2	NM_000633.3	c.327_330delCCGCinsTCGT	p.[Arg109=;Arg110=]	NA	NA	5.12
PDX233	0152-PDX vehicle	chr18:60985570	BCL2	NM_000633.3	c.327_330delCCGCinsTCGT	p.[Arg109=;Arg110=]	NA	NA	3.76
PDX229	0152-PDX vehicle	chr18:60985879	BCL2	NM_000633.3	c.21A>G	p.Thr7=	rs1801018	NA	99.7
PDX230	0152-PDX vehicle	chr18:60985879	BCL2	NM_000633.3	c.21A>G	p.Thr7=	rs1801018	NA	99.75
PDX233	0152-PDX vehicle	chr18:60985879	BCL2	NM_000633.3	c.21A>G	p.Thr7=	rs1801018	NA	99.8
PDX232	0152-PDX VEN	chr18:60985879	BCL2	NM_000633.3	c.21A>G	p.Thr7=	rs1801018	NA	99.8
PDX229	0152-PDX vehicle	chr19:49458262	BAX	NM_004324.4	c.34+43C>T	p.?	rs4645881	NA	37.42
PDX230	0152-PDX vehicle	chr19:49458262	BAX	NM_004324.4	c.34+43C>T	p.?	rs4645881	NA	31.85
PDX233	0152-PDX vehicle	chr19:49458262	BAX	NM_004324.4	c.34+43C>T	p.?	rs4645881	NA	31.32
PDX232	0152-PDX VEN	chr19:49458262	BAX	NM_004324.4	c.34+43C>T	p.?	rs4645881	NA	34.47
PDX229	0152-PDX vehicle	chr19:49459104	BAX	NM_004324.4	c.233+14A>G	p.?	rs1805419	NA	56.18
PDX230	0152-PDX vehicle	chr19:49459104	BAX	NM_004324.4	c.233+14A>G	p.?	rs1805419	NA	55.56
PDX233	0152-PDX vehicle	chr19:49459104	BAX	NM_004324.4	c.233+14A>G	p.?	rs1805419	NA	52.96
PDX232	0152-PDX VEN	chr19:49459104	BAX	NM_004324.4	c.233+14A>G	p.?	rs1805419	NA	53.31
PDX217	0159-PDX vehicle	chr6:33541507	BAK1	NM_001188.4	c.*73T>C	p.?	rs511515	NA	99.65
PDX219	0159-PDX vehicle	chr6:33541507	BAK1	NM_001188.4	c.*73T>C	p.?	rs511515	NA	99.6
PDX222	0159-PDX vehicle	chr6:33541507	BAK1	NM_001188.4	c.*73T>C	p.?	rs511515	NA	99.95
PDX221	0159-PDX VEN	chr6:33541507	BAK1	NM_001188.4	c.*73T>C	p.?	rs511515	NA	99.9
PDX217	0159-PDX vehicle	chr6:33545340	BAK1	NM_001188.4	c.42C>T	p.Cys14=	rs2227925	NA	99.3
PDX219	0159-PDX vehicle	chr6:33545340	BAK1	NM_001188.4	c.42C>T	p.Cys14=	rs2227925	NA	99.55
PDX222	0159-PDX vehicle	chr6:33545340	BAK1	NM_001188.4	c.42C>T	p.Cys14=	rs2227925	NA	99.4
PDX221	0159-PDX VEN	chr6:33545340	BAK1	NM_001188.4	c.42C>T	p.Cys14=	rs2227925	NA	99.3
PDX217	0159-PDX vehicle	chr17:7579472	TP53	NM_000546.6	c.215C>G	p.Pro72Arg	rs1042522	germline/somatic benign	46.26
PDX219	0159-PDX vehicle	chr17:7579472	TP53	NM_000546.6	c.215C>G	p.Pro72Arg	rs1042522	germline/somatic benign	48.25
PDX222	0159-PDX vehicle	chr17:7579472	TP53	NM_000546.6	c.215C>G	p.Pro72Arg	rs1042522	germline/somatic benign	45.73
PDX221	0159-PDX VEN	chr17:7579472	TP53	NM_000546.6	c.215C>G	p.Pro72Arg	rs1042522	germline/somatic benign	48.7
PDX217	0159-PDX vehicle	chr17:7579633	TP53	NM_000546.6	c.96+41_97-54delACCTGGAGGGCTGGGG	p.?	NA	NA	53.63
PDX219	0159-PDX vehicle	chr17:7579633	TP53	NM_000546.6	c.96+41_97-54delACCTGGAGGGCTGGGG	p.?	NA	NA	53.73
PDX222	0159-PDX vehicle	chr17:7579633	TP53	NM_000546.6	c.96+41_97-54delACCTGGAGGGCTGGGG	p.?	NA	NA	51.98
PDX221	0159-PDX VEN	chr17:7579633	TP53	NM_000546.6	c.96+41_97-54delACCTGGAGGGCTGGGG	p.?	NA	NA	61.4
PDX217	0159-PDX vehicle	chr17:7579801	TP53	NM_000546.6	c.74+38C>G	p.?	rs1642785	germline benign	51.91
PDX219	0159-PDX vehicle	chr17:7579801	TP53	NM_000546.6	c.74+38C>G	p.?	rs1642785	germline benign	54.28
PDX222	0159-PDX vehicle	chr17:7579801	TP53	NM_000546.6	c.74+38C>G	p.?	rs1642785	germline benign	51.18
PDX221	0159-PDX VEN	chr17:7579801	TP53	NM_000546.6	c.74+38C>G	p.?	rs1642785	germline benign	57.19
PDX217	0159-PDX vehicle	chr19:49458262	BAX	NM_004324.4	c.34+43C>T	p.?	rs4645881	NA	99.7
PDX219	0159-PDX vehicle	chr19:49458262	BAX	NM_004324.4	c.34+43C>T	p.?	rs4645881	NA	99.9
PDX222	0159-PDX vehicle	chr19:49458262	BAX	NM_004324.4	c.34+43C>T	p.?	rs4645881	NA	99.55
PDX221	0159-PDX VEN	chr19:49458262	BAX	NM_004324.4	c.34+43C>T	p.?	rs4645881	NA	100
PDX217	0159-PDX vehicle	chr19:49459104	BAX	NM_004324.4	c.233+14A>G	p.?	rs1805419	NA	99.9
PDX219	0159-PDX vehicle	chr19:49459104	BAX	NM_004324.4	c.233+14A>G	p.?	rs1805419	NA	100
PDX222	0159-PDX vehicle	chr19:49459104	BAX	NM_004324.4	c.233+14A>G	p.?	rs1805419	NA	99.95
PDX221	0159-PDX VEN	chr19:49459104	BAX	NM_004324.4	c.233+14A>G	p.?	rs1805419	NA	99.85
PDX217	0159-PDX vehicle	chr19:49464971	BAX	NM_004324.4	c.*617G>A	p.?	rs704243	NA	99.55
PDX219	0159-PDX vehicle	chr19:49464971	BAX	NM_004324.4	c.*617G>A	p.?	rs704243	NA	99.9
PDX222	0159-PDX vehicle	chr19:49464971	BAX	NM_004324.4	c.*617G>A	p.?	rs704243	NA	99.9
PDX221	0159-PDX VEN	chr19:49464971	BAX	NM_004324.4	c.*617G>A	p.?	rs704243	NA	99.9

Table S3: Fold changes of gene expression in cell lines, CDX and PDX models in controls vs VEN-resistant samples.

Gene	SEM cell lin	RS4;11 cell	REH cell lin	NALM-6 ce	SEM-CDX	RS4;11-CD)	0122-PDX	0134-PDX	0159-PDX
ACIN1	1	1.06	-1.22	-1.03	1.49	1.18	1.36	1.06	-1.08
AFF3	-1.25	-1.15	1.82	1.73	-1.86	-1.52	6.89	-1.59	1.66
AIFM1	1.11	-1.21	-1.13	-1.39	1.65	-1.32	-1.6	1.37	1.33
AKT1	-1.08	1.13	1.07	-1.01	-1.07	1.79	-1.62	1	-1.72
AKT2	-1.29	-1.14	-1.13	-1.05	1.03	-2.21	1	-1.28	1.77
AKT3	1.15	-1.23	1.54	1.21	-1.55	1.83	-1.15	1.82	1.83
ANKHD1	-1.08	-1.1	1.03	1.25	-1.67	-1.12	1.12	-1.4	-1.74
APAF1	1.13	-1.41	-1.09	-1.15	1.14	2.52	1.04	-1.12	1.01
ARID1A	1.16	-1.12	-1.17	1.17	1.15	-1.65	-1.15	-1.43	-1.11
ASXL1	-1.2	-1.07	1	1.11	1.61	7.95	-1.01	8.41	1.16
ATF1	1.24	-1.07	1.16	1.01	1.23	-1.33	18.45	1.06	1.58
ATF2	-1.07	1.1	1.16	-1.31	-1.17	1.37	-1.32	-1.58	-1.21
ATM	1.94	-1.29	1.29	2.51	1.37	1.26	23.84	-1.39	-1.68
ATRX	1.47	-1.4	1.18	2.38	-3.16	-1.74	2.35	-1.95	-2.21
AVEN	-1.04	-1.01	1.04	-1.1	1.18	-1.46	5.07	1.55	1.05
BACH2	-1.77	-4.23	-1.07	1.1	-3.58	-1.85	-1.2	-1.14	-1.23
BAD	-1.16	1.21	1.05	-1.46	-1.14	-1.31	1.25	1.17	-1.91
BAK1	-1.18	1.12	-1.23	-1.12	1.15	-1.45	1.02	2.44	1.05
BAX	-1.41	1.31	-1.55	-1.62	-1.03	1.49	-1.27	1.78	-1.01
BBC3	1	2.85	-1.8	-1.24	1.17	3.41	-1.25	2.4	-2.3
BCL11A	1.47	1.13	-1.01	1.47	1.1	1.44	2.16	-1.66	-2.11
BCL2	1.23	1.05	1.09	-1.65	1.74	1.88	1.94	1.59	1.51
BCL2A1	-1.03	1.33	1.32	2.07	-1.6	-1.15	4.7	1.27	-2.56
BCL2L1	-1.14	1.04	-1.07	-1.03	-1.47	1.35	-1.29	-1	-1.31
BCL2L10	-2.2	-1	-1.25	-1	-2.01	2.02	-1	-1	-1.97
BCL2L11	-1.1	-2.17	-1.01	1.95	1.74	1.14	3.54	-1.21	2.17
BCL2L13	-1.06	1.46	-1.08	-1.37	1.55	2.64	-1.38	1.42	1.21
BCL2L2	1.33	1.33	-1.45	-1.08	1.64	2.69	1.47	-1.09	-3.14
BCL6	-1.16	1.79	-1.4	-4.4	-2.82	-1.73	1.29	-1.41	-1.21
BID	1.22	-1.32	-1.18	1.02	1.42	-1.95	1.3	1.29	1.65
BIK	-2.65	-1.48	-2.23	-2.62	-1.97	-37.52	-6.42	-1.35	1.34
BIRC2	-1.13	-1.46	1.44	1.65	-1.27	-1.59	1.32	-1.23	1.61
BIRC3	-1.08	-1.45	-2.32	2.19	-3.95	1.42	2.86	-2.04	-2.83
BIRC5	-1.23	-1.31	-1.12	-1.02	1.35	-2	1.3	-1.18	1.64
BIRC6	-1.01	-1.06	1.08	1.36	1.21	1.08	1.72	-1.51	1.03
BIRC7	-1.31	1.11	-1.26	-2.49	2.33	1.6	-1	-1	-1
BMF	1.26	1.72	1.38	1.02	-4.2	-1.36	-1.11	-1.19	1.05
BRAF	-1.11	1.1	1.04	-1.05	-1.59	1.78	-1.28	1.06	-1.13
BTG1	1.18	1.15	1.15	1.12	-1.31	-1.1	1.24	-1.45	1.33
CASP1	1.56	-1.62	1.03	-1.63	1.7	-1.49	11.02	-1.06	1.17
CASP10	-1.02	1.27	-1.2	1.28	1.11	-1.34	2.75	-2.24	1.26
CASP14	-8.47	-1	-1.08	-1	-4.74	1.18	-4.55	-1	1.38
CASP2	-1.04	1.04	-1.08	-1.13	-1	-1.64	-1.16	1.04	1.8
CASP3	1.06	-1.64	1.09	1.14	-1.21	-2.18	-2.31	-1	1.6
CASP4	1.38	-1.27	1.23	1.18	1.09	-1.57	3.03	1.42	1.5
CASP5	1.02	-1.7	-1.09	-1	-1.75	-1.41	1.45	-3.98	-1.95
CASP6	1.02	-1.13	1.2	1.19	1.86	-1.85	1.9	1.23	2.99
CASP7	1.21	-1.3	-1.08	2.01	-1.04	-1.03	1.98	-2.62	-1.07
CASP8	1.16	-1.53	1.08	1.4	-1.88	1.36	1.52	-1.51	-1.89
CASP9	-1.03	-1.2	-1.01	-1.12	-1.4	-1.52	1.3	-1.38	-1.19
CCND1	4.77	2.16	-1.68	-1	-2.34	-1.94	-5.63	2.97	-5.63

CDKN1A	1.36	2.26	-2.02	-1.08	-1.33	1.2	-1.47	-1.1	-1.34
CDKN1B	1.38	1.06	1.04	1.31	1.4	2	8.88	-2.64	1.19
CDKN2A	-1.17	-1.42	1.41	-1.37	1.67	-1	4.67	2.28	2.88
CDKN2B	2.34	-1	-1	-1	-1.9	-1	-1	-1	-1.18
CDX2	-1.29	2.48	-1.68	-1	-1.78	3.96	4.56	-1.41	-2.09
CELSR3	1.35	1.44	-1.29	-1.57	-2.06	1.25	2.16	1.95	-3.15
CFLAR	1.6	-1.25	1.13	1.94	1.79	-1.07	11.9	-1.6	1.71
CREB1	1.11	-1.35	1.06	1.36	-1.09	-1.02	2.58	-1.15	1.77
CREBBP	1.07	-1.11	-1.01	1.47	-1.75	-1.13	2.47	-1.78	-1.41
CRLF2	1.91	-2.42	-1	-2.16	1.65	-1.2	-2.89	-2.38	1.01
CSNK2A1	1.49	-1.4	1.31	1.95	1.73	-1.54	14.25	-2.17	-1.48
DAP	1.08	-1.08	-1.02	-1.11	1.3	-1.1	-1.2	1	2.29
DAPK1	-1	-2.26	1.57	1.54	-2.05	1.78	-3.3	1.31	-4.86
DAPK3	-1.24	-1.04	-1.16	-1.08	-1.57	1.39	-2.38	1.38	-1.63
DAXX	-1.1	-1.08	-1.06	-1.09	1.29	-1.59	-1.13	1.25	1.21
DDIT4	-1.33	2.08	-1.11	-1.65	1.14	2.18	-2.43	1.31	-1.43
DEPTOR	-1.36	-1.15	-1.13	-1.43	-1.74	-1.91	-2.92	2.03	1.06
DIABLO	-1.25	1.06	-1.1	-1.09	1.29	-1.98	-1.11	1.1	1.55
DNMT3A	1.28	-1.16	-1.15	1.1	1.34	-2.34	1.25	-1.67	-1.01
DOT1L	1.07	1.04	-1.12	1.1	-1.1	1.78	1.73	1.57	-1.61
EIF4EBP1	1.02	-1.31	1.39	-1.61	1.53	1.01	-1.09	1.62	1.06
EP300	2.18	1.8	-1.37	1.66	1.66	-1.33	21.98	-2.68	-1.98
ERG	-1.5	1.56	1.02	-1.17	-1.07	1.78	-1.38	-1.22	-1.25
ESR1	-1.11	-2.09	1.26	1.15	-2.39	-2.13	7.01	-2.66	-2.13
ETV6	1.95	1.8	1.14	-1	1.28	-1.11	3.66	-2.08	1.27
EZH2	1.02	-1.32	1.16	2.49	-1.18	-2.75	9.27	-4.09	-1.51
FADD	-1.19	-1.06	-1.27	1.24	1.04	-1.17	1.13	-1.11	-2.42
FAF1	-1.1	-1.03	1.06	-1.05	-1.26	-1.32	1.39	1.26	-1.51
FAIM	-1.1	1.16	1.14	-1.29	1.19	1.13	1.75	1.37	1.2
FAS	1.12	1.64	-1.88	1.65	-2.2	1.32	-1.16	-1.03	1.46
FASLG	1.91	1.58	-1	3.27	-1	-2.31	-1	-1	-1
FHIT	1.71	-1	1.17	-1	1.13	-1.39	-1	-1	-1
FLCN	-1.15	-1.17	1.02	-2	-1.43	1.53	-2.73	1.45	-1.25
FLT1	1.16	-51.91	-1.05	-1.88	-1	3.15	-1.09	-1	1.01
FLT3	1.23	-1.17	1.1	-2.54	1.14	1.62	4.24	-2.07	1.15
FOS	-1.05	3.5	-1.27	1.06	2.64	2.24	17.32	-2.22	-1.6
FOXO1	-2.25	-1.98	-1.05	1.74	-1.46	-1.88	1.17	-1.79	-1.47
FOXO3	1	1.39	1.04	1.26	-1.3	1.61	1.49	-1.35	1.1
FOXO4	-1.14	1.7	-1.21	-1.59	-1.29	3.42	-1.69	1.37	1.33
GAB2	-1.12	1.51	1.03	-1.3	-1.44	-1.03	-1.63	1.1	-1.24
GSK3A	1	1.15	1.09	-1.01	1.79	-1.13	1.25	3.49	1.75
GSK3B	-1.07	-1.22	1.02	-1.05	-1.03	1.62	-2.24	1.47	1.88
GZMA	2.56	1.78	-1.12	-2.31	3.64	-1.93	1.24	1.13	12.13
GZMB	-1	1.58	-1	-1	-1	-1.56	-1	-1	1.38
HDAC9	-1.23	1.52	1.28	1.63	-1.77	-2.71	2.65	-1.43	1.81
HOXA3	1.11	1.08	-1.02	1.12	1.22	-1.44	31.3	-6.3	-1
HOXA5	1.28	1.42	1.06	-1	1.04	1.48	23.48	-25.41	-1
HOXA9	1.17	1.04	-1.08	-1	-1.39	1.33	50.14	-1.32	-1
HRK	-1	-37.25	-1.55	-1.31	-1	1.09	-1	-1	-1
HTRA2	1.05	-1.17	1.09	-1.01	-1	-1.21	-1.03	1.95	1.01
IKZF1	-1.4	1.18	1.04	1.04	-1.26	2.37	-1.49	3.74	-1.17
IL10	-1	1.52	-1	-1	-1.04	1.53	1.62	1.28	-2.69
IL7	1.25	1.1	-1	-1	2.66	1.43	2.79	-3.42	-1
IL7R	-2.4	-1.49	-1.12	-1.19	-3.54	-1.79	3.46	-1.86	1.45

JAK1	1.05	-1.46	1.13	1.78	-1.53	-2.93	2.39	-2.7	1.15
JAK2	1.24	-1.28	1.34	1.46	-1.05	1.14	2.89	-2.22	1.44
JUN	-1.35	1.12	-1.23	-2.08	1.24	2.06	2.5	1.02	-1.88
KDM6A	2.27	-1.46	1.29	6.94	1.73	-2.65	9.11	-6.14	1.77
KRAS	-1.29	1.01	1.12	-1.2	1.12	-1.13	2.12	1.16	2.51
LYN	-1.14	1.12	-1.02	1.03	-2.15	-1.38	-1.05	-1.2	1.2
MAP2K1	1.07	-1.5	1.05	1.03	-1.42	-1.06	-1.59	-1.31	1.16
MAP2K2	-1.07	1.31	-1.04	-1.98	1.21	-2.1	-2.3	1.82	1.08
MAP2K3	1.17	-1.17	-1.08	1.15	-1.48	-4.78	-1.56	-1.58	1.19
MAP2K4	-1.19	-1.09	1.44	1.26	1.09	1.94	4.68	2.76	3.28
MAP2K6	-1.05	1.16	-1.08	-1.59	1.22	-1	1.12	1.03	-1.05
MAP2K7	-1.36	1.08	1.05	-1.1	-1.44	-1.31	-2.42	-1.23	-1.01
MAP3K11	-1.49	-1.22	-1.29	-1.12	-1.07	-1.07	-1.31	2	-2.03
MAP3K12	1.32	1.39	-1.38	-1.2	-1.22	1.45	-1.35	1.11	-1.34
MAPK1	-1.06	-1.38	1.01	1.06	-1.21	1.02	-1.22	1.07	1.51
MAPK10	2.32	-4.37	1.09	-1	-1.62	1	-1	-1	-1
MAPK14	1.08	-1.24	1.09	1.93	1.32	-1.05	4.22	-2.6	1.1
MAPK3	1.03	1.12	1.09	-1.23	-1.15	-2.16	-1.31	1.61	1.13
MAPK9	-1.09	-1.58	1.26	1.27	1.06	1.04	1.35	-1.2	2.04
MAPKAPK2	-1.17	-1.21	-1.13	-1.43	-1.1	-1.42	-1.17	-1.35	1.69
MAX	-1.07	1.29	1.03	-1.38	1.07	1.13	1.4	-1.66	1.19
MCL1	1.2	-1.16	-1.06	1.87	1	1.53	1.31	-2.43	1.13
MDM2	-1.33	1.04	1.06	-1.37	-1.15	1.1	-1.03	1.08	-1.01
MDM4	1.08	1.17	-1.01	-1.41	1.03	2.88	1.79	-1.34	-1.8
MEIS1	1.08	1.46	1.41	-5.08	-2.28	1.88	-1.77	-1.63	-2.67
MELK	-1.05	-1.73	1.22	1.2	1.47	-1.61	1.82	-1.14	1.58
MKNK2	1.16	1.29	-1.05	-1.18	-1.13	1.45	-2.28	1.68	-1.36
MLL2	1.28	1.02	-1.3	-1.16	1.65	1.3	1.55	1.85	-1.41
MTOR	-1.11	1.05	-1.15	1.6	-2.16	-1.07	-1.11	-1.49	-1.69
MXD1	1.96	-1.14	-1.17	1.45	1.19	1.62	2.93	-1.48	-1.32
NF1	1.08	-1.32	1.22	1.5	1.42	1.17	1.65	-1.58	1.48
NFKB1	-1.1	1.06	-1.1	1.29	-1.14	-1.24	-1.63	1	2.13
NOTCH1	-1.17	1.61	1	-1.38	-1.51	1.67	-2.8	-1.08	-2.05
NOTCH2	-1.05	1.52	-1.07	1.28	-1.8	1.64	-2.44	-1.37	-1.15
NOTCH3	1.91	-3.84	-1.15	1.1	1.48	4.77	-5.53	-1	-2.48
NOTCH4	-1	-1	-1	-1	-1	-1	-1	-1	-1
NRAS	-1.14	-1.22	1.06	1.46	1.03	-1.31	-1.13	-1.49	2.41
PAWR	1.34	-1	-1.16	-1	-1.02	1.59	-2.3	-1	-1
PAX5	1.07	-1.07	-1.02	1.08	1.07	1.95	1.1	1.01	-1.77
PDCD4	1.13	1.04	1.16	-1.14	-1.65	-1.38	-2.05	1.01	-1.42
PDCD6IP	1.04	-1.57	1.16	1.86	1.22	-1.45	1.97	-1.35	1.78
PDK1	1.6	1.69	1.31	-1.18	1.02	1.61	2.73	-1.54	-1.83
PEA15	1.23	1.07	1.06	1.03	1.38	1	-1.22	-1.51	1.29
PHLDA3	-1	1.85	-4.74	-1.88	-1.4	-4.86	-1.56	-1.28	-1.82
PIDD	-1.02	1.02	-1.2	-1.43	1.17	-1.92	-1.25	1.37	-3.07
PIK3CA	-1.23	-1.31	1.03	1.78	1.35	-1.58	2.03	-1.8	1.9
PIK3R1	1.02	-1.79	1.26	1.76	-1.39	-1.51	1.5	-1.48	1.34
PMAIP1	1.14	1.2	1.02	-1.15	1	-1.13	1.76	-1.28	-1.21
POLR2A	-1.06	1.21	-1.04	-1.21	1.1	-1.02	-2.59	1.92	1.19
POLR2B	-1.18	-1.04	1.07	1.21	1.11	-1.28	1.25	-1.68	1.85
PRKCD	-1.14	1.15	-1.43	-1.66	-1.64	1.7	-2.8	1.1	-3.34
PTEN	1.24	-1.16	1.11	1.4	1.2	-1.4	13.25	-2.54	1.56
PTPN11	1.27	-1.48	1.09	1.29	1.09	-1.21	1.74	-2.17	1.3
PTPRS	-1	-325.55	-1	-1.02	1.58	1.09	-14.92	-1	1.38

PTRH2	1.41	-1.05	-1.25	-1.75	-1.03	-3.7	-1.31	1.1	-1.41
RAG1	-51.52	-2.7	1.13	1.16	1.31	-1.82	-1.23	-1.22	1.25
RB1	1.15	-1.35	1.09	1.46	1.28	-1.46	2	-2.13	3.05
RGCC	-2.11	-1.05	1.4	1.32	-1.15	-1.14	-1.42	-1.48	1.16
RHEB	1.05	-1.04	1.15	1.54	1.28	-2.12	8.31	-1.48	1.28
RICTOR	1.06	1.07	1.04	-1.13	-1.21	1.52	-1.04	-1.86	-1.49
ROS1	-1	-2.26	-1	-1	-1	4.78	-1	-1	-1
RPS6KA1	-1.01	-1.02	-1.11	1.39	-1.31	-1.16	-2.47	1.1	-1.27
RPS6KA3	1.05	-1.22	1.04	1.33	1.41	-1.04	-1.03	-1.07	1.63
RPS6KB1	-1.2	-1.36	1.3	2.05	1.7	-2.02	4.18	-3.36	1.23
RPS6KB2	1.27	-1.42	-1.01	1.57	1.68	-2.02	1.32	1.09	2.68
RUNX1	1.23	1.24	-1.01	-1.13	1.58	-1.51	1.64	-2.34	-1.37
SETD2	-1.01	1.22	1.03	1.05	1.43	1.06	-1.44	-1.07	1.73
SH3RF1	1.75	-2.39	-1.02	1.23	1.21	-1.01	7.23	-1.4	1.25
SIVA1	1	1.39	-1.12	-2.29	2	1.25	-2.35	6.99	-2.25
STAT3	1.22	-1.2	-1.2	-1.13	-1.37	1.2	1.16	-1.25	1.43
STAT5A	-1.06	-1.53	-1.14	-3.9	1.2	1.28	-1.03	1.07	1.59
STAT5B	-1.06	1.12	-1.14	1.06	1.15	1.73	1.01	-1.13	-1.06
STK17B	1.58	1.25	1.13	1.68	1.04	1.14	10.29	-2.38	1.59
STK3	1.23	1.08	1.1	1.21	1.53	3.46	2.49	2.1	-2.02
STK4	-1.09	-1.04	1.07	1.06	-1.09	-1.45	2.69	1.01	-1.32
TET2	1.18	-1.04	1.04	-1.18	1.47	1.57	2.14	1.2	1.01
THEM4	2.24	-1	1.25	-1	-1.07	1.28	7.16	1.11	-1.81
TNF	-1.55	2	-1.41	-1.35	1.42	1.54	1.76	-1.1	-1.14
TNFRSF10A	-1.62	2.16	-2	2.39	1.44	-1.47	-2.23	-1	-1.88
TNFRSF10C	-2.07	2.17	-1.33	-1.37	1.38	-1.34	4.42	-1.12	-1.37
TNFRSF13B	-1.15	1.84	-2.39	-2.19	-1.07	-2.72	-1.36	-1	-1.93
TNFRSF13C	1.6	1.92	-1.16	1.05	-3.19	5.55	-1.93	-2.11	-4.11
TNFRSF1A	1.4	-1	1.13	2.48	3.29	-2.47	2.43	-1.61	1.84
TNFRSF6B	-1.06	1.63	-1.15	-1.39	1.31	2.58	-1.17	2.16	-3.45
TNFSF10	-1.02	-1.68	1.06	-1	-3.12	-1.06	-1.33	-1.72	1.12
TNFSF13	1.51	1.84	-1.06	2.52	-1.95	-1.29	-1.11	-2.21	-1.96
TNFSF13B	2.1	1.3	-1.44	1.52	1.7	-1.63	11	-1.7	1.5
TP53	1.11	-1.14	-1.15	-1.19	-1.17	-2.07	-1.13	-1.26	1.58
TRADD	1.17	1.21	-1.14	-1.16	-1.09	1.86	-1.3	2.7	-2.65
TRAF3	-1.16	-1.34	-1.1	-1.21	1.01	1.85	-1.86	1.61	-3.09
TRAF6	1.17	-1.32	1.08	2.79	1.42	-1.56	7.84	-2.9	3.44
TRIM24	-1.03	1.02	1.14	1.9	1.27	-1.46	2.69	-1.96	2.16
TSC1	-1.14	-1.13	-1.03	-1.03	1.07	-1.39	1.84	-1.92	-1.26
TSC2	-1.25	1.09	-1.03	1.24	-1.2	-1.31	-1.51	1.16	1.21
TWIST1	-1.17	1.01	-1	-1	-1.06	-1.46	-3.24	-1.3	-1
VDAC1	-1	-1.09	1.09	-1.6	1.08	-1.77	1.37	-1.09	1.45
VDAC2	-1.2	-1.25	1.02	1.21	1.74	-3.19	1.78	1.26	2.99
WEE1	-1.17	-1.55	1.26	1.58	1.97	-1.87	10.56	-3.65	1.21
WWOX	1.17	-1.29	1.11	1.15	1.18	1.19	8.12	1.04	-1.31
XAF1	1.36	1.44	1.12	-1.16	1.33	-2.08	25.06	-2.41	1.25
XIAP	1.13	-1.2	1.02	1.07	-1.06	1.39	1.5	-1.47	-1.15
YAP1	-1.05	-3.11	-1	-1	3.66	-3.21	-1	-1	-1
YWHAB	1.1	-1.24	1.03	1.59	1.33	-1.27	5.07	-1.63	1.49

Table S4: Genes upregulated in cluster 2

FeatureID	FeatureName	Cluster 2 Average	Cluster 2 Log2 Fold Change	Cluster 2 P-Value
feature_27387	PRSS12	1.260335725	2.467948297	5.75E-22
feature_35284	XIST	5.923666849	2.315417043	1.14E-19
feature_23141	MCTP1	1.196296083	2.202220047	1.01E-16
feature_2987	CD109	1.279013953	1.80189785	9.14E-11
feature_35659	ZDHHC14	4.000254002	1.485164757	4.43E-07
feature_27618	PTPN14	2.391702726	1.313139048	2.32E-05
feature_31899	SNTB1	1.871380638	1.270481874	5.92E-05
feature_19130	HMGA2	3.090357428	1.179296792	0.000318286
feature_30678	SEL1L3	1.347945512	1.170831033	0.000416722
feature_35702	ZFHX3	2.36991146	1.11608509	0.000970386
feature_696	ALOX5AP	1.865154562	1.113791291	0.000975796
feature_26622	PLD1	3.965565863	1.039859821	0.003065427
feature_1594	ATXN1	4.392052087	1.022027234	0.004142514
feature_25204	NRIP1	3.202426801	1.008755535	0.004975913
feature_25820	PAM	1.085560869	0.959655127	0.010957755
feature_7477	ENSG00000234147	6.467559083	0.948705591	0.010962129
feature_1357	ASPH	1.159829065	0.948033851	0.012428233
feature_22743	LST1	1.921189248	0.933438536	0.013827963
feature_34855	UST	1.28435059	0.925663756	0.016559456
feature_14759	ENSG00000287092	24.41733696	0.903266512	0.019245226
feature_19706	IGF2BP2	4.374708018	0.902445861	0.019876213
feature_33214	THSD7A	2.487317469	0.8950376	0.022428864
feature_18694	GYPC	1.438668338	0.893819137	0.022762013
feature_17516	FMNL2	7.718555694	0.888071914	0.023182704
feature_27671	PUDP	3.276694996	0.888947072	0.02344698
feature_33479	TMEM181	2.554470149	0.872302036	0.028996759
feature_31971	SORBS2	2.461523725	0.875921533	0.029185187
feature_22836	MACROD1	1.06732736	0.873042735	0.031822607
feature_34134	TRIO	1.076221755	0.842798817	0.043834857
feature_26711	PLXDC2	2.282301672	0.824551859	0.05102713
feature_5279	DTNA	2.657645127	0.814027522	0.056156454
feature_14020	ENSG00000285756	1.131811722	0.785969066	0.074345046
feature_31274	SLC25A6	8.893505229	0.772728934	0.079485793
feature_24757	NEGR1	30.14221409	0.771379678	0.080552628
feature_17910	GAS7	7.867536804	0.764255293	0.086794621
feature_17463	FKBP5	3.177522495	0.755441824	0.095956926
feature_1888	BLNK	2.645192975	0.75038303	0.100721431
feature_1145	ARHGEF3	2.0350375	0.7465495	0.106818893
feature_23808	MPO	1.256333247	0.750655488	0.119150617
feature_27685	PUS7	1.091342226	0.732691599	0.12534254
feature_31746	SNHG3	2.292085506	0.715425465	0.139189239
feature_548	AIF1	5.471386881	0.709744308	0.143582863
feature_34722	UNG	1.343498315	0.712926495	0.144607661
feature_2858	CCDC85B	2.384587211	0.706112904	0.146868507
feature_32928	TBXAS1	1.953653789	0.708625468	0.146868507
feature_32584	SUMF1	2.817744231	0.698860396	0.157690113
feature_20246	JPX	3.422563069	0.695439132	0.158721622

feature_25637	OSBPL3	1.200298561	0.6997897	0.161375319
feature_27028	PPM1H	2.791061047	0.680783419	0.180515272
feature_27691	PVT1	3.484823831	0.676100707	0.186532317
feature_27515	PSMG4	1.274122036	0.639849621	0.247378137
feature_25736	P2RY8	1.561410984	0.638215347	0.249768618
feature_18369	GPM6B	3.179301374	0.635120808	0.250208931
feature_31754	SNHG8	2.374358657	0.624776345	0.265962466
feature_20315	KCNAB2	1.010403234	0.621438286	0.279791277
feature_32804	TARBP1	3.057448167	0.613579643	0.2862324
feature_32671	SYNGR1	1.823350907	0.604572836	0.305632816
feature_26602	PLCB1	27.66201214	0.593222758	0.322148867
feature_34214	TSEN2	1.473801197	0.595377063	0.325832532
feature_30511	SCARB1	1.049093851	0.59596907	0.33093609
feature_25792	PAG1	1.506265737	0.588788334	0.339517213
feature_22404	LINC03000	20.36594019	0.582257107	0.34517361
feature_3082	CD99	3.508838697	0.58227862	0.345816764
feature_2274	C1QBP	2.690109668	0.581551493	0.348017941
feature_20963	LGALS1	12.0839246	0.576119702	0.358779782
feature_30132	RPS4X	61.05201445	0.572309161	0.360485489
feature_358	ADAT2	1.103349659	0.5764011	0.362442966
feature_32237	SRM	2.610949555	0.568379354	0.376208581
feature_30929	SH2B3	1.311478494	0.571332466	0.376938125
feature_17445	FIRRE	2.434840541	0.563435497	0.389984828
feature_34447	TXN	1.931862522	0.562609338	0.390783363
feature_1492	ATP5MC1	3.104588459	0.561082011	0.391575448
feature_27537	PTCH1	1.55118243	0.558817668	0.402015868
feature_5681	EIF4EBP1	1.423103147	0.55578963	0.408702656
feature_28344	RHOH	2.874223637	0.548833885	0.422017328
feature_529	AHCY	1.276790355	0.546644813	0.428534499
feature_27640	PTPRE	1.146487473	0.543692441	0.428534499
feature_23400	MFSD1	1.007734916	0.539420443	0.432655965
feature_4568	DANCR	1.562300424	0.533636489	0.43924473
feature_19775	IGLC3	4.47165692	0.528208663	0.462239482
feature_24732	NEAT1	4.474769958	0.516820277	0.474742618
feature_33863	TPI1	3.148170993	0.514897901	0.479519676
feature_5595	EIF1AX	4.255967849	0.51090144	0.48833628
feature_5186	DPH6	1.358174066	0.512516182	0.49842375
feature_26452	PIK3C3	5.52297437	0.506703323	0.498845922
feature_24969	NME4	1.206969357	0.507395377	0.505947769
feature_24686	NDUFAF8	1.582312812	0.501032478	0.518907788
feature_31125	SLC12A2-DT	1.295468584	0.501433297	0.520146473
feature_17197	FARSB	1.041533616	0.500885552	0.52204665
feature_34187	TRPM2	1.88561167	0.489665589	0.5429652
feature_24922	NKG7	1.930973083	0.486020979	0.550164391
feature_32503	STS	1.915852612	0.486867173	0.551813962
feature_24175	MTHFD1L	5.264592204	0.481349014	0.561675195
feature_20650	KLHL5	1.01840819	0.478935084	0.577465686
feature_23445	MGST2	1.446228573	0.473667655	0.580403861
feature_33406	TMEM117	1.423103147	0.475330674	0.580403861
feature_901	ANXA2	2.061275964	0.470922726	0.581286242

feature_25268	NT5DC1	1.028636743	0.473130269	0.585150359
feature_23560	MIR3667HG	1.267006521	0.472060464	0.588737992
feature_18916	HDAC4	1.661917644	0.468342202	0.59512134
feature_26068	PDCD5	1.816235391	0.465641899	0.599404675
feature_1617	AUTS2	19.59746449	0.461679634	0.604590077
feature_31053	SIL1	1.190070007	0.466420552	0.604590077
feature_28823	RNF149	1.296358023	0.464442167	0.606271716
feature_17808	GADD45GIP1	1.477358954	0.463247997	0.606718053
feature_30535	SCFD2	4.165245023	0.461136757	0.609020307
feature_31377	SLC38A4-AS1	1.555184908	0.465454093	0.609020307
feature_24962	NME1	3.139721318	0.45305568	0.62561457
feature_32780	TAGLN2	4.293324307	0.451446814	0.628434687
feature_31562	SMAD3	1.369736779	0.454441446	0.629930795
feature_2482	CACNB4	6.8735882	0.449030592	0.634842234
feature_176	ACER3	1.169612899	0.451599355	0.635893391
feature_5064	DNAJC10	1.028636743	0.449729836	0.640493008
feature_30391	S100A16	1.56763706	0.448212998	0.640493008
feature_18081	GINS2	1.460904324	0.448114283	0.640612751
feature_32882	TBC1D8	1.231428942	0.449693417	0.640699458
feature_1730	BAX	1.95231963	0.446728623	0.642194468
feature_23126	MCM6	2.579819174	0.442182375	0.653797271
feature_24174	MTHFD1	1.257667406	0.440833305	0.661469899
feature_27405	PRSS57	2.037261099	0.439621233	0.661469899
feature_4678	DDB2	1.286574189	0.439272689	0.666508175
feature_23991	MRTO4	1.493813585	0.436255731	0.673025421
feature_32879	TBC1D4	1.48491919	0.435812638	0.679692005
feature_4296	CSTF3	1.256777967	0.434380149	0.681091007
feature_23124	MCM4	2.367687861	0.432474724	0.681091007
feature_3759	CLNS1A	2.829751664	0.430575259	0.684718453
feature_5629	EIF2S3	4.127888566	0.42791546	0.690222945
feature_23120	MCM2	1.519607329	0.426341016	0.699532615
feature_23937	MRPS12	1.037975858	0.427052516	0.700825484
feature_28383	RIMKLB	1.135369479	0.425849394	0.701634069
feature_3189	CDK4	1.7690951	0.420825334	0.707280562
feature_22498	LMO2	1.881164472	0.419951058	0.707280562
feature_22514	LNCAROD	15.84936658	0.419308571	0.707280562
feature_24311	MYC	1.983005291	0.419331508	0.709523829
feature_30049	RPS27L	1.819793149	0.418464478	0.712203584
feature_18247	GNL3	1.815345952	0.418087461	0.712469691
feature_787	ANKMY1	2.153777669	0.412407863	0.727988718
feature_5823	ENO1	8.040088061	0.4094835	0.730721741
feature_32037	SPART	1.20163272	0.411388947	0.734249681
feature_30485	SBF2	2.066612601	0.410150838	0.73603845
feature_4871	DHRSX	1.661917644	0.407702884	0.742688014
feature_33803	TOMM40	1.2349867	0.406897196	0.744301325
feature_3963	COLGALT1	1.331935602	0.404359025	0.751259929
feature_27677	PUM3	2.413049274	0.402127911	0.752272585
feature_23961	MRPS26	1.271453718	0.402602487	0.753269455
feature_24959	NMD3	1.094455264	0.403779907	0.75374658
feature_32304	SSBP4	1.916742051	0.398129107	0.764996889

feature_4257	CSMD1	1.331046162	0.403646213	0.767219765
feature_26506	PITPNC1	4.25418897	0.393055339	0.771561527
feature_25635	OSBPL1A	2.004351839	0.392045115	0.775965304
feature_4664	DCTPP1	1.639681657	0.39123035	0.778211431
feature_567	AK2	1.712615693	0.389182241	0.784622675
feature_30823	SESN1	2.406823197	0.386846196	0.790403957
feature_23470	MICOS13	1.006400757	0.386974358	0.796713187
feature_25796	PAICS	2.206254598	0.383614866	0.800660816
feature_31595	SMC1A	3.341624077	0.381683467	0.806158184
feature_23121	MCM3	4.096758184	0.380738709	0.80743831
feature_959	APBA1	1.589873047	0.381787582	0.813740646
feature_19516	HSPE1	4.060291166	0.377895537	0.814300111
feature_15928	ENSG00000289474	1.680595873	0.381115023	0.814805281
feature_27275	PRKX	1.528501724	0.377054327	0.816306949
feature_25196	NREP	1.453788809	0.375021911	0.820206654
feature_18345	GPATCH4	1.159829065	0.374816431	0.823209267
feature_19711	IGFBP2	2.516668972	0.371442088	0.823780544
feature_25013	NOLC1	1.509378775	0.372386292	0.823780544
feature_32104	SPECC1	1.347056073	0.372910537	0.824699703
feature_24440	NAA25	2.007464877	0.369889282	0.825767167
feature_26429	PIGL	1.619224549	0.369295214	0.826214929
feature_32894	TBCD	1.575642016	0.368475041	0.827313593
feature_24684	NDUFAF6	1.367068461	0.368074286	0.828851697
feature_33289	TKT	2.699448782	0.364057893	0.832161487
feature_1427	ATIC	1.424437306	0.363866675	0.835374398
feature_5379	E2F1	1.078445354	0.360548571	0.845731588
feature_3316	CENPX	1.014850432	0.359818736	0.848919403
feature_18469	GPX4	3.35274207	0.355858831	0.850990625
feature_19060	HIP1	3.306935938	0.356017152	0.851330085
feature_3210	CDKN1A	1.00150884	0.356442946	0.854827948
feature_3793	CMC1	1.437334178	0.354860439	0.855446815
feature_5134	DNTT	1.932307242	0.35434977	0.855488635
feature_24060	MT-RNR2	194.2495771	0.354735748	0.855488635
feature_17778	GABPB1-AS1	1.987897208	0.353543846	0.856732747
feature_23125	MCM5	2.98673773	0.351718227	0.859326741
feature_34010	TRAPPC2L	1.328822564	0.350268944	0.867140369
feature_32061	SPATA48	1.31814929	0.349971879	0.86805106
feature_23441	MGMT	1.717507611	0.348237995	0.871271672
feature_5805	EMP3	1.130922282	0.349454552	0.87470992
feature_32666	SYNE2	1.512491813	0.347441918	0.87470992
feature_23874	MRPL20	1.648131332	0.344925851	0.875680521
feature_35777	ZMAT3	1.35105855	0.34530315	0.875680521
feature_5258	DSE	1.408872116	0.341882178	0.877813838
feature_26847	POLR2H	1.004177158	0.343934633	0.877813838
feature_16735	ERI3	1.130477562	0.338696911	0.882565032
feature_21101	LINC00342	2.031924462	0.337418246	0.883395799
feature_17172	FANCA	1.555184908	0.335780915	0.887934419
feature_32753	TAF1D	3.615126713	0.332005347	0.893614062
feature_16913	FABP5	4.015374473	0.331680747	0.894510864
feature_5701	EIF5B	2.533123602	0.331607333	0.895905647

feature_35259	WWOX	8.384301135	0.331052493	0.896221329
feature_861	ANKS1A	2.770603939	0.328515067	0.901057719
feature_32797	TAOK3	5.334413203	0.327611397	0.901848115
feature_28090	RBM38	1.022855387	0.330078884	0.902958424
feature_25359	NUP210	1.25099661	0.328297352	0.907120235
feature_2054	BST2	2.05149213	0.324928603	0.91143422
feature_5695	EIF5A	1.96832954	0.3242791	0.91298677
feature_26815	POLE2	1.361731824	0.324913773	0.913754862
feature_4353	CTPS1	1.381299492	0.323925985	0.915297622
feature_27056	PPP1R14B	3.855275369	0.321295878	0.918428668
feature_23901	MRPL4	1.046870253	0.323310467	0.919704288
feature_1444	ATP11A	1.91852093	0.320467202	0.920242399
feature_21499	LINC01374	1.158939625	0.325564947	0.920242399
feature_22570	LRCH1	2.830641103	0.319344887	0.920878073
feature_2223	C19orf48	1.36751318	0.320086179	0.921503434
feature_1190	ARL2	1.058432966	0.320686036	0.92205632
feature_5325	DUT	9.656199572	0.31790739	0.92205632
feature_33263	TIMM8B	1.220310949	0.320161463	0.92205632
feature_2688	CBFA2T3	1.06777208	0.320912355	0.922482911
feature_29283	RPL12	34.45510607	0.317285215	0.922759317
feature_18042	GGCT	1.225202866	0.319410535	0.923625833
feature_81	ABCE1	1.376852295	0.318133001	0.925926878
feature_17898	GART	1.189180567	0.318412778	0.926248438
feature_30249	RREB1	1.98478417	0.316741772	0.928475332
feature_26318	PHB1	1.471132878	0.31432929	0.935769148
feature_19056	HINT1	6.765521305	0.311615098	0.938016949
feature_30755	SEPTIN6	4.918155532	0.311796986	0.938016949
feature_25336	NUDT5	2.231158903	0.312430488	0.938557919
feature_5616	EIF2B3	1.393751645	0.312098689	0.941558343
feature_20803	L3MBTL3	1.706834337	0.311844908	0.941558343
feature_24472	NAE1	1.412429873	0.311960965	0.941558343
feature_23487	MIF	1.13492476	0.311912437	0.942178664
feature_3187	CDK2AP1	1.054875208	0.310484995	0.942444515
feature_16648	EPB41L4A-AS1	1.194517204	0.308307972	0.944945749
feature_33346	TMA16	1.391972766	0.3076385	0.945350047
feature_1581	ATP8B4	4.891472348	0.306603968	0.946238616
feature_24059	MT-RNR1	74.10053615	0.306747602	0.946238616
feature_17542	FNDC3B	1.697939942	0.306450537	0.948927831
feature_160	ACAT1	1.002842999	0.306344293	0.95043408
feature_4386	CUL1	1.563189863	0.302627089	0.956174805
feature_29894	RPRD1A	1.871825358	0.300937728	0.957179497
feature_33250	TIMM13	2.030590303	0.300590716	0.957202347
feature_29972	RPS2	69.94062776	0.299063546	0.957409737
feature_26814	POLE	1.197185522	0.301886747	0.957419385
feature_33071	TEX14	3.696510424	0.300748912	0.958470406
feature_26959	PPFIBP1	1.061101284	0.301001979	0.959079365
feature_24618	NCOA7	1.187846408	0.299099416	0.960410071
feature_71	ABCC4	1.139816677	0.29930031	0.961276969
feature_5731	ELMO1	3.681389953	0.296995041	0.961276969
feature_4841	DGLUCY	1.570305379	0.296038753	0.96578589

feature_18300	GOLIM4	1.06732736	0.297291984	0.96578589
feature_23864	MRPL11	1.119804289	0.296252011	0.96578589
feature_4717	DDX21	5.198328964	0.29403636	0.965817628
feature_24693	NDUFB2	2.542907436	0.294131048	0.96589414
feature_26383	PHPT1	1.279013953	0.295655178	0.96589414
feature_19127	HMGA1	6.487126752	0.29323845	0.966456747
feature_33924	TRAF3	1.161607944	0.296439815	0.966456747
feature_34150	TRMT11	1.245215254	0.294292262	0.968237581
feature_2814	CCDC26	15.12269454	0.292109643	0.9687062
feature_18248	GNL3L	1.012626833	0.294157313	0.969136264
feature_17581	FOXK2	2.525563366	0.290555956	0.971278992
feature_25105	NPM1	15.96988563	0.290136129	0.971278992
feature_26722	PM20D2	1.324820086	0.29202322	0.971278992
feature_29629	RPL36	21.82728924	0.289208231	0.971345729
feature_24435	NAA10	1.304807698	0.290726381	0.971444378
feature_25750	PA2G4	4.730039085	0.289002857	0.971444378
feature_30394	S100A4	5.733771523	0.288296929	0.974549751
feature_25027	NOP56	3.411000355	0.286881717	0.978469511
feature_35068	VWA8	2.175568936	0.286462053	0.979616744
feature_23791	MPG	1.387080849	0.286359144	0.979865211
feature_2126	BZW2	2.56870118	0.284928287	0.980723654
feature_19503	HSPD1	6.888263951	0.283530017	0.981497746
feature_19585	IARS1	2.678546955	0.283130089	0.982187773
feature_20028	IPO5	1.322596487	0.283863571	0.983288548
feature_1325	ASCC3	3.597782644	0.281613623	0.983373056
feature_26848	POLR2I	1.21808735	0.282446423	0.983373056
feature_28790	RNASET2	2.384587211	0.280260172	0.983373056
feature_29879	RPLP1	115.8703924	0.2788507	0.983373056
feature_29967	RPS19	57.78421384	0.280241439	0.983373056
feature_34718	UNC93B1	1.022855387	0.283029759	0.983373056
feature_35124	WDFY4	2.192913006	0.281514209	0.983373056
feature_17188	FAR2	1.089118627	0.28109738	0.985331598
feature_29870	RPLP0	25.76972967	0.276744779	0.98688265
feature_5704	EIPR1	1.15182411	0.277857697	0.988534222
feature_26310	PHACTR1	2.40193128	0.276950574	0.988534222
feature_26732	PMM2	1.599212161	0.277441695	0.988534222
feature_3801	CMSS1	4.151458712	0.275528678	0.988598015
feature_29958	RPS17	3.758771187	0.275059726	0.989244211
feature_1026	APRT	2.658089847	0.274479308	0.990373277
feature_1503	ATP5MC3	4.252410091	0.273758349	0.990373277
feature_1695	BAG1	1.507155177	0.272503671	0.990373277
feature_2791	CCDC18-AS1	1.230539502	0.27314391	0.990373277
feature_3306	CENPP	7.832403945	0.265715408	0.990373277
feature_4183	CRIM1	1.793554685	0.273875406	0.990373277
feature_4937	DKC1	1.925191726	0.27119004	0.990373277
feature_4990	DMC1	1.081558392	0.265810207	0.990373277
feature_5501	EEF1E1	1.536506679	0.268078301	0.990373277
feature_16615	ENTPD1-AS1	1.572528977	0.273383043	0.990373277
feature_16881	EYA4	1.645463014	0.269213544	0.990373277
feature_18182	GMDS	5.673734359	0.268696039	0.990373277

feature_18485	GRB10	2.083511951	0.266083473	0.990373277
feature_19453	HSP90AB1	21.29718331	0.263728863	0.990373277
feature_19816	IKBIP	1.028192024	0.268704676	0.990373277
feature_20169	ITPA	1.186067529	0.273877955	0.990373277
feature_20458	KDM6A	3.112593414	0.265044035	0.990373277
feature_22804	LYRM4	2.199139082	0.271046302	0.990373277
feature_23885	MRPL3	1.369736779	0.275546986	0.990373277
feature_24605	NCL	10.9752383	0.267443925	0.990373277
feature_26737	PMS1	1.013960992	0.268936318	0.990373277
feature_26808	POLD2	1.338161678	0.274363656	0.990373277
feature_26935	PPA1	2.00257296	0.265132647	0.990373277
feature_26936	PPA2	1.857594327	0.267368118	0.990373277
feature_28854	RNF213	2.003017679	0.26974734	0.990373277
feature_29963	RPS18	135.15344	0.268148699	0.990373277
feature_32117	SPG7	1.55162715	0.27039228	0.990373277
feature_32233	SRI	1.078000634	0.267707657	0.990373277
feature_34557	UBE2G2	1.882943351	0.265668906	0.990373277
feature_34604	UBE3D	1.292800265	0.266872222	0.990373277
feature_30962	SH3RF1	1.347945512	0.264876705	0.990484281
feature_1223	ARMC2	1.012182113	0.265006589	0.990770536
feature_6724	ENSG00000227615	1.282126992	0.265358243	0.991620672
feature_4850	DHFR	3.049887932	0.262352533	0.992590487
feature_31186	SLC20A2	1.622337588	0.262532546	0.995083332
feature_4697	DDT	2.062610124	0.260691093	0.996865788
feature_24242	MTR	1.709502655	0.261161183	0.996865788
feature_29911	RPS12	122.8876251	0.259385108	0.996890076
feature_17937	GBE1	1.061546004	0.262521042	0.997029025
feature_22812	LYST	1.431552822	0.261628773	0.997377654
feature_669	ALG8	1.041978335	0.2608884	0.997578472
feature_4701	DDX10	2.144438555	0.260170066	0.997578472
feature_5408	EBNA1BP2	1.187846408	0.260868144	0.997578472
feature_5610	EIF2AK2	1.637458059	0.260026575	0.997578472
feature_16819	ETV6	7.065262405	0.259328584	0.997578472
feature_26844	POLR2E	1.314591532	0.260788093	0.997578472
feature_18594	GSTO1	1.339051117	0.259981612	0.99816925
feature_1087	ARHGAP18	1.026857865	0.260053268	0.999601503
feature_5688	EIF4G1	1.438223618	0.257324284	0.999601503
feature_18633	GTF3A	2.553580709	0.255471522	0.999601503
feature_19847	IL17RA	1.165165702	0.257088877	0.999601503
feature_26114	PDE8A	2.039039978	0.256520477	0.999601503
feature_27267	PRKDC	7.965819865	0.254101533	0.999601503
feature_28786	RNASEH2C	1.352392709	0.257317027	0.999601503
feature_61	ABCB7	1.093121105	0.000482709	1
feature_82	ABCF1	1.433776421	0.068874727	1
feature_109	ABHD17B	2.346786033	0.024112422	1
feature_111	ABHD18	1.192293605	0.102374134	1
feature_124	ABL1	1.512047094	0.114809942	1
feature_151	ACADM	1.471577598	0.116093685	1
feature_166	ACBD6	2.145772714	0.203949246	1
feature_217	ACSL4	1.156271307	0.18692011	1

feature_267	ACTN4	1.11713597	0.125808147	1
feature_293	ACYP2	1.378186454	0.060560434	1
feature_353	ADAR	1.438668338	0.106186291	1
feature_424	ADK	7.221803751	0.235941619	1
feature_437	ADPGK	1.023300107	0.148063036	1
feature_453	ADSS2	1.115801811	0.148674711	1
feature_463	AFF1	14.44938886	0.235405487	1
feature_466	AFF3	10.81691808	0.028448593	1
feature_471	AFG3L2	1.004621878	0.180858167	1
feature_509	AGO2	2.504216819	0.034610268	1
feature_527	AHCTF1	1.708613216	0.130486178	1
feature_531	AHCYL2	1.08422671	0.040219845	1
feature_534	AHI1	3.028541385	0.035891703	1
feature_555	AIMP1	1.90162158	0.198822215	1
feature_606	AKR7A2	1.250107171	0.201023869	1
feature_652	ALG13	1.074442876	0.150571255	1
feature_711	AMD1	2.728355565	0.162462608	1
feature_737	AMZ1	2.831085823	9.13E-05	1
feature_754	ANAPC5	2.259620966	0.011052669	1
feature_790	ANKRD10	2.134654721	0.06701596	1
feature_798	ANKRD13D	1.008624355	0.168498001	1
feature_800	ANKRD17	4.375597457	0.007563977	1
feature_814	ANKRD26	1.239433897	0.122640169	1
feature_883	ANP32A	3.684058272	0.039708446	1
feature_906	ANXA2R-AS1	1.114912372	0.000766813	1
feature_919	AOPEP	1.925636446	0.067174632	1
feature_941	AP2S1	1.553406029	0.165699173	1
feature_973	APEX1	3.33628744	0.194716051	1
feature_984	APLP2	1.062880163	0.233418608	1
feature_1023	APPL1	1.755753508	0.205123494	1
feature_1066	ARFGEF2	1.510268215	0.106805215	1
feature_1075	ARGLU1	4.573053019	0.031871359	1
feature_1095	ARHGAP25	3.146392114	0.179480664	1
feature_1111	ARHGAP4	2.13554416	0.11100187	1
feature_1124	ARHGDIA	1.504042138	0.182720681	1
feature_1135	ARHGEF18	4.495227066	0.067506689	1
feature_1158	ARHGEF6	1.656136287	0.009800267	1
feature_1217	ARL8B	1.395085804	0.06043712	1
feature_1250	ARPC1B	1.05042801	0.076294653	1
feature_1303	ASAP2	1.873604237	0.097473372	1
feature_1389	ATE1	1.295913303	0.02940495	1
feature_1404	ATF7IP2	1.973666177	0.025202615	1
feature_1405	ATG10	1.494703024	0.095043778	1
feature_1417	ATG3	1.024189546	0.083492992	1
feature_1424	ATG7	2.942710476	0.203592724	1
feature_1453	ATP13A3	1.260780444	0.055381112	1
feature_1464	ATP1B3	3.633804942	0.132430278	1
feature_1477	ATP2C1	1.014405712	0.100764917	1
feature_1481	ATP5F1A	3.227331106	0.058284218	1
feature_1484	ATP5F1B	4.464986124	0.075299088	1

feature_1486	ATP5F1C	2.959609826	0.076749988	1
feature_1488	ATP5F1D	2.713235094	0.169491487	1
feature_1489	ATP5F1E	8.603992683	0.161989433	1
feature_1491	ATP5IF1	1.742411916	0.227906089	1
feature_1499	ATP5MC2	9.249725736	0.051497077	1
feature_1504	ATP5ME	1.282126992	0.02855068	1
feature_1505	ATP5MF	2.461079005	0.089066361	1
feature_1518	ATP5MJ	2.889788828	0.050905879	1
feature_1519	ATP5MK	2.438398298	0.107116913	1
feature_1528	ATP5PD	1.460904324	0.078062818	1
feature_1531	ATP5PF	3.159733706	0.178279962	1
feature_1546	ATP6V0D1	1.188291128	0.131989308	1
feature_1549	ATP6V0E1	2.060386525	0.126326961	1
feature_1561	ATP6V1F	1.144708594	0.066829584	1
feature_1569	ATP6V1H	1.239433897	0.047337025	1
feature_1572	ATP8A1	1.958100986	0.182764959	1
feature_1588	ATR	2.513555933	0.13260313	1
feature_1591	ATRN	1.315925691	0.023383664	1
feature_1596	ATXN10	3.459919526	0.011258612	1
feature_1603	ATXN7L1	1.588983608	0.214473408	1
feature_1620	AVL9	1.146487473	0.132507267	1
feature_1653	B3GNT2	1.130922282	0.219338505	1
feature_1666	B4GALT1	1.166055141	0.060402484	1
feature_1684	BABAM2	2.411715114	0.019426356	1
feature_1702	BAHCC1	2.330331403	0.013089217	1
feature_1740	BBLN	1.747748552	0.079404407	1
feature_1766	BCAT1	3.474595277	0.000225951	1
feature_1859	BICD1	1.98611833	0.110409287	1
feature_1889	BLOC1S1	1.287908348	0.02683769	1
feature_1898	BLTP2	1.05176217	0.124239041	1
feature_1914	BMP2K	2.065278442	0.033912947	1
feature_1961	BOD1L1	1.931417802	0.148815198	1
feature_2001	BRCA1	3.926875246	0.095531581	1
feature_2002	BRCA2	2.384142491	0.003405799	1
feature_2028	BRIP1	3.484379112	0.040724202	1
feature_2030	BRK1	2.388589688	0.046233054	1
feature_2076	BTF3	11.78818597	0.163493972	1
feature_2096	BTK	1.012182113	0.129763454	1
feature_2186	C16orf74	4.669557201	0.146424759	1
feature_2224	C19orf53	1.536951399	0.086695505	1
feature_2264	C1orf43	1.560966264	0.03546293	1
feature_2359	C4orf3	1.259001565	0.023384031	1
feature_2478	CACNA2D4	1.272343157	0.113008484	1
feature_2540	CAMK2D	4.797636484	0.209999274	1
feature_2547	CAMKMT	2.489985788	0.195356574	1
feature_2562	CANX	2.55313599	0.097142468	1
feature_2584	CAPNS1	1.364844862	0.083314456	1
feature_2605	CARD8	1.340829996	0.065694508	1
feature_2624	CARS2	1.058877685	0.142873866	1
feature_2702	CBR4	1.208303516	0.250399167	1

feature_2709	CBX3	3.6711614	0.058872615	1
feature_2741	CCDC12	1.357729346	0.01021362	1
feature_2755	CCDC138	1.194517204	0.11989489	1
feature_2756	CCDC14	1.957656267	0.030370894	1
feature_2870	CCDC91	3.263798124	0.021840761	1
feature_2907	CCNB1IP1	1.806006838	0.112589055	1
feature_2941	CCNY	2.149330472	0.114597946	1
feature_2964	CCT3	2.878226115	0.077026395	1
feature_2967	CCT5	3.040993537	0.061206936	1
feature_2970	CCT6A	2.658534567	0.007083766	1
feature_2975	CCT7	2.290306627	0.129744617	1
feature_2978	CCT8	2.906243458	0.061992185	1
feature_3050	CD48	1.177173134	0.112939847	1
feature_3059	CD63	2.271183679	0.246134413	1
feature_3088	CDC123	1.914518452	0.076493161	1
feature_3126	CDC45	1.385746689	0.235928647	1
feature_3136	CDCA7	3.994472646	0.06033179	1
feature_3137	CDCA7L	1.404424918	0.190766488	1
feature_3176	CDK12	1.600546321	0.022261723	1
feature_3198	CDK6-AS1	15.69415939	0.119073705	1
feature_3235	CDV3	1.527612284	0.095846448	1
feature_3241	CDYL	2.560696225	0.009908122	1
feature_3296	CENPH	1.599212161	0.121208148	1
feature_3354	CEP83	1.519162609	0.11851437	1
feature_3357	CEP85L	1.572084258	0.253275574	1
feature_3375	CERS6	3.38654077	0.047482193	1
feature_3431	CFAP97	1.33460392	0.189430118	1
feature_3437	CFDP1	1.687711388	0.052246247	1
feature_3463	CHAF1A	2.031035023	0.068178559	1
feature_3470	CHCHD2	8.040088061	0.132128366	1
feature_3476	CHCHD3	3.137497719	0.043397662	1
feature_3493	CHD7	2.747033793	0.241784899	1
feature_3497	CHEK1	2.186686929	0.126803934	1
feature_3524	CHMP2A	1.008624355	0.001625089	1
feature_3576	CHST11	7.359666869	0.085152146	1
feature_3580	CHST15	1.617000951	0.151469041	1
feature_3597	CIAO1	1.125585645	0.111690058	1
feature_3603	CIB1	1.004621878	0.068426113	1
feature_3658	CLASP2	5.820491871	0.109064117	1
feature_3699	CLEC14A	1.025078986	0.060259595	1
feature_3772	CLSPN	1.60143576	0.132158433	1
feature_3776	CLTA	2.676323356	0.01889773	1
feature_3778	CLTC	1.742856635	0.065788775	1
feature_3796	CMIP	1.579199773	0.027587879	1
feature_3799	CMPK1	1.395975243	0.00794677	1
feature_3814	CNBP	4.453423411	0.2075135	1
feature_3865	CNST	1.05042801	0.040304551	1
feature_3869	CNTLN	2.420164789	0.019040515	1
feature_3889	COA1	1.5685265	0.130560232	1
feature_3891	COA4	1.276345635	0.228972463	1

feature_3968	COMMD10	3.15306291	0.176074611	1
feature_3974	COMMD6	3.647146534	0.095752455	1
feature_3979	COMT	3.362081185	0.094923489	1
feature_4003	COPS9	1.415098192	0.088664581	1
feature_4046	COX4I1	8.490144431	0.166851414	1
feature_4049	COX5A	4.013595594	0.248346638	1
feature_4052	COX5B	2.95338375	0.057387912	1
feature_4063	COX6C	4.494782346	0.103603423	1
feature_4071	COX7A2	4.861231406	0.114586751	1
feature_4075	COX7B	2.821301989	0.097956982	1
feature_4078	COX7C	5.827607387	0.089439652	1
feature_4104	CPLANE1	1.286129469	0.192834114	1
feature_4122	CPNE8	7.714108496	0.213608038	1
feature_4136	CPSF6	2.489541068	0.054397327	1
feature_4153	CRACR2A	1.377297014	0.090812514	1
feature_4155	CRADD	1.75175103	0.088916928	1
feature_4245	CSE1L	1.749082712	0.024682001	1
feature_4255	CSK	1.267006521	0.050962369	1
feature_4315	CTBP2	3.828147465	0.018045347	1
feature_4360	CTSC	2.030145583	0.126219891	1
feature_4390	CUL4A	1.15093467	0.1137063	1
feature_4395	CUTA	2.787058569	0.179167112	1
feature_4446	CYBA	11.00770284	0.182189919	1
feature_4450	CYC1	1.604548798	0.084315469	1
feature_4451	CYCS	2.125760326	0.055393762	1
feature_4545	CYTIP	1.290131947	0.162626374	1
feature_4563	DAD1	7.151538033	0.013570858	1
feature_4573	DAP	1.220755668	0.085026885	1
feature_4575	DAP3	1.383523091	0.090509041	1
feature_4583	DARS1	2.73636052	0.115865106	1
feature_4588	DAZAP1	1.313257373	0.020391397	1
feature_4597	DBI	2.885341631	0.029613543	1
feature_4604	DBNL	1.113133493	0.139637975	1
feature_4660	DCTN4	1.275456196	0.030285021	1
feature_4677	DDB1	1.037531138	0.130430385	1
feature_4710	DDX18	1.693048025	0.044150747	1
feature_4719	DDX24	2.095519384	0.029763185	1
feature_4729	DDX3X	2.491319947	0.068566997	1
feature_4733	DDX42	1.379965333	0.116071268	1
feature_4735	DDX46	3.035212181	0.037643242	1
feature_4739	DDX50	1.339495837	0.087006947	1
feature_4755	DECR1	1.225202866	0.025226599	1
feature_4768	DEK	9.036704984	0.018799003	1
feature_4774	DENND1A	2.070615079	0.134761878	1
feature_4790	DENND6A	1.617445671	0.042807413	1
feature_4806	DERA	1.258112126	0.03984066	1
feature_4835	DGKH	2.374803377	0.078087507	1
feature_4873	DHTKD1	1.156716027	0.145879114	1
feature_4877	DHX30	1.004621878	0.035727847	1
feature_4884	DHX36	2.044376615	0.091268796	1

feature_4898	DIAPH2	5.629707106	0.173845011	1
feature_4903	DICER1	1.626340065	0.090887888	1
feature_4905	DIDO1	1.383523091	0.108474615	1
feature_4948	DLEU1	1.054430488	0.192736112	1
feature_5006	DNA2	1.052651609	0.054587686	1
feature_5080	DNAJC2	1.162942103	0.106639189	1
feature_5117	DNM1L	1.442670815	0.014570351	1
feature_5126	DNMT1	3.790791008	0.072696338	1
feature_5141	DOCK10	2.999634602	0.068266905	1
feature_5144	DOCK2	3.398992923	0.095742594	1
feature_5160	DOK3	1.125140926	0.03398844	1
feature_5168	DOP1A	1.046425533	0.028068021	1
feature_5197	DPP7	1.324820086	0.210696515	1
feature_5223	DPYD	3.189085209	0.134911495	1
feature_5237	DRAP1	1.366179021	0.065023342	1
feature_5278	DTL	2.057718207	0.177573571	1
feature_5339	DYM	2.205365158	0.053944775	1
feature_5349	DYNC2I1	1.096678863	0.244024774	1
feature_5381	E2F3	3.515954213	0.106565033	1
feature_5410	EBPL	1.528946444	0.112476763	1
feature_5415	ECHDC1	2.221375069	0.140717404	1
feature_5418	ECHS1	1.053096329	0.041004082	1
feature_5441	EDF1	3.476374156	0.085427903	1
feature_5452	EEA1	1.874493676	0.207519684	1
feature_5454	EEF1A1	137.6412022	0.012045267	1
feature_5488	EEF1B2	21.26516349	0.120909104	1
feature_5511	EEF2	11.05884561	0.057942625	1
feature_5553	EFR3A	1.344387754	0.126186355	1
feature_5555	EFTUD2	1.235876139	0.082978633	1
feature_5585	EHMT1	2.063054843	0.077695116	1
feature_5589	EID1	3.565762823	0.133023146	1
feature_5613	EIF2AK4	1.45067577	0.204540286	1
feature_5622	EIF2S2	2.128428644	0.12920017	1
feature_5631	EIF3A	6.001937523	0.135311281	1
feature_5632	EIF3B	2.259176246	0.236864955	1
feature_5636	EIF3E	6.317688533	0.127084987	1
feature_5641	EIF3I	2.105303218	0.049770789	1
feature_5643	EIF3J	1.63478974	0.178320026	1
feature_5645	EIF3K	5.030224905	0.120138	1
feature_5652	EIF3M	2.767046181	0.037778618	1
feature_5669	EIF4B	3.15261819	0.021165396	1
feature_5703	EIF6	1.176728415	0.00894202	1
feature_5743	ELOB	3.249567092	0.048897611	1
feature_6406	ENSG00000224905	1.076666475	0.084714704	1
feature_6722	ENSG00000227598	2.930703043	0.026790469	1
feature_8259	ENSG00000244313	1.305697137	0.074210697	1
feature_8938	ENSG00000253693	1.96921898	0.214563022	1
feature_9075	ENSG00000254420	1.572084258	0.012311597	1
feature_11130	ENSG00000267383	1.241212776	0.119549481	1
feature_12319	ENSG00000273748	1.161163224	0.154390748	1

feature_14231	ENSG00000286153	1.375962855	0.200360301	1
feature_16455	ENSG00000290928	1.066437921	0.049176085	1
feature_16630	ENY2	1.93453084	0.054287853	1
feature_16640	EP400	1.80244908	0.151612444	1
feature_16642	EPB41	2.279633354	0.043741044	1
feature_16691	EPRS1	1.436000019	0.025294512	1
feature_16727	ERG	4.155461189	0.228303373	1
feature_16732	ERH	3.511951735	0.012171436	1
feature_16760	ERP29	2.872889478	0.048031778	1
feature_16778	ESD	2.080843633	0.012273248	1
feature_16779	ESF1	1.448896892	0.203505305	1
feature_16802	ETFA	2.659868726	0.042314806	1
feature_16808	ETNK1	1.488921668	0.141872906	1
feature_16832	EVI5	1.528057004	0.017982127	1
feature_16937	FAIM	1.337272238	0.003927179	1
feature_16961	FAM120A	1.896729663	0.12629865	1
feature_16976	FAM135A	2.339225798	0.099633673	1
feature_17047	FAM204A	1.33504864	0.044255883	1
feature_17193	FARS2	2.946712954	0.107163024	1
feature_17211	FAU	20.51002939	0.044530378	1
feature_17217	FBL	2.01813815	0.218500733	1
feature_17237	FBXL17	3.088133829	0.14829115	1
feature_17242	FBXL20	1.81890371	0.174544615	1
feature_17245	FBXL4	1.30213938	0.183884632	1
feature_17293	FBXW4	1.166944581	0.112450339	1
feature_17337	FDPS	1.505821017	0.037054213	1
feature_17362	FERMT3	1.723288967	0.046477903	1
feature_17446	FIS1	1.681485312	0.195794722	1
feature_17456	FKBP1A	1.858928486	0.120770076	1
feature_17460	FKBP3	1.598322722	0.186858563	1
feature_17580	FOXK1	1.263448763	0.169641578	1
feature_17590	FOXO3	3.27046892	0.070807397	1
feature_17606	FPGS	1.515604852	0.119765522	1
feature_17686	FTL	9.825193071	0.182274198	1
feature_17696	FTO	3.122377248	0.153527248	1
feature_17700	FTX	4.864344444	0.208850608	1
feature_17757	G3BP1	2.429503904	0.12139884	1
feature_17764	GAB1	3.027207226	0.153370553	1
feature_17765	GAB2	6.676577358	0.008836776	1
feature_17822	GALNT1	2.776830016	0.024584983	1
feature_17833	GALNT2	9.366242306	0.219040834	1
feature_17851	GANAB	1.019297629	0.001288713	1
feature_17854	GAPDH	20.68124648	0.123527167	1
feature_17905	GAS5	9.561029549	0.118319843	1
feature_17924	GATAD2A	2.21870675	0.104804534	1
feature_17996	GDI2	3.393211566	0.00446584	1
feature_18039	GGA2	1.336382799	0.098659253	1
feature_18090	GIT2	1.530725323	0.01855026	1
feature_18141	GLO1	1.320372889	0.083501424	1
feature_18153	GLRX3	1.123362047	0.150917177	1

feature_18158	GLS	1.913629013	0.141271292	1
feature_18183	GMDS-DT	3.323835288	0.12231931	1
feature_18197	GMPS	1.499150221	0.149817373	1
feature_18204	GNA15	2.458855406	0.039280523	1
feature_18255	GNPTAB	2.423277828	0.143638046	1
feature_18311	GOPC	1.763313743	0.12306275	1
feature_18342	GPATCH2	1.11713597	0.118193667	1
feature_18361	GPD2	1.016629311	0.056983674	1
feature_18487	GRB2	3.524403888	0.009140068	1
feature_18577	GSPT1	1.746414393	0.070043198	1
feature_18587	GSTK1	1.149600511	0.108088153	1
feature_18596	GSTP1	6.735725083	0.253083247	1
feature_18607	GTF2F2	1.765982061	0.001245205	1
feature_18641	GTF3C6	2.226266986	0.162957484	1
feature_18648	GTPBP4	1.419545389	0.130039814	1
feature_18669	GUK1	1.228760623	0.113715563	1
feature_18822	HACD3	1.676593395	0.127477109	1
feature_18853	HAUS1	1.188291128	0.04710484	1
feature_18897	HCLS1	1.728625604	0.061910977	1
feature_18913	HDAC2	3.444354335	0.044857152	1
feature_18925	HDDC2	1.892727186	0.12965148	1
feature_18934	HDLBP	1.748637992	0.122946721	1
feature_18961	HELLS	3.089023268	0.224046403	1
feature_18984	HERC4	2.150664631	0.051386608	1
feature_19027	HIBADH	1.01751875	0.023743528	1
feature_19028	HIBCH	1.011292674	0.107571032	1
feature_19033	HIF1A	1.422213708	0.012811757	1
feature_19071	HIVEP2	6.030844305	0.122712207	1
feature_19116	HM13	1.546735233	0.202619826	1
feature_19289	HNRNPAB	3.587998809	0.064966933	1
feature_19301	HNRNPF	4.584615732	0.062587973	1
feature_19379	HPRT1	1.341274716	0.12250176	1
feature_19494	HSPB11	1.342608875	0.040825488	1
feature_19541	HTR1F	11.41640028	0.142558155	1
feature_19558	HTRA3	2.230269463	0.156781498	1
feature_19560	HTT	1.233207821	0.008398907	1
feature_19566	HUWE1	1.863375683	0.017255694	1
feature_19601	ICE2	1.040199457	0.112396932	1
feature_19656	IFITM2	3.847715134	0.076463048	1
feature_19703	IGF1R	3.01964699	0.053961778	1
feature_19817	IKBKB	1.310144335	0.170530552	1
feature_19822	IKZF1	13.97531762	0.015810284	1
feature_19925	IMMT	1.263893482	0.004020426	1
feature_19952	ING5	1.082892551	0.253178148	1
feature_19974	INPP4A	1.11847013	0.152954549	1
feature_20014	INTS7	1.10112606	0.087772969	1
feature_20019	INVS	2.217817311	0.231818006	1
feature_20030	IPO7	2.891567707	0.136954626	1
feature_20041	IQCB1	2.074172837	0.044159318	1
feature_20069	IRAK3	1.231873662	0.134788903	1

feature_20071	IREB2	1.091786946	0.053113112	1
feature_20072	IRF1	1.824240347	0.006731399	1
feature_20115	ITFG1	3.135274121	0.011017343	1
feature_20168	ITM2C	2.014580392	0.210344576	1
feature_20251	JTB	1.395530524	0.030618705	1
feature_20478	KHSRP	1.7352964	0.079137708	1
feature_20518	KIF16B	1.187401688	0.076229151	1
feature_20701	KNTC1	1.742411916	0.049206236	1
feature_20712	KPNB1	4.140785438	0.151761194	1
feature_20831	LAMP1	1.702831859	0.194425942	1
feature_20836	LAMTOR1	1.019742349	0.155563194	1
feature_20840	LAMTOR4	1.854036569	0.093607201	1
feature_20841	LAMTOR5	1.578755054	0.028973253	1
feature_20847	LAP3	1.159829065	0.229330641	1
feature_20857	LARP1	2.737694679	0.178087475	1
feature_20860	LARP4	1.277235074	0.118900076	1
feature_20861	LARP4B	2.31876869	0.152185097	1
feature_20866	LARS1	2.03548222	0.243940396	1
feature_20891	LCLAT1	1.40086716	0.059194143	1
feature_20909	LDAH	1.223423987	0.236337534	1
feature_20914	LDHA	5.147186195	0.167535387	1
feature_20921	LDHB	5.582122094	0.14007899	1
feature_20984	LGR6	4.93905736	0.157750576	1
feature_21004	LIG1	1.069550959	0.10307109	1
feature_21022	LIMD1	1.436444739	0.134912606	1
feature_21024	LIMD2	1.443560255	0.170395084	1
feature_21028	LIMS1	1.688600828	0.009762768	1
feature_21041	LINC-PINT	1.164720982	0.083006678	1
feature_21322	LINC01004	1.445783853	0.081025816	1
feature_21337	LINC01036	2.128428644	0.089492024	1
feature_21542	LINC01473	1.277679794	0.2187949	1
feature_22475	LMAN1	1.130032843	0.049683152	1
feature_22531	LONP2	1.303028819	0.158134578	1
feature_22569	LRBA	2.745699634	0.056863707	1
feature_22606	LRPPRC	2.051047411	0.206026777	1
feature_22700	LRRK1	1.629897823	0.250158801	1
feature_22727	LSM2	1.750416871	0.008625994	1
feature_22728	LSM3	1.970108419	0.101433739	1
feature_22731	LSM5	1.473801197	0.091103678	1
feature_22732	LSM6	1.061546004	0.162896726	1
feature_22734	LSM7	3.547974033	0.134132242	1
feature_22735	LSM8	1.432886981	0.056465083	1
feature_22764	LUC7L3	5.141849558	0.09869211	1
feature_22770	LUZP1	1.543177475	0.056845695	1
feature_22797	LYPLA1	2.114642333	0.017253534	1
feature_22803	LYRM2	1.11847013	0.077429946	1
feature_22832	MACF1	7.163990186	0.037542027	1
feature_22882	MAILR	1.480471993	0.140720687	1
feature_22901	MAML3	9.271072283	0.114315715	1
feature_22930	MAP1LC3B	2.718127011	0.08625144	1

feature_22955	MAP3K20	2.348564912	0.170360434	1
feature_22961	MAP3K5	6.604532761	0.149483292	1
feature_22970	MAP4	2.697669903	0.06687494	1
feature_22976	MAP4K5	1.822016748	0.115288899	1
feature_22992	MAPK14	2.521116169	0.039779855	1
feature_23060	MAT2A	2.038595258	0.154298259	1
feature_23075	MAZ	2.332555002	0.073806141	1
feature_23079	MBD2	1.500484381	0.079858144	1
feature_23092	MBNL3	1.492924145	0.212873787	1
feature_23119	MCM10	1.115357091	0.217812923	1
feature_23130	MCM9	1.656581007	0.042590411	1
feature_23131	MCMBP	1.352837429	0.140623475	1
feature_23158	MDH2	2.047044933	0.143156832	1
feature_23163	MDN1	2.534902481	0.219637855	1
feature_23168	ME2	1.302584099	0.13585374	1
feature_23201	MED28	1.009069075	0.061001535	1
feature_23214	MEF2A	7.8742076	0.008163558	1
feature_23217	MEF2C-AS1	2.547799353	0.132528936	1
feature_23262	METAP2	2.057273487	0.126508277	1
feature_23353	METTL15	1.337716958	0.011546442	1
feature_23368	METTL26	1.422658427	0.133134368	1
feature_23408	MFSD14CP.1	1.195851363	0.072197591	1
feature_23446	MGST3	1.444449694	0.138180621	1
feature_23467	MICOS10	1.326598965	0.254799618	1
feature_23575	MIR4432HG	1.013071553	0.07446403	1
feature_23727	MMS22L	4.12032833	0.135129363	1
feature_23785	MPC1	1.483140311	0.245368414	1
feature_23818	MPRIP	1.553406029	0.034267276	1
feature_23835	MRE11	1.599212161	0.030315799	1
feature_23862	MRPL1	1.211416554	0.178744678	1
feature_23867	MRPL14	1.86871232	0.01123502	1
feature_23890	MRPL33	3.176188336	0.154648776	1
feature_23891	MRPL34	1.26745124	0.166962888	1
feature_23928	MRPL57	1.689045548	0.141439615	1
feature_23962	MRPS27	1.303918258	0.254864962	1
feature_23972	MRPS34	1.607217117	0.226577951	1
feature_24014	MSH2	1.670367319	0.156642737	1
feature_24020	MSH6	12.68385152	0.029092133	1
feature_24030	MSN	1.950540751	0.13573263	1
feature_24047	MT-ATP8	2.41927535	0.159612029	1
feature_24052	MT-ND1	22.83368999	0.117565569	1
feature_24053	MT-ND2	23.76404368	0.106577955	1
feature_24056	MT-ND4L	3.37008614	0.166518983	1
feature_24057	MT-ND5	15.18895778	0.184964789	1
feature_24058	MT-ND6	1.445783853	0.191811155	1
feature_24313	MYCBP2	7.34054392	0.026760794	1
feature_24324	MYDGF	1.10157078	0.209108091	1
feature_24339	MYH9	3.594224886	0.023676275	1
feature_24354	MYL6B	1.319483449	0.17184859	1
feature_24426	MZT2A	1.337272238	0.244150515	1

feature_24427	MZT2B	4.616635553	0.047472664	1
feature_24437	NAA15	2.425056706	0.031381784	1
feature_24443	NAA38	2.261844564	0.190568909	1
feature_24445	NAA50	1.737964718	0.053981239	1
feature_24457	NACA	25.00080925	0.174071933	1
feature_24499	NAP1L1	19.07714241	0.142421577	1
feature_24505	NAP1L4	1.18473337	0.0356363	1
feature_24522	NARS2	1.726846725	0.180075885	1
feature_24542	NBDY	1.442226096	0.113968294	1
feature_24592	NCF4	1.569415939	0.104409721	1
feature_24596	NCK2	1.102460219	0.187867575	1
feature_24631	NDC1	1.187846408	0.024373727	1
feature_24649	NDUFA1	2.060831245	0.078592883	1
feature_24650	NDUFA10	1.403980198	0.128465214	1
feature_24651	NDUFA11	1.648576052	0.086129262	1
feature_24652	NDUFA12	1.446673293	0.047818175	1
feature_24659	NDUFA4	4.370260821	0.0852522	1
feature_24672	NDUFAB1	1.911850134	0.150384056	1
feature_24687	NDUFB1	1.25144133	0.1823768	1
feature_24688	NDUFB10	1.904734618	0.177878831	1
feature_24689	NDUFB11	3.132605802	0.04690199	1
feature_24696	NDUFB3	1.476914235	0.023835666	1
feature_24697	NDUFB4	1.760645425	0.005001972	1
feature_24706	NDUFB7	1.676148675	0.087922906	1
feature_24707	NDUFB8	1.856260167	0.146399303	1
feature_24709	NDUFB9	1.288797788	0.18881829	1
feature_24718	NDUFS3	1.122472607	0.017089984	1
feature_24719	NDUFS4	1.455567688	0.001276462	1
feature_24720	NDUFS5	3.617350312	0.081826714	1
feature_24722	NDUFS6	2.658979286	0.143948578	1
feature_24725	NDUFS8	1.281237552	0.083799074	1
feature_24726	NDUFV1	1.075332315	0.033931466	1
feature_24752	NEDD8	2.324994766	0.088907587	1
feature_24845	NFX1	2.057273487	0.188300645	1
feature_24859	NGLY1	1.654357408	0.042588192	1
feature_24867	NHP2	2.099521862	0.136730375	1
feature_24882	NIFK	1.6023252	0.197366798	1
feature_24906	NIPSNAP2	1.15093467	0.020833967	1
feature_24928	NKTR	3.334953281	0.088615086	1
feature_24938	NLK	1.529391163	0.020411022	1
feature_25001	NOL10	1.380410053	0.079711415	1
feature_25009	NOL7	2.50199322	0.094651702	1
feature_25010	NOL8	1.104683818	0.133732263	1
feature_25020	NOP10	1.438668338	0.166613737	1
feature_25031	NOP58	4.00425648	0.072312887	1
feature_25224	NSA2	2.108416256	0.042098385	1
feature_25242	NSMAF	1.298136902	0.158335372	1
feature_25249	NSRP1	1.779768373	0.103812097	1
feature_25263	NT5C3A	1.001953559	0.052274613	1
feature_25301	NUBPL	1.013516273	0.240410327	1

feature_25308	NUDC	2.078175314	0.023478976	1
feature_25314	NUDT1	1.814456512	0.166015092	1
feature_25333	NUDT3	1.880719753	0.08467157	1
feature_25358	NUP205	1.078445354	0.006989928	1
feature_25377	NUP93	1.153158269	0.022654623	1
feature_25389	NUTM2A-AS1	1.362176544	0.060470094	1
feature_25397	NVL	1.068661519	0.067427495	1
feature_25471	OGDH	1.016184591	0.17580586	1
feature_25482	OGT	1.620558709	0.087569352	1
feature_25486	OLA1	3.988246569	0.148581732	1
feature_25622	ORC3	1.163831542	0.092184219	1
feature_25625	ORC6	1.360397665	0.182339066	1
feature_25626	ORMDL1	1.058877685	0.037449276	1
feature_25658	OSTC	2.213370113	0.142410716	1
feature_25760	PABPC1	15.81467844	0.107279599	1
feature_25767	PABPC4	1.542732755	0.240568713	1
feature_25866	PARK7	3.310048976	0.147972077	1
feature_25872	PARP1	5.953463072	0.060913546	1
feature_25877	PARP14	2.000794081	0.08497401	1
feature_25883	PARP4	1.218976789	0.080297034	1
feature_25887	PARP8	9.271072283	0.062539498	1
feature_25895	PARVG	1.28524003	0.178082536	1
feature_25920	PAXX	1.08422671	0.185516231	1
feature_25928	PBX3	14.15409496	0.105423373	1
feature_26003	PCED1B-AS1	1.087339748	0.205517978	1
feature_26016	PCLAF	3.985578251	0.038711233	1
feature_26020	PCMTD1	1.4017566	0.001788635	1
feature_26023	PCNA	3.727196086	0.091258678	1
feature_26057	PDAP1	1.556519067	0.067788965	1
feature_26064	PDCD2	1.697050503	0.093166942	1
feature_26093	PDE3B	1.446228573	0.017534248	1
feature_26184	PEBP1	7.103063583	0.211216575	1
feature_26237	PFDN2	2.072838678	0.126846758	1
feature_26245	PFKL	1.452899369	0.030716662	1
feature_26249	PFN1	5.407347239	0.046264447	1
feature_26260	PGAM1	1.093565825	0.203891977	1
feature_26281	PGD	1.064214322	0.054425729	1
feature_26286	PGK1	3.274026678	0.021341955	1
feature_26288	PGLS	1.521386208	0.12110452	1
feature_26326	PHB2	2.368132581	0.037975098	1
feature_26331	PHC3	1.704166019	0.142593946	1
feature_26342	PHF14	3.843267936	0.193626265	1
feature_26345	PHF20	2.400152401	0.007661946	1
feature_26352	PHF3	2.623401708	0.01564783	1
feature_26366	PHKB	2.441511337	0.081080708	1
feature_26431	PIGN	1.223868706	0.030232757	1
feature_26455	PIK3CB	1.068661519	0.038868039	1
feature_26462	PIK3R1	3.333619122	0.103588711	1
feature_26494	PIP5K1A	1.489811107	0.074960466	1
feature_26505	PITPNB	2.006130718	0.179346003	1

feature_26546	PKIG	1.369736779	0.012661151	1
feature_26548	PKM	2.996076844	0.222330871	1
feature_26588	PLAC8	1.273232597	0.083127746	1
feature_26685	PLP2	1.182509771	0.207905816	1
feature_26750	PNISR	7.673639001	0.088332639	1
feature_26768	PNN	5.209891677	0.193947014	1
feature_26772	PNP	1.006400757	0.129794232	1
feature_26781	PNPT1	1.146042753	0.155933804	1
feature_26804	POLA1	2.15244351	0.181601449	1
feature_26816	POLE3	1.674814516	0.044180795	1
feature_26832	POLR1C	1.108241576	0.090183323	1
feature_26833	POLR1D	1.772652857	0.030654598	1
feature_26835	POLR1F	2.550467671	0.190696141	1
feature_26845	POLR2F	1.01751875	0.176017218	1
feature_26856	POLR2L	1.657915166	0.17417501	1
feature_26874	POM121	1.16783402	0.127633463	1
feature_26885	POMP	3.160178426	0.036877057	1
feature_26962	PPIA	15.32904449	0.044599265	1
feature_27002	PPIB	3.022760028	0.17438331	1
feature_27010	PPIH	1.327933124	0.121036264	1
feature_27018	PPIP5K2	1.20074328	0.003774157	1
feature_27027	PPM1G	2.599831562	0.033638757	1
feature_27122	PPP3CB	1.387080849	0.023829843	1
feature_27189	PRDX3	1.505376298	0.012038954	1
feature_27193	PRDX5	1.088673907	0.03113554	1
feature_27194	PRDX6	2.036371659	0.051754285	1
feature_27198	PRELID1	1.739743597	0.245722316	1
feature_27210	PREX1	1.43466586	0.054233784	1
feature_27232	PRKACB	1.199409121	0.223288847	1
feature_27274	PRKRIP1	1.447562732	0.061191389	1
feature_27281	PRMT1	2.329441964	0.168876213	1
feature_27284	PRMT3	1.829576983	0.143369153	1
feature_27424	PSD3	4.276869676	0.008886581	1
feature_27433	PSMA1	2.079509474	0.07886985	1
feature_27439	PSMA3	1.902955739	0.174336099	1
feature_27441	PSMA4	2.554025429	0.028599622	1
feature_27442	PSMA5	1.898953262	0.073449426	1
feature_27447	PSMA7	5.876526558	0.216881285	1
feature_27449	PSMB1	3.776559976	0.103453979	1
feature_27455	PSMB4	1.462683203	0.004903532	1
feature_27456	PSMB5	1.21808735	0.223653449	1
feature_27459	PSMB8	2.163116783	0.005885813	1
feature_27473	PSMC5	1.565858181	0.056517255	1
feature_27483	PSMD13	1.246104693	0.092346382	1
feature_27494	PSMD7	2.28541471	0.116136925	1
feature_27500	PSME1	3.622242229	0.006901734	1
feature_27501	PSME2	2.646527134	0.153761715	1
feature_27583	PTK2	6.333698444	0.132655832	1
feature_27584	PTK2B	4.478772435	0.007149021	1
feature_27645	PTPRJ	1.021076508	0.066291404	1

feature_27710	PYCARD	1.953209069	0.193948968	1
feature_27751	R3HDM2	3.147281554	0.148726929	1
feature_27800	RAB34	1.055764647	0.247823557	1
feature_27843	RABEP1	1.138927237	0.197931242	1
feature_27847	RABGAP1L	2.274741437	0.091397461	1
feature_27860	RABL6	1.378186454	0.104998283	1
feature_27869	RACK1	29.13092142	0.104416072	1
feature_27881	RAD23A	1.90251102	0.000428497	1
feature_27882	RAD23B	1.133590601	0.207075103	1
feature_27885	RAD51	1.061101284	0.018886357	1
feature_27890	RAD51B	4.281316874	0.134875934	1
feature_27896	RAD54L2	1.010403234	0.045976526	1
feature_27915	RALBP1	2.028811424	0.12409711	1
feature_27934	RAN	7.828846187	0.052349696	1
feature_27935	RANBP1	4.451644532	0.238540914	1
feature_27967	RAPGEF1	2.701227661	0.24743888	1
feature_27987	RASA2	2.032813902	0.162553609	1
feature_28008	RASGRP2	1.329267283	0.044047301	1
feature_28022	RASSF3	1.335938079	0.130392556	1
feature_28039	RBBP4	2.814186473	0.005031102	1
feature_28046	RBBP8	1.273677317	0.065217455	1
feature_28055	RBIS	1.509823495	0.213724489	1
feature_28058	RBL1	3.225107507	0.01006217	1
feature_28070	RBM17	1.520496769	0.056551437	1
feature_28082	RBM26	2.832864702	0.090650245	1
feature_28086	RBM3	5.195215926	0.050098691	1
feature_28135	RBX1	2.755483468	0.012283611	1
feature_28150	RCC2	1.814456512	0.153748216	1
feature_28189	RECQL	1.141595556	0.022258233	1
feature_28243	RFC2	1.096678863	0.135146512	1
feature_28244	RFC3	1.533838361	0.099832198	1
feature_28259	RFTN1	2.065278442	0.242625517	1
feature_28265	RFX3-DT	1.011737394	0.062118766	1
feature_28269	RFX7	2.137323039	0.014649681	1
feature_28373	RIF1	2.556249028	0.093240673	1
feature_28378	RILPL2	1.509378775	0.031493564	1
feature_28397	RIOK1	1.154492428	0.013552424	1
feature_28408	RIPOR2	2.677212795	0.122736948	1
feature_28784	RNASEH2B	4.468099162	0.034404405	1
feature_28798	RNF111	1.359063505	0.01381398	1
feature_28810	RNF13	1.396864683	0.106421697	1
feature_28811	RNF130	6.057082769	0.107669984	1
feature_28858	RNF216	1.680595873	0.099869487	1
feature_28900	RNPS1	2.13598888	0.03496316	1
feature_29210	ROMO1	1.294134424	0.119112983	1
feature_29229	RPA2	1.188735847	0.136098812	1
feature_29230	RPA3	1.328377844	0.051057733	1
feature_29235	RPAP2	1.156716027	0.058534506	1
feature_29255	RPIA	1.064214322	0.189479138	1
feature_29256	RPL10	66.02220219	0.07430298	1

feature_29257	RPL10A	40.27204018	0.125248747	1
feature_29279	RPL11	54.05034696	0.161754486	1
feature_29316	RPL13	81.32812125	0.183279607	1
feature_29317	RPL13A	61.75155859	0.162462557	1
feature_29334	RPL14	27.19416698	0.216142352	1
feature_29339	RPL15	27.98532339	0.086004431	1
feature_29346	RPL17	1.031749782	0.018716354	1
feature_29368	RPL18	37.83675492	0.153348663	1
feature_29369	RPL18A	47.09804337	0.110904874	1
feature_29375	RPL18AP3	1.305697137	0.201153191	1
feature_29381	RPL19	53.95161918	0.096409457	1
feature_29388	RPL21	24.63925211	0.117977373	1
feature_29432	RPL22	5.718651052	0.100114685	1
feature_29433	RPL22L1	10.69328599	0.246676811	1
feature_29444	RPL23	37.00957622	0.073390899	1
feature_29445	RPL23A	30.12131227	0.159503918	1
feature_29496	RPL24	27.86569378	0.18957062	1
feature_29501	RPL26	21.19000586	0.106377229	1
feature_29514	RPL27	22.73674109	0.142325431	1
feature_29515	RPL27A	33.00309614	0.211298206	1
feature_29523	RPL28	45.59622483	0.090191392	1
feature_29524	RPL29	36.82679641	0.115449044	1
feature_29532	RPL3	41.43231397	0.145491422	1
feature_29533	RPL30	42.95103186	0.122363975	1
feature_29542	RPL31	15.0564313	0.109346875	1
feature_29558	RPL32	51.96861389	0.127513586	1
feature_29582	RPL34	37.51344367	0.085477332	1
feature_29597	RPL35	25.94494925	0.23467069	1
feature_29598	RPL35A	31.73431074	0.134862974	1
feature_29630	RPL36A	3.282031633	0.141462827	1
feature_29632	RPL36AL	3.635139101	0.04116976	1
feature_29673	RPL37	41.11878655	0.102258169	1
feature_29674	RPL37A	44.00768594	0.188420899	1
feature_29685	RPL38	16.25806402	0.157088689	1
feature_29690	RPL39	36.41009402	0.081982359	1
feature_29709	RPL4	15.65546878	0.133975879	1
feature_29710	RPL41	52.52051108	0.075138726	1
feature_29719	RPL5	30.34411685	0.149153838	1
feature_29744	RPL6	26.46927381	0.097868133	1
feature_29763	RPL7	12.9720299	0.029553997	1
feature_29764	RPL7A	52.14160987	0.185722006	1
feature_29855	RPL8	25.53669653	0.186745241	1
feature_29857	RPL9	29.42977308	0.150018081	1
feature_29882	RPLP2	40.92444403	0.111153081	1
feature_29885	RPN2	2.065723162	0.048125474	1
feature_29898	RPS10	9.061164569	0.025066396	1
feature_29907	RPS11	44.97005945	0.04131227	1
feature_29922	RPS13	32.16479944	0.055442471	1
feature_29927	RPS14	54.53731507	0.241204316	1
feature_29931	RPS15	35.62738729	0.154103566	1

feature_29932	RPS15A	36.20552294	0.088204797	1
feature_29955	RPS16	40.85818079	0.150167795	1
feature_29973	RPS20	27.74028281	0.111532309	1
feature_29986	RPS21	24.4947182	0.100603311	1
feature_29988	RPS23	61.38422009	0.182231959	1
feature_29996	RPS24	44.44039824	0.102680868	1
feature_30005	RPS25	14.98171838	0.048581577	1
feature_30008	RPS26	4.515239454	0.083767316	1
feature_30030	RPS27	35.56201348	0.063204083	1
feature_30031	RPS27A	52.60856559	0.22907966	1
feature_30054	RPS28	32.70646808	0.138855781	1
feature_30057	RPS29	25.39705454	0.130039615	1
feature_30092	RPS3	53.91070497	0.221107026	1
feature_30093	RPS3A	37.62595777	0.056407212	1
feature_30151	RPS5	28.828512	0.161104213	1
feature_30155	RPS6	66.71952273	0.143727726	1
feature_30177	RPS7	38.32905967	0.142167268	1
feature_30191	RPS8	59.60845419	0.117819605	1
feature_30195	RPS9	34.32302431	0.180837844	1
feature_30196	RPSA	25.12488606	0.153583491	1
feature_30232	RPTOR	1.657025727	0.204804809	1
feature_30245	RRAS2	1.247883572	0.181170066	1
feature_30261	RRP1B	1.129588123	0.204115554	1
feature_30279	RSL1D1	2.759485946	0.121659474	1
feature_30302	RSU1	1.308810176	0.229670787	1
feature_30348	RUNX1	18.35269396	0.094550434	1
feature_30358	RUVBL1	1.228760623	0.218696295	1
feature_30361	RWDD1	2.004796558	0.091068755	1
feature_30396	S100A6	1.146042753	0.392573255	1
feature_30500	SCAI	1.279458673	0.131808202	1
feature_30530	SCCPDH	1.758421826	0.1641964	1
feature_30549	SCMH1	4.502342581	0.232523657	1
feature_30596	SDAD1	1.128253964	0.093801077	1
feature_30665	SEC61B	2.573148378	0.066678197	1
feature_30666	SEC61G	1.846476333	0.053577349	1
feature_30668	SEC62	1.617000951	0.010013113	1
feature_30670	SEC63	3.836152421	0.015598331	1
feature_30675	SEH1L	1.321707048	0.121227878	1
feature_30683	SELENOI	1.077555914	0.22192797	1
feature_30690	SELENOO	1.295023864	0.175102493	1
feature_30702	SEM1	2.646971853	0.085812465	1
feature_30744	SEPTIN11	1.773542297	0.182289027	1
feature_30770	SEPTIN9	4.858118368	0.071662208	1
feature_30773	SERBP1	7.461507688	0.235189584	1
feature_30782	SERF2	8.252219374	0.035703775	1
feature_30790	SERINC5	2.112863454	0.12680063	1
feature_30791	SERP1	2.510442895	0.04104892	1
feature_30794	SERPINB1	1.712170974	0.045782736	1
feature_30833	SETD3	1.010403234	0.09995868	1
feature_30836	SETD5	2.446847973	0.008109438	1

feature_30841	SETDB2	1.206524637	0.081174789	1
feature_30866	SF3A3	1.321262328	0.101981423	1
feature_30871	SF3B3	1.411095714	0.141395548	1
feature_30873	SF3B5	2.830641103	0.010505827	1
feature_30877	SFMBT2	2.999634602	0.163462205	1
feature_31002	SHLD2	1.670812039	0.1520427	1
feature_31015	SHPRH	1.567192341	0.134328313	1
feature_31057	SIMC1	1.552516589	0.067759351	1
feature_31068	SIPA1L3	1.616111511	0.028347917	1
feature_31084	SIVA1	1.80111492	0.102600483	1
feature_31098	SKIC3	1.492034706	0.048746288	1
feature_31114	SLBP	1.582312812	0.078028772	1
feature_31208	SLC23A2	1.074887596	0.112191261	1
feature_31219	SLC25A13	1.143374435	0.185131091	1
feature_31242	SLC25A3	6.691253109	0.067423607	1
feature_31251	SLC25A36	1.214084872	0.019429124	1
feature_31267	SLC25A5	6.674353759	0.080368289	1
feature_31385	SLC39A11	1.337716958	0.148239441	1
feature_31434	SLC4A7	2.716348132	0.01597827	1
feature_31540	SLIRP	1.710836815	0.140084041	1
feature_31596	SMC1B	5.563443865	0.110233628	1
feature_31604	SMC6	1.961658744	0.01000005	1
feature_31612	SMDT1	1.206969357	0.021374726	1
feature_31624	SMG6	1.295913303	0.174700996	1
feature_31650	SMIM26	1.200298561	0.209868972	1
feature_31686	SMS	1.430218663	0.11968016	1
feature_31697	SMYD3	7.284953953	0.251493548	1
feature_31723	SND1	3.191308807	0.20943007	1
feature_31728	SNF8	1.098902461	0.024099631	1
feature_31730	SNHG1	1.379075893	0.035208365	1
feature_31745	SNHG29	21.99717218	0.210993029	1
feature_31751	SNHG5	17.35385344	0.228019135	1
feature_31752	SNHG6	9.967948105	0.054700546	1
feature_31854	SNRNP25	1.154047708	0.204225247	1
feature_31859	SNRNP70	2.439732458	0.171874225	1
feature_31861	SNRPA1	1.390638606	0.079013182	1
feature_31864	SNRPB	4.628642986	0.153513933	1
feature_31866	SNRPC	2.641190497	0.008167566	1
feature_31871	SNRPD1	4.472546359	0.235593743	1
feature_31872	SNRPD2	4.550372313	0.05587709	1
feature_31874	SNRPD3	1.330156723	0.063581103	1
feature_31875	SNRPE	3.667158922	0.142367956	1
feature_31880	SNRPF	4.031384384	0.220912364	1
feature_31885	SNRPG	2.379695294	0.00152585	1
feature_31905	SNU13	2.707898457	0.173497905	1
feature_31926	SNX25	1.830021703	0.003123912	1
feature_31929	SNX29	1.242546935	0.111274058	1
feature_31938	SNX5	1.387525568	0.202705615	1
feature_31952	SOCS2-AS1	1.532948921	0.04007608	1
feature_31961	SOD1	2.883562752	0.185566475	1

feature_31965	SOD2	1.838471378	0.094569274	1
feature_32004	SP100	1.33549336	0.187736104	1
feature_32005	SP110	1.269230119	0.004705307	1
feature_32075	SPCS1	2.554470149	0.02646889	1
feature_32080	SPCS3	1.035307539	0.024010607	1
feature_32115	SPG11	1.40086716	0.045567569	1
feature_32125	SPIDR	5.133844603	0.220955473	1
feature_32140	SPINT2	1.086005589	0.031150943	1
feature_32207	SRBD1	1.309699615	0.165858985	1
feature_32219	SREK1IP1	1.009513795	0.030096204	1
feature_32232	SRGN	1.46846456	0.141044103	1
feature_32248	SRP72	2.542462716	0.098833892	1
feature_32253	SRPK1	2.664315923	0.048243926	1
feature_32254	SRPK2	1.466240961	0.1241633	1
feature_32274	SRSF11	5.224567428	0.086106219	1
feature_32276	SRSF2	5.233017103	0.037420258	1
feature_32295	SSB	3.239338538	0.192744466	1
feature_32299	SSBP1	2.741252437	0.222266436	1
feature_32317	SSR4	2.971172539	0.097220029	1
feature_32323	SSU72	1.203856318	0.082632214	1
feature_32452	STK3	2.525118646	0.08605944	1
feature_32529	STX7	1.693492745	0.217439688	1
feature_32544	SUB1	4.663775845	0.042017684	1
feature_32546	SUCLA2	1.096234143	0.103586634	1
feature_32551	SUCLG2	2.459300126	0.018821472	1
feature_32562	SUGP2	1.550737711	0.150047527	1
feature_32585	SUMF2	1.214529592	0.125167835	1
feature_32601	SUPT16H	2.232937782	0.134337774	1
feature_32662	SYNCRIP	2.048823812	0.024668866	1
feature_32715	SYTL3	1.616111511	0.24868185	1
feature_32786	TALDO1	2.055049888	0.011909535	1
feature_32803	TAPT1-AS1	1.538285558	0.028217147	1
feature_32806	TARDBP	1.216308471	0.000546834	1
feature_32836	TASOR2	2.583821651	0.172996343	1
feature_32849	TBC1D1	2.940042158	0.008533268	1
feature_32855	TBC1D14	12.37299242	0.171209169	1
feature_32862	TBC1D22A	2.749257392	0.116103918	1
feature_32875	TBC1D32	1.76865038	0.160559235	1
feature_32887	TBCA	4.203046201	0.168290957	1
feature_32899	TBCK	1.208748235	0.091663342	1
feature_32948	TCERG1	3.11926421	0.144560923	1
feature_32957	TCF25	2.15155407	0.002571225	1
feature_33055	TERT	1.714394572	0.196974238	1
feature_33066	TET3	1.28390587	0.029413935	1
feature_33067	TEX10	1.079334793	0.092346442	1
feature_33088	TEX41	4.65443673	0.207553148	1
feature_33099	TFAM	1.354616308	0.012588009	1
feature_33108	TFB1M	1.047759692	0.106511851	1
feature_33129	TGFB1	1.295468584	0.114053622	1
feature_33136	TGFBR2	3.057892887	0.125492548	1

feature_33153	THADA	2.493098826	0.237536843	1
feature_33198	THOC7	1.560076825	0.183336304	1
feature_33220	THUMPD2	1.040644176	0.014275645	1
feature_33286	TK1	1.296358023	0.031158076	1
feature_33348	TMA7	4.484998512	0.056012272	1
feature_33441	TMEM14B	2.514890093	0.133625237	1
feature_33443	TMEM14C	1.362176544	0.021366145	1
feature_33460	TMEM165	2.070170359	0.178993834	1
feature_33554	TMEM258	1.911850134	0.072630706	1
feature_33674	TMSB10	24.78734378	0.157132164	1
feature_33687	TMTC2	1.347056073	0.203614157	1
feature_33761	TNPO1	1.245215254	0.010017504	1
feature_33770	TNRC6A	1.230984222	0.004699692	1
feature_33793	TOMM20	4.716697493	0.180678928	1
feature_33799	TOMM22	1.308810176	0.02821374	1
feature_33807	TOMM7	3.813026995	0.166183941	1
feature_33860	TPGS2	1.127809244	0.030994159	1
feature_33869	TPM3	4.529025765	0.117348126	1
feature_33880	TPP2	2.041708296	0.099319171	1
feature_33898	TPT1	79.12720329	0.07118004	1
feature_33911	TPTEP1.1	1.294579144	0.126112869	1
feature_33927	TRAF3IP2-AS1	3.140610758	0.016743726	1
feature_34018	TRAPPC9	3.196645444	0.087365696	1
feature_34095	TRIM44	2.518003131	0.085669043	1
feature_34151	TRMT112	1.836247779	0.000950662	1
feature_34292	TTC17	3.798795963	0.098590801	1
feature_34293	TTC19	1.196296083	0.116622309	1
feature_34407	TUBGCP3	1.488476948	0.051551956	1
feature_34411	TUFM	2.247168813	0.065992678	1
feature_34418	TULP4	1.697939942	0.234382884	1
feature_34460	TXNL4A	1.586315289	0.102514726	1
feature_34466	TXNRD1	1.648576052	0.056980752	1
feature_34478	TYW1	1.302584099	0.003050226	1
feature_34504	UBA2	2.816854792	0.059744088	1
feature_34505	UBA3	1.05131745	0.11356097	1
feature_34507	UBA52	21.80950045	0.012983219	1
feature_34516	UBAC2	4.868791642	0.122156185	1
feature_34522	UBAP2	2.034148061	0.03939307	1
feature_34523	UBAP2L	1.037975858	0.111671226	1
feature_34547	UBE2E1	4.6041834	0.031152073	1
feature_34549	UBE2E2	4.667778322	0.216676016	1
feature_34566	UBE2L3	2.190689407	0.021153884	1
feature_34595	UBE2V2	1.055319927	0.082235934	1
feature_34603	UBE3C	2.423722547	0.186884361	1
feature_34652	UCK2	1.939422758	0.117672441	1
feature_34685	UIMC1	1.500039661	0.097363625	1
feature_34748	UQCR10	2.714124533	0.012420589	1
feature_34750	UQCR11	2.022585348	0.184783929	1
feature_34751	UQCRB	6.067311323	0.174501558	1
feature_34756	UQCRFS1	1.51782845	0.15732462	1

feature_34760	UQCRH	6.1727099	0.167484219	1
feature_34764	UQCRQ	2.15199879	0.122413257	1
feature_34771	URI1	2.25250545	0.158456478	1
feature_34787	USP10	1.973221457	0.132236286	1
feature_34793	USP14	1.376407575	0.071282662	1
feature_34816	USP32	2.416607032	0.11345793	1
feature_34823	USP37	1.545401074	0.091122415	1
feature_34862	UTP18	1.014850432	0.04699527	1
feature_34868	UTP6	1.153602989	0.070168444	1
feature_34877	UXS1	1.319483449	0.103866993	1
feature_34904	VAV1	1.18473337	0.002638025	1
feature_34914	VCP	1.107352136	0.014221813	1
feature_34917	VDAC1	2.476644196	0.18579127	1
feature_34926	VDAC2	2.023474787	0.022744643	1
feature_34929	VDAC3	1.043757214	0.088952055	1
feature_34946	VGLL4	1.81890371	0.185382352	1
feature_34950	VIM	12.45659973	0.210618454	1
feature_34961	VKORC1L1	1.278124514	0.097707748	1
feature_34988	VPS13A	1.604993518	0.061902231	1
feature_35011	VPS36	1.875827836	0.121095248	1
feature_35026	VPS54	1.120693728	0.120086833	1
feature_35031	VRK1	2.858213727	0.053193581	1
feature_35032	VRK2	1.098457742	0.014831478	1
feature_35121	WDFY3	1.104239098	0.003127025	1
feature_35125	WDHD1	1.692603305	0.132611172	1
feature_35127	WDR1	1.232318381	0.035673894	1
feature_35142	WDR33	1.680151153	0.025564917	1
feature_35150	WDR43	2.33611276	0.186042351	1
feature_35159	WDR49	1.142484995	0.139163953	1
feature_35173	WDR70	2.389479128	0.055591691	1
feature_35178	WDR76	1.174949536	0.189490281	1
feature_35244	WRN	2.579374454	0.190977707	1
feature_35306	XPO5	1.080668952	0.143615896	1
feature_35307	XPO6	1.299915781	0.250200463	1
feature_35316	XRCC5	6.207842759	0.045696046	1
feature_35317	XRCC6	2.882673312	0.029915654	1
feature_35456	YARS1	1.280348113	0.13194692	1
feature_35459	YBX1	13.21840464	0.148767977	1
feature_35470	YEATS2	1.808230436	0.056364152	1
feature_35516	YWHAG	1.690824427	0.058265011	1
feature_35623	ZC3H15	2.057273487	0.05540116	1
feature_35683	ZEB2	24.13316105	0.094256489	1
feature_35694	ZFAND5	1.103349659	0.070143852	1
feature_35696	ZFAS1	9.517447015	0.211348346	1
feature_35716	ZFP36L2	3.259795646	0.105116521	1
feature_35740	ZFX	1.50226326	0.157045448	1
feature_35780	ZMIZ1	4.599291483	0.076883217	1
feature_35788	ZMYM4	2.711011495	0.037664431	1
feature_35811	ZNF124	1.108686295	0.05985182	1
feature_35957	ZNF33A	1.314146812	0.140186693	1

feature_35979	ZNF37BP	1.293244985	0.185336412	1
feature_36014	ZNF43	1.608106556	0.108997149	1
feature_36016	ZNF431	1.132256441	0.088688687	1
feature_36175	ZNF609	4.831435184	0.004144812	1
feature_36261	ZNF706	1.752640469	0.021855303	1
feature_36273	ZNF718	1.778878934	0.107768256	1
feature_36304	ZNF767P.1	1.048649131	0.096466079	1
feature_36357	ZNF83	1.211861274	0.077697861	1
feature_36393	ZNF91	1.115357091	0.1385287	1
feature_36428	ZRANB2	3.287812989	0.184953595	1
feature_36431	ZRANB3	1.932307242	0.142547388	1
feature_36470	ZXDC	1.08422671	0.105357795	1
feature_36475	ZZZ3	2.016803991	0.098342584	1

Table S5: Genes upregulated in cluster 14

FeatureID	FeatureName	Cluster 14 Average	Cluster 14 Log2 Fold Change	Cluster 14 P-V
feature_22588	LRMDA	1.544089036	3.352647402	8.90E-16
feature_23141	MCTP1	1.488942999	2.221058373	1.11E-06
feature_27387	PRSS12	1.382327327	2.194151808	1.59E-06
feature_35284	XIST	5.936164516	1.932298104	7.83E-05
feature_2987	CD109	1.511001414	1.823034355	0.00048371
feature_35702	ZFHX3	3.58204147	1.652711354	0.00346419
feature_4590	DAZL	1.209536411	1.574182417	0.00993703
feature_22900	MAML2	1.36639625	1.566176793	0.01083175
feature_1594	ATXN1	6.247433258	1.470329616	0.02105457
feature_19130	HMGA2	4.018307896	1.462816359	0.0231635
feature_26622	PLD1	5.111424896	1.327433857	0.06668795
feature_26711	PLXDC2	3.30876222	1.324609314	0.07099316
feature_33214	THSD7A	3.411701489	1.29915389	0.08346567
feature_30678	SEL1L3	1.569823853	1.278960561	0.1033144
feature_18712	H1-5	3.833262305	1.208041137	0.15805602
feature_35659	ZDHHC14	3.780567203	1.20696489	0.16298935
feature_1077	ARHGAP10	1.116400882	1.210164576	0.17023224
feature_18776	H3C2	2.218096155	1.189113031	0.18259134
feature_18720	H2AC16	1.529383426	1.250242586	0.19029468
feature_31899	SNTB1	1.91295475	1.155654964	0.22658854
feature_19706	IGF2BP2	5.334459979	1.118637342	0.25731215
feature_32503	STS	2.850437379	1.057866881	0.36103999
feature_17516	FMNL2	8.991254965	1.033857191	0.39542548
feature_27618	PTPN14	2.213194285	1.033840104	0.41145919
feature_25820	PAM	1.215663749	1.035153994	0.41544177
feature_18743	H2BC11	1.718105419	1.006935473	0.47258196
feature_25204	NRIP1	3.406799619	0.996386453	0.47792167
feature_18715	H2AC11	1.958297047	0.987796588	0.48990418
feature_14759	ENSG0000028	27.22866213	0.978541645	0.49251613
feature_18724	H2AC20	1.579627593	0.972018007	0.52402553
feature_18709	H1-2	6.829530315	0.957048255	0.53733637
feature_18729	H2AC6	1.807564546	0.943203988	0.5982578
feature_18716	H2AC12	1.411738547	0.936779879	0.60458697
feature_34134	TRIO	1.199732671	0.926019526	0.63245579
feature_32666	SYNE2	2.227899895	0.913281552	0.64716804
feature_24757	NEGR1	34.38539227	0.898226834	0.67188636
feature_25637	OSBPL3	1.431346027	0.904581993	0.67981881
feature_5279	DTNA	2.911710753	0.873144402	0.74645534
feature_1581	ATP8B4	7.057467268	0.842819892	0.82256459
feature_7477	ENSG0000023	6.428802446	0.838495439	0.83459159
feature_34855	UST	1.298995538	0.84636428	0.84137457
feature_696	ALOX5AP	1.666635785	0.813764972	0.90404241
feature_32928	TBXAS1	2.143342638	0.782440831	0.96991958
feature_26571	PLA2G4A	1.08821513	0.721018419	0.98480627
feature_31971	SORBS2	2.448484043	0.777016105	0.99848834
feature_20	AAK1	3.189891873	0.252899087	1
feature_71	ABCC4	1.074734988	0.184668672	1

feature_109	ABHD17B	2.575932661	0.162199807	1
feature_111	ABHD18	1.235271229	0.147509713	1
feature_113	ABHD3	2.022021356	0.068571155	1
feature_118	ABI1	3.008522685	0.23340435	1
feature_124	ABL1	1.658057512	0.244716111	1
feature_125	ABL2	1.031843626	0.169877194	1
feature_151	ACADM	1.37129812	6.21E-05	1
feature_157	ACAP2	3.7768908	0.034088811	1
feature_176	ACER3	1.210761879	0.463945741	1
feature_178	ACIN1	1.672763122	0.008558318	1
feature_217	ACSL4	1.08821513	0.079858122	1
feature_272	ACTR2	4.995005484	0.049056801	1
feature_293	ACYP2	1.476688324	0.159700948	1
feature_294	ADA	1.887219933	0.059724301	1
feature_302	ADAM17	2.12741156	0.085187974	1
feature_353	ADAR	1.439924299	0.098681128	1
feature_376	ADD1	1.874965258	0.249015141	1
feature_378	ADD3	1.183801594	0.096645425	1
feature_390	ADGRE2	3.558757587	0.359286898	1
feature_392	ADGRE5	3.046512177	0.356722303	1
feature_428	ADNP	3.378613866	0.026429948	1
feature_461	AFDN	2.487699002	0.123282798	1
feature_463	AFF1	16.01563461	0.371195908	1
feature_467	AFF4	2.417847355	0.144871753	1
feature_474	AFTPH	1.154390374	0.126127228	1
feature_509	AGO2	2.786713069	0.192662698	1
feature_511	AGO4	1.655606577	0.366496873	1
feature_523	AGTPBP1	1.40070934	0.120903039	1
feature_527	AHCTF1	1.791633469	0.191264465	1
feature_530	AHCYL1	1.31247568	0.041530405	1
feature_531	AHCYL2	1.116400882	0.081060727	1
feature_533	AHDC1	1.104146207	0.435558644	1
feature_548	AIF1	4.256048588	0.255746928	1
feature_567	AK2	1.466884584	0.11983415	1
feature_579	AKAP10	1.642126435	0.178567897	1
feature_582	AKAP13	7.409176437	0.250997909	1
feature_592	AKAP8L	1.13233196	0.010191369	1
feature_597	AKNA	1.047774703	0.166553814	1
feature_606	AKR7A2	1.111499012	0.006943571	1
feature_685	ALMS1	1.001206938	0.165015562	1
feature_710	AMBRA1	2.372505058	0.108409	1
feature_737	AMZ1	3.857771655	0.468718525	1
feature_745	ANAPC11	2.275693127	0.069799226	1
feature_754	ANAPC5	2.397014408	0.098538649	1
feature_784	ANKIB1	1.996286539	0.099195846	1
feature_786	ANKLE2	1.335759563	0.164544596	1
feature_787	ANKMY1	2.133538898	0.361073377	1
feature_792	ANKRD11	5.183727477	0.038731891	1
feature_794	ANKRD12	1.200958139	0.085911499	1
feature_798	ANKRD13D	1.196056269	0.413562717	1

feature_814	ANKRD26	1.237722164	0.110499742	1
feature_818	ANKRD28	5.747442522	0.231955509	1
feature_840	ANKRD44	2.677646463	0.324023409	1
feature_861	ANKS1A	3.073472462	0.457770401	1
feature_868	ANLN	1.062480313	0.34149707	1
feature_876	ANO6	1.63477363	0.140723303	1
feature_901	ANXA2	1.757320379	0.185515232	1
feature_906	ANXA2R-AS1	1.12252822	0.010830061	1
feature_919	AOPEP	2.138440768	0.219776578	1
feature_941	AP2S1	1.514677816	0.113668499	1
feature_942	AP3B1	2.904357948	0.098192094	1
feature_958	APAF1	1.084538728	0.404331532	1
feature_959	APBA1	1.991384669	0.693204908	1
feature_963	APBB1IP	7.531723186	0.058281968	1
feature_966	APC	1.383552795	0.165996344	1
feature_980	APIP	1.001206938	0.127883912	1
feature_1064	ARFGEF1	1.944816905	0.053021824	1
feature_1066	ARFGEF2	1.647028305	0.229006748	1
feature_1075	ARGLU1	4.726628104	0.078285589	1
feature_1084	ARHGAP15	9.302523707	0.111435612	1
feature_1087	ARHGAP18	1.084538728	0.321191054	1
feature_1095	ARHGAP25	3.7768908	0.441729168	1
feature_1108	ARHGAP35	1.710752614	0.174483966	1
feature_1111	ARHGAP4	2.129862495	0.097492817	1
feature_1130	ARHGEF12	2.16172465	0.157190694	1
feature_1135	ARHGEF18	5.127355973	0.260377055	1
feature_1145	ARHGEF3	2.140891703	0.750891205	1
feature_1158	ARHGEF6	1.958297047	0.261861876	1
feature_1159	ARHGEF7	1.33698503	0.020441262	1
feature_1163	ARID1A	3.371261062	0.148772035	1
feature_1164	ARID1B	42.74185507	0.171505697	1
feature_1165	ARID2	4.691089547	0.01789343	1
feature_1169	ARID4A	1.677664992	0.172678694	1
feature_1172	ARID5B	4.399428285	0.007212773	1
feature_1186	ARL15	3.56243399	0.135140816	1
feature_1214	ARL6IP5	3.039159372	0.157678908	1
feature_1215	ARL6IP6	1.38110186	0.188384802	1
feature_1217	ARL8B	1.642126435	0.302077353	1
feature_1223	ARMC2	1.113949947	0.38836295	1
feature_1242	ARMH3	1.567372918	0.153463364	1
feature_1252	ARPC3	7.128544382	0.009792853	1
feature_1260	ARPC5	2.814898822	0.031407063	1
feature_1301	ASAP1	5.720482237	0.152537433	1
feature_1303	ASAP2	2.216870687	0.344136753	1
feature_1325	ASCC3	3.184990003	0.072584823	1
feature_1334	ASH1L	5.258480994	0.072118163	1
feature_1337	ASH2L	1.191154399	0.369360824	1
feature_1376	ASXL2	1.724232757	0.061049399	1
feature_1381	ATAD2	5.231520709	0.315893321	1
feature_1382	ATAD2B	2.243830972	0.09552024	1

feature_1386	ATAD5	2.95215118	0.308382479	1
feature_1389	ATE1	1.382327327	0.12420293	1
feature_1398	ATF6	1.64947924	0.173182638	1
feature_1403	ATF7IP	3.436210838	0.057729138	1
feature_1404	ATF7IP2	2.037952434	0.070977199	1
feature_1405	ATG10	1.792858936	0.362793653	1
feature_1417	ATG3	1.019588951	0.07007204	1
feature_1424	ATG7	3.218077625	0.321829506	1
feature_1431	ATM	2.628627763	0.15536675	1
feature_1436	ATOSA	1.811240948	0.026954878	1
feature_1444	ATP11A	1.812466416	0.206251158	1
feature_1447	ATP11B	1.637224565	0.023550037	1
feature_1452	ATP13A2	1.214438281	0.115847139	1
feature_1470	ATP2A2	1.509775946	0.030669466	1
feature_1472	ATP2B1	2.399465343	0.055289328	1
feature_1476	ATP2B4	1.471786454	0.160849199	1
feature_1477	ATP2C1	1.128655557	0.254148341	1
feature_1546	ATP6V0D1	1.325955823	0.287184867	1
feature_1549	ATP6V0E1	1.970551722	0.048321276	1
feature_1569	ATP6V1H	1.444826169	0.275273313	1
feature_1572	ATP8A1	2.088196601	0.264735427	1
feature_1584	ATP9B	1.536736231	0.21721711	1
feature_1588	ATR	2.408043615	0.056507539	1
feature_1591	ATRN	1.479139259	0.197810802	1
feature_1600	ATXN2L	1.302671941	0.164787308	1
feature_1602	ATXN7	1.427669624	0.229478717	1
feature_1603	ATXN7L1	1.638450032	0.242787225	1
feature_1615	AURKB	1.439924299	0.396005855	1
feature_1617	AUTS2	19.185919	0.386838673	1
feature_1620	AVL9	1.07841139	0.029405774	1
feature_1653	B3GNT2	1.031843626	0.062581449	1
feature_1666	B4GALT1	1.401934807	0.334390786	1
feature_1684	BABAM2	2.808771484	0.247602049	1
feature_1702	BAHCC1	2.640882438	0.200335558	1
feature_1710	BALR6	8.121173048	0.318836625	1
feature_1730	BAX	1.827172026	0.305481698	1
feature_1735	BAZ2B	4.160462124	0.155019229	1
feature_1752	BBX	3.258518053	0.098620384	1
feature_1764	BCAS3	2.71808689	0.23249549	1
feature_1766	BCAT1	3.868800862	0.161564414	1
feature_1773	BCKDHB	1.85903418	0.015191947	1
feature_1786	BCL2L13	1.015912548	0.266864197	1
feature_1794	BCL7A	5.952095593	0.123633189	1
feature_1808	BCR	3.003620815	0.18178726	1
feature_1867	BICRAL	2.085745666	0.198630269	1
feature_1886	BLM	1.833299363	0.218190182	1
feature_1888	BLNK	2.741370772	0.730927529	1
feature_1897	BLTP1	1.115175415	0.117614398	1
feature_1898	BLTP2	1.041647365	0.099637319	1
feature_1914	BMP2K	2.547746909	0.348583919	1

feature_1961	BOD1L1	1.92766036	0.13327857	1
feature_1996	BPTF	6.828304847	0.036273435	1
feature_2001	BRCA1	5.035445911	0.46482965	1
feature_2002	BRCA2	3.121265694	0.410699835	1
feature_2005	BRD1	1.13723383	0.471946258	1
feature_2010	BRD7	1.905601945	0.040980849	1
feature_2020	BRI3	1.283064461	0.034277619	1
feature_2021	BRI3BP	1.786731599	0.074243961	1
feature_2028	BRIP1	4.751137454	0.507765979	1
feature_2040	BRWD1	2.878623131	0.061858065	1
feature_2054	BST2	1.901925543	0.18171341	1
feature_2069	BTBD7	1.08331326	0.030884132	1
feature_2072	BTBD9	3.575914132	0.197212454	1
feature_2109	BTRC	1.009785211	0.074152442	1
feature_2111	BUB1B	1.12743009	0.141539289	1
feature_2118	BUD31	1.066156715	0.061086761	1
feature_2186	C16orf74	4.57712107	0.10346223	1
feature_2283	C1QTNF3-AM	1.147037569	0.087011	1
feature_2302	C21orf58	1.737712899	0.657873229	1
feature_2316	C2CD5	1.13723383	0.355821392	1
feature_2397	C6orf62	1.423993222	0.058381229	1
feature_2449	CAB39	2.372505058	0.132596165	1
feature_2452	CABIN1	1.113949947	0.269761636	1
feature_2478	CACNA2D4	1.279388058	0.112064959	1
feature_2482	CACNB4	8.657927808	0.762895263	1
feature_2508	CALCB	1.537961698	0.431017079	1
feature_2540	CAMK2D	4.730304507	0.170354696	1
feature_2596	CAPZB	3.044061242	0.0808057	1
feature_2605	CARD8	1.509775946	0.239630676	1
feature_2624	CARS2	1.012236146	0.063321588	1
feature_2653	CASP8AP2	1.671537655	0.083638603	1
feature_2687	CBFA2T2	1.091891532	0.211763907	1
feature_2688	CBFA2T3	1.067382183	0.292860248	1
feature_2690	CBL	1.175223322	0.199248697	1
feature_2691	CBLB	1.305122876	0.101521023	1
feature_2702	CBR4	1.08331326	0.064263694	1
feature_2716	CBX4	1.024490821	0.41632783	1
feature_2717	CBX5	3.507287953	0.009480049	1
feature_2741	CCDC12	1.372523587	0.025430356	1
feature_2756	CCDC14	2.324711826	0.287370288	1
feature_2790	CCDC18	2.121284223	0.225940668	1
feature_2791	CCDC18-AS1	1.050225638	0.010606728	1
feature_2814	CCDC26	18.5376467	0.577311709	1
feature_2862	CCDC88A	5.770726405	0.003259935	1
feature_2870	CCDC91	3.307536752	0.03939034	1
feature_2898	CCM2	2.276918594	0.297246572	1
feature_2931	CCNL1	2.623725893	0.03132748	1
feature_2941	CCNY	2.302653411	0.209032022	1
feature_2994	CD164	4.649423652	0.065011159	1
feature_3020	CD2AP	1.32718129	0.004779523	1

feature_3038	CD38	2.262212984	0.013657812	1
feature_3048	CD46	1.07841139	0.05339202	1
feature_3081	CD96	5.001132822	0.046553322	1
feature_3082	CD99	3.339398907	0.451418324	1
feature_3089	CDC14A	1.477913791	0.142696538	1
feature_3102	CDC27	1.982806397	0.087553858	1
feature_3125	CDC42SE2	2.377406928	0.057688757	1
feature_3126	CDC45	1.340661433	0.165923077	1
feature_3129	CDC7	1.095567935	0.265055058	1
feature_3130	CDC73	1.676439525	0.040144956	1
feature_3135	CDCA5	1.042872833	0.421385642	1
feature_3176	CDK12	1.810015481	0.205832588	1
feature_3184	CDK19	4.154334787	0.047053344	1
feature_3195	CDK5RAP2	2.865142989	0.375652602	1
feature_3198	CDK6-AS1	16.30852134	0.166511288	1
feature_3204	CDKAL1	6.379783746	0.05123409	1
feature_3210	CDKN1A	1.162968647	0.553865211	1
feature_3219	CDKN2D	1.245074969	0.057624599	1
feature_3234	CDT1	1.318603018	0.177041969	1
feature_3241	CDYL	2.984013335	0.239618568	1
feature_3270	CELF1	3.669049661	0.068793057	1
feature_3271	CELF2	6.997419361	0.031120242	1
feature_3292	CENPC	1.61516615	0.23566848	1
feature_3296	CENPH	1.504874076	0.019381005	1
feature_3299	CENPK	2.67397006	0.233057916	1
feature_3301	CENPM	1.11762635	0.17366501	1
feature_3306	CENPP	8.184897357	0.309259026	1
feature_3311	CENPU	1.835750298	0.167789642	1
feature_3321	CEP128	3.881055537	0.323173104	1
feature_3323	CEP135	1.86393605	0.092471773	1
feature_3324	CEP152	1.215663749	0.115294447	1
feature_3325	CEP162	1.530608893	0.231795312	1
feature_3332	CEP192	2.74749811	0.256901466	1
feature_3345	CEP57	2.058785381	0.138903365	1
feature_3346	CEP57L1	1.334534095	0.204902018	1
feature_3357	CEP85L	2.084520198	0.662879141	1
feature_3377	CERT1	1.350465173	0.000351638	1
feature_3437	CFDP1	1.638450032	0.003060192	1
feature_3452	CGAS	1.303897408	0.192081951	1
feature_3463	CHAF1A	2.078392861	0.09698972	1
feature_3488	CHD2	3.097981812	0.049917919	1
feature_3492	CHD6	2.316133554	0.104680793	1
feature_3493	CHD7	2.887201403	0.296435859	1
feature_3494	CHD8	1.075960455	0.046311007	1
feature_3495	CHD9	5.520731037	0.130488634	1
feature_3524	CHMP2A	1.014687081	0.010506199	1
feature_3550	CHRAC1	1.051451105	0.063024906	1
feature_3576	CHST11	8.238817927	0.248173948	1
feature_3580	CHST15	2.187459467	0.599229835	1
feature_3597	CIAO1	1.055127508	0.005238536	1

feature_3622	CIP2A	1.023265353	0.188620695	1
feature_3626	CIRBP	4.469279931	0.127144253	1
feature_3634	CIT	2.324711826	0.289450129	1
feature_3645	CKAP5	1.758545846	0.004568917	1
feature_3656	CLASP1	2.841859106	0.153252591	1
feature_3658	CLASP2	6.806246433	0.33660484	1
feature_3700	CLEC16A	1.719330887	0.231703038	1
feature_3725	CLECL1P	2.720537825	0.017532528	1
feature_3732	CLIC4	1.67398859	0.546813244	1
feature_3738	CLINT1	1.892121803	0.091818259	1
feature_3739	CLIP1	2.144568105	0.238944477	1
feature_3741	CLIP2	3.563659457	0.083503689	1
feature_3744	CLK1	2.383534266	0.126961819	1
feature_3759	CLNS1A	2.252409244	0.045762848	1
feature_3772	CLSPN	1.796535339	0.295241009	1
feature_3776	CLTA	2.69847941	0.029116657	1
feature_3778	CLTC	1.711978082	0.033170943	1
feature_3793	CMC1	1.258555111	0.122560317	1
feature_3796	CMIP	1.850455908	0.264646762	1
feature_3843	CNOT1	1.941140502	0.080163588	1
feature_3844	CNOT10	1.252427774	0.121983928	1
feature_3849	CNOT4	1.604136943	0.030418423	1
feature_3850	CNOT6	1.209536411	0.091924368	1
feature_3854	CNOT8	1.003657873	0.050417718	1
feature_3865	CNST	1.186252529	0.220767754	1
feature_3869	CNTLN	2.617598556	0.135196784	1
feature_3887	CNTRL	4.65064912	0.146691621	1
feature_3889	COA1	1.588205865	0.138482825	1
feature_3963	COLGALT1	1.047774703	0.005380781	1
feature_3967	COMMD1	2.16172465	0.11322132	1
feature_3979	COMT	3.253616183	0.037096456	1
feature_3981	COP1	4.11389436	0.003794197	1
feature_3984	COPA	1.583303995	0.076196169	1
feature_3985	COPB1	1.109048077	0.025171566	1
feature_4023	CORO1C	1.443600702	0.184092689	1
feature_4104	CPLANE1	1.247525904	0.130695892	1
feature_4122	CPNE8	9.204486308	0.463836176	1
feature_4129	CPQ	1.397032937	0.414581983	1
feature_4153	CRACR2A	1.432571494	0.14270075	1
feature_4155	CRADD	2.267114854	0.473044358	1
feature_4164	CREB1	2.351672111	0.207801011	1
feature_4172	CREBBP	3.24993978	0.053301896	1
feature_4183	CRIM1	1.958297047	0.383936269	1
feature_4198	CRLF3	1.528157958	0.077339171	1
feature_4216	CRTC3	1.159292244	0.096161005	1
feature_4245	CSE1L	1.93256223	0.172845707	1
feature_4255	CSK	1.401934807	0.19956714	1
feature_4257	CSMD1	1.692370602	0.736467194	1
feature_4296	CSTF3	1.046549235	0.117939742	1
feature_4299	CSTPP1	1.780604261	0.103818081	1

feature_4315	CTBP2	4.593052148	0.29136475	1
feature_4321	CTCF	2.586961869	0.150564204	1
feature_4330	CTDSPL2	2.102902211	0.050860865	1
feature_4339	CTNNA1	1.002432406	0.080542215	1
feature_4386	CUL1	1.372523587	0.079663479	1
feature_4398	CUX1	8.328277054	0.017102784	1
feature_4444	CYB5R4	1.661733915	0.025778152	1
feature_4446	CYBA	10.2375554	0.056638145	1
feature_4534	CYRIA	2.520786624	0.155549596	1
feature_4541	CYTH1	1.8884454	0.244562685	1
feature_4545	CYTIP	1.321053953	0.184984022	1
feature_4563	DAD1	7.168984809	0.015180647	1
feature_4589	DAZAP2	2.240154569	0.116820215	1
feature_4597	DBI	2.915387156	0.042153741	1
feature_4604	DBNL	1.079636858	0.082156282	1
feature_4610	DCAF10	1.051451105	0.124351729	1
feature_4644	DCLRE1C	1.311250213	0.060343008	1
feature_4647	DCP1A	1.147037569	0.031688052	1
feature_4660	DCTN4	1.635999097	0.404890313	1
feature_4678	DDB2	1.37129812	0.497422002	1
feature_4680	DDHD1	1.588205865	0.120788039	1
feature_4697	DDT	1.935013165	0.141552501	1
feature_4709	DDX17	5.48519248	0.103286326	1
feature_4724	DDX39A	1.727909159	0.153018767	1
feature_4729	DDX3X	2.562452519	0.104948792	1
feature_4733	DDX42	1.32227942	0.042070472	1
feature_4739	DDX50	1.298995538	0.033542364	1
feature_4755	DECR1	1.425218689	0.251553074	1
feature_4774	DENND1A	2.360250383	0.321771123	1
feature_4782	DENND3	1.401934807	0.457208762	1
feature_4786	DENND4C	1.07350952	0.192490302	1
feature_4787	DENND5A	1.520805154	0.060082355	1
feature_4790	DENND6A	1.729134627	0.139778046	1
feature_4806	DERA	1.403160275	0.201271597	1
feature_4832	DGKD	2.528139429	0.232055935	1
feature_4850	DHFR	2.665391788	0.03583898	1
feature_4871	DHRSX	1.899474608	0.575763453	1
feature_4873	DHTKD1	1.256104176	0.258724934	1
feature_4898	DIAPH2	6.148170391	0.292312244	1
feature_4900	DIAPH3	4.317321963	0.455523124	1
feature_4903	DICER1	1.718105419	0.166148441	1
feature_4905	DIDO1	1.58575493	0.306107489	1
feature_4914	DIP2B	2.905583416	0.070523619	1
feature_4929	DIS3L2	2.23035083	0.179805539	1
feature_4930	DISC1	1.469335519	0.143726699	1
feature_4933	DISP1	1.042872833	0.428976689	1
feature_4949	DLEU2	9.287818097	0.190122544	1
feature_4990	DMC1	1.151939439	0.339150194	1
feature_5003	DMXL1	1.095567935	0.184949106	1
feature_5006	DNA2	1.109048077	0.128974442	1

feature_5018	DNAAF9	2.860241119	0.203469234	1
feature_5063	DNAJC1	2.265889387	0.045323722	1
feature_5064	DNAJC10	1.05880391	0.453730535	1
feature_5100	DNAJC9	1.209536411	0.003488621	1
feature_5117	DNM1L	1.506099544	0.077917951	1
feature_5120	DNM2	2.850437379	0.085141002	1
feature_5126	DNMT1	3.893310212	0.106379054	1
feature_5134	DNTT	1.726683692	0.152545416	1
feature_5141	DOCK10	3.301409415	0.207018177	1
feature_5142	DOCK11	2.345544773	0.220757287	1
feature_5144	DOCK2	3.745028646	0.233996202	1
feature_5150	DOCK7	1.047774703	0.212226158	1
feature_5152	DOCK8	5.032994976	0.229853739	1
feature_5160	DOK3	1.221791086	0.15554664	1
feature_5168	DOP1A	1.263456981	0.310880154	1
feature_5198	DPP8	1.442375234	0.300387983	1
feature_5223	DPYD	4.218059096	0.548988978	1
feature_5258	DSE	1.567372918	0.474675714	1
feature_5280	DTNB	1.492619401	0.069969962	1
feature_5282	DTNBP1	2.764654655	0.138075793	1
feature_5321	DUSP6	1.60046054	0.051879299	1
feature_5325	DUT	8.135878658	0.030437622	1
feature_5341	DYNC1H1	2.075941926	0.032370812	1
feature_5361	DYNLT1	1.272035253	0.04675823	1
feature_5381	E2F3	3.629834702	0.145396322	1
feature_5395	EAF2	1.829622961	0.274766534	1
feature_5400	EBF1	41.69162943	0.172847517	1
feature_5452	EEA1	1.665410317	0.011337461	1
feature_5511	EEF2	11.30126118	0.085046158	1
feature_5525	EFCAB2	2.74259624	0.281524048	1
feature_5553	EFR3A	1.372523587	0.147053673	1
feature_5555	EFTUD2	1.229143891	0.067953774	1
feature_5585	EHMT1	2.040403369	0.05430429	1
feature_5595	EIF1AX	3.292831142	0.073847305	1
feature_5610	EIF2AK2	1.602911475	0.20546853	1
feature_5613	EIF2AK4	1.323504888	0.048793058	1
feature_5629	EIF2S3	3.903113952	0.303878578	1
feature_5649	EIF3L	3.599198014	0.016708866	1
feature_5668	EIF4A3	1.764673184	0.433429008	1
feature_5690	EIF4G3	7.091780357	0.05796809	1
feature_5704	EIPR1	1.020814418	0.07172306	1
feature_5716	ELF2	3.785469073	0.147753177	1
feature_5731	ELMO1	3.958259989	0.381293928	1
feature_5769	ELP4	1.362719847	0.030776462	1
feature_5773	EMB	5.265833799	0.000773469	1
feature_5807	EMSY	1.268358851	0.096050803	1
feature_5853	ENSA	2.828378964	0.109001489	1
feature_6406	ENSG0000022	1.357817978	0.429859575	1
feature_6713	ENSG0000022	1.161743179	0.42539553	1
feature_6722	ENSG0000022	4.238892044	0.585230738	1

feature_8938	ENSG0000025	1.810015481	0.068873188	1
feature_11130	ENSG0000026	1.284289928	0.161297064	1
feature_12319	ENSG0000027	1.249976839	0.253303891	1
feature_14231	ENSG0000028	1.252427774	0.041545751	1
feature_15452	ENSG0000028	1.832073896	0.444749548	1
feature_15537	ENSG0000028	1.154390374	0.576683507	1
feature_15835	ENSG0000028	2.623725893	0.211448248	1
feature_15836	ENSG0000028	1.578402126	0.379973953	1
feature_15928	ENSG0000028	1.705850744	0.370133952	1
feature_16455	ENSG0000029	1.113949947	0.110981676	1
feature_16569	ENSG0000029	1.329632225	0.095605808	1
feature_16615	ENTPD1-AS1	1.564921983	0.242595935	1
feature_16619	ENTPD4	1.175223322	0.333058125	1
feature_16638	EP300	1.939915035	0.002256517	1
feature_16640	EP400	1.943591437	0.252991468	1
feature_16692	EPS15	2.208292415	0.105361871	1
feature_16711	ERC1	3.112687421	0.1396902	1
feature_16713	ERCC1	1.87373979	0.283682892	1
feature_16720	ERCC6L2	2.555099714	0.336382374	1
feature_16721	ERCC6L2-AS1	1.020814418	0.588214285	1
feature_16727	ERG	4.847949385	0.443033571	1
feature_16762	ERP44	1.352916108	0.1021643	1
feature_16798	ETF1	1.933787697	0.009040643	1
feature_16802	ETFA	2.622500426	0.016960887	1
feature_16808	ETNK1	1.433796962	0.073102648	1
feature_16819	ETV6	8.127300385	0.449859973	1
feature_16832	EVI5	1.62987176	0.113444278	1
feature_16845	EXOC2	1.454629909	0.164487873	1
feature_16851	EXOC4	5.102846623	0.04814739	1
feature_16854	EXOC6	2.033050564	0.155513063	1
feature_16881	EYA4	1.899474608	0.464734396	1
feature_16928	FAF1	4.552611721	0.02786449	1
feature_16930	FAF2	1.406836677	0.279746862	1
feature_16952	FAM111A	2.093098471	0.522805655	1
feature_16976	FAM135A	2.264663919	0.042177068	1
feature_17010	FAM168A	1.35169064	0.111796324	1
feature_17019	FAM172A	2.40069081	0.104428717	1
feature_17041	FAM193A	1.452178974	0.050047266	1
feature_17047	FAM204A	1.301446473	0.002122881	1
feature_17172	FANCA	1.574725723	0.325509773	1
feature_17174	FANCC	1.661733915	0.364468196	1
feature_17175	FANCD2	1.954620645	0.384748278	1
feature_17181	FANCI	2.388436136	0.267904395	1
feature_17186	FAR1	1.188703464	0.122855706	1
feature_17188	FAR2	1.285515396	0.509937953	1
feature_17193	FARS2	2.94724931	0.098115961	1
feature_17237	FBXL17	2.938671038	0.060606565	1
feature_17245	FBXL4	1.285515396	0.14915486	1
feature_17246	FBXL5	1.237722164	0.321779458	1
feature_17267	FBXO34	1.090666065	0.034499495	1

feature_17276	FBXO42	1.12743009	0.078128601	1
feature_17283	FBXO5	1.03919643	0.100798497	1
feature_17290	FBXW11	2.041628836	0.083129379	1
feature_17293	FBXW4	1.281838993	0.245356512	1
feature_17296	FBXW7	3.911692224	0.026396802	1
feature_17323	FCHSD2	4.610208693	0.09006378	1
feature_17354	FER	1.134782895	0.303956779	1
feature_17362	FERMT3	1.751193041	0.066564143	1
feature_17376	FGD3	1.08331326	0.310741229	1
feature_17463	FKBP5	2.816124289	0.494481925	1
feature_17477	FLI1	5.048926054	0.240540279	1
feature_17500	FLT3	17.40899115	0.209035426	1
feature_17534	FNBP1	3.596747079	0.154913496	1
feature_17541	FNDC3A	3.236459638	0.226190371	1
feature_17542	FNDC3B	1.753643976	0.328962522	1
feature_17560	FOSB	8.664055145	0.412415843	1
feature_17579	FOXJ3	2.1372153	0.066190495	1
feature_17581	FOXK2	2.438680303	0.212160506	1
feature_17586	FOXN3	2.561227051	0.299848699	1
feature_17589	FOXO1	2.571030791	0.133849996	1
feature_17590	FOXO3	3.504837018	0.168979804	1
feature_17606	FPGS	1.466884584	0.060482955	1
feature_17646	FRS2	1.140910232	0.153666881	1
feature_17650	FRYL	3.594296145	0.003396488	1
feature_17696	FTO	3.420279761	0.278398228	1
feature_17700	FTX	5.236422579	0.302557073	1
feature_17711	FUS	6.518261573	0.079662626	1
feature_17764	GAB1	3.732773971	0.458684576	1
feature_17765	GAB2	7.183690419	0.117597881	1
feature_17811	GAK	1.057578443	0.18338545	1
feature_17833	GALNT2	9.956923346	0.292694343	1
feature_17838	GALNT7	4.295263548	0.125452644	1
feature_17851	GANAB	1.07841139	0.086083283	1
feature_17910	GAS7	8.058674206	0.724703479	1
feature_17924	GATAD2A	2.202165077	0.084513336	1
feature_17925	GATAD2B	1.594333203	0.052394216	1
feature_17937	GBE1	1.055127508	0.231251273	1
feature_17938	GBF1	2.104127678	0.225444988	1
feature_17956	GCC2	1.67889046	0.2454614	1
feature_18017	GEN1	1.798986273	0.319645585	1
feature_18039	GGA2	1.388454665	0.148245341	1
feature_18069	GIGYF2	2.409269083	0.11958363	1
feature_18080	GINS1	1.079636858	0.256423656	1
feature_18090	GIT2	1.786731599	0.250402104	1
feature_18113	GLA	1.046549235	0.185962049	1
feature_18158	GLS	1.762222249	0.005014103	1
feature_18178	GLYR1	1.632322695	0.178088706	1
feature_18183	GMDS-DT	4.450897919	0.556208978	1
feature_18192	GMNN	2.403141745	0.051827036	1
feature_18197	GMPS	1.398258405	0.032206279	1

feature_18200	GNA12	3.501160615	0.262476298	1
feature_18204	GNA15	2.566128921	0.099930912	1
feature_18215	GNAS	8.907923176	0.075891984	1
feature_18224	GNB2	1.254878708	0.052997264	1
feature_18226	GNB4	1.533059828	0.035507619	1
feature_18255	GNPTAB	2.819800692	0.361287386	1
feature_18267	GOLGA4	2.155597313	0.019093185	1
feature_18300	GOLIM4	1.061254845	0.26335152	1
feature_18304	GOLPH3	2.143342638	0.002069076	1
feature_18309	GON4L	1.502423141	0.162180678	1
feature_18342	GPATCH2	1.085764195	0.065649677	1
feature_18343	GPATCH2L	1.298995538	0.10742389	1
feature_18346	GPATCH8	3.675176999	0.11121135	1
feature_18349	GPBP1L1	1.900700075	0.06523571	1
feature_18358	GPCPD1	1.408062145	0.160305976	1
feature_18361	GPD2	1.08331326	0.148356411	1
feature_18369	GPM6B	3.198470146	0.583347959	1
feature_18485	GRB10	2.259762049	0.3665768	1
feature_18487	GRB2	3.867575394	0.147812946	1
feature_18527	GRK2	2.105353146	0.186553319	1
feature_18566	GSE1	1.051451105	0.27074512	1
feature_18587	GSTK1	1.091891532	0.021692515	1
feature_18606	GTF2F1	1.274486188	0.208926577	1
feature_18651	GTSE1	1.464433649	0.267212069	1
feature_18659	GUCY1A1	2.571030791	0.24876136	1
feature_18694	GYPC	1.167870517	0.479920734	1
feature_18706	H1-10	5.032994976	0.033515739	1
feature_18710	H1-3	5.952095593	0.628293202	1
feature_18711	H1-4	2.256085647	0.75829415	1
feature_18810	H4C3	31.5570133	0.721779303	1
feature_18830	HADHB	1.085764195	0.193078453	1
feature_18853	HAUS1	1.215663749	0.077560954	1
feature_18897	HCLS1	1.689919667	0.022429241	1
feature_18916	HDAC4	1.635999097	0.401878685	1
feature_18922	HDAC9	13.42499634	0.281910867	1
feature_18934	HDLBP	1.724232757	0.091372094	1
feature_18948	HECA	1.814917351	0.151667206	1
feature_18961	HELLS	3.002395347	0.161630181	1
feature_18963	HELZ	2.44970951	0.145828732	1
feature_18984	HERC4	2.550197844	0.304637269	1
feature_18999	HEXB	1.194830802	0.057303005	1
feature_19027	HIBADH	1.095567935	0.133318539	1
feature_19028	HIBCH	1.06370578	0.17531117	1
feature_19033	HIF1A	1.479139259	0.070601012	1
feature_19060	HIP1	3.164157056	0.257345094	1
feature_19071	HIVEP2	6.918989441	0.320179728	1
feature_19081	HLA-A	3.748705048	0.118511217	1
feature_19082	HLA-B	4.991329082	0.054006231	1
feature_19099	HLA-E	2.932543701	0.092171816	1
feature_19112	HLTF	1.286740863	0.061184561	1

feature_19116	HM13	1.368847185	0.001166622	1
feature_19119	HMBOX1	1.486492064	0.003285664	1
feature_19199	HMGN2	15.66269997	0.1419153	1
feature_19217	HMGXB4	1.164194114	0.262079725	1
feature_19323	HNRNPUL1	2.365152253	0.085637284	1
feature_19335	HOOK3	1.61026428	0.040318683	1
feature_19425	HSD17B11	1.071058585	0.045699507	1
feature_19446	HSH2D	2.099225808	0.085755287	1
feature_19494	HSPB11	1.447277104	0.150621017	1
feature_19541	HTR1F	12.90294719	0.315618896	1
feature_19558	HTRA3	2.268340322	0.169030086	1
feature_19560	HTT	1.40561121	0.205059413	1
feature_19566	HUWE1	1.85413231	0.007887611	1
feature_19585	IARS1	2.381083331	0.080349404	1
feature_19601	ICE2	1.150713972	0.25612473	1
feature_19614	IDH2	2.569805324	0.037295629	1
feature_19638	IFI16	4.267077796	0.212865494	1
feature_19693	IGBP1	1.447277104	0.126948942	1
feature_19703	IGF1R	3.23523417	0.153083839	1
feature_19708	IGF2BP3	8.738808662	0.013312083	1
feature_19709	IGF2R	2.627402296	0.017438997	1
feature_19722	IGHD	1.61026428	0.074775884	1
feature_19775	IGLC3	3.827134967	0.241814737	1
feature_19817	IKBKB	1.502423141	0.364161369	1
feature_19822	IKZF1	17.55359631	0.358826961	1
feature_19847	IL17RA	1.384778262	0.498401277	1
feature_19915	ILRUN	1.416640417	0.036408646	1
feature_19965	INO80	2.47421886	0.101175345	1
feature_19974	INPP4A	1.234045761	0.289403893	1
feature_19976	INPP5A	1.446051637	0.368621478	1
feature_19977	INPP5B	1.047774703	0.443063256	1
feature_19992	INSR	4.595503083	0.054314052	1
feature_20014	INTS7	1.113949947	0.098176938	1
feature_20019	INVS	2.550197844	0.424328432	1
feature_20020	IP6K1	1.617617085	0.159458867	1
feature_20033	IPO9	1.512226881	0.127724678	1
feature_20041	IQCB1	2.064912719	0.033358161	1
feature_20054	IQGAP1	4.354085988	0.074607761	1
feature_20055	IQGAP2	6.013368967	0.122180635	1
feature_20064	IRAG2	14.90903747	0.085440339	1
feature_20069	IRAK3	1.349239705	0.261479878	1
feature_20071	IREB2	1.176448789	0.161570651	1
feature_20072	IRF1	1.849230441	0.026168493	1
feature_20074	IRF2	1.110273545	0.058720303	1
feature_20115	ITFG1	3.470523928	0.162707464	1
feature_20127	ITGA4	8.225337784	0.082439481	1
feature_20141	ITGB1	1.069833118	0.219442795	1
feature_20149	ITGB3BP	1.711978082	0.163737474	1
feature_20168	ITM2C	1.85413231	0.066998784	1
feature_20180	ITPR2	1.943591437	0.120971217	1

feature_20191	ITSN2	3.26464539	0.214845935	1
feature_20195	IWS1	1.626195358	0.203219275	1
feature_20208	JAK1	1.676439525	0.120352026	1
feature_20219	JARID2	5.262157397	0.0926621	1
feature_20222	JAZF1	1.204634541	0.566516241	1
feature_20246	JPX	3.196019211	0.522121453	1
feature_20256	JUND	9.035371795	0.100285254	1
feature_20265	KANSL1L	1.458306312	0.275138242	1
feature_20278	KAT6A	2.664166321	0.071397024	1
feature_20283	KATNAL1	1.033069093	0.329919514	1
feature_20286	KATNBL1	1.781829729	0.253486	1
feature_20307	KCMF1	1.699723407	0.100677502	1
feature_20421	KCTD20	1.946042372	0.044643995	1
feature_20443	KDM2A	3.457043786	0.080293536	1
feature_20444	KDM2B	1.340661433	0.069906765	1
feature_20447	KDM3B	1.916631152	0.27530218	1
feature_20451	KDM4C	1.553892776	0.055951229	1
feature_20453	KDM5A	2.476669795	0.022133481	1
feature_20458	KDM6A	3.80140015	0.546988329	1
feature_20483	KIAA0319L	1.378650925	0.234924036	1
feature_20495	KIAA1328	1.998737474	0.231384652	1
feature_20509	KIAA2026	1.046549235	0.161215673	1
feature_20512	KIF11	1.742614769	0.27914132	1
feature_20518	KIF16B	1.513452349	0.438107086	1
feature_20550	KIF4A	1.028167223	0.211842275	1
feature_20559	KIF9	1.635999097	0.242704413	1
feature_20603	KLF6	5.873665674	0.109155868	1
feature_20629	KLHL2	1.120077285	0.287439858	1
feature_20677	KLRK1-AS1	1.377425457	0.226835629	1
feature_20679	KMT2A	3.22052856	0.101099462	1
feature_20696	KNL1	1.961973449	0.05899309	1
feature_20701	KNTC1	2.110255016	0.334868457	1
feature_20702	KPNA1	1.280613526	0.005864981	1
feature_20710	KPNA6	1.257329643	0.044054793	1
feature_20804	L3MBTL4	1.237722164	0.139622607	1
feature_20831	LAMP1	1.648253772	0.128736679	1
feature_20860	LARP4	1.218114684	0.037664463	1
feature_20861	LARP4B	2.158048248	0.030801421	1
feature_20865	LARP7	1.165419582	0.022262072	1
feature_20891	LCLAT1	1.480364726	0.13751128	1
feature_20901	LCOR	3.117589291	0.030478416	1
feature_20902	LCORL	2.481571665	0.029064482	1
feature_20909	LDAH	1.172772387	0.152493682	1
feature_20984	LGR6	5.292794084	0.24881883	1
feature_21004	LIG1	1.164194114	0.222974777	1
feature_21022	LIMD1	1.589431333	0.276958411	1
feature_21024	LIMD2	1.30757381	0.00692387	1
feature_21028	LIMS1	1.982806397	0.251232294	1
feature_21035	LIN52	1.427669624	0.361169402	1
feature_21036	LIN54	1.017138016	0.143200616	1

feature_21041	LINC-PINT	1.37129812	0.323490644	1
feature_21101	LINC00342	1.914180217	0.217224434	1
feature_21322	LINC01004	1.672763122	0.294977482	1
feature_21326	LINC01013	24.50199697	0.454625433	1
feature_21337	LINC01036	2.22054709	0.145769753	1
feature_21377	LINC01122	4.760941194	0.245792755	1
feature_21421	LINC01221	5.545240387	0.037773402	1
feature_21499	LINC01374	1.31247568	0.487192733	1
feature_21542	LINC01473	1.354141575	0.28858805	1
feature_21605	LINC01572	3.257292585	0.36940193	1
feature_22359	LINC02940	1.023265353	0.532246152	1
feature_22404	LINC03000	20.44569958	0.532894972	1
feature_22477	LMAN2	1.541638101	0.169917299	1
feature_22481	LMBRD1	1.534285296	0.180115799	1
feature_22491	LMNB1	2.297751541	0.011269763	1
feature_22498	LMO2	2.122509691	0.566994565	1
feature_22514	LNCAROD	19.14057671	0.670622052	1
feature_22525	LNPEP	2.341868371	0.161773943	1
feature_22531	LONP2	1.202183606	0.02353728	1
feature_22559	LPIN2	1.207085476	0.10412082	1
feature_22562	LPP	6.368754539	0.221909734	1
feature_22569	LRBA	3.231557768	0.298279773	1
feature_22570	LRCH1	3.268321792	0.51067846	1
feature_22572	LRCH3	1.681341395	0.135725559	1
feature_22696	LRRFIP2	1.735261964	0.099893297	1
feature_22700	LRRK1	1.574725723	0.176659707	1
feature_22743	LST1	1.839426701	0.76763118	1
feature_22770	LUZP1	1.627420825	0.132184929	1
feature_22789	LYN	2.566128921	0.36950333	1
feature_22803	LYRM2	1.147037569	0.109237378	1
feature_22812	LYST	1.958297047	0.718800624	1
feature_22832	MACF1	7.958185872	0.192463373	1
feature_22834	MACO1	1.028167223	0.063338747	1
feature_22890	MALAT1	180.1486227	0.338345924	1
feature_22894	MALT1	1.008559743	0.064096688	1
feature_22901	MAML3	10.73386973	0.326252636	1
feature_22904	MAN1A1	1.003657873	0.605164154	1
feature_22909	MAN2A1	1.931336762	0.02836938	1
feature_22915	MANBA	1.816142818	0.17355108	1
feature_22930	MAP1LC3B	3.227881365	0.339150162	1
feature_22940	MAP2K5	1.852906843	0.089654071	1
feature_22953	MAP3K2	1.830848428	0.025895925	1
feature_22955	MAP3K20	2.41539642	0.198289962	1
feature_22961	MAP3K5	7.279276883	0.283847823	1
feature_22965	MAP3K7	1.452178974	0.095627891	1
feature_22976	MAP4K5	1.753643976	0.047844471	1
feature_22986	MAPK1	2.367603188	0.077896033	1
feature_22992	MAPK14	2.867593924	0.230613684	1
feature_23006	MAPKAP1	2.497502742	0.193067676	1
feature_23007	MAPKAPK2	1.128655557	0.021463165	1

feature_23011	MAPKBP1	1.814917351	0.142669276	1
feature_23028	MARCHF7	1.990159202	0.098767497	1
feature_23059	MASTL	1.265907916	0.554542966	1
feature_23074	MAX	1.422767754	0.095378185	1
feature_23079	MBD2	1.604136943	0.174056965	1
feature_23084	MBD5	2.145793573	0.083802309	1
feature_23091	MBNL2	1.31247568	0.073834341	1
feature_23092	MBNL3	1.738938367	0.426815167	1
feature_23097	MBP	2.919063558	0.017832459	1
feature_23107	MCCC1	1.313701148	0.196976052	1
feature_23118	MCL1	2.71808689	0.167700028	1
feature_23119	MCM10	1.096793402	0.174296153	1
feature_23130	MCM9	1.819819221	0.180714365	1
feature_23145	MCU	1.235271229	0.222550624	1
feature_23146	MCUB	1.3909056	0.231279991	1
feature_23175	MECP2	1.12743009	0.221280283	1
feature_23183	MED13L	14.54262269	0.00526965	1
feature_23184	MED14	1.133557427	0.338897376	1
feature_23187	MED15	1.454629909	0.244261947	1
feature_23196	MED23	1.090666065	0.221131193	1
feature_23200	MED27	1.35659251	0.05277312	1
feature_23207	MED4	1.011010678	0.054785747	1
feature_23214	MEF2A	8.899344903	0.191356827	1
feature_23217	MEF2C-AS1	2.44970951	0.061896773	1
feature_23219	MEF2D	1.742614769	0.107955804	1
feature_23228	MEGF9	1.101695272	0.165320402	1
feature_23239	MELK	1.784280664	0.577798297	1
feature_23353	METTL15	1.458306312	0.14052952	1
feature_23408	MFSD14CP.1	1.149488504	0.006759093	1
feature_23440	MGME1	1.090666065	0.221794654	1
feature_23441	MGMT	1.818593753	0.404821036	1
feature_23445	MGST2	1.181350659	0.1236117	1
feature_23455	MIB1	1.531834361	0.120621644	1
feature_23458	MICAL1	1.248751371	0.181849692	1
feature_23471	MICU1	1.579627593	0.133151772	1
feature_23472	MICU2	2.301427944	0.178299995	1
feature_23484	MIER1	1.37129812	0.034885534	1
feature_23560	MIR3667HG	1.253653241	0.413252234	1
feature_23575	MIR4432HG	1.123753687	0.225263271	1
feature_23639	MIS18BP1	3.751155983	0.09654477	1
feature_23649	MKI67	3.019551892	0.071361004	1
feature_23727	MMS22L	4.493789281	0.254772137	1
feature_23740	MOB3A	2.317359021	0.089245773	1
feature_23758	MON2	2.169077455	0.240625445	1
feature_23785	MPC1	1.452178974	0.192571911	1
feature_23791	MPG	1.359043445	0.230865512	1
feature_23802	MPHOSPH9	2.117607821	0.26353513	1
feature_23808	MPO	1.223016554	0.635066588	1
feature_23818	MPRIP	1.856583245	0.300894421	1
feature_23835	MRE11	1.746291171	0.160305976	1

feature_23890	MRPL33	3.379839334	0.235432433	1
feature_23928	MRPL57	1.58085306	0.029692486	1
feature_23988	MRTFA	2.270791257	0.211879039	1
feature_24016	MSH3	1.769575054	0.106334958	1
feature_24020	MSH6	13.08676731	0.072909554	1
feature_24030	MSN	1.897023673	0.082232892	1
feature_24058	MT-ND6	1.295319136	0.009903715	1
feature_24059	MT-RNR1	77.26940157	0.343192829	1
feature_24060	MT-RNR2	196.0024956	0.336581066	1
feature_24189	MTM1	1.121302752	0.254105383	1
feature_24193	MTMR12	1.818593753	0.275626813	1
feature_24238	MTOR	1.270809786	0.017970315	1
feature_24290	MXD3	1.036745495	0.49150561	1
feature_24305	MYBL2	2.471767925	0.224420707	1
feature_24313	MYCBP2	7.860148473	0.126886823	1
feature_24339	MYH9	3.640863909	0.040418544	1
feature_24361	MYLIP	1.215663749	0.327708501	1
feature_24371	MYO15B	1.154390374	0.407482613	1
feature_24374	MYO18A	1.794084404	0.155017655	1
feature_24384	MYO1F	1.291642733	0.46437946	1
feature_24390	MYO5A	1.018363483	0.069377759	1
feature_24397	MYO9B	2.903132481	0.242131532	1
feature_24426	MZT2A	1.221791086	0.086963849	1
feature_24429	N4BP2	2.050207109	0.017524108	1
feature_24431	N4BP2L2	2.843084574	0.020987572	1
feature_24442	NAA35	1.149488504	0.071031226	1
feature_24443	NAA38	2.138440768	0.089511981	1
feature_24469	NADK2	1.066156715	0.236173468	1
feature_24472	NAE1	1.209536411	0.050479287	1
feature_24540	NBAS	1.993835604	0.18539251	1
feature_24571	NCAPD2	1.536736231	0.412355144	1
feature_24572	NCAPD3	1.518354219	0.193601981	1
feature_24573	NCAPG	1.104146207	0.313968829	1
feature_24574	NCAPG2	2.213194285	0.429381428	1
feature_24576	NCAPH	1.268358851	0.286754265	1
feature_24596	NCK2	1.204634541	0.306744502	1
feature_24598	NCKAP1L	1.422767754	0.235844841	1
feature_24611	NCOA1	1.617617085	0.139767788	1
feature_24612	NCOA2	3.605325352	0.194881724	1
feature_24613	NCOA3	1.995061072	0.040308369	1
feature_24617	NCOA6	1.205860009	0.089589541	1
feature_24618	NCOA7	1.247525904	0.348035562	1
feature_24624	NCOR2	1.173997854	0.366656697	1
feature_24631	NDC1	1.338210498	0.202247718	1
feature_24632	NDC80	1.013461613	0.354198013	1
feature_24650	NDUFA10	1.340661433	0.048199907	1
feature_24732	NEAT1	5.094268351	0.668000743	1
feature_24772	NEK7	1.997512007	0.093515014	1
feature_24781	NEMF	2.389661603	0.179271837	1
feature_24809	NF1	2.975435063	0.102231112	1

feature_24816	NFATC2IP	1.252427774	0.171988919	1
feature_24818	NFATC3	3.958259989	0.089974446	1
feature_24845	NFX1	2.017119486	0.142470388	1
feature_24859	NGLY1	1.825946558	0.187834334	1
feature_24922	NKG7	1.765898651	0.305428548	1
feature_24928	NKTR	3.689882609	0.233773837	1
feature_24938	NLK	1.511001414	0.000212386	1
feature_24943	NLRP1	1.298995538	0.724005162	1
feature_24959	NMD3	1.006108808	0.240359126	1
feature_24988	NNT	1.546539971	0.028187628	1
feature_25071	NPEPPS	1.531834361	0.113369421	1
feature_25104	NPLOC4	1.003657873	0.288206042	1
feature_25165	NR2C2	1.598009605	0.127907072	1
feature_25194	NRDC	1.501197674	0.061249119	1
feature_25196	NREP	1.525707023	0.415612221	1
feature_25198	NRF1	2.088196601	0.178494646	1
feature_25211	NRM	1.479139259	0.159628057	1
feature_25232	NSD1	2.351672111	0.008936221	1
feature_25233	NSD2	4.350409585	0.246444575	1
feature_25234	NSD3	2.4840226	0.050058615	1
feature_25242	NSMAF	1.373749055	0.230916394	1
feature_25245	NSMCE2	3.132294901	0.249451902	1
feature_25249	NSRP1	1.862710583	0.16385437	1
feature_25262	NT5C2	5.186178412	0.032843069	1
feature_25301	NUBPL	1.143361167	0.403576386	1
feature_25306	NUCKS1	10.88950411	0.017499796	1
feature_25314	NUDT1	1.643351902	0.002580428	1
feature_25336	NUDT5	1.884768998	0.029959127	1
feature_25346	NUMA1	1.814917351	0.044314339	1
feature_25347	NUMB	1.984031864	0.557904372	1
feature_25349	NUP107	1.871288855	0.083922346	1
feature_25358	NUP205	1.199732671	0.167128165	1
feature_25363	NUP214	1.747516639	0.129437882	1
feature_25368	NUP50	1.003657873	0.179613195	1
feature_25371	NUP54	1.095567935	0.0683206	1
feature_25376	NUP88	1.080862325	0.151376231	1
feature_25377	NUP93	1.139684765	0.003053003	1
feature_25397	NVL	1.13723383	0.155915662	1
feature_25466	OFD1	1.658057512	0.035911929	1
feature_25470	OGA	1.731585562	0.07297163	1
feature_25482	OGT	1.607813345	0.068271857	1
feature_25512	OPA1	1.441149767	0.082413008	1
feature_25622	ORC3	1.154390374	0.072421271	1
feature_25625	ORC6	1.370072652	0.177852503	1
feature_25642	OSBPL9	1.852906843	0.001808465	1
feature_25646	OSER1	1.158066777	0.106849672	1
feature_25663	OSTF1	1.449728039	0.05248912	1
feature_25714	OXR1	1.134782895	0.159510602	1
feature_25736	P2RY8	1.598009605	0.612856371	1
feature_25777	PACS1	2.187459467	0.055620632	1

feature_25780	PACSIN2	1.834524831	0.042535349	1
feature_25786	PAFAH1B1	1.501197674	0.167956889	1
feature_25792	PAG1	1.481590194	0.508127716	1
feature_25824	PAN3	34.1807392	0.143903088	1
feature_25877	PARP14	2.423974693	0.368513787	1
feature_25883	PARP4	1.224242021	0.080238832	1
feature_25887	PARP8	10.20569325	0.201782473	1
feature_25895	PARVG	1.3909056	0.283017496	1
feature_25915	PAXBP1	1.552667308	0.036551046	1
feature_25920	PAXX	1.023265353	0.082866177	1
feature_25924	PBRM1	4.296489016	0.14392107	1
feature_25928	PBX3	17.7423183	0.439151038	1
feature_25942	PCBD2	1.128655557	0.313219666	1
feature_25945	PCBP2	5.066082598	0.083593983	1
feature_26003	PCED1B-AS1	1.012236146	0.080397961	1
feature_26016	PCLAF	5.089366481	0.405500377	1
feature_26018	PCM1	4.013406026	0.041192997	1
feature_26020	PCMTD1	1.655606577	0.252768671	1
feature_26023	PCNA	3.599198014	0.030457172	1
feature_26030	PCNX1	2.115156886	0.19031776	1
feature_26033	PCNX4	1.226692956	0.0710446	1
feature_26097	PDE4D	2.074716458	0.067841361	1
feature_26114	PDE8A	2.436229368	0.505660341	1
feature_26162	PDSS2	2.544070507	0.014097444	1
feature_26222	PEX14	1.042872833	0.37588707	1
feature_26245	PFKL	1.528157958	0.104108087	1
feature_26288	PGLS	1.650704707	0.234431845	1
feature_26310	PHACTR1	2.75730185	0.463109718	1
feature_26315	PHACTR4	2.844310041	0.107130265	1
feature_26331	PHC3	1.867612453	0.26931132	1
feature_26346	PHF20L1	1.867612453	0.344622703	1
feature_26347	PHF21A	1.562471048	0.001252259	1
feature_26352	PHF3	2.684999268	0.048679241	1
feature_26360	PHIP	7.9005889	0.058819621	1
feature_26366	PHKB	2.768331057	0.264085455	1
feature_26385	PHTF1	1.013461613	0.02767861	1
feature_26386	PHTF2	1.148263037	0.194200078	1
feature_26390	PHYKPL	1.091891532	0.131896041	1
feature_26395	PI4KA	1.435022429	0.018328847	1
feature_26400	PIAS1	3.769537995	0.138719881	1
feature_26405	PICALM	2.955827583	0.003952485	1
feature_26429	PIGL	1.436247897	0.154912041	1
feature_26431	PIGN	1.38600373	0.215555428	1
feature_26446	PIH1D1	1.002432406	0.25065134	1
feature_26448	PIK3AP1	5.795235754	0.016741938	1
feature_26452	PIK3C3	5.808715897	0.536978411	1
feature_26455	PIK3CB	1.082087793	0.054568667	1
feature_26456	PIK3CD	2.68867567	0.110865003	1
feature_26462	PIK3R1	3.720519296	0.260746746	1
feature_26469	PIKFYVE	1.773251456	0.144309	1

feature_26494	PIP5K1A	1.721781822	0.287650071	1
feature_26506	PITPNC1	4.520749566	0.450506434	1
feature_26546	PKIG	1.562471048	0.210144919	1
feature_26553	PKN2	2.344319306	0.028535435	1
feature_26559	PKP4	1.030618158	0.135220922	1
feature_26588	PLAC8	1.38600373	0.204545896	1
feature_26602	PLCB1	29.94919996	0.658853875	1
feature_26685	PLP2	1.213212814	0.229419365	1
feature_26719	PLXNC1	1.626195358	0.030720351	1
feature_26768	PNN	4.758490259	0.040179358	1
feature_26772	PNP	1.011010678	0.126263364	1
feature_26785	PNRC2	1.338210498	0.003220846	1
feature_26803	POGZ	2.078392861	0.106286604	1
feature_26804	POLA1	2.061236316	0.100701183	1
feature_26814	POLE	1.276937123	0.374119569	1
feature_26815	POLE2	1.267133383	0.187627487	1
feature_26825	POLK	1.013461613	0.209374864	1
feature_26829	POLQ	3.297733012	0.538259138	1
feature_26835	POLR1F	3.088178072	0.465382779	1
feature_26840	POLR2B	1.105371675	0.013852355	1
feature_26852	POLR2J3.1	1.612715215	0.313746601	1
feature_26874	POM121	1.148263037	0.09171447	1
feature_26914	POU2F1	2.241380037	0.224824616	1
feature_26936	PPA2	1.660508447	0.074734596	1
feature_26959	PPFIBP1	1.07350952	0.293029097	1
feature_27018	PPIP5K2	1.412964015	0.249122189	1
feature_27023	PPM1D	1.11272448	0.088784078	1
feature_27028	PPM1H	2.900681546	0.673879287	1
feature_27043	PPP1R10	1.836975766	0.334669041	1
feature_27046	PPP1R12A	3.464396591	0.055515445	1
feature_27049	PPP1R12B	1.672763122	0.314865971	1
feature_27073	PPP1R21	1.368847185	0.099835332	1
feature_27111	PPP2R2D	1.06370578	0.138478554	1
feature_27114	PPP2R3C	1.37129812	0.04728675	1
feature_27117	PPP2R5C	33.45526244	0.136303565	1
feature_27120	PPP2R5E	2.368828656	0.02384198	1
feature_27122	PPP3CB	1.776927859	0.396886233	1
feature_27126	PPP4C	1.276937123	0.013265597	1
feature_27138	PPP6R2	2.275693127	0.307488112	1
feature_27139	PPP6R3	3.970514664	0.039839568	1
feature_27210	PREX1	1.86393605	0.446725491	1
feature_27224	PRIM2	4.172716799	0.263600462	1
feature_27232	PRKACB	1.310024745	0.338499554	1
feature_27264	PRKD2	1.377425457	0.351677432	1
feature_27274	PRKRIP1	1.561245581	0.170182893	1
feature_27275	PRKX	1.681341395	0.489275176	1
feature_27283	PRMT2	1.047774703	0.19060278	1
feature_27306	PROM1	8.906697708	0.161342767	1
feature_27350	PRR14L	1.226692956	0.125893658	1
feature_27374	PRRC2B	2.854113781	0.007797977	1

feature_27405	PRSS57	2.025697759	0.391277826	1
feature_27424	PSD3	5.417791768	0.36545125	1
feature_27426	PSEN1	1.64947924	0.239958352	1
feature_27475	PSMD1	1.818593753	0.027740087	1
feature_27508	PSME3IP1	1.227918424	0.285152788	1
feature_27510	PSMF1	1.004883341	0.071433199	1
feature_27533	PTBP3	2.447258575	0.013558506	1
feature_27537	PTCH1	1.780604261	0.718978837	1
feature_27557	PTGER4	1.009785211	0.165284508	1
feature_27583	PTK2	7.37363788	0.351647474	1
feature_27584	PTK2B	5.115101298	0.206360857	1
feature_27614	PTPN1	1.360268913	0.07032526	1
feature_27634	PTPRA	2.95705305	0.154687537	1
feature_27636	PTPRC	3.183764536	0.037056919	1
feature_27640	PTPRE	1.153164907	0.502440821	1
feature_27645	PTPRJ	1.263456981	0.383766534	1
feature_27671	PUDP	3.156804251	0.738642768	1
feature_27676	PUM2	4.164138527	0.021321252	1
feature_27691	PVT1	2.617598556	0.17375016	1
feature_27703	PXK	1.950944242	0.08823038	1
feature_27710	PYCARD	1.748742106	0.010568272	1
feature_27732	QKI	4.992554549	0.060250917	1
feature_27736	QPRT	2.017119486	0.060690344	1
feature_27749	R3HCC1L	1.626195358	0.185092532	1
feature_27751	R3HDM2	3.670275129	0.369220857	1
feature_27791	RAB2A	2.794065874	0.135951152	1
feature_27832	RAB6A	1.247525904	0.034045723	1
feature_27838	RAB8A	1.933787697	0.020475859	1
feature_27843	RABEP1	1.177674257	0.232195927	1
feature_27846	RABGAP1	1.557569178	0.069465229	1
feature_27847	RABGAP1L	2.839408171	0.419978757	1
feature_27860	RABL6	1.30757381	0.01703633	1
feature_27882	RAD23B	1.018363483	0.028092056	1
feature_27885	RAD51	1.156841309	0.147638487	1
feature_27887	RAD51AP1	1.593107735	0.410722403	1
feature_27890	RAD51B	5.135934245	0.399466568	1
feature_27896	RAD54L2	1.208310944	0.312969783	1
feature_27915	RALBP1	2.010992149	0.100230299	1
feature_27917	RALGAPA1	1.232820294	0.11913166	1
feature_27944	RANBP9	4.193549747	0.075354273	1
feature_27955	RAP1A	1.879867128	0.17739967	1
feature_27961	RAP1GAP2	1.676439525	0.286463853	1
feature_27962	RAP1GDS1	2.055108979	0.172433972	1
feature_27967	RAPGEF1	3.028130165	0.399786513	1
feature_27985	RARS2	1.123753687	0.116855784	1
feature_27987	RASA2	2.572256259	0.506889899	1
feature_28022	RASSF3	1.421542287	0.213561608	1
feature_28034	RB1	6.097926224	0.128279745	1
feature_28039	RBBP4	2.843084574	0.019270803	1
feature_28055	RBIS	1.382327327	0.062404751	1

feature_28058	RBL1	3.789145475	0.252106129	1
feature_28059	RBL2	1.12252822	0.199283248	1
feature_28070	RBM17	1.502423141	0.033695494	1
feature_28078	RBM23	1.40561121	0.102336829	1
feature_28082	RBM26	2.774458395	0.051274721	1
feature_28087	RBM33	1.930111295	0.078048124	1
feature_28090	RBM38	1.109048077	0.424938063	1
feature_28092	RBM39	9.067233949	0.048172329	1
feature_28094	RBM4	1.199732671	0.042275173	1
feature_28104	RBM5	1.496295804	0.010461078	1
feature_28106	RBM6	6.01704537	0.25109054	1
feature_28111	RBMS1	1.3909056	0.189390584	1
feature_28137	RC3H1	2.349221176	0.169077869	1
feature_28140	RC3H2	1.306348343	0.143499343	1
feature_28166	RCOR3	1.033069093	0.200202385	1
feature_28183	RDX	4.961917862	0.296156775	1
feature_28188	RECK	1.187477997	0.424164116	1
feature_28195	REEP3	2.635980568	0.216421718	1
feature_28204	RELCH	1.32227942	0.127827473	1
feature_28217	REPS1	2.301427944	0.07211832	1
feature_28219	RER1	1.020814418	0.125338637	1
feature_28220	RERE	7.291531558	0.192173985	1
feature_28225	REST	1.193605334	0.006605951	1
feature_28234	REV3L	5.15799266	0.023773276	1
feature_28242	RFC1	1.995061072	0.058182268	1
feature_28243	RFC2	1.045323768	0.0518308	1
feature_28259	RFTN1	2.241380037	0.346101091	1
feature_28261	RFWD3	1.509775946	0.104567803	1
feature_28265	RFX3-DT	1.164194114	0.269451058	1
feature_28269	RFX7	2.373730526	0.171245982	1
feature_28294	RGS12	1.637224565	0.096379487	1
feature_28339	RHOBTB3	1.449728039	0.068621884	1
feature_28344	RHOH	3.205822951	0.665322797	1
feature_28366	RIC1	1.871288855	0.175482196	1
feature_28371	RICTOR	1.988933734	0.065134057	1
feature_28373	RIF1	2.687450203	0.160752374	1
feature_28383	RIMKLB	1.262231513	0.54999107	1
feature_28399	RIOK3	1.011010678	0.137951297	1
feature_28408	RIPOR2	3.491356875	0.51603982	1
feature_28784	RNASEH2B	4.651874587	0.091612435	1
feature_28786	RNASEH2C	1.158066777	0.001223357	1
feature_28798	RNF111	1.432571494	0.091821061	1
feature_28802	RNF115	1.578402126	0.180188445	1
feature_28810	RNF13	1.397032937	0.097786403	1
feature_28811	RNF130	6.351597994	0.169885368	1
feature_28823	RNF149	1.276937123	0.399543566	1
feature_28834	RNF168	1.286740863	0.172039723	1
feature_28835	RNF169	1.191154399	0.125753145	1
feature_28846	RNF19A	1.264682448	0.182398164	1
feature_28854	RNF213	1.95216971	0.207505957	1

feature_28858	RNF216	1.723007289	0.129083072	1
feature_28862	RNF220	27.3806201	0.293737194	1
feature_28891	RNGTT	3.905564887	0.152002615	1
feature_29207	ROCK2	2.060010849	0.067975097	1
feature_29229	RPA2	1.12252822	0.038530432	1
feature_29235	RPAP2	1.136008362	0.026571541	1
feature_29375	RPL18AP3	1.166645049	0.014409624	1
feature_29515	RPL27A	29.3671029	0.016377497	1
feature_29879	RPLP1	99.90991342	0.029989101	1
feature_29894	RPRD1A	1.949718775	0.336812402	1
feature_29896	RPRD2	2.023246824	0.050925558	1
feature_29911	RPS12	106.2161691	0.016058427	1
feature_29967	RPS19	48.90350561	0.003133957	1
feature_29973	RPS20	26.02525306	0.005228161	1
feature_30132	RPS4X	54.35683593	0.340521457	1
feature_30160	RPS6KA3	2.903132481	0.08785931	1
feature_30195	RPS9	30.99329826	0.010835606	1
feature_30232	RPTOR	1.651930175	0.182822922	1
feature_30245	RRAS2	1.38110186	0.319946436	1
feature_30249	RREB1	2.122509691	0.391236213	1
feature_30251	RRM1	1.412964015	0.139556603	1
feature_30252	RRM2	4.458250724	0.646968219	1
feature_30273	RSBN1L	1.828397493	0.006248393	1
feature_30299	RSRC1	5.280539409	0.000861974	1
feature_30302	RSU1	1.197281737	0.075744486	1
feature_30309	RTF1	2.099225808	0.022418518	1
feature_30325	RTN4	4.12859997	0.10696451	1
feature_30336	RTTN	1.428895092	0.16567388	1
feature_30348	RUNX1	18.8881304	0.129378263	1
feature_30350	RUNX2	12.49486652	0.000368062	1
feature_30402	S100PBP	1.041647365	0.259918933	1
feature_30417	SAE1	1.757320379	0.17790099	1
feature_30419	SAFB2	1.203409074	0.046019052	1
feature_30432	SAMD4A	1.003657873	0.166455314	1
feature_30485	SBF2	2.170302923	0.448160693	1
feature_30498	SCAF4	1.177674257	0.150365885	1
feature_30499	SCAF8	3.438661773	0.084070809	1
feature_30500	SCAI	1.225467489	0.055915338	1
feature_30509	SCAPER	2.438680303	0.051624026	1
feature_30547	SCLT1	3.078374332	0.22279078	1
feature_30549	SCMH1	4.224186434	0.115793973	1
feature_30633	SEC11A	1.428895092	0.024426061	1
feature_30657	SEC24B	1.379876392	0.101835922	1
feature_30670	SEC63	3.84061511	0.015273225	1
feature_30672	SECISBP2	2.030599629	0.254734785	1
feature_30690	SELENOO	1.363945315	0.239073471	1
feature_30732	SENP5	1.735261964	0.029545055	1
feature_30734	SENP7	1.171546919	0.249498794	1
feature_30749	SEPTIN2	1.754869444	0.003634196	1
feature_30755	SEPTIN6	4.790352414	0.244308654	1

feature_30788	SERINC3	1.008559743	0.004784934	1
feature_30790	SERINC5	2.216870687	0.188675304	1
feature_30823	SESN1	2.812447887	0.590658706	1
feature_30826	SESTD1	2.466866055	0.184394956	1
feature_30832	SETD2	4.116345295	0.105576349	1
feature_30836	SETD5	2.501179145	0.039870658	1
feature_30841	SETDB2	1.365170782	0.261402903	1
feature_30858	SETX	2.836957236	0.132889514	1
feature_30879	SFPQ	6.637131919	0.013373479	1
feature_30886	SFSWAP	1.794084404	0.12569406	1
feature_30929	SH2B3	1.512226881	0.737285432	1
feature_30934	SH2D3C	1.264682448	0.1743446	1
feature_30959	SH3PXD2A	1.588205865	0.04618719	1
feature_30962	SH3RF1	1.466884584	0.370858384	1
feature_30983	SHCBP1	1.224242021	0.417203292	1
feature_31001	SHLD1	1.283064461	0.378972975	1
feature_31002	SHLD2	1.611489748	0.084680854	1
feature_31015	SHPRH	1.506099544	0.063092646	1
feature_31019	SHROOM3	6.254786063	0.33322397	1
feature_31057	SIMC1	1.731585562	0.227019203	1
feature_31058	SIN3A	1.036745495	0.01547198	1
feature_31068	SIPA1L3	1.64947924	0.056489819	1
feature_31089	SKA2	1.177674257	0.031669437	1
feature_31095	SKAP2	1.650704707	0.079450527	1
feature_31124	SLC12A2	1.118851817	0.105474036	1
feature_31125	SLC12A2-DT	1.06370578	0.156167672	1
feature_31186	SLC20A2	1.436247897	0.055885769	1
feature_31194	SLC22A16	1.148263037	0.215851814	1
feature_31208	SLC23A2	1.188703464	0.255413981	1
feature_31251	SLC25A36	1.207085476	0.009013566	1
feature_31274	SLC25A6	8.415285245	0.609763122	1
feature_31307	SLC2A3	1.04900017	0.425053569	1
feature_31373	SLC38A2	1.36149438	0.011558468	1
feature_31377	SLC38A4-AS1	1.876190725	0.71148724	1
feature_31382	SLC38A9	1.224242021	0.066037454	1
feature_31385	SLC39A11	1.234045761	0.014317467	1
feature_31408	SLC44A2	1.530608893	0.261450869	1
feature_31549	SLMAP	2.185008532	0.05485267	1
feature_31562	SMAD3	1.526932491	0.579866753	1
feature_31575	SMARCA2	3.615129092	0.04358427	1
feature_31577	SMARCA4	3.107785551	0.033944896	1
feature_31595	SMC1A	3.705813686	0.505582321	1
feature_31596	SMC1B	7.003546698	0.450204903	1
feature_31601	SMC4	5.420242703	0.099903005	1
feature_31602	SMC5	2.274467659	0.014570778	1
feature_31604	SMC6	2.001188409	0.038701127	1
feature_31605	SMCHD1	4.35286052	0.075968096	1
feature_31624	SMG6	1.525707023	0.408100811	1
feature_31625	SMG7	1.68379233	0.028619198	1
feature_31654	SMIM3	1.328406758	0.127200004	1

feature_31694	SMURF2	1.839426701	0.086679706	1
feature_31745	SNHG29	19.40527768	0.003102889	1
feature_31751	SNHG5	15.29015786	0.016683116	1
feature_31853	SNRNP200	1.492619401	0.114473235	1
feature_31909	SNX1	1.115175415	0.000245654	1
feature_31926	SNX25	2.354123046	0.38374794	1
feature_31929	SNX29	1.406836677	0.290163478	1
feature_31952	SOCS2-AS1	1.734036497	0.222776875	1
feature_31965	SOD2	1.835750298	0.084288252	1
feature_31982	SOS1	2.823477094	0.060040651	1
feature_31983	SOS2	1.370072652	0.106481125	1
feature_32004	SP100	1.303897408	0.135880231	1
feature_32005	SP110	1.394582002	0.146131955	1
feature_32033	SPAG9	2.486473535	0.030729525	1
feature_32037	SPART	1.056352975	0.179384279	1
feature_32061	SPATA48	1.071058585	0.005591344	1
feature_32062	SPATA5	2.006090279	0.019146785	1
feature_32104	SPECC1	1.439924299	0.441827846	1
feature_32115	SPG11	1.560020113	0.20420928	1
feature_32117	SPG7	1.612715215	0.30587278	1
feature_32121	SPI1	1.017138016	0.086549061	1
feature_32125	SPIDR	5.695972888	0.359597255	1
feature_32126	SPIN1	2.345544773	0.020735777	1
feature_32143	SPN	4.241342979	0.054702276	1
feature_32155	SPOP	1.60536241	0.081744846	1
feature_32163	SPPL3	2.350446643	0.072401017	1
feature_32192	SPTLC1	1.019588951	0.130337486	1
feature_32194	SPTLC2	2.574707194	0.017070725	1
feature_32207	SRBD1	1.584529463	0.441528727	1
feature_32225	SRGAP2	1.042872833	0.218305852	1
feature_32232	SRGN	1.566147451	0.226750941	1
feature_32245	SRP54	1.008559743	0.015240694	1
feature_32254	SRPK2	1.586980398	0.233559413	1
feature_32282	SRSF4	3.979092936	0.193332353	1
feature_32283	SRSF5	4.517073164	0.050302994	1
feature_32314	SSR1	1.588205865	0.025498547	1
feature_32343	ST3GAL1	1.517128751	0.160528435	1
feature_32379	STAG1	10.49612904	0.109961956	1
feature_32397	STARD13	2.901907013	0.207854873	1
feature_32430	STIM1	1.971777189	0.547071942	1
feature_32439	STK10	2.71318502	0.258221435	1
feature_32447	STK24	2.249958309	0.239956688	1
feature_32452	STK3	3.104109149	0.391650966	1
feature_32501	STRN3	2.235252699	0.144460622	1
feature_32526	STX5	1.192379867	0.124040559	1
feature_32529	STX7	1.720556354	0.223010097	1
feature_32530	STX8	3.028130165	0.183776915	1
feature_32546	SUCLA2	1.072284053	0.061924715	1
feature_32551	SUCLG2	2.518335689	0.052410715	1
feature_32562	SUGP2	1.707076212	0.283007854	1

feature_32584	SUMF1	3.014650022	0.734716355	1
feature_32606	SUPT3H	26.61102652	0.082807892	1
feature_32625	SUZ12	3.139647706	0.144754642	1
feature_32654	SYF2	1.464433649	0.000324487	1
feature_32656	SYK	4.490112879	0.185875082	1
feature_32667	SYNE3	2.14701904	0.192865476	1
feature_32671	SYNGR1	1.631097228	0.376954482	1
feature_32715	SYTL3	1.982806397	0.539212515	1
feature_32723	TAB2	2.300202476	0.259590091	1
feature_32730	TACC1	1.220565619	0.041019181	1
feature_32732	TACC3	1.172772387	0.036270026	1
feature_32742	TAF1	1.189928932	0.160977769	1
feature_32744	TAF11	1.298995538	0.126337956	1
feature_32751	TAF1B	1.120077285	0.075178968	1
feature_32755	TAF2	1.243849501	0.043998997	1
feature_32756	TAF3	2.019570421	0.011614458	1
feature_32778	TAGAP-AS1	1.007334276	0.406209498	1
feature_32785	TALAM1	3.279351	0.042374881	1
feature_32795	TAOK1	2.600442011	0.059073887	1
feature_32797	TAOK3	6.170228806	0.520803942	1
feature_32802	TAPT1	7.768238411	0.094319484	1
feature_32803	TAPT1-AS1	1.892121803	0.338983663	1
feature_32804	TARBP1	2.422749225	0.200314829	1
feature_32835	TASOR	1.954620645	0.315954746	1
feature_32849	TBC1D1	3.291605675	0.177622332	1
feature_32855	TBC1D14	13.44950569	0.282693559	1
feature_32862	TBC1D22A	3.51831716	0.480974236	1
feature_32875	TBC1D32	2.004864812	0.337158343	1
feature_32879	TBC1D4	1.220565619	0.099411545	1
feature_32882	TBC1D8	1.08331326	0.214620473	1
feature_32894	TBCD	1.541638101	0.302886087	1
feature_32899	TBCK	1.273260721	0.162715242	1
feature_32903	TBL1X	1.193605334	0.064634661	1
feature_32951	TCF12	18.06584172	0.16336218	1
feature_32958	TCF3	1.719330887	0.004381991	1
feature_32962	TCF7L2	2.481571665	0.299157401	1
feature_33035	TENT2	2.295300606	0.128554393	1
feature_33064	TET2	1.115175415	0.194183532	1
feature_33067	TEX10	1.033069093	0.018924927	1
feature_33071	TEX14	4.34305678	0.520294667	1
feature_33088	TEX41	5.184952945	0.353477133	1
feature_33108	TFB1M	1.074734988	0.136344418	1
feature_33110	TFCP2	1.828397493	0.075680903	1
feature_33136	TGFBR2	3.656794986	0.386079198	1
feature_33153	THADA	2.280594997	0.082348465	1
feature_33286	TK1	1.613940683	0.360228612	1
feature_33308	TLK2	1.973002657	0.065386701	1
feature_33342	TM9SF2	1.412964015	0.100582637	1
feature_33362	TMCC1	3.105334616	0.378927279	1
feature_33406	TMEM117	1.501197674	0.513954373	1

feature_33421	TMEM131	1.795309871	0.092812396	1
feature_33422	TMEM131L	2.377406928	0.065697218	1
feature_33460	TMEM165	2.061236316	0.157236256	1
feature_33479	TMEM181	2.426425628	0.702491269	1
feature_33518	TMEM219	1.022039886	0.117237145	1
feature_33540	TMEM245	1.94236597	0.140663079	1
feature_33586	TMEM41B	1.023265353	0.311750107	1
feature_33611	TMEM65	3.615129092	0.361307244	1
feature_33627	TMEM87A	1.802662676	0.018844918	1
feature_33652	TMOD3	1.613940683	0.059403525	1
feature_33654	TMPO	4.008504156	0.082569086	1
feature_33687	TMTC2	1.770800521	0.603688867	1
feature_33695	TMX4	1.040421898	0.108915192	1
feature_33703	TNFAIP8	1.159292244	0.147868209	1
feature_33750	TNKS2	1.10782261	0.026071918	1
feature_33765	TNPO3	1.628646293	0.090987059	1
feature_33767	TNRC18	3.898212082	0.239568139	1
feature_33770	TNRC6A	1.305122876	0.092237893	1
feature_33771	TNRC6B	5.530534777	0.033641403	1
feature_33819	TOPBP1	2.129862495	0.128909693	1
feature_33860	TPGS2	1.209536411	0.133961728	1
feature_33869	TPM3	4.320998365	0.036095156	1
feature_33875	TPM4	6.740071188	0.030010314	1
feature_33880	TPP2	2.017119486	0.072538241	1
feature_33911	TPTEP1.1	1.274486188	0.092118386	1
feature_33915	TRA2B	6.252335128	0.275167657	1
feature_33926	TRAF3IP2	1.437473364	0.185063139	1
feature_33927	TRAF3IP2-AS1	3.7768908	0.29372996	1
feature_33993	TRAK1	3.148225979	0.06853469	1
feature_34002	TRAPPC10	1.473011921	0.179125967	1
feature_34010	TRAPPC2L	1.10292074	0.037835799	1
feature_34017	TRAPPC8	1.466884584	0.100363824	1
feature_34018	TRAPPC9	3.802625618	0.34273214	1
feature_34085	TRIM33	1.772025989	0.020856437	1
feature_34090	TRIM38	1.588205865	0.265647824	1
feature_34095	TRIM44	2.421523758	0.019279411	1
feature_34138	TRIP12	2.149469975	0.100370933	1
feature_34178	TRPC4AP	1.88354353	0.241302442	1
feature_34187	TRPM2	1.64947924	0.241236918	1
feature_34201	TRRAP	1.571049321	0.030328077	1
feature_34218	TSG101	1.235271229	0.018544786	1
feature_34242	TSPAN14	1.194830802	0.135534513	1
feature_34266	TSPOAP1-AS1	1.086989663	0.138158197	1
feature_34284	TTBK2	2.822251627	0.246489145	1
feature_34292	TTC17	3.642089377	0.026349715	1
feature_34303	TTC28	16.37837299	0.249286026	1
feature_34321	TTC39C	2.769556525	0.136711296	1
feature_34336	TTF1	2.72789063	0.05627254	1
feature_34337	TTF2	1.551441841	0.100692418	1
feature_34352	TTLL5	1.773251456	0.074574016	1

feature_34366	TTYH3	1.237722164	0.37087009	1
feature_34369	TUBA1B	21.38318221	0.393995854	1
feature_34380	TUBB	15.7521591	0.444747664	1
feature_34390	TUBB6	1.040421898	0.041139602	1
feature_34407	TUBGCP3	1.769575054	0.308915787	1
feature_34418	TULP4	1.486492064	0.013577919	1
feature_34458	TXNIP	1.147037569	0.325356807	1
feature_34471	TYMS	3.612678157	0.140338939	1
feature_34505	UBA3	1.022039886	0.061838343	1
feature_34516	UBAC2	5.945968255	0.414939613	1
feature_34520	UBAP1	1.383552795	0.048695438	1
feature_34525	UBASH3B	32.17464892	0.039703245	1
feature_34532	UBC	6.416547771	0.1243298	1
feature_34541	UBE2D2	3.745028646	0.133081132	1
feature_34547	UBE2E1	5.028093106	0.160801284	1
feature_34549	UBE2E2	4.468054464	0.131671337	1
feature_34562	UBE2J1	2.814898822	0.147243702	1
feature_34564	UBE2K	3.899437549	0.055784084	1
feature_34576	UBE2Q2	1.270809786	0.022242401	1
feature_34606	UBE4B	1.916631152	0.163131352	1
feature_34617	UBN1	1.460757247	0.191809184	1
feature_34628	UBR1	1.702174342	0.046060188	1
feature_34629	UBR2	2.447258575	0.090366098	1
feature_34630	UBR3	1.192379867	0.072839973	1
feature_34632	UBR5	3.091854474	0.07054941	1
feature_34638	UBXN1	1.995061072	0.075747253	1
feature_34645	UBXN7	1.140910232	0.081376883	1
feature_34652	UCK2	2.029374161	0.176224372	1
feature_34673	UGGT1	1.025716288	0.076231935	1
feature_34685	UIMC1	1.550216373	0.138919269	1
feature_34723	UNK	1.623744423	0.393109272	1
feature_34728	UPF2	2.118833288	0.040977748	1
feature_34729	UPF3A	1.790408001	0.123208777	1
feature_34733	UPF3B	1.328406758	0.043005745	1
feature_34786	USP1	2.251183777	0.081763996	1
feature_34787	USP10	1.833299363	0.010108449	1
feature_34794	USP15	3.160480653	0.126968822	1
feature_34804	USP24	1.813691883	0.123353394	1
feature_34805	USP25	1.87373979	0.134132513	1
feature_34816	USP32	2.834506301	0.345529106	1
feature_34818	USP33	1.971777189	0.224488304	1
feature_34819	USP34	4.686187677	0.002492736	1
feature_34823	USP37	1.544089036	0.082198268	1
feature_34827	USP4	1.348014238	0.064305722	1
feature_34836	USP47	2.316133554	0.064978095	1
feature_34837	USP48	1.810015481	0.106141959	1
feature_34845	USP6NL	1.181350659	0.133807975	1
feature_34847	USP7	1.0440983	0.001505271	1
feature_34849	USP8	1.35659251	0.003260446	1
feature_34869	UTRN	4.89451715	0.259164045	1

feature_34877	UXS1	1.297770071	0.070281074	1
feature_34904	VAV1	1.316152083	0.160917687	1
feature_34906	VAV3	8.694691833	0.315419778	1
feature_34946	VGLL4	1.874965258	0.215655982	1
feature_34947	VHL	1.074734988	0.056030409	1
feature_34950	VIM	11.09415718	0.017178636	1
feature_34967	VMP1	2.150695443	0.14374185	1
feature_34990	VPS13B	4.221735499	0.081313915	1
feature_34994	VPS13D	2.204616012	0.219347583	1
feature_34998	VPS26A	1.07350952	0.065233128	1
feature_35004	VPS29	2.372505058	0.305631687	1
feature_35018	VPS41	1.07841139	0.141265497	1
feature_35019	VPS45	1.120077285	0.370160622	1
feature_35021	VPS4B	1.11272448	0.049145498	1
feature_35023	VPS51	1.094342467	0.035710868	1
feature_35026	VPS54	1.139684765	0.135721092	1
feature_35053	VTI1A	3.360231854	0.006025168	1
feature_35068	VWA8	2.138440768	0.235502552	1
feature_35075	WAC	3.792821878	0.018619839	1
feature_35087	WASF2	2.311231684	0.034798348	1
feature_35102	WASHC4	2.221772557	0.154359776	1
feature_35120	WDFY2	2.579609064	0.119957314	1
feature_35121	WDFY3	1.552667308	0.520000406	1
feature_35124	WDFY4	2.222998025	0.277807708	1
feature_35125	WDHD1	1.85903418	0.263475505	1
feature_35126	WDPCP	3.794047345	0.221372966	1
feature_35127	WDR1	1.32718129	0.144554994	1
feature_35138	WDR26	1.205860009	0.025054424	1
feature_35146	WDR37	1.367621717	0.257530315	1
feature_35149	WDR41	1.094342467	0.087798845	1
feature_35153	WDR45B	1.131106492	0.29187191	1
feature_35159	WDR49	1.365170782	0.398007365	1
feature_35169	WDR62	1.085764195	0.256277156	1
feature_35171	WDR7	1.573500256	0.250945913	1
feature_35173	WDR70	2.585736401	0.169769865	1
feature_35178	WDR76	1.268358851	0.28974245	1
feature_35215	WIPF1	1.825946558	0.081237337	1
feature_35219	WIPI2	1.138459297	0.091867617	1
feature_35246	WSB1	1.841877636	0.04013353	1
feature_35259	WWOX	7.377314282	0.107708856	1
feature_35264	WWP2	1.65438111	0.334577386	1
feature_35304	XPO1	3.720519296	0.067557966	1
feature_35307	XPO6	1.124979155	0.010773536	1
feature_35308	XPO7	1.009785211	0.04754937	1
feature_35315	XRCC4	1.519579686	0.056900162	1
feature_35454	YAF2	1.215663749	0.098660207	1
feature_35470	YEATS2	1.841877636	0.079220257	1
feature_35472	YEATS4	1.560020113	0.250210799	1
feature_35480	YIPF4	1.368847185	0.129604192	1
feature_35489	YME1L1	2.086971133	0.089569372	1

feature_35491	YPEL1	1.547765438	0.169024728	1
feature_35564	ZBTB20	3.833262305	0.156539159	1
feature_35618	ZC3H12B	1.427669624	0.068068526	1
feature_35621	ZC3H13	2.269565789	0.053648143	1
feature_35645	ZCCHC7	67.95217225	0.066733968	1
feature_35666	ZDHHC20	1.818593753	0.111113624	1
feature_35683	ZEB2	32.303323	0.529239974	1
feature_35691	ZFAND3	10.04638247	0.095584406	1
feature_35695	ZFAND6	1.38110186	0.047416557	1
feature_35715	ZFP36L1	3.604099884	0.159903052	1
feature_35716	ZFP36L2	3.751155983	0.308650021	1
feature_35740	ZFX	1.845554038	0.456703334	1
feature_35755	ZGRF1	1.334534095	0.256409167	1
feature_35777	ZMAT3	1.330857693	0.292360751	1
feature_35780	ZMIZ1	5.295245019	0.283225994	1
feature_35788	ZMYM4	2.650686178	9.29E-05	1
feature_35794	ZMYND11	1.162968647	0.104807499	1
feature_35799	ZMYND8	7.162857472	0.145930087	1
feature_35805	ZNF107	1.497521271	0.058415889	1
feature_35827	ZNF148	3.064894189	0.150984746	1
feature_35896	ZNF254	1.414189482	0.02199617	1
feature_35926	ZNF292	5.093042883	0.011645859	1
feature_35977	ZNF367	1.226692956	0.331702275	1
feature_35979	ZNF37BP	1.34678877	0.23134072	1
feature_35996	ZNF407	2.594314674	0.345475937	1
feature_36014	ZNF43	1.682566862	0.168254914	1
feature_36016	ZNF431	1.099244337	0.036928788	1
feature_36035	ZNF451	1.665410317	0.11350579	1
feature_36086	ZNF518A	1.264682448	0.209738326	1
feature_36175	ZNF609	5.318528901	0.147850446	1
feature_36205	ZNF652	1.710752614	0.226666974	1
feature_36208	ZNF654	1.297770071	0.230173499	1
feature_36270	ZNF714	1.490168466	0.017771195	1
feature_36273	ZNF718	1.844328571	0.153248435	1
feature_36304	ZNF767P.1	1.197281737	0.289342139	1
feature_36357	ZNF83	1.487717531	0.382184834	1
feature_36393	ZNF91	1.036745495	0.017061143	1
feature_36413	ZNRF1	2.689901138	0.156459065	1
feature_36414	ZNRF2	1.606587878	0.237072358	1
feature_36431	ZRANB3	1.890896335	0.097764074	1
feature_36460	ZSWIM6	4.941084915	0.016931307	1
feature_36470	ZXDC	1.116400882	0.141023761	1

Table S6: Patient characteristics.

Patient ID	Age at Diagnosis	Sex	Cytogenetics KMT2A	Further aberrations	Status
0054	70	F	KMT2A::AFF1	NA	Initial
0122	47	M	KMT2A::AFF1	+X, -9, +21	Initial
0134	43	M	KMT2A::AFF1	+22	Initial
0152	52	F	KMT2A::AFF1	Biphenotypic	Initial
0159	74	F	KMT2A::AFF1	NA	Initial

Table S7: Antibodies used for immunoblot.

Target	Brand	Cat-No	Solvent	Dilution
Revert™ Total Protein Stain	LI-COR	926-10011	-	-
p-BCL-2 Ser70	Cell Signaling	#2827	1:5 LI-COR blocking buffer:PBST	1:500
BCL-2	Cell Signaling	#15071	1:5 LI-COR blocking buffer:PBST	1:1000
MCL-1	Cell Signaling	#4572	1:5 LI-COR blocking buffer:PBST	1:1000
BCL-xL	Cell Signaling	#2764	1:5 LI-COR blocking buffer:PBST	1:1000
BAX	Cell Signaling	#5023	1:5 LI-COR blocking buffer:PBST	1:1000
BAK	Cell Signaling	#12105	1:5 LI-COR blocking buffer:PBST	1:500
BIM	Cell Signaling	#2933	1:5 LI-COR blocking buffer:PBST	1:1000
IRDye 800CW Goat anti-Rabbit IgG	LI-COR	926-32211	1:5 LI-COR blocking buffer:PBST	1:5000

Table S8 Antibodies used for intracellular flow cytometry.

Target	Brand	Cat-No	Dilution
CD148-PE	Biolegend	328708	5 µl of 200 µg/ml
CD54-PE/Cy7	Biolegend	353116	5 µl of 100 µg/ml
CD9-PE	Biolegend	312106	2 µl of 20 µg/ml
NOTCH1-APC	Biolegend	352108	5 µl of 100 µg/ml
CD26-PE/Cy7	Biolegend	302714	5 µl of 50 µg/ml
CD45-FITC	BD	345808	5 µl of 50 µg/ml
CD19-PE	BD	345777	5 µl of 12.5 µg/ml

Immunoblot: Cell lines, membrane 1

Total protein stain

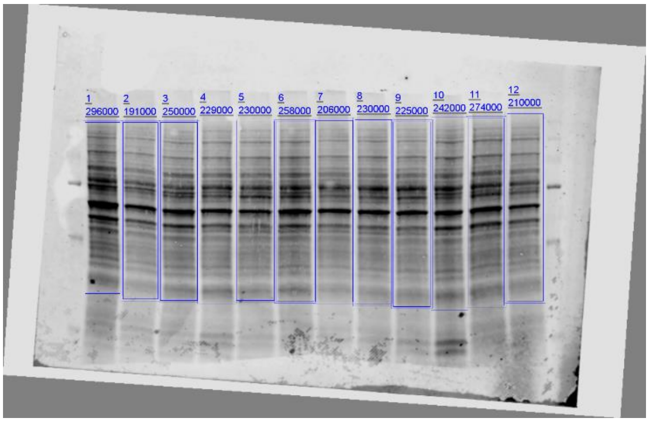

BAX (20 kDa)

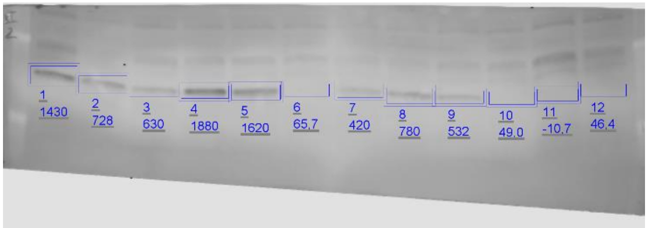

BAK (25 kDa)

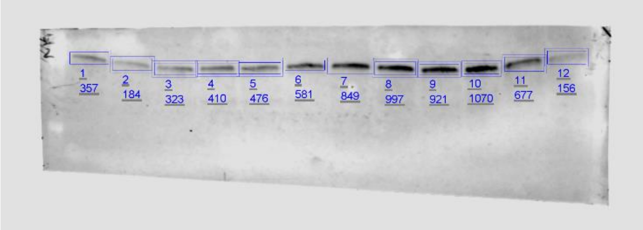

BIM
(12/15/23 kDa)

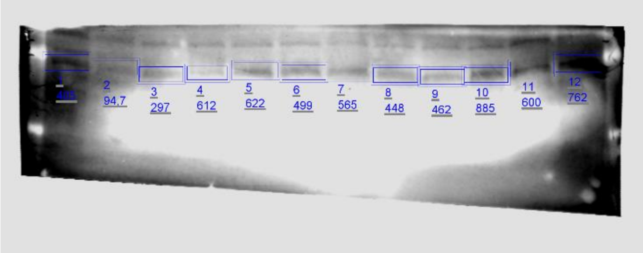

Line 1: SEM, parental
Line 2: SEM, vehicle
Line 3: SEM, VEN-resistant
Line 4: RS4;11, parental
Line 5: RS4;11, vehicle
Line 6: RS4;11, VEN-resistant
Line 7: REH, parental
Line 8: REH, vehicle
Line 9: REH, VEN-resistant
Line 10: NALM-6, parental
Line 11: NALM-6, vehicle
Line 12: NALM-6, VEN-resistant

Immunoblot: Cell lines, membrane 2

Total protein stain

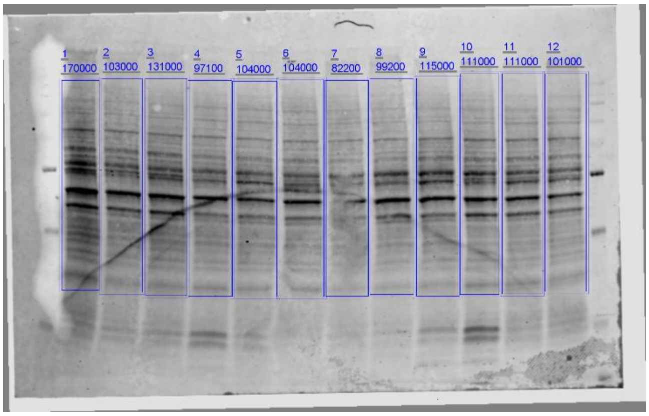

P-BCL-2 (26 kDa)

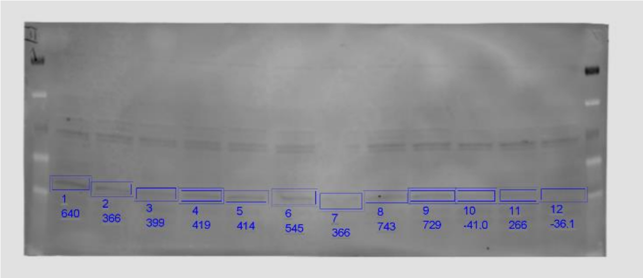

BCL-2 (26 kDa)

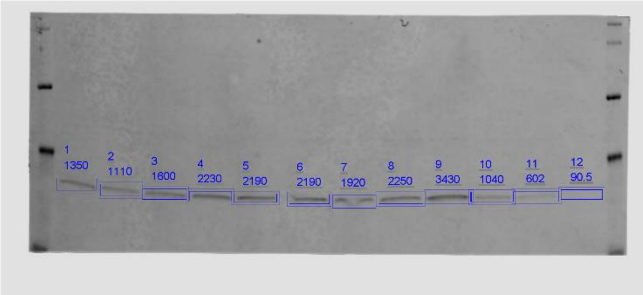

Line 1: SEM, parental
Line 2: SEM, vehicle
Line 3: SEM, VEN-resistant
Line 4: RS4;11, parental
Line 5: RS4;11, vehicle
Line 6: RS4;11, VEN-resistant

Line 7: REH, parental
Line 8: REH, vehicle
Line 9: REH, VEN-resistant
Line 10: NALM-6, parental
Line 11: NALM-6, vehicle
Line 12: NALM-6, VEN-resistant

Immunoblot: Cell lines, membrane 3

Total protein stain

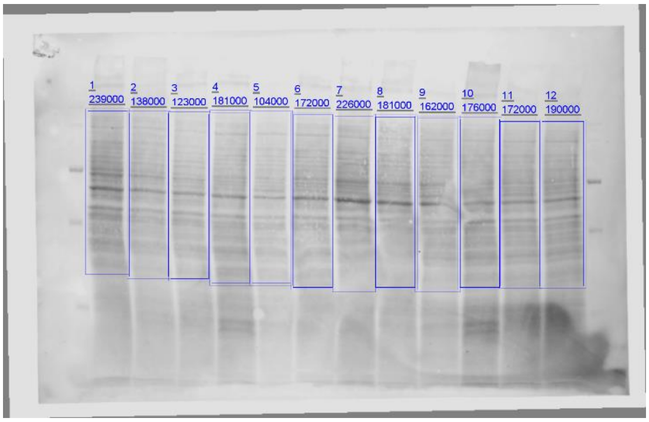

MCL-1 (40 kDa)

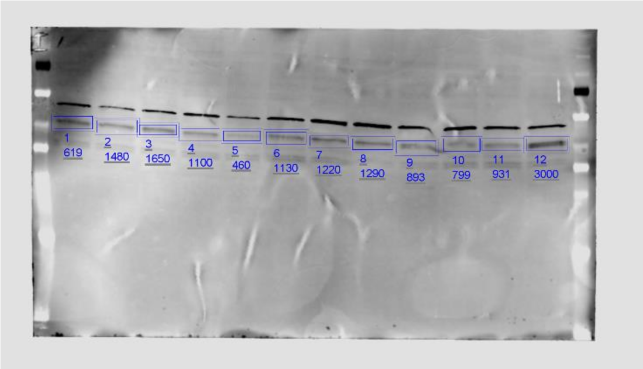

BCL-xL (30 kDa)

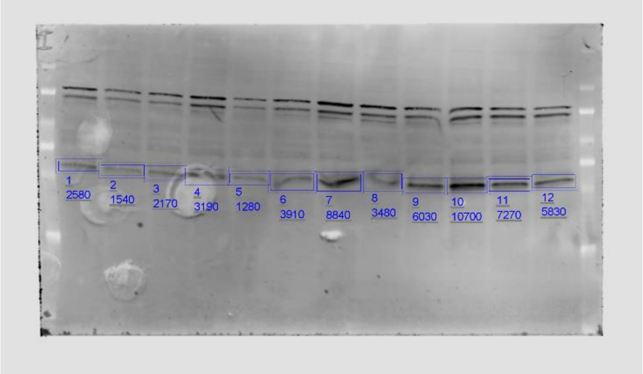

Line 1: SEM, parental
Line 2: SEM, vehicle
Line 3: SEM, VEN-resistant
Line 4: RS4;11, parental
Line 5: RS4;11, vehicle
Line 6: RS4;11, VEN-resistant
Line 7: REH, parental
Line 8: REH, vehicle
Line 9: REH, VEN-resistant
Line 10: NALM-6, parental
Line 11: NALM-6, vehicle
Line 12: NALM-6, VEN-resistant

Immunoblot: SEM-CDX, membrane 1

Total protein stain

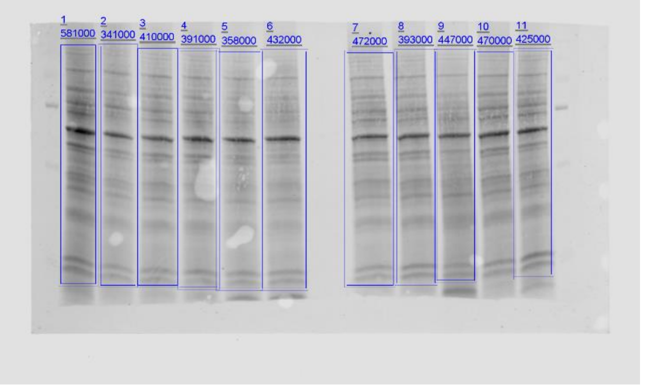

MCL-1 (40 kDa)

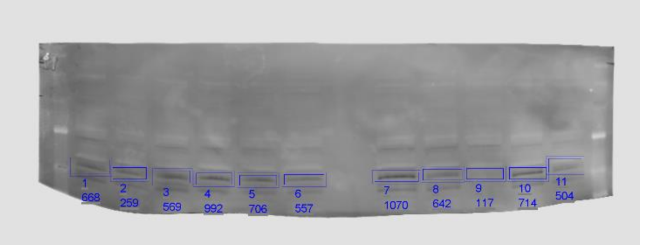

BAX (20 kDa)

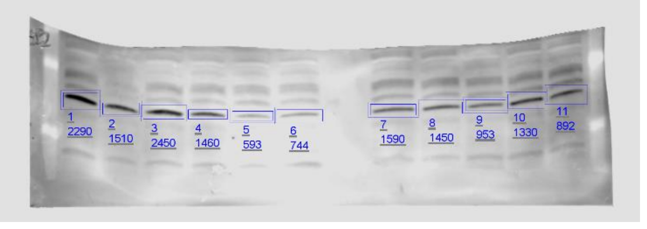

BAK (25 kDa)

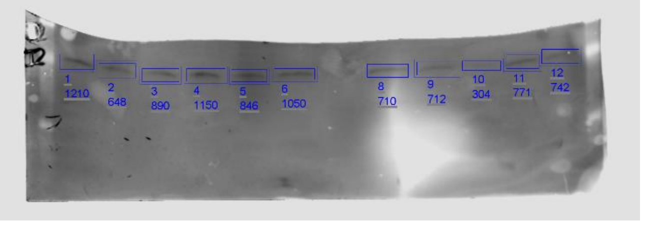

BIM (12/15/23 kDa)

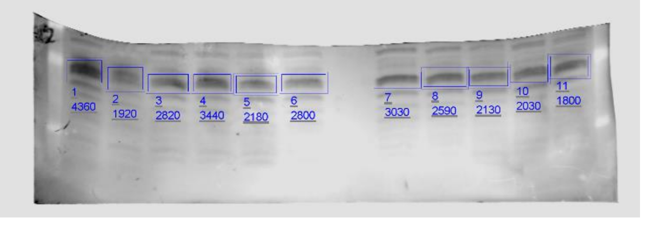

Line 1: NSG-274 (vehicle)
Line 2: NSG-275 (vehicle)
Line 3: NSG-276 (vehicle)
Line 4: NSG-289 (vehicle)
Line 5: NSG-290 (vehicle)
Line 6: NSG-293 (vehicle)
Line 7: NSG-267 (VEN-resistant)
Line 8: NSG-271 (VEN-resistant)
Line 9: NSG-273 (VEN-resistant)
Line 10: NSG-292 (VEN-resistant)
Line 11: NSG-294 (VEN-resistant)

Immunoblot: SEM-CDX, membrane 2

Total protein stain

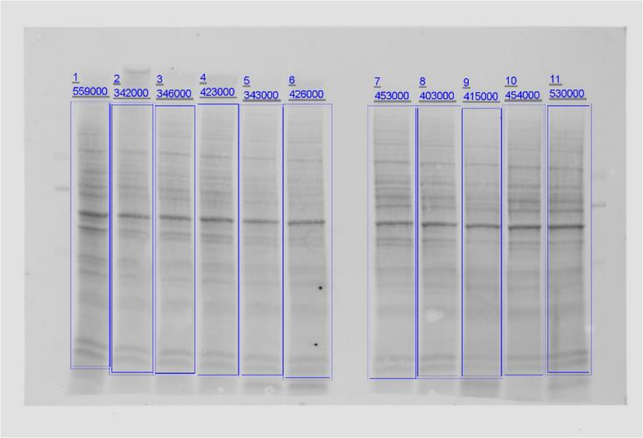

P-BCL-2 (26 kDa)

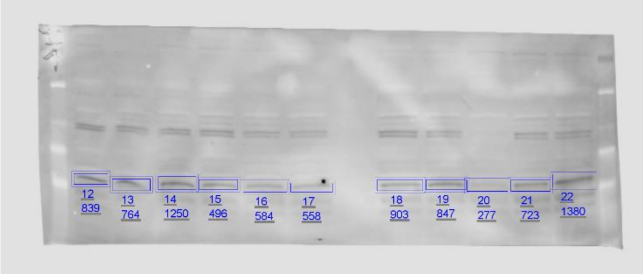

BCL-2 (26 kDa)

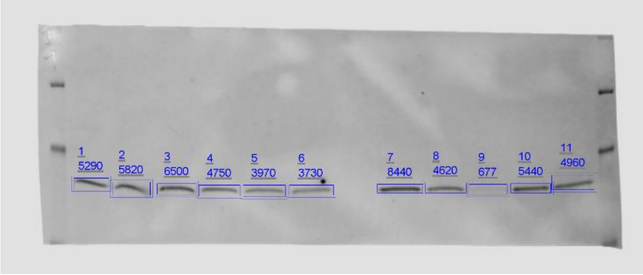

BCL-xL (30 kDa)

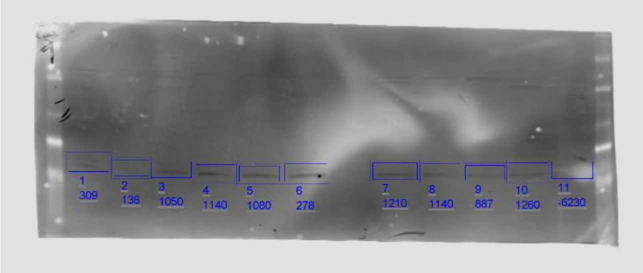

Line 1: NSG-274 (vehicle)
Line 2: NSG-275 (vehicle)
Line 3: NSG-276 (vehicle)
Line 4: NSG-289 (vehicle)
Line 5: NSG-290 (vehicle)
Line 6: NSG-293 (vehicle)

Line 7: NSG-267 (VEN-resistant)
Line 8: NSG-271 (VEN-resistant)
Line 9: NSG-273 (VEN-resistant)
Line 10: NSG-292 (VEN-resistant)
Line 11: NSG-294 (VEN-resistant)

Immunoblot: RS4;11-CDX, membrane 1

Total protein stain

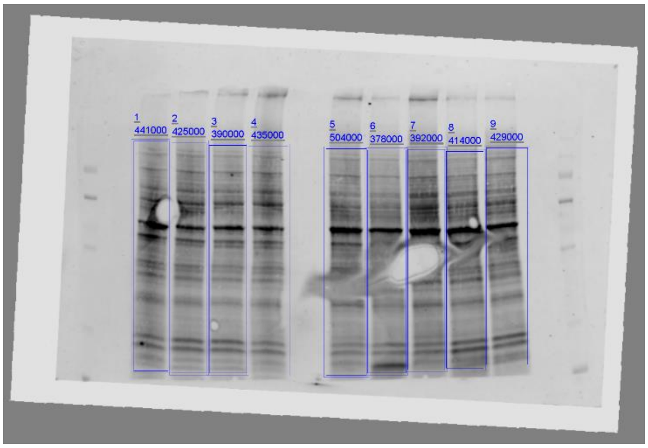

MCL-1 (40 kDa)

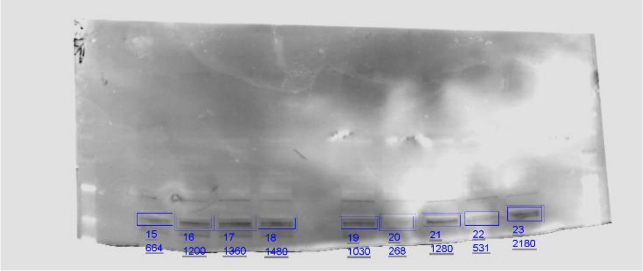

BAX (20 kDa)

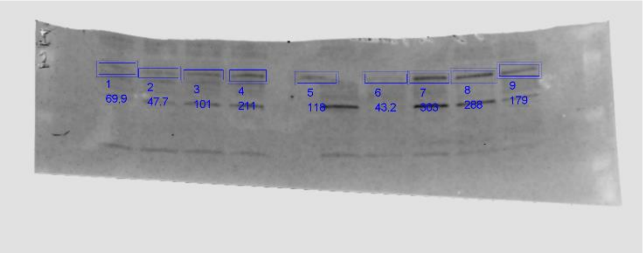

BAK (25 kDa)

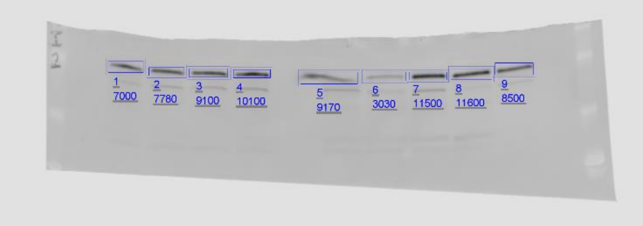

BIM
(12/15/23 kDa)

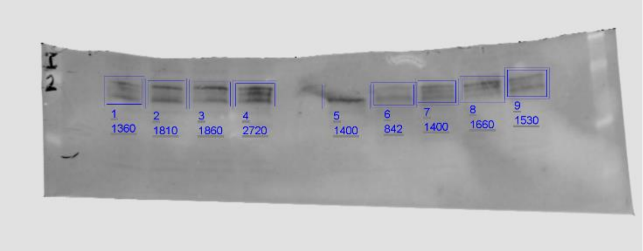

Line 1: NSG-278 (vehicle)
Line 2: NSG-281 (vehicle)
Line 3: NSG-284 (vehicle)
Line 4: NSG-288 (vehicle)

Line 5: NSG-277 (VEN-resistant)
Line 6: NSG-279 (VEN-resistant)
Line 7: NSG-282 (VEN-resistant)
Line 8: NSG-283 (VEN-resistant)
Line 9: NSG-287 (VEN-resistant)

Immunoblot: RS4;11-CDX, membrane 2

Total protein stain

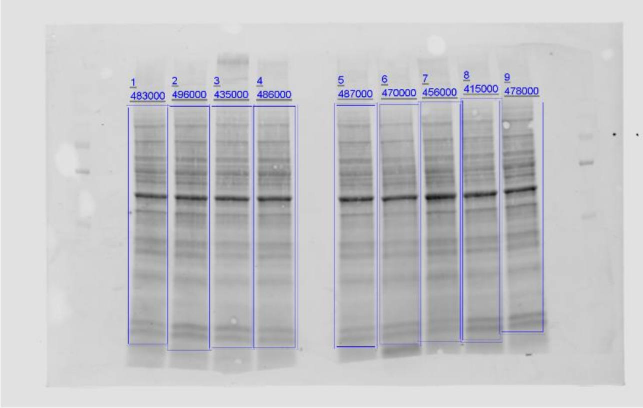

P-BCL-2 (26 kDa)

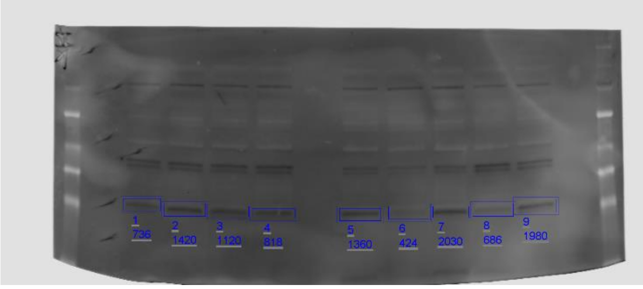

BCL-2 (26 kDa)

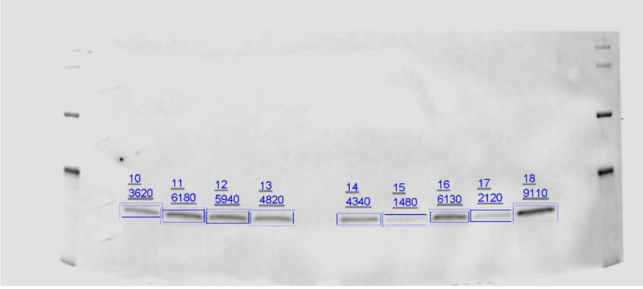

BCL-xL (30 kDa)

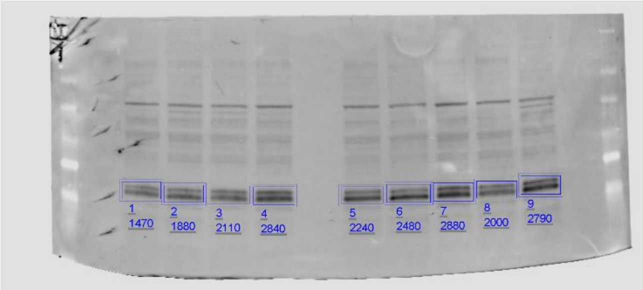

Line 1: NSG-278 (vehicle)
Line 2: NSG-281 (vehicle)
Line 3: NSG-284 (vehicle)
Line 4: NSG-288 (vehicle)

Line 5: NSG-277 (VEN-resistant)
Line 6: NSG-279 (VEN-resistant)
Line 7: NSG-282 (VEN-resistant)
Line 8: NSG-283 (VEN-resistant)
Line 9: NSG-287 (VEN-resistant)

Immunoblot: #0054, #0152, #0159 PDX, membrane 1

Total protein stain

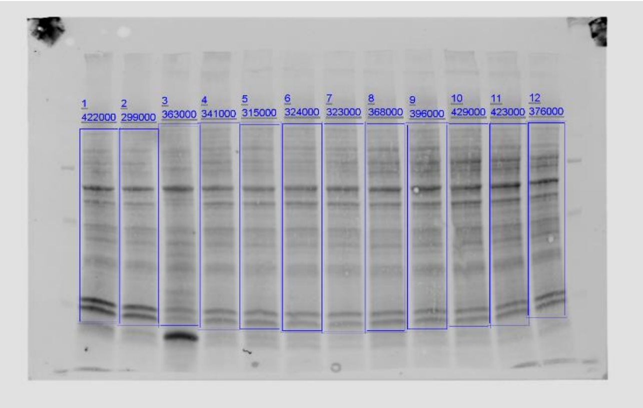

MCL-1 (40 kDa)

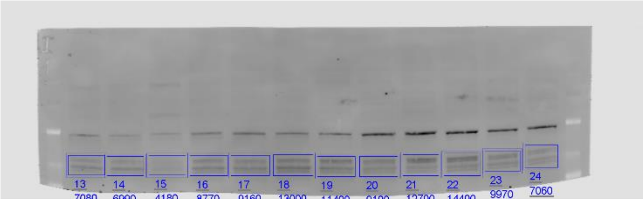

BAX (20 kDa)

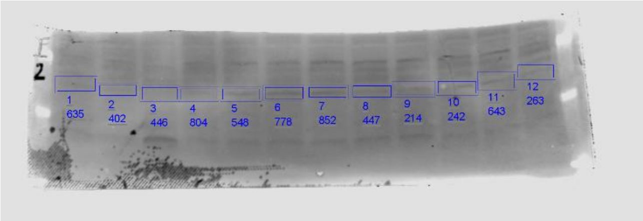

BAK (25 kDa)

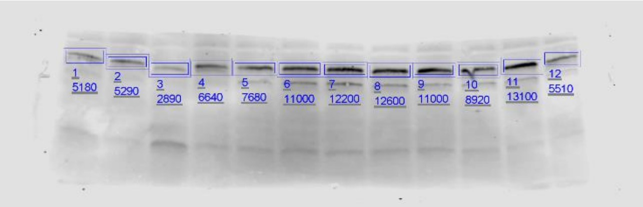

BIM (12/15/23 kDa)

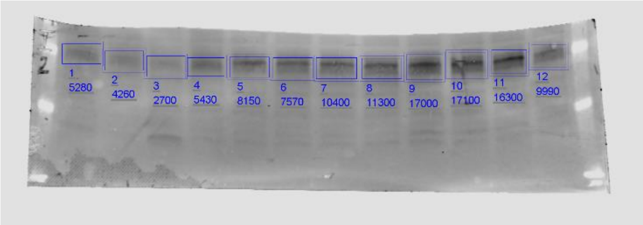

Line 1: PDX-184 (#0054, vehicle)
Line 2: PDX-185 (#0054, vehicle)
Line 3: PDX-189 (#0054, vehicle)
Line 4: PDX-187 (#0054, VEN-res)
Line 5: PDX-229 (#0152, vehicle)
Line 6: PDX-230 (#0152, vehicle)
Line 7: PDX-233 (#0152, vehicle)
Line 8: PDX-232 (#0152, VEN-res)
Line 9: PDX-217 (#0159, vehicle)
Line 10: PDX-219 (#0159, vehicle)
Line 11: PDX-222 (#0159, vehicle)
Line 12: PDX-221 (#0159, VEN-res)

Immunoblot: #0054, #0152, #0159 PDX, membrane 2

Total protein stain

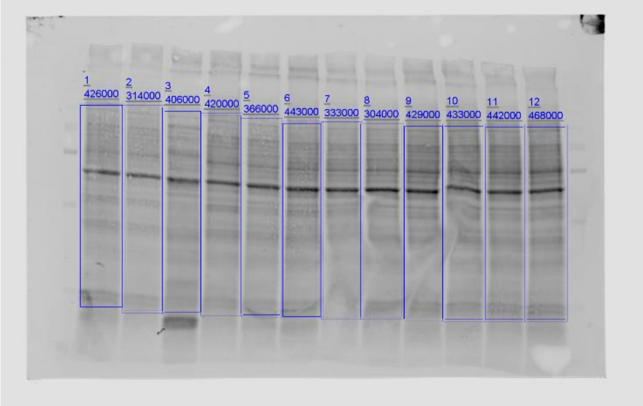

P-BCL-2 (26 kDa)

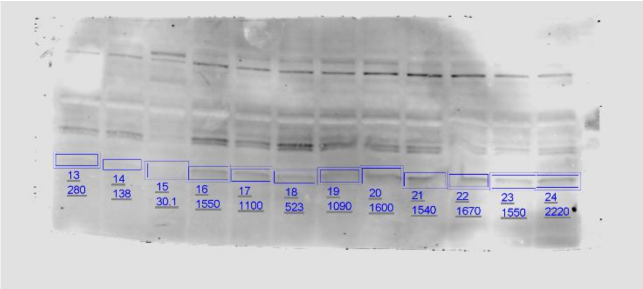

BCL-2 (26 kDa)

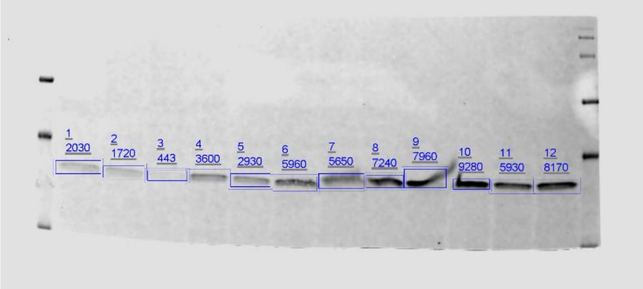

BCL-xL (30 kDa)

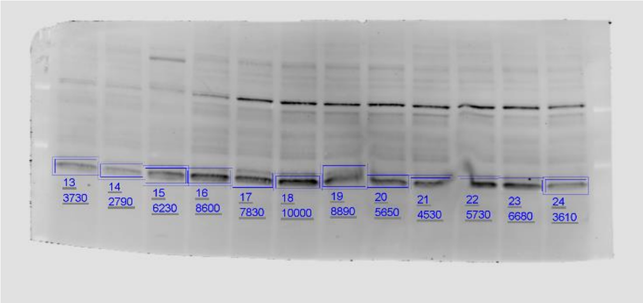

Line 1: PDX-184 (#0054, vehicle)
Line 2: PDX-185 (#0054, vehicle)
Line 3: PDX-189 (#0054, vehicle)
Line 4: PDX-187 (#0054, VEN-res)
Line 5: PDX-229 (#0152, vehicle)
Line 6: PDX-230 (#0152, vehicle)
Line 7: PDX-233 (#0152, vehicle)
Line 8: PDX-232 (#0152, VEN-res)
Line 9: PDX-217 (#0159, vehicle)
Line 10: PDX-219 (#0159, vehicle)
Line 11: PDX-222 (#0159, vehicle)
Line 12: PDX-221 (#0159, VEN-res)

Immunoblot: #0122, #0134 PDX, membrane 1

Total protein stain

MCL-1 (40 kDa)

BAX (20 kDa)

BAK (25 kDa)

BIM
(12/15/23 kDa)

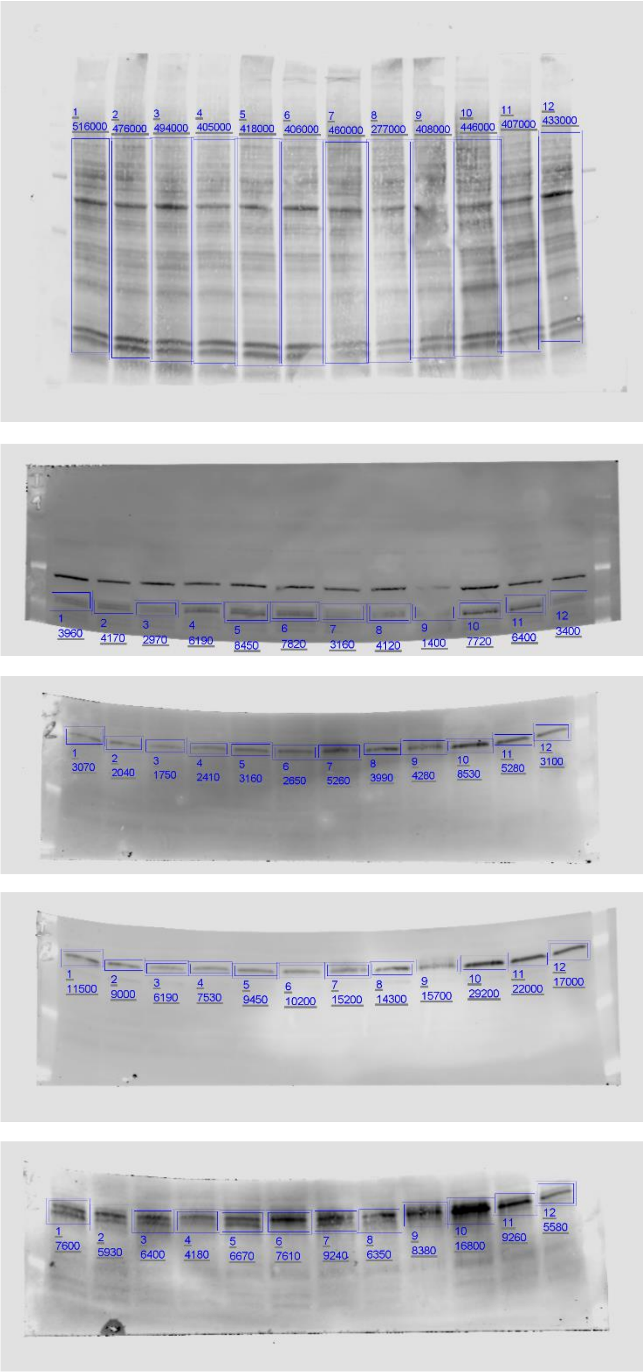

Line 1: PDX-184 (#0122, vehicle)
Line 2: PDX-185 (#0122, vehicle)
Line 3: PDX-189 (#0122, vehicle)
Line 4: PDX-187 (#0122, VEN-res)
Line 5: PDX-229 (#0122, VEN-res)
Line 6: PDX-230 (#0122, VEN-res)
Line 7: PDX-223 (#0134, vehicle)
Line 8: PDX-226 (#0134, vehicle)
Line 9: PDX-227 (#0134, vehicle)
Line 10: PDX-224 (#0134, VEN-res)
Line 11: PDX-225 (#0134, VEN-res)
Line 12: PDX-228 (#0134, VEN-res)

Immunoblot: 0122, #0134 PDX, membrane 2

Total protein stain

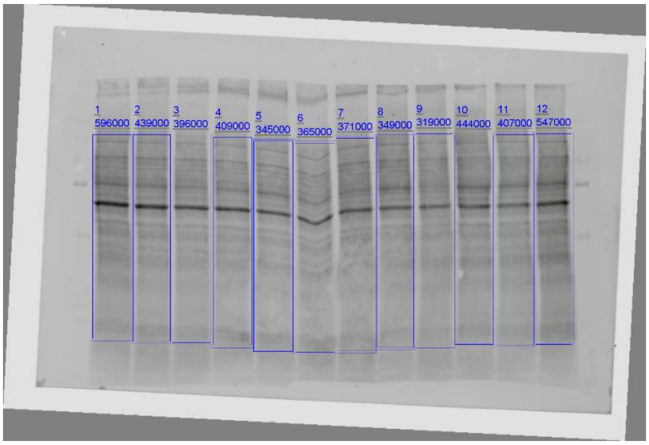

P-BCL-2 (26 kDa)

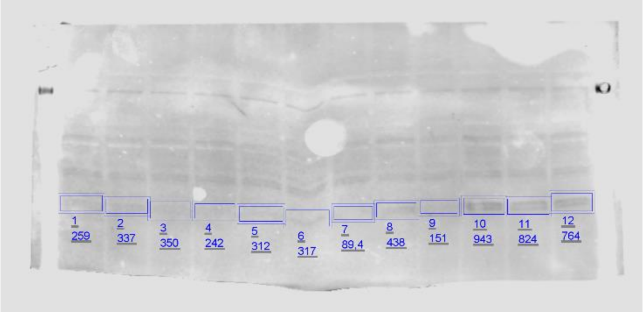

BCL-2 (26 kDa)

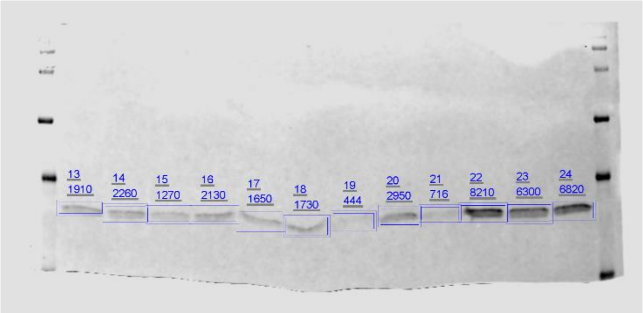

BCL-xL (30 kDa)

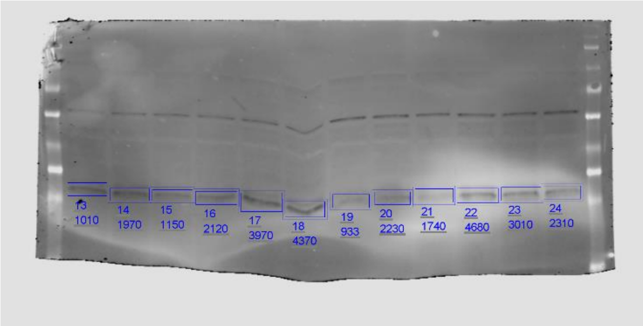

Line 1: PDX-184 (#0122, vehicle)
Line 2: PDX-185 (#0122, vehicle)
Line 3: PDX-189 (#0122, vehicle)
Line 4: PDX-187 (#0122, VEN-res)
Line 5: PDX-229 (#0122, VEN-res)
Line 6: PDX-230 (#0122, VEN-res)
Line 7: PDX-223 (#0134, vehicle)
Line 8: PDX-226 (#0134, vehicle)
Line 9: PDX-227 (#0134, vehicle)
Line 10: PDX-224 (#0134, VEN-res)
Line 11: PDX-225 (#0134, VEN-res)
Line 12: PDX-228 (#0134, VEN-res)
